# Empowering Informal Caregivers of Persons With Early-Stage Dementia by Large Language Models: Mixed Methods Evaluation

**DOI:** 10.2196/79975

**Published:** 2026-03-05

**Authors:** Huayu Zhou, Ziwei Zhu, Kyeung Mi Oh, Sungsoo Ray Hong

**Affiliations:** 1 Department of Information Sciences and Technology, College of Engineering and Computing, George Mason University, Research Hall Building, Room 211, 10401 York River Road, Fairfax, VA, 22030, United States, 1 2062291872; 2 Department of Computer Science, College of Engineering and Computing, George Mason University, Fairfax, VA, United States; 3 School of Nursing, College of Public Health, George Mason University, Fairfax, VA, United States

**Keywords:** large language model, human-computer interaction, human-AI collaboration, early-stage dementia, informal caregivers, prompt engineering

## Abstract

**Background:**

Acquiring relevant knowledge and support is essential for informal caregivers of persons with early-stage dementia, including awareness, access, and use of comprehensive resources for both persons with dementia and caregiver support. With appropriate strategies and early-stage support, informal caregivers can play a vital role in enhancing the well-being of persons with dementia and potentially slowing their progression. While large language models (LLMs) can provide easy access to caregiving knowledge, the risks, perceived challenges, and ways to improve LLM-generated responses in practice remain underexplored.

**Objective:**

In this study, we aim to (1) examine the risks and perceived challenges of using a baseline ChatGPT-4o, an internet-accessible artificial intelligence model, for dementia caregiving support and (2) understand how an enhanced version of ChatGPT-4o, equipped with up-to-date dementia caregiving knowledge, can mitigate these risks and challenges.

**Methods:**

We compiled 32 representative questions from informal caregivers seeking guidance on early-stage dementia. We developed two ChatGPT-4o conditions: C1, the publicly available baseline model, and C2, an experimental version enhanced through prompt engineering and grounded in a conceptual framework—drawn from health science and gerontology literature—to empower caregivers of individuals with early-stage dementia. Using these conditions, we generated 64 responses (32 pairs) to the questions. Twelve experts evaluated them with validated tools assessing accuracy, reasoning, clarity, usefulness, trust, satisfaction, safety, harm, and relevance. A Mann-Whitney *U* test compared the conditions. After the survey, we conducted interviews to explore experts’ perceived differences, remaining challenges, and design opportunities. Interviews were transcribed and analyzed using descriptive thematic analysis.

**Results:**

Responses in C2 showed significant improvements in 3 criteria—actionability, relevance, and perceived satisfaction—compared to C1. However, no significant differences were found in the remaining 5 criteria: response accuracy, the model’s ability to understand the question, intelligibility, trustworthiness, response safety, and perceived harm. Qualitative analysis of interviews revealed two key insights: (1) differences between baseline and experimental responses and (2) possible reasons for these differences. Twelve experts evaluated wordiness, detail, empathy, satisfaction, accuracy, relevance, and bias. Both models were considered somewhat verbose, but the experimental model’s responses were viewed as more detailed, relevant, and actionable. Accuracy appeared similar across models, yet participants reported greater satisfaction with the experimental model’s outputs.

**Conclusions:**

Results indicate that both conditions generated responses perceived as reasonable and intelligible. However, the experimental model offered more relevant, practical guidance on caregiving needs, providing specific information aligned with the 32 testing questions and actionable recommendations. This led to higher perceived satisfaction compared to the baseline model.

## Introduction

Informal caregivers’ dementia literacy plays a critical role in mitigating the progression of dementia; however, obtaining relevant knowledge and effectively applying it in real-life situations can be difficult. These challenges are further compounded by limited access to information and resources related to caregiving. The aging population is increasing rapidly; in 2024, about 6.9 million Americans aged 65 and older are living with Alzheimer’s or dementia, a number expected to double by 2060 [1]. Dementia is a leading contributor to older mortality, with one in three individuals over age 85 dying with the disease [2,3]. Informal caregivers, who support persons with dementia, typically family members, face barriers such as limited English proficiency [4], inadequate health insurance [5], low income [6], and social stigma [7], which hinder access to essential dementia knowledge and effective care strategies [8].

The lack of accessible, accurate, and personalized knowledge contributes to both ineffective care and substantial socioeconomic burdens [9,10]. In 2019, the worldwide cost of dementia care was US $1.3 trillion [11], including medical expenses, social care, and informal caregiving, with US $196 billion spent on direct medical costs in the United States alone in 2020 [12]. Beyond finances, caregivers and persons with dementia experience declines in quality of life, as persons with dementia lose autonomy and dignity [13,14] and caregivers face heightened psychological and physical stress [15], yet few tools provide tailored guidance for informal caregivers, highlighting a critical gap in support.

Addressing informal caregivers’ limited access can empower them to manage their responsibilities more effectively [16] and strengthen their emotional resilience [17] by recognizing early symptoms [18], managing initial behavioral changes effectively [8], and establishing consistent routines to reduce confusion and anxiety [19]. Additionally, informed informal caregivers can create a stable and safe environment that reduces stress and enhances emotional well-being while also engaging persons with dementia in cognitive and social activities that help maintain mental function, support independence, and foster a sense of purpose [20]. To further support informal caregivers, culturally tailored support can provide personalized guidance respecting caregivers’ cultural and language needs [21-23], enhancing the accessibility and relevance of dementia care information.

Although large language models (LLMs) can be an effective technological aid for providing dementia-related knowledge [24,25], there have been concerns about applying these tools to informal caregivers. For instance, using LLMs often fails at providing valid information [26], relevant answers based on personalized context, nonactionable suggestions [27,28], answers that lack genuine contextual understanding and empathy [29], and answers with bias related to age, disability, and race, thereby contributing to disparities in care delivery [30]. While studies started applying ChatGPT in various health care applications, including patient interaction [31], clinical diagnosis support [32], telehealth services [33], health education [34], personalized health advice [35], and broader health promotion efforts [36], several limitations and risks must be addressed to ensure the safe and effective integration of LLMs into dementia caregiving. Finally, it is worth noting that the use of LLMs in caregiving raises privacy and data security concerns; without robust safeguards, sensitive information may be vulnerable to unauthorized access or misuse.

While prior research has extensively documented the burden of dementia and the challenges faced by informal caregivers, fewer studies have examined the potential of artificial intelligence (AI)–driven digital health tools to support caregiving. Recent work has begun to explore the application of LLMs in this domain. For example, Kim et al [27] analyzed digital caregiving strategies for Alzheimer disease and related dementias and highlighted how AI-driven tools can align caregiving tasks with disease stage–specific needs. Pérez-Esteve et al [37] demonstrated that GPT-4o models can provide tailored guidance and reduce caregiver errors, although they do not yet replace professional instruction. Similarly, Shi et al [38] developed Carey, a GPT-4o–based chatbot to provide informational and emotional support to Alzheimer disease and related dementias caregivers, identifying key caregiver needs to inform AI design. Hasan et al [39] introduced ADQueryAid, a conversational AI system leveraging LLMs with retrieval-augmented knowledge to deliver personalized support. Additionally, Chien et al [40] showed that LLMs could accurately identify high-burden caregivers in long-term care settings, highlighting their utility in caregiver assessment. Building on these developments, our study investigates how broadly accessible everyday LLMs can both challenge and benefit informal caregivers, aiming to improve their practical utility in real-world caregiving contexts.

**Figure 1.**
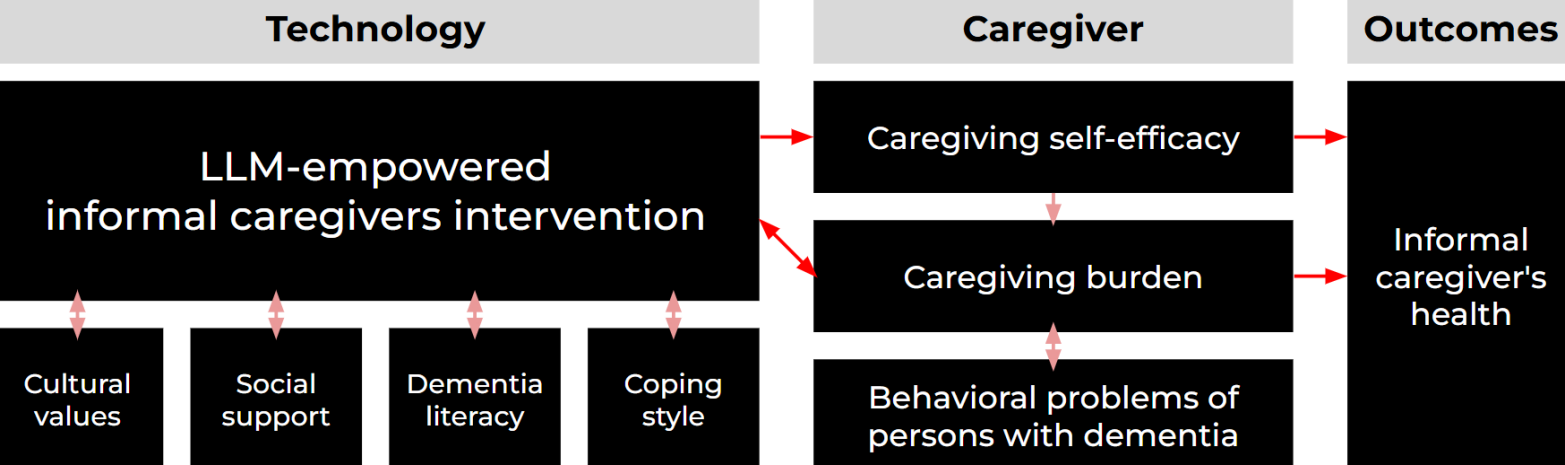
Conceptual framework integrating technology, caregiver factors, and outcomes in dementia caregiving. The framework integrates the sociocultural stress and coping model [41] and the self-efficacy model [42] for caregivers to illustrate the dynamic interactions among contextual, psychological, and technological factors. It comprises three main components: (1) technology, referring to LLM-empowered interventions that support informal caregivers; (2) caregiver, encompassing factors such as caregiving self-efficacy, caregiving burden, and behavioral challenges of persons with dementia; and (3) outcomes, focusing on the health and well-being of informal caregivers. LLM: large language model.

In this study, we used ChatGPT, a publicly available LLM model that is considered the most advanced one at the time of the study, as our baseline condition (C1). In parallel, we designed an experimental condition (C2) using a prompt-engineered ChatGPT-4o model, enhanced through a conceptual framework shown in Figure 1, which is built upon our comprehensive review of literature in geriatrics and gerontology, with a specific focus on dementia caregiving.

Our study uniquely explores how widely LLMs affect informal caregivers, highlighting both the challenges and opportunities they present for enhancing caregiving support in everyday, real-world contexts. Then, this study is guided by the following four research questions: (RQ1) What risks and challenges do informal caregivers encounter when using everyday LLMs (eg, ChatGPT-4o) for dementia care support? (RQ2) How can ChatGPT be enhanced through prompt engineering with dementia-related knowledge, caregiving skills, and culturally relevant supports? (RQ3) To what extent does the enhanced ChatGPT improve caregivers’ experiences in terms of (1) actionability, (2) relevance, (3) perceived satisfaction, (4) response accuracy, (5) model’s ability to understand the question, (6) intelligibility, (7) trustworthiness, and (8) response safety and perceived harm? (RQ4) What are the implications of these findings for the future design of AI tools tailored to caregiver–person with dementia dyads?

## Methods

### Study Design

A mixed methods study using surveys and interviews was conducted via Zoom to evaluate a prompt-engineered ChatGPT-4o compared to baseline ChatGPT-4o—an internet-accessible AI tool—based on the evaluation criteria listed above. The study aimed to assess its potential to support informal caregivers of persons with early-stage dementia.

**Figure 2.**
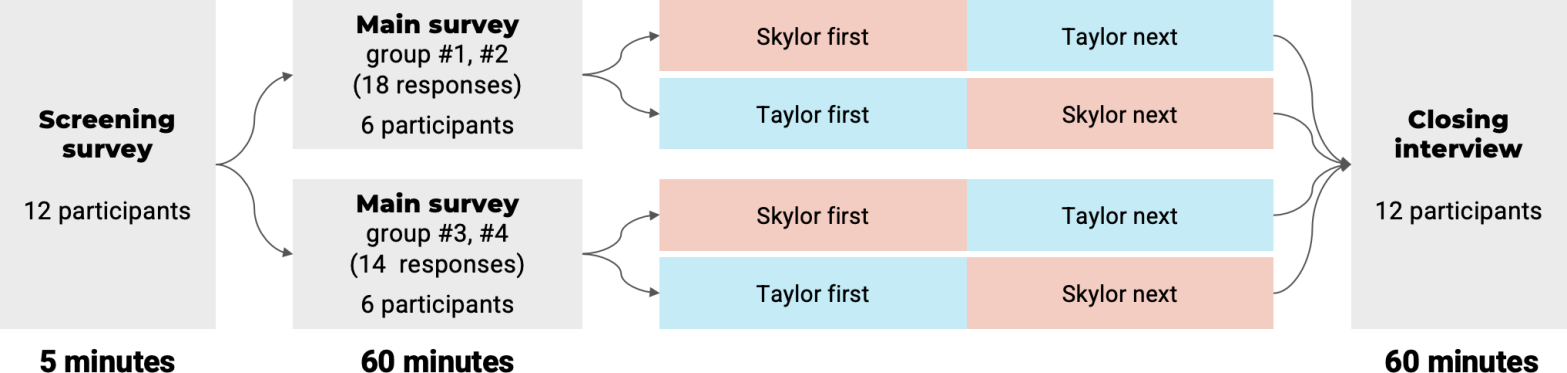
Study flow diagram illustrating the mixed methods design. The study began with a 5-minute screening survey. Participants who met eligibility criteria were assigned a unique ID and participated in a 1-hour quantitative session. Participants in groups 1 and 2 (n=6) evaluated 18 pairs of test scenario–based LLM responses, while those in groups 3 and 4 (n=6) evaluated 14 pairs. Under each test scenario, two types of LLM responses were labeled “Skylor” and “Taylor.” Finally, all participants completed a 1-hour semistructured qualitative interview. LLM: large language model.

Figure 2 shows a clear study flow diagram applied to summarize the proposed mixed methods. The overall study pipeline proceeded as follows. (1) Each participant first completed a brief 5-minute screening survey to collect professional background information. Participants who passed the screening were assigned a unique ID number used to record their results from both the quantitative (1 hour) and qualitative (1 hour) studies. (2) In the 1-hour quantitative study, participants 1‐6 were divided into group 1 and group 2. Each group evaluated 18 testing scenarios containing 18 pairs of LLM-based responses while completing corresponding evaluation surveys. Similarly, participants 7‐12 were divided into group 3 and group 4, and each group evaluated 14 testing scenarios with 14 pairs of LLM-based responses, also accompanied by quantitative evaluation surveys. (3) Finally, during the 1-hour qualitative study, all participants completed a semistructured interview consisting of general and specific questions. The corresponding study materials are covered in Multimedia Appendix 1.

### Prompt Engineering Design

We used ChatGPT-4o (September 2024 till November 2024). To prepare C2, we compiled a dataset of 90 question-and-answer pairs to inform the prompt engineering of C1. These pairs were developed across 9 domains. Four of them are key domains identified in the conceptual framework: cultural values, social support, dementia literacy, and coping style. To create the question-and-answer pairs, we collaborated with community outreach lay health workers with over a decade of experience working with persons with dementia and their caregivers. Drawing on their expertise, these professionals generated questions commonly asked by informal caregivers of persons with early-stage dementia within each of the 4 domains. Subsequently, an experienced nurse practitioner with over a decade of clinical practice responded to the generated questions. The answers were grounded in evidence-based guidelines from credible organizations, including the Alzheimer’s Association [43], the National Institutes of Health, the National Institute on Aging [44], the Retirement Research Foundation [45], and the Korean Medical Center [46]. Following the initial drafting, one of the authors (KMO) with expertise in nursing and gerontology reviewed the responses to ensure their accuracy, clarity, and relevance. This iterative process resulted in a finalized dataset of 90 question-and-answer pairs attached in Multimedia Appendix 2. Selected examples are provided in Table 1.

**Table 1.** Selected examples from the 90 question-and-answer pairs across 4 domains: cultural values, social support, coping style, and dementia literacy.

	Questions	Answers	Domains
Q1	I am Hispanic and my spouse was just diagnosed with Alzheimer’s disease—how can I be sure I’m getting the care I need for my Hispanic spouse?	The Alzheimer’s Association has partnered with several agencies within different cultures to make sure care and help are provided appropriately. Your first resource would be to reach out to the National Hispanic Council on Aging and you can find them here: [47].	Cultural values
Q11	Are there places I can go to socialize? I feel so isolated since my spouse was diagnosed with Alzheimer’s disease. I just need to meet some other people—I don’t even have time to go meet my friends.	Yes, there are places you can go and people you can talk to. You can start with the support groups online that you can find here: [48]. You can find community resources close to you here: [49]. You can also call the Alzheimer’s Association’s hotline and talk to an actual person that can help you 800.272.3900. Or you can click this link for resources specific to the National Capital Area (NCA) [50].	Social support
Q21	What is Alzheimer’s disease? What do I tell my family?	Alzheimer’s disease is the most common form of dementia—more information here: [51]. Regarding what to tell your family, remember Alzheimer disease is nobody’s fault and there is help available. For help telling your family you can start here: [52].	Dementia literacy
Q31	My spouse is my best friend and the one I go to with everything, and now I feel like I can’t because my spouse is what I’m worried about, my spouse is why I need coping help. I don’t know what to do.	It is hard when your person is the one you have concerns and worries about. There are resources available to help you. There are online support groups, local activities and support groups, and a helpline available 24/7. You can find the online support groups here: [48]. You can find local activities and supports groups here: [49] and the helpline is 800.272.3900.	Coping style

Prompt engineering is essential for adapting ChatGPT to specific contexts, such as supporting informal caregivers of individuals with early-stage dementia. Studies have shown that prompt design significantly influences the quality, relevance, and accuracy of AI-generated responses. For instance, Li et al [53] found that structured, role-specific prompts improved ChatGPT’s performance in dementia care scenarios. Without such guidance, general-purpose models may produce vague or misleading information, particularly in sensitive health-related contexts. Parmanto et al [54] highlighted that unguided prompts often result in irrelevant or unreliable outputs. Prompt engineering helps mitigate these issues by narrowing the model’s focus, reducing hallucinations, and encouraging context-specific, empathetic responses. A study by Cheng et al [55] further demonstrated that prompt-engineered AI can provide personalized, culturally sensitive support tailored to caregiver needs and the stages of dementia. Ultimately, prompt engineering enhances clarity, aligns output with user intent, and bridges the gap between general AI capabilities and the specialized demands of caregiving.

In our work, after collecting the 90 question-and-answer pairs, we proceeded to generate the corresponding 90 prompts for C2 by applying prompt engineering techniques to C1. The 90 prompts for C2 are provided in Multimedia Appendix 3. These examples show the details of how the prompts were generated with context-related information. In designing these prompts, we drew on principles of prompt engineering grounded in five fundamental factors [56]: (1) clarity and specificity—ensuring that prompts are unambiguous and clearly stated; (2) user intent—aligning the prompt with the user’s needs and the desired output; (3) model understanding—considering the strengths and limitations of ChatGPT to craft prompts that maximize its capabilities while mitigating its weaknesses; (4) domain specificity—incorporating relevant terminology and context to guide the model toward more accurate and relevant responses; and (5) constraints—explicitly defining requirements such as response length or format.

### Generating Responses From C1 and C2 for Evaluation

To select a natural and realistic set of questions that informal caregivers may ask frequently in practice, we collaborated with two experts: a community outreach lay health worker and an academic researcher, both with over a decade of experience working with persons with dementia and their caregivers. They were independent of the community caregiving lay health workers involved in the prompt engineering design, minimizing potential bias during the evaluation. They were asked to generate 32 test questions commonly posed by caregivers of persons with early-stage dementia across 4 domains: cultural values, social support, coping style, and dementia literacy. The dementia literacy domain includes 4 subcategories: causes and characteristics, health risks and promotion, communication and behavior, and care considerations. The 32 testing questions are provided in Multimedia Appendix 4.

Using the 32 questions, we generated 64 LLM responses: 32 from C1 and another 32 from C2. Specifically, for each question, we manually entered it into the LLM interface to initiate response generation. Once the LLM’s textual output was displayed, we collected it directly as a single response. Each response was saved and assigned a unique identifier corresponding to its input question.

### Participants

In January 2025, purposive sampling was used to recruit 12 participants through a screening survey assessing eligibility based on predefined inclusion and exclusion criteria. Eligible participants included informal caregivers of persons with dementia, as well as health care professionals or community outreach program leaders with at least 1 year of experience working with persons with dementia or their caregivers. Recruitment continued until qualitative data saturation was reached, that is, we intended to keep recruiting participants as long as each new case enhanced our understanding; recruitment would end once additional participants no longer offered new information. Moreover, Caine [57] conducted the analysis which revealed that 12 participants appeared most frequently among CHI papers—effectively becoming a de facto community norm for small-scale human-computer interaction (HCI) user studies. This was not presented as the optimal number, but rather as the most common practice (a cultural standard in CHI research). Furthermore, these participants are considered content experts and invited to evaluate LLM-generated responses using validated tools measuring accuracy, reasoning, clarity, usefulness, trust, satisfaction, safety, harm, and relevance.

### Data Collection Procedures

From the 64 LLM responses generated by C1 and C2, content experts conducted evaluations using two methods: a quantitative survey and a qualitative follow-up semistructured interview conducted via Zoom. Each session lasted about 2 hours on average—1 hour for the survey and 1 hour for the follow-up interview. Participants received a US $50 Amazon gift card in appreciation of their time.

The 12 experts were divided into 4 groups of 3 to ensure deeper and more focused discussion, as reviewing all 32 questions together could limit individual focus and engagement. Of the 32 testing questions or scenarios, 6 are from the cultural values domain, 7 from social support, 5 from coping style, and 14 from dementia literacy. For the evaluation surveys, groups 1 and 2 assessed 18 scenarios drawn from the cultural values, social support, and coping style domains, while groups 3 and 4 evaluated the remaining 14 from the dementia literacy domain.

Selected examples are provided in Table 2.

**Table 2.** Selected examples from the 32 test questions assigned to groups across 4 domains: cultural values, social support, coping style, and dementia literacy.

	Group ID	Test question example	Domains
Q5	G1&G2	I live in Virginia and am a caregiver for my family member with dementia. What local resources or organizations in Spanish can provide me with support, whether for caregiving or guidance on managing the situation?	Cultural values
Q10	G1&G2	As a caregiver in Virginia, I'm looking for local resources or organizations that can provide support for caregivers like me. What options are available to help me navigate this journey?	Social support
Q11	G1&G2	I’m looking to connect with other caregivers in my community for support. Are there any peer support groups or opportunities to meet others who are going through similar experiences? Can you suggest any online support groups or forums for caregivers?	Social support
Q14	G1&G2	I would like to connect with someone who is experiencing similar situations. I feel overwhelmed as I am responsible for taking care of both my children and my father at home. How can I ensure I take care of myself as well?	Coping style
Q2	G3&G4	My husband’s behavior has been changing lately, and I’m not sure if it’s just because he’s getting older or if it could be something like dementia. He forgets things, gets confused, and sometimes doesn’t act like himself. What are the early signs of dementia, and how can I tell if this is just aging or something more serious?	Dementia literacy
Q8	G3&G4	I live in Fairfax County and I’m looking for activities or therapies in Korean that might help slow down my husband’s dementia. Are there any programs or services available that have been proven to help with this?	Dementia literacy
Q10	G3&G4	My husband has been acting differently, and sometimes he gets upset or does things that are hard to understand. How can I handle these behaviors, especially if it’s because of dementia?	Dementia literacy
Q14	G3&G4	My husband has been showing signs of mild memory problems, like forgetting names or where he put things, but I’m not sure if this is just part of getting older or something more serious. I’ve heard about mild cognitive impairment (MCI), and I’m worried that it might get worse. What are the risks if his MCI progresses to full dementia, and how can I prepare for what might come next?	Dementia literacy

In the survey, experts reviewed responses in a within-subjects design with a counterbalanced order to eliminate order effects. To avoid bias, we used gender-neutral labels—“Skylor” for C1 and “Taylor” for C2—and withheld information about how these responses were generated. The two responses to test question no. 18 from the coping style domain are presented as an example in Figure S1 in Multimedia Appendix 5. More examples are shown in Table 2. After the survey, we conducted semistructured interviews to gain a deeper understanding of (1) when and why experts felt that Taylor’s or Skylor’s responses were not fully satisfactory; (2) when and why experts felt that responses from Taylor or Skylor were superior to others; (3) what general challenges experts encountered during the interviews; and (4) given the rapid progress in LLMs, which aspects future technology should prioritize to deliver higher-quality caregiving knowledge to informal caregiver–person with dementia dyads. The interview questions are presented in Multimedia Appendix 6.

### Evaluation Measures for Quantitative Data

We adapted our evaluation measures from a literature-based framework for human evaluation of LLMs in health care [58]. This multidimensional framework assessed accuracy, understanding and reasoning, clarity and coherence, usefulness and supportiveness, trust and confidence, overall satisfaction, safety and harm, and relevance. Each dimensional measure was rigorously validated in previous studies [34,35,59-68] and assessed using a single item, except for safety and harm, which was evaluated with two separate items. Building on this foundation, we developed our own expanded set of evaluation measures, as shown in Table 3.

**Table 3.** The 9 evaluation items and their corresponding dimensions adapted from a literature-based framework for large language model assessment in health care.

	Evaluation items	Dimensions
Q1	The response is accurate.	Accuracy of responses [61,63,64]
Q2	The response demonstrates correct reading comprehension, showing that it understands the question.	Understanding and reasoning [65]
Q3	The language used in the response is clear and easy to understand.	Clarity and coherence [61,68]
Q4	The response includes actionable advice or resources.	Usefulness and supportiveness [59,60]
Q5	The response is reliable and based on credible medical and scientific sources.	Trust and confidence [66]
Q6	I am satisfied with the chatbot's response overall.	Satisfaction [34]
Q7	The response provided is balanced and free from bias.	Safety [67]
Q8	The response contains no potentially harmful information.	Harm [35]
Q9	The response provides relevant answers.	Relevance [60-62]

In total, 9 items were rated on a 7-point Likert scale, where 1=strongly disagree and 7=strongly agree; higher scores indicate greater perceived quality.

### Data Analysis

#### Quantitative Analysis

Histograms were generated for each 7-point evaluation item. To compare C1 and C2, Mann-Whitney *U* tests were conducted for each criterion. Moreover, the comparisons of the mean and SD values were reported for each evaluation criterion as well. Additionally, all analyses were performed in Python using pandas, matplotlib, seaborn, and scipy.

#### Qualitative Analysis

The interviews were audio-recorded and transcribed. We then conducted an iterative qualitative coding process [69,70] to analyze the data. One of the authors (HZ) independently read the transcription and conducted coding by tagging specific text in the transcriptions. As the author coded multiple transcriptions, she started finding commonalities and discrepancies between transcriptions and wrote her findings in the analytic memos. As the author started finding no new topics for codes and memos, she started building themes and categories together. Finally, after reviewing all her coded text snippets, memos, and insightful quotes, she refined the final structure and details of the findings. Every analysis has been done without the help of AI, other than transcription.

### Ethical Considerations

This study was reviewed and approved by the institutional review board at George Mason University (STUDY00000288). All participants provided informed consent prior to participation. To protect privacy and confidentiality, all personal identifiers were removed, and audio recordings were securely stored on password-protected servers accessible only to the research team. Participation was entirely voluntary, and participants could withdraw from the study at any time without penalty. Each participant received a US $50 honorarium as compensation for their time and contributions. All study procedures adhered to the ethical principles outlined in the Declaration of Helsinki and complied with JMIR and COPE (Committee on Publication Ethics) standards for research involving human participants.

## Results

### Study Participants

Twelve content experts participated, each with over a decade of professional experience and substantial involvement with older adults with dementia and their caregivers, including informal caregivers. Their professions included registered nurses, nurse practitioners (psychiatric-mental health, geriatric, and family health), physicians, social workers, and a community outreach program director. Participant details are provided in Table 4.

**Table 4.** Demographic and professional characteristics of the 12 content experts in this study.

Participant ID	Gender	Age (years), range	Profession	Primary work settings
P1	Female	45‐54	Registered nurse	Academic institution
P2	Female	45‐54	Physician	Academic institution
P3	Female	55‐64	Physician	Academic institution
P4	Female	35‐44	Registered nurse	Academic institution
P5	Female	45‐54	Social worker	Academic institution
P6	Female	55‐64	Registered nurse	Academic institution
P7	Female	35‐44	Family nurse practitioner	Academic institution
P8	Female	65 or older	Informal caregiver	Retired
P9	Female	65 or older	Informal caregiver	Retired
P10	Female	55‐64	Social worker	Academic institution
P11	Male	35‐44	Psychiatric-mental health nurse practitioner	Community-based organization
P12	Female	35‐44	Geriatric nurse practitioner, research nurse specialist	National Institutes of Health Clinical Center

### Survey Results

Our evaluation comprised 9 items (Q1-Q9) listed in Table 3, each rated on a 7-point Likert scale (1=strongly disagree, 7=strongly agree). We collected 192 responses for each evaluation item: 36 for cultural values, 42 for social support, 30 for coping style, and 84 for dementia literacy domains. Next, we used Mann-Whitney *U* tests to compare the baseline and experimental models on each evaluation item. For each comparison, the *U* statistic and *P* value were calculated to determine if there was a statistically significant difference between the 7-point Likert scale distributions, indicating whether the evaluation responses under the two conditions differed.

**Figure 3.**
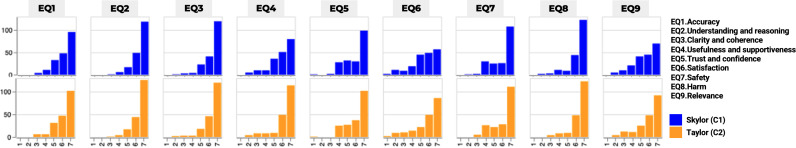
Comparison of answer score distributions for evaluation items Q1-Q9 across 4 domains (192 responses): cultural values, social support, coping style, and dementia literacy. Blue histograms represent responses under the baseline condition (C1), and orange histograms represent responses under the experimental condition (C2). Statistically significant differences were observed for Q4 (usefulness and supportiveness), Q6 (overall satisfaction), and Q9 (response relevance), indicating that C2 outperformed C1 in these three aspects (Mann-Whitney *U* test; *P*<.05).

The key research findings across all 4 domains—cultural value, social support, coping style, and dementia literacy—are presented in Figure 3. In the conceptual framework, that is, Figure 1, we can tell that the data used for prompt engineering are from all the domains. Specifically, the blue histograms represent the distribution of answers under the baseline condition, while the orange histograms represent those under the experimental condition. Notably, the Mann-Whitney *U* test identified statistically significant differences between the two conditions for evaluation items Q4, Q6, and Q9 (*P*<.05). The statistical details such as *U* statistics and exact *P* values are reported in Table S1 in Multimedia Appendix 7.

Overall, among the 9 evaluation items, responses generated by the prompt-engineered ChatGPT (ie, the experimental model, C2) outperformed those from the general ChatGPT (ie, the baseline model, C1) on items Q4 (providing actionable advice or resources), Q6 (overall satisfaction with the response), and Q9 (relevance of the answers). In the multidimensional evaluation framework, Q4 is a measure that decides if the response includes actionable advice or resources; Q6 is a measure that determines if the participants are overall satisfied with the given response; additionally, Q9 measures if the given response provides relevant answers. Therefore, this comparison result across all 4 domains says that C2 is better than C1 in terms of usefulness, supportiveness, and satisfaction, as well as relevance.

Comparisons of answer-score distribution in terms of mean and SD values for each evaluation criterion are reported in Tables S1 and S2 in Multimedia Appendix 8. After analyzing Table S1, we observed that, overall, C2 achieved slightly higher mean scores than C1 across most evaluation criteria (EQ1-EQ5, EQ7-EQ9), indicating a modest advantage in performance. However, C1 outperformed C2 on EQ6 (5.44 vs 4.58), representing the largest negative difference (−0.86), while the largest positive difference favoring C2 was observed for EQ4 (+0.40). These results suggest that although C2 generally provides stronger responses, C1 demonstrates notable strength on specific criteria. After analyzing Table S2, we observed that, overall, the variability of scores was similar across most evaluation criteria. The exception was EQ6, where C2 exhibited substantially higher SD (2.16 vs 1.49), indicating less consistent performance, while differences for all other criteria were minimal (<0.12). In summary, across all evaluation criteria, C2 generally achieved slightly higher mean scores than C1, except for EQ6 where C1 outperformed C2. Overall variability was comparable between conditions, though C2 showed notably higher SD on EQ6, indicating less consistent performance for that criterion. These results suggest that while C2 tends to provide marginally better answers on average, its consistency varies across specific evaluation dimensions.

**Figure 4.**
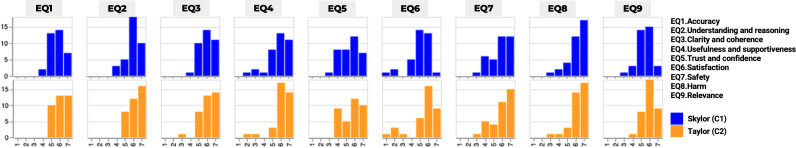
Comparison of answer score distributions for evaluation items Q1-Q9 within the cultural values domain (36 responses). Blue histograms represent responses under the baseline condition (C1), and orange histograms represent responses under the experimental condition (C2). Statistically significant differences were observed for Q6 (overall satisfaction) and Q9 (response relevance), indicating that C2 outperformed C1 in these two aspects (Mann-Whitney *U* test; *P*<.05).

Moreover, within the cultural values domain, which is one of the four domains in the conceptual framework in Figure 1 and provides 36 responses for evaluation, the Mann-Whitney *U* test indicated statistically significant differences between C1 and C2 for evaluation items Q6 (overall satisfaction with the chatbot’s response) and Q9 (relevance of the response), with both *P* values falling below .05. The key research findings are presented in Figure 4. The statistical details such as *U* statistics and exact *P* values are reported in Table S2 in Multimedia Appendix 7. In the multidimensional evaluation framework, Q6 is a measure that determines if the participants are overall satisfied with the given response; additionally, Q9 measures if the given response provides relevant answers. Therefore, this comparison result across the cultural values domain says that C2 is better than C1 in terms of satisfaction and relevance.

Comparisons of answer-score distribution in terms of mean and SD values for each evaluation criterion are reported in Tables S3 and S4 in Multimedia Appendix 8. After analyzing Table S3, we observed that, for the cultural values domain, C2 consistently achieved higher mean scores than C1 across all evaluation criteria, with differences ranging from modest (+0.16 on EQ7) to more pronounced (+0.53 on EQ6). This indicates that C2 provided generally stronger responses than C1 in addressing questions related to cultural values. After analyzing Table S4, we observed that, in the cultural values domain, C2 consistently achieved higher mean scores than C1 across all evaluation criteria, indicating generally stronger performance. Variability was comparable for most criteria, though C2 showed slightly higher SD on EQ6 (1.57 vs 1.25), suggesting less consistent responses for that criterion, while differences for other criteria were minimal.

**Figure 5.**
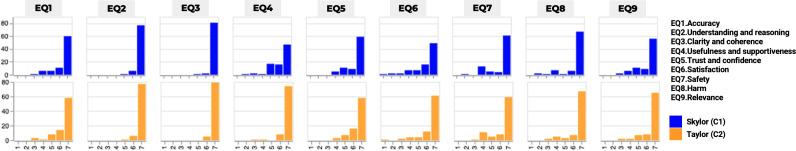
Comparison of answer score distributions for evaluation items Q1-Q9 within the dementia literacy domain (84 responses). Blue histograms represent responses under the baseline condition (C1), and orange histograms represent responses under the experimental condition (C2). Statistically significant differences were observed for Q4 (usefulness and supportiveness) and Q6 (overall satisfaction), indicating that C2 outperformed C1 in these two aspects (Mann-Whitney *U* test; *P*<.05).

Furthermore, within the dementia literacy domain, which is one of the four domains in the conceptual framework in Figure 1 and provides 84 responses for evaluation, the Mann-Whitney *U* test revealed statistically significant differences between C1 and C2 for evaluation items Q4 (the response includes actionable advice or resources) and Q6 (overall satisfaction with the chatbot’s response), with both *P* values below .05. The key research findings are presented in Figure 5. The statistical details such as *U* statistics and exact *P* values are reported in Table S3 in Multimedia Appendix 7. In the multidimensional evaluation framework, Q4 is a measure that decides if the response includes actionable advice or resources; Q6 is a measure that determines if the participants are overall satisfied with the given response. Therefore, this comparison result across the dementia literacy domain says that C2 is better than C1 in terms of usefulness, supportiveness, as well as satisfaction.

Comparisons of answer-score distribution in terms of mean and SD values for each evaluation criterion are reported in Tables S5 and S6 in Multimedia Appendix 8. After analyzing Table S5, we observed that in the dementia literacy domain, C2 achieved slightly higher mean scores than C1 for most evaluation criteria, particularly on EQ4 (6.82 vs 6.21) and EQ9 (6.57 vs 6.32). For several criteria, such as EQ1 and EQ2, mean scores were identical or nearly identical, indicating that both conditions performed similarly on certain aspects of dementia literacy. Overall, C2 demonstrated a modest advantage in performance across this domain. After analyzing Table S6, we observed that, in the dementia literacy domain, C2 achieved slightly higher mean scores than C1 for most evaluation criteria, with the largest improvements observed on EQ4 (6.82 vs 6.21) and EQ9 (6.57 vs 6.32). Variability was generally comparable between the two conditions, although C2 exhibited lower SDs on several criteria, including EQ4 (0.60 vs 1.08) and EQ9 (0.92 vs 1.09), indicating more consistent responses. Overall, these results suggest that C2 provided marginally stronger and more consistent performance in addressing dementia literacy questions.

### Semistructured Interview Results

Immediately after completing the survey, each participant was invited to a follow-up interview. Before answering the interview questions, they reviewed the test scenarios and the corresponding model responses they had seen during the survey.

#### Response Differences Between C1 and C2

Qualitative insights from the semistructured interviews provided a deeper understanding of participants’ perspectives on the differences between responses from C1 and C2. First, the interviews focused on two central questions: (1) What are the key differences between responses generated under the baseline and experimental conditions? and (2) What factors might explain these differences? Participants discussed aspects such as wordiness, detail, empathy, satisfaction, accuracy, relevance, and potential bias. Overall, both models’ responses were viewed as somewhat wordy, but the experimental model’s replies were generally considered more detailed, relevant, and actionable. Accuracy was acknowledged in both, yet participants reported higher satisfaction with the experimental model. These findings, organized by overarching themes with related subthemes and illustrative participant quotes, are presented in Table 5.

**Table 5.** Themes and illustrative quotes summarizing participant feedback on differences between C1 and C2 responses.

Theme and subtheme	Supporting quote	Participant ID
Response delivery difference		
Detailed	“The same observation but Taylor’s answers were more detailed, links to resources, information about the location and the transportation.”	P7
Empathy	“For the first six questions, Taylor’s answers seem to be more empathetic.”	P6
Response content difference		
Actionable	“For Taylor, it sorts of provided more actionable ways to get the information that one was desiring.”	P5
Satisfied	“Taylor’s provided phone numbers, web addresses, links while Skylor’s didn’t. Taylor provided more links, more useful ways for someone to actually take the action.”	P10
Accurate	“Taylor’s was kind of like, find a doctor or find a provider. And Skylor’s were like, no, go to your general, you know either your Pcp, or your general practitioner.And Taylor’s was a little bit more ambiguous. As to, you know. Great to find a doctor that’s not terribly helpful. I know I need to do that. But you know which one do I go to see? So, I felt like again, Taylor’s was a bit more ambiguous. Skylor’s was a little bit more direct.”	P2
Relevant	“Taylor seemed to have provided more accurate information and more specific information, or more relevant information based on the scenario in terms of clear and easier to understand.”	P6
Biased	“I think there were some points where, like your questions asked about like just bias between, you know, it was this one bias, or that one bias, and just in general, of course, coming from nurse practitioner lens like. I tend to like or favor responses that say, healthcare provider versus doctor, or primary care physician, or whatever, or just include that, as like a little bit more general, and it seems like it wasn’t necessarily consistent that, like Skylor, use this language and Taylor use this terminology like it. It kind of was interchangeable. I think like at some points where someone would say doctor and the others would say healthcare provider. Maybe it seemed like in the beginning, Taylor was saying, like healthcare provider, a little bit more. Which I always prefer.”	P9

In summary, the qualitative findings map closely to the evaluation framework. Participants’ perceptions of greater detail and relevance in the experimental model reflect usefulness and supportiveness and clarity and coherence. Observations of accuracy align with the framework’s accuracy dimension, while concerns about potential bias relate to safety and harm and trust and confidence. Comments on wordiness pertain to clarity and coherence, and reports of higher satisfaction and empathy connect to overall satisfaction and supportiveness. Overall, these insights demonstrate how participants’ experiences substantiate the framework’s multidimensional evaluation of LLM responses.

#### Implications

##### Reflections on Interview Design and Future LLM Applications

In addition to exploring participants’ perspectives on the differences between responses from C1 and C2, we included two additional questions aimed at identifying limitations of the current interview design and eliciting participants’ views on the potential of LLM-based tools to empower informal caregivers of persons with dementia. Specifically, we asked the following questions: (1) What general challenges did participants encounter during the interviews? and (2) What future opportunities do they envision for using LLMs in this area of research? The following sections present key implications derived from the interview findings.

##### Implication 1: Future Interview Design

Two main themes emerged regarding the limitations of the current interview design, as shown in Table 6. The first theme, response content, includes 5 subthemes. The second theme, testing scenario design, reflected concerns that the interview scenarios were not tailored specifically for dementia care. Therefore, interview results revealed that participants valued empathy and actionable advice but found responses overly verbose and lacking perspectives from social workers or community-based resources. Quantitative ratings confirmed that concise, emotionally attuned answers were associated with higher satisfaction, linking linguistic style to usability outcomes. These insights point to the need for future interviews to employ dementia-specific scenarios and include interdisciplinary expertise when shaping prompts and evaluation criteria. Incorporating diverse professional viewpoints and assessing dimensions such as emotional tone, cognitive load, and decision support can refine both data collection and model evaluation, guiding the design of more realistic and user-centered caregiving dialogues.

**Table 6.** Themes and illustrative quotes summarizing participant feedback on limitations of the current interview design.

Theme and subtheme	Supporting quotes	Participant ID
Response content limitation		
Wordy	“The volume of words needs to be cut down.”	P8
Lacking social worker contact information	“So, what I thought lacked from both of them were. There was very little information regarding talk to a social worker, which is something that they both really missed out. On neither of them indicated that these people should get in contact with a social worker, and how to do that.”	P2
Lacking coping strategy	“And then none of them talked about. Really seeing your own provider for coping strategies, as well as oftentimes the caregivers become anxious or depressed, and so treatment for that, either by, you know, medicinally or with therapy.”	P2
Lacking care team information	“Usually, people who have dementia have a care team made up of a primary care provider, a social worker, and potentially a pharmacist … So, the social worker and the primary care, or the neurologist were glaringly missing from almost every answer.”	P11
Bias on guidance provided	“In the responses, there are suggestions such as ‘this center’ you can visit for help, or ‘that organization’ you can reach out for assistance, or ‘that medical provider’ we can contact, so, how could I trust this, how could I believe that there is no preference chosen by the system?”	P3
Test scenario design limitation		
Not specifically designed for dementia	“Taylor already assumes that it is true that they have dementia, or they have a cognitive decline. Did they tell the system (ChatGPT) already? They know that it was a cognitive decline which I'm assuming. That was the basis of the conversation with the system.”	P5

##### Implication 2: LLM-Based Applications

The results highlight the potential for LLMs to provide personalized, empathetic, and context-aware support for informal caregivers. Building on these findings, future systems could integrate multimodal data from wearables and caregiver inputs to tailor recommendations dynamically—such as suggesting rest during stress or prompting social connection during isolation. Embedding such intelligence within existing caregiver platforms would facilitate coordination among families and professionals. To ensure responsible deployment, systems must incorporate transparent data practices, bias mitigation, and ethical oversight aligned with clinical and privacy standards. These principles can guide the evolution of LLM-driven caregiver tools from research prototypes to trustworthy, scalable digital companions.

## Discussion

### Principal Findings

The study addressed the overarching aim of exploring how LLMs such as ChatGPT can support informal dementia caregivers by systematically evaluating both their limitations and potential benefits. In relation to RQ1, our findings indicated that while baseline LLM responses were generally clear and reasonable, they often lacked sufficient relevance and contextual depth for caregiving situations, reflecting current challenges and risks in everyday use. Addressing RQ2 and RQ3, the experimental model—enhanced through prompt engineering with dementia-specific and culturally informed caregiving knowledge—significantly improved the actionability, relevance, and practical value of responses, leading to higher informal caregiver satisfaction. These results demonstrate that tailored prompt strategies can meaningfully enhance LLM usability and reliability in informal caregiving contexts. In connection to RQ4, the findings underscore the importance of integrating domain-specific knowledge, cultural sensitivity, and safety considerations into future AI tool design to better support informal caregiver–person with dementia dyads in real-world settings.

### Interpretation and Contextualization of Findings

Overall, the findings suggest that both the baseline and experimental models produced responses that were clear and reasonable. However, the experimental model delivered more relevant and practical guidance for informal caregiving, offering detailed information aligned with the 32 test questions and actionable suggestions. As a result, participants reported greater satisfaction with the experimental model than with the baseline one. Additionally, our study found that the qualitative insights complemented the quantitative results, revealing nuanced user perceptions that provided a deeper understanding of how caregivers evaluated response clarity, relevance, and emotional tone. These perceptions further illuminate the emotional and cognitive contexts in which caregivers engage with AI-generated content, offering insight into the types of communication that foster trust and reassurance.

Caregiving in the context of cognitive impairment often involves significant emotional burden [71], uncertainty, and a need for reassurance [72,73]. Participants valued responses that provided accurate information with empathy and compassion, resembling communication within supportive caregiving networks. Consistent with prior work showing that emotionally attuned messaging enhances engagement and trust [74,75], our findings underscore that tone and empathy are integral to digital health communication. Nonetheless, recent studies caution that AI-generated empathy can appear inconsistent or superficial, potentially affecting user trust. Our results extend this discussion by demonstrating that even subtle tone variations in LLM responses can shape caregivers’ perceptions of credibility and emotional support, emphasizing both the promise and responsibility of emotionally intelligent design.

### Comparison With Prior Work

Recent research has increasingly explored LLMs as tools to support dementia caregiving. Parmanto et al [54], for instance, introduced CaLM, a retrieval-augmented system that delivers context-specific guidance to family caregivers of persons with dementia. Similarly, the DEMENTIA PLAN framework [76] integrates LLMs with knowledge graphs to combine practical care recommendations and emotional support for persons with mild dementia. Other work has used LLMs to analyze clinical documentation for assessing caregiver burden, showing improved accuracy over traditional text-analysis approaches [40]. However, findings across these studies remain mixed—while many report promise in enhancing personalization and empathy, others caution about biases, limited generalizability, and potential overreliance on model-generated information. Addressing such variability is essential for understanding how LLMs can be responsibly deployed in real caregiving contexts.

Our study extends this emerging literature by providing a quantitative, within-subject comparison between an experimental LLM-based caregiving support model and a baseline condition across multiple evaluation domains. Although we did not benchmark C2 directly against retrieval-augmented or knowledge-graph–based systems such as CaLM or DEMENTIA PLAN, our approach differs in focusing on interaction-level validation—empirically assessing caregivers’ perceived actionability, relevance, and satisfaction rather than optimizing information retrieval or structured knowledge integration. This distinction underscores the model’s emphasis on practical impact and caregiver-centered outcomes. By linking user feedback to specific caregiving needs, our findings highlight how conversational tuning and prompt design can translate advanced language capabilities into tangible, emotionally attuned support for informal dementia caregivers, while also delineating future pathways for integrating retrieval or knowledge-graph components in more comprehensive systems.

Prior studies indicate that LLMs can deliver emotionally attuned responses when guided by tailored prompting or fine-tuning. Wang et al [77] developed a fine-tuned GPT-2 trained on dementia caregiver therapy transcripts that emulated natural, emotionally resonant dialogue. Lee et al [78] found that multiple LLMs, including GPT-4, produced responses rated as more empathic than human-written ones in sensitive scenarios. A systematic review by Sorin et al [29] similarly confirmed that LLMs can demonstrate cognitive empathy toward users’ emotional cues. Nonetheless, recent work cautions that empathy quality varies with training data, prompt design, and context. Our qualitative results extend this literature by showing that empathy, tone, and contextual relevance are perceived by caregivers as essential features of supportive AI communication in dementia care.

### Implications for Future AI Tool Design

This study highlights the potential of LLMs, specifically ChatGPT-4o, to support informal caregivers of individuals in the early stages of dementia. By combining prompt engineering with a carefully curated dataset, we tailored AI-generated responses to better meet caregivers’ informational and emotional needs—an area often overlooked in traditional digital health tools.

A key strength of this work lies in its interdisciplinary innovation. In the medical domain, our approach demonstrates how LLMs can be adapted to deliver context-sensitive support, offering caregivers timely guidance and emotional reassurance. This is particularly valuable in early-stage dementia care, where decision-making is complex and emotionally charged. LLMs can also serve as accessible knowledge hubs, helping caregivers navigate challenges and risks while identifying areas for improvement in AI-generated communication.

From an HCI perspective, our use of prompt engineering contributes to the development of more intuitive, adaptive, and inclusive interfaces. By aligning AI responses with user expectations and emotional tone, we enhance usability and trust, two critical factors in the adoption of health technologies. This interdisciplinary application bridges technical innovation with human-centered design, advancing both fields toward more intelligent and empathetic digital health systems.

While these contributions highlight the study’s methodological and practical strengths, several limitations should also be acknowledged.

### Limitations

First, the prompt generation methodology was manually designed, which may limit scalability and reproducibility. Future research should explore automated or data-driven prompt optimization techniques to improve consistency and efficiency. Pathways toward scalable prompt engineering include methods that move beyond manual prompt crafting toward data-driven and adaptive optimization. Automated prompt optimization can iteratively refine prompt structures using feedback signals or performance metrics, reducing reliance on human trial-and-error. Fine-tuning approaches, in which LLMs are adapted using domain-specific conversational data, can further align system responses with the contextual and emotional nuances of caregiving interactions. Additionally, retrieval-augmented generation techniques can dynamically incorporate up-to-date, evidence-based caregiving information from external databases or knowledge repositories. Together, these strategies enable more consistent, interpretable, and scalable LLM performance across diverse caregiving scenarios, supporting sustainable deployment in real-world digital health environments.

Second, the use of the public ChatGPT interface limited our ability to control key model parameters such as memory and maximum output length, which may affect consistency and reproducibility across sessions. This constraint also restricted opportunities for systematic tuning and integration within the experimental setup. In future work, transitioning to an application programming interface (API) or a self-hosted model will enable more precise control over system configurations, logging, and parameter settings. Such an approach would enhance reproducibility, support iterative optimization, and facilitate seamless integration into real-world caregiving platforms where reliability and personalization are critical.

Third, the sample size in our study (12 content expert participants) was small, which might raise concerns about statistical power for Mann-Whitney *U* tests. However, Caine [57] reviewed 5 years of CHI proceedings, tallied participant numbers, and found that 12 participants was the modal (most common) sample size, reflecting a community norm rather than an evidence-based standard. In addition, the expert sample was predominantly composed of nurses and physicians, with only a small number of informal caregivers represented. This professional weighting may limit the extent to which the evaluation captures the lived experiences, daily challenges, and nuanced perspectives of caregivers who provide continuous, hands-on support to individuals with cognitive impairment. Consequently, the findings may reflect a more clinical than experiential understanding of user needs.

Future research should aim to balance professional and informal perspectives by including a greater number of family caregivers, as well as allied care professionals such as social workers, occupational therapists, and community health aides. These participants can provide valuable insights into the social, emotional, and logistical dimensions of caregiving, thereby enriching system design and ensuring that interventions are grounded in both professional expertise and authentic caregiver experience. Additionally, except for ChatGPT, at least one more LLM model, such as Gemini, could be applied as another baseline for more robust comparative evaluation.

Taken together, these limitations highlight areas for methodological refinement, broader participant inclusion, and technical enhancement in future work. Addressing these considerations will strengthen the reliability, generalizability, and practical impact of LLM-based interventions for informal dementia caregivers, laying the groundwork for more robust, scalable, and caregiver-centered digital health solutions.

### Conclusions

#### Future Directions for LLM-Supported Dementia Caregiving

This study demonstrates the potential of LLMs to support informal caregivers of individuals with dementia through emotionally attuned, context-specific communication. By applying prompt engineering within a medical HCI framework, we tailored AI responses to better address caregivers’ informational and emotional needs. Findings from 12 expert participants provide preliminary insights into how such systems might enhance caregiver support, though these results should not be generalized beyond this initial evaluation.

Beyond this exploratory evaluation, the study offers a framework for integrating AI, HCI, and health care, highlighting how intelligent digital tools can be designed to meet the complex cognitive, emotional, and informational needs of informal caregivers. Rather than presenting a scalable solution, this work lays the groundwork for more rigorous, large-scale validation of empathetic, context-aware digital health interventions. Future research should examine real-world impact on caregiver knowledge, well-being, and decision-making, while leveraging technical advances—such as transitioning to the ChatGPT API—to overcome current limitations and enable deeper integration with diverse datasets.

Considering the broader implications of this work alongside insights from the literature [79-81], we propose 5 directions through which future design can enhance LLM-driven empowerment for informal caregivers and persons with dementia.

#### Direction 1: Considering Social Dynamics and Resources

Designing future LLM-based agents to support informal caregivers of individuals with early-stage dementia requires moving beyond a dyadic caregiving model and considering a broader network of care [82-84]. These agents should facilitate coordination among primary care physicians, neurologists, social workers, and local community organizations, recognizing that dementia care is inherently collaborative. Designing with this extended care team in mind can help ensure that the agent’s outputs align with multidisciplinary care goals and reduce the informational fragmentation caregivers often face.

#### Direction 2: Advancing Actionability Through Biometrics

It is essential to incorporate diverse behavioral and perceptual data about the person with dementia to contextualize LLM-generated guidance. Informal caregivers make decisions based not only on static health records but also on evolving patterns, such as changes in mobility, sleep cycles, or daily stress levels. Future systems should be designed to integrate such multimodal signals, enabling LLMs to generate more personalized and situationally aware recommendations that align with real-time conditions.

#### Direction 3: Conversational Tone and Manner Matter

The tone and structure of LLM-generated content must be carefully refined to support caregivers’ emotional and cognitive load. Informal caregivers, who often operate under high stress, may find verbose or abstract language overwhelming. LLMs should adopt a succinct, step-by-step, and conversational manner of delivery that facilitates comprehension and action. Designing for interactional clarity can help transform generic advice into practically actionable microinterventions.

#### Direction 4: Integrating Up-to-Date Dementia-Related Resources With Personal Context

LLM-based agents must be grounded not only in a person’s medical history but also in the latest clinical and community-based dementia care practices. As dementia care evolves, outdated or generalized information can lead caregivers astray. Future systems should incorporate mechanisms for continuously updating their knowledge base with validated sources, ensuring that recommendations [85-89] remain aligned with current standards of care.

#### Direction 5: Privacy-Minded Conversation

Finally, as LLMs are positioned to support caregiving teams, privacy must be treated as a first-class design concern. Stakeholders may have different sensitivities regarding what information is shared or withheld—for instance, a caregiver may wish to conceal emotional burnout from a physician, or a person with dementia may prefer to limit what their adult children can access. Designing privacy-aware groupware requires mechanisms for nuanced control over information disclosure, aligned with the preferences and boundaries of each stakeholder.

Collectively, these directions underscore the broader implications of LLM-based interventions: when thoughtfully designed and ethically implemented, such systems can strengthen LLM-driven empowerment by enhancing informal caregivers’ decision-making, emotional resilience, and access to reliable knowledge—ultimately fostering a more responsive, connected, and compassionate dementia care ecosystem.

## Supplementary material

10.2196/79975 Multimedia Appendix 1 Study materials.

10.2196/79975 Multimedia Appendix 2 Dataset of 90 question-and-answer pairs.

10.2196/79975 Multimedia Appendix 3 Prompt examples.

10.2196/79975 Multimedia Appendix 4 Dataset of 32 test questions or scenarios.

10.2196/79975 Multimedia Appendix 5 Interface for test question no. 18 with two types of responses.

10.2196/79975 Multimedia Appendix 6 Interview questions.

10.2196/79975 Multimedia Appendix 7 Table reports on the Mann-Whitney *U* test results.

10.2196/79975 Multimedia Appendix 8 Table reports on the comparisons in terms of mean and SD values for each evaluation criterion under two conditions.

10.2196/79975 Multimedia Appendix 9 Study materials appendix: 32 testing scenarios and the corresponding 32 pairs of large language model–based responses.

## Abbreviations

AI: artificial intelligence
API: application programming interface
C1: baseline condition
C2: experimental condition
COPE: Committee on Publication Ethics
EQ: evaluation criterion
HCI: human-computer interaction
LLM: large language model
RQ: research question
