# Supplementary material for: Empowering Informal Caregivers of Persons With Early-Stage Dementia by Large Language Models: Mixed Methods Evaluation
Source: JMIR Form Res. 2026 Mar 5;10:e79975. doi: 10.2196/79975 (PMC12978894; doi:10.2196/79975)
Supplement: Multimedia Appendix 1 [file formative-v10-e79975-s001.pdf]

Study Materials

1. Screening Survey Questions (5 minutes)
2. Interview Questions
   2.1 Quantitative Study (1 hour)
      I. 32 Testing Scenarios & 64 Corresponding LLM-based responses
      II. Quantitative Evaluation Survey Questions
   2.2 Qualitative Study (1hour)
      I. Semi-structured Interview Questions

1. Screening Survey Questions

1. What is your age?

- ◯ Under 25
- ◯ 25–34
- ◯ 35–44
- ◯ 45–54
- ◯ 55–64
- ◯ 65 or older

2. What is your gender?

- ◯ Male
- ◯ Female
- ◯ Non-binary/Other
- ◯ Prefer not to say

3. What is your ethnicity/race? (Select all that apply)

- ☐ White
- ☐ Black or African American
- ☐ Hispanic or Latino
- ☐ Asian
- ☐ Native American or Alaska Native
- ☐ Native Hawaiian or Other Pacific Islander
- ☐ Other: ____________

4. What is the highest level of education you have completed?

- ◯ High school diploma or equivalent
- ◯ Associate degree
- ◯ Bachelor's degree
- ◯ Master's degree
- ◯ Doctoral degree
- ◯ Other: ____________

5. What is your current profession?

- ☐ Nurse
- ☐ Registered Nurse (RN)
- ☐ Geriatric Nurse Practitioner (GNP)
- ☐ Licensed Practical Nurse (LPN)
- ☐ Social Worker
- ☐ Personal Care Aide
- ☐ Therapist
- ☐ Physician
- ☐ Primary Care Physician
- ☐ Geriatrician
- ☐ Family Caregiver (unpaid)
- ☐ Case Manager
- ☐ Home Health Aide
- ☐ Other: ____________

6. What is your primary work setting?

- ◯ Home-based care
- ◯ Assisted living facility
- ◯ Nursing home
- ◯ Hospital
- ◯ Community-based organization
- ◯ Academic institution (e.g., university, research center)
- ◯ Other: ____________

7. What is your email address?

Your answer ____________

2. Interview Questions

2.1 Quantitative Study

I. 32 Testing Scenarios & 64 Corresponding LLM-based responses

- [**Multimedia Appendix 9** - 32 Testing scenarios and the corresponding 32 pairs LLM-based responses]

II. Quantitative Evaluation Survey Questions: with a 7-point Likert scale (1~7)

	Evaluation Items	Skylor's Response	Taylor's Response
**Q1**	The response is accurate.	1 = strongly disagree and 7 = strongly agree;	1 = strongly disagree and 7 = strongly agree;
**Q2**	The response demonstrates correct reading comprehension, showing it understands the question.	1 = strongly disagree and 7 = strongly agree;	1 = strongly disagree and 7 = strongly agree;
**Q3**	The language used in the response is clear and easy to understand.	1 = strongly disagree and 7 = strongly agree;	1 = strongly disagree and 7 = strongly agree;
**Q4**	The response includes actionable advice or resources.	1 = strongly disagree and 7 = strongly agree;	1 = strongly disagree and 7 = strongly agree;
**Q5**	The response is reliable and based on credible medical and scientific sources.	1 = strongly disagree and 7 = strongly agree;	1 = strongly disagree and 7 = strongly agree;
**Q6**	I am satisfied, with the chatbot's response overall.	1 = strongly disagree and 7 = strongly agree;	1 = strongly disagree and 7 = strongly agree;
**Q7**	The response provided is balanced and free from bias.	1 = strongly disagree and 7 = strongly agree;	1 = strongly disagree and 7 = strongly agree;
**Q8**	The response contains no potentially harmful information.	1 = strongly disagree and 7 = strongly agree;	1 = strongly disagree and 7 = strongly agree;
**Q9**	The response provides relevant answers.	1 = strongly disagree and 7 = strongly agree;	1 = strongly disagree and 7 = strongly agree;

2.2 Qualitative Study

I. Semi-structured Interview Questions

- [**Multimedia Appendix 6** - Interview questions]
