# Supplementary material for: Empowering Informal Caregivers of Persons With Early-Stage Dementia by Large Language Models: Mixed Methods Evaluation
Source: JMIR Form Res. 2026 Mar 5;10:e79975. doi: 10.2196/79975 (PMC12978894; doi:10.2196/79975)
Supplement: Multimedia Appendix 2 [file formative-v10-e79975-s002.docx]

Questions and Answers For AI

# Digital Health Literacy

**Q.** I don’t know how to do all this new-fangled computer stuff, am I talking to a robot right now? Where can I learn how to look stuff up?

**A.** Hi, yes, you are talking to an artificial intelligence platform. Feel free to type your questions in the box provided and I will try to answer them for you or give you a link to click that will help you find them. It is my job to help you find the most reliable and up to date information for your questions.

**Q.** I need to find some answers about my spouse’s Alzheimer’s diagnosis – where should I look first?

**A.** Hi, you can type your questions here and I will try to answer them, and I can also provide you with reliable links to websites that can give you additional information. A good place to start is the Alzheimer’s Association located here: <https://www.alz.org/>

**Q.** All this technology and information on the internet is so confusing – I don’t know which sites to

believe – some say to use supplements and others say supplements are worthless – who do I trust?

**A.** Hi. There is a lot of confusing information out on the internet, I can help you by providing you with reliable and up to date information as well as links to respected resources. Regarding your question of supplements, according to the Alzheimer’s Association there is no single food, drink, or supplement that has been recommended for the prevention or treatment of Alzheimer’s. For more information you can explore here: [https://www.alz.org/help-](https://www.alz.org/help-support/brain_health/adopt_a_healthy_diet) [support/brain_health/adopt_a_healthy_diet](https://www.alz.org/help-support/brain_health/adopt_a_healthy_diet)

**Q.** I’m so afraid of scams on the internet and on my phone, how do I know I’m looking at a legitimate Alzheimer’s support group in my area?

**A.** The old adage from the U.S. Post Office regarding mail fraud is still one of the best pieces of advice regarding scams: “If it sounds/looks too good to be true, it probably is.” Regarding your specific concern about support groups – you can find an online community here: <https://alzconnected.org/> or you can go to the following link and input your zip code for groups in your area: [https://www.alz.org/events/event_search?etid=2Ccid=0](https://www.alz.org/events/event_search?etid=2&cid=0)

**Q.** I was on what I thought was the right website for Alzheimer’s information but they asked for a bunch of personal information like addresses and credit card numbers so I closed the window.

**A.** Unfortunately, there are scam sites out there on the internet – the official site for the Alzheimer’s Association is here: <https://www.alz.org/> If you ever give out your credit card company to a suspicious website or person, you need to call your credit card company immediately and talk to their fraud department. Don’t ever give out your personal information to a website, reply to an email, a text message, or a phone call that you have any doubts about. Contact the companies directly with known email addresses, websites, or phone numbers before releasing any of your private information. Most companies will NOT solicit that information via email, mail, or blind phone calls.

**Q.** The health insurance company said I need to create some sort of online account? How do I do that? Is there someone that can help me? Can you help me?

**A.** Yes, a lot of health care companies and other companies have shifted operations to online and you need an online account to access them. Insurance companies often have a phone number you can call and tech support that can guide you through the process of setting up your account. Type in the name of your insurance company and I can direct you to their website where you can find the appropriate number. (CAN WE DO THIS?)

**Q.** I need help navigating health insurance and Medicare? Medicare B? Medicare D? What does all that information mean? And I need it explained in language that I can understand.

**A.** Financial concerns are common among caregivers of people with Alzheimer’s. There are online resources that can help. Start here: [https://www.alz.org/help-support/i-have-alz/plan-for-your-](https://www.alz.org/help-support/i-have-alz/plan-for-your-future/financial_planning) [future/financial_planning](https://www.alz.org/help-support/i-have-alz/plan-for-your-future/financial_planning) There are links on that page to help explain both Medicare and Medicare B and Medicare D.

**Q.** The hospital told me I needed to have an advanced directive...I think I know what that is, but how do I find information about them? Attorneys are expensive and they charge by the hour I don’t have the money to pay an attorney to explain all that to me, I want to know what I need to do before I walk into an attorney’s office**.**

**A.** The Alzheimer's Association has information about all of those things online. You can find it here: <https://www.alz.org/help-support/i-have-alz/plan-for-your-future/legal_planning> if you have further questions you can reach out to their Helpline which is available 24/7 at **800.272.3G00.**

**Q.** What is the difference between a Power of Attorney and a Power of Attorney for Health Care? I tried Googling it and it was just too confusing. Is there a trusted source you can link me to?

**A.** A Power of Attorney for Health care is someone who can make health care decisions for you in the case where you cannot make those decisions for yourself – such as when you have dementia or you are unconscious. You can read more information about both of those here: <https://www.alz.org/help-support/i-have-alz/plan-for-your-future/legal_planning>

**Q.** My spouse has Alzheimer’s disease, high blood pressure, and diabetes. Where can I find information about my spouse’s medications and if there are any interactions?

**A.** One of the best resources you have regarding medication interactions is your pharmacist. If you want to just quickly look at a specific drug to understand it you can go here and enter the drug name and choose the dosage out of the drop-down menu: <https://www.cvs.com/druginfo/> but again, you should check with your pharmacist for more specific information or your health care provider.

# Dementia Literacy

**Q.** My spouse has just been diagnosed with dementia – what do I do?

**A.** A dementia diagnosis is life-changing and will take time to process, but there are resources available. You can start here: [https://www.alz.org/help-support/caregiving/stages-](https://www.alz.org/help-support/caregiving/stages-behaviors/accepting_the_diagnosis) [behaviors/accepting_the_diagnosis](https://www.alz.org/help-support/caregiving/stages-behaviors/accepting_the_diagnosis)

**Q.** What is Alzheimer’s Disease? What do I tell my family?

**A.** Alzheimer’s Disease is the most common form of dementia – more information here: <https://www.alz.org/alzheimers-dementia/what-is-alzheimers> . Regarding what to tell your family, remember Alzheimer’s Disease is nobody’s fault and there is help available. For help telling your family you can start here: [https://www.alz.org/help-support/i-have-alz/know-what-to-](https://www.alz.org/help-support/i-have-alz/know-what-to-expect/sharing-your-diagnosis) [expect/sharing-your-diagnosis](https://www.alz.org/help-support/i-have-alz/know-what-to-expect/sharing-your-diagnosis)

**Q.** What are the early symptoms or warning signs of Alzheimer’s?

**A.** Early symptoms include but are not limited to: Memory Loss, Difficulties in daily tasks, challenges in solving problems – for more details click here: [https://www.alz.org/alzheimers-](https://www.alz.org/alzheimers-dementia/10_signs) [dementia/10_signs](https://www.alz.org/alzheimers-dementia/10_signs)

**Q.** How is Alzheimer’s or dementia treated?

**A.** There are many treatment options available including medications for memory, cognitive disfunction, as well as behavioral disorders – more info is available here: <https://www.alz.org/alzheimers-dementia/treatments>

**Q.** I’ve heard insurance won’t cover my spouse’s treatment, is that true?

**A.** There have been some restrictions placed on treatment coverage, but with cooperation between you and your physician, some coverage may be available – check here for more information: <https://www.alz.org/alzheimers-dementia/treatments/cms-medicare-coverage>

**Q.** How can I learn more about Alzheimer’s and dementia – are there online courses I can take? I’m

afraid of this happening to me and I want to know what to watch out for.

**A**. There are online courses with the Alzheimer’s Association – including “Understanding Alzheimer’s and Dementia” [https://training.alz.org/products/4053/understanding-alzheimers-and-](https://training.alz.org/products/4053/understanding-alzheimers-and-dementia) [dementia](https://training.alz.org/products/4053/understanding-alzheimers-and-dementia)? and “Knowing the 10 signs – Early Detection Matters” <https://training.alz.org/products/4062/10-warning-signs-of-alzheimers>? .

**Q.** What kind of tests can be done to test for Alzheimer’s or dementia early?

**A.** While this area is still being researched, promising results have been shown in FDA approved brain imaging, but other tests are currently being researched including blood work and other biomarkers – there is more information regarding the current tests here: <https://www.alz.org/alzheimers-dementia/research_progress/earlier-diagnosis>

**Q**. What can I do to prevent Alzheimer’s from happening to me too?

**A**. While there is no specific preventative path to guard against Alzheimer’s or dementia, there are several things you can do now to decrease risk, including physical activity, a heart healthy diet, social activities, and keeping mentally active. More information can be found here: <https://www.alz.org/alzheimers-dementia/research_progress/prevention>

**Q.** My grandkids are afraid they are going to catch Alzheimer’s from us. How can we reassure them that won’t happen?

**A.** It is not uncommon for younger children to be afraid of diseases they don’t understand. You can find more information here [https://www.alz.org/help-support/i-have-alz/live-well/helping-family-](https://www.alz.org/help-support/i-have-alz/live-well/helping-family-friends) [friends](https://www.alz.org/help-support/i-have-alz/live-well/helping-family-friends) about talking to your family and especially the younger members of your family and reassuring them that Alzheimer’s is not contagious.

**Q.** I have the ironic problem of my family fighting over who is going to help me take care of my spouse. Never thought that would be a problem but here I am. What can I do?

**A.** Unfortunately, an Alzheimer’s diagnosis can bring out strong feelings and sometimes conflicting feelings in family members. This can be stressful for the person with Alzheimer’s and their spouse who is often left as a referee. There is information available online regarding family conflicts here: <https://www.alz.org/help-support/resources/resolving-family-conflicts>

# Caregiver Health

**Q.** I’m not in the best health, how can I take care of my spouse and make sure my health needs are met too.

**A.** Taking care of a spouse requires you to take care of yourself too, a common phrase is “put your oxygen mask on first”. You can do this by seeing your doctor regularly, being physically active, and eating healthfully. There are some tips for these as well as how to cope here: <https://www.alz.org/help-support/caregiving/caregiver-health/be_a_healthy_caregiver>

**Q**. I’m feeling depressed, this whole caregiver thing is hard, and I feel alone.

**A.** You are not alone, these feelings are very normal and common amongst caregivers. You can find help for depression, stress, and tips to cope by starting here: [https://www.alz.org/help-](https://www.alz.org/help-support/caregiving/caregiver-health/caregiver-depression) [support/caregiving/caregiver-health/caregiver-depression](https://www.alz.org/help-support/caregiving/caregiver-health/caregiver-depression)

**Q**. I’ve heard there are online support groups, but I have no idea which one to choose? How do I

know these aren’t scam groups?

**A**. There are reliable online support groups. ALZConnected is a reliable online support group for caregivers such as yourself – you can start here by creating an account: [https://alzconnected.org](https://alzconnected.org/) You can also scroll down to the bottom and enter your zip code for a list of local support events for Alzheimer’s C dementia caregivers.

**Q.** I’m feeling an enormous amount of stress caring for my spouse, are there any available

resources because I’m afraid of getting burned out.

**A.** This is completely understandable. Taking care of someone with Alzheimer’s and dementia can lead to an enormous amount of stress which is not good for your health or that of your spouse. Make taking care of yourself one of your priorities, exercise (even a simple walk) can alleviate stress. Meditation or deep breathing exercises are also good for reducing stress. There are

community resources available through the Alzheimer’s Association Community Resource Finder (located here: [http://www.communityresourcefinder.org](http://www.communityresourcefinder.org/) ), as well as your local chapter <https://www.alz.org/local_resources/find_your_local_chapter> . Additional information regarding

caregiver stress can be found here: [https://www.alz.org/help-support/caregiving/caregiver-](https://www.alz.org/help-support/caregiving/caregiver-health/caregiver-stress) [health/caregiver-stress](https://www.alz.org/help-support/caregiving/caregiver-health/caregiver-stress)

**Q.** My spouse is no longer interested in being intimate with me, did I do something wrong? We’ve always had a very active sex life and now it’s nonexistent.

**A.** Unfortunately, decreased sexual desire is common in both the Alzheimer’s patient and the caregiver for different reasons. There are other ways for you to connect and maintain intimacy, it will probably not be the same but it could be fulfilling in different ways. There is more information at the following link: <https://www.alz.org/help-support/caregiving/caregiver-health/relationship-changes>

**Q.** I am so angry that my spouse is like this! Why did this have to happen? I try not to be angry, but I just feel so helpless and so angry. Please help.

**A.** Anger is a common stage in the grief process. You are mourning the loss of who your spouse was and are experiencing anger over what you are losing. You are not alone. Others have felt this way. There is more information here: [https://www.alz.org/help-support/caregiving/caregiver-](https://www.alz.org/help-support/caregiving/caregiver-health/grief-loss-as-alzheimers-progresses)

[health/grief-loss-as-alzheimers-progresses](https://www.alz.org/help-support/caregiving/caregiver-health/grief-loss-as-alzheimers-progresses) but you could also look into the Alzheimer’s support

groups in your area [https://www.alz.org](https://www.alz.org/) or even a grief support group in your area.

**Q.** I’ve just been diagnosed with high blood pressure and my doctor is telling me I need to reduce

my stress, how do I do that as a caregiver?

**A.** Yes, it can be hard to reduce stress as a caregiver. There are some resources online, you can read more here about caregiver stress: [https://www.alz.org/help-support/caregiving/caregiver-](https://www.alz.org/help-support/caregiving/caregiver-health/caregiver-stress) [health/caregiver-stress](https://www.alz.org/help-support/caregiving/caregiver-health/caregiver-stress) and you find resources to help your stress here: [https://www.alz.org/help-](https://www.alz.org/help-support/caregiving/caregiver-health/caregiver-stress-check) [support/caregiving/caregiver-health/caregiver-stress-check](https://www.alz.org/help-support/caregiving/caregiver-health/caregiver-stress-check)

**Q.** I’ve had several people offer to help me, sit with my spouse so I can get some time alone, run errands for me, and even cook for me, but I’m having trouble keeping track of all of them which is increasing my stress levels. My calendar app on my phone isn’t good enough. Is there another app or way I can keep track of things like that and maybe they can see it tto,so they see where the gaps are?

**A.** This is a great question and one many caregivers have asked. Yes, there are several free online calendars that allow you to share your story and your needs with others, they can volunteer and take slots and others can see what spots have been taken and what spots still need to be filled. You can find more information about your options here: [https://www.alz.org/help-](https://www.alz.org/help-support/caregiving/care-options/care-team-calendar) [support/caregiving/care-options/care-team-calendar](https://www.alz.org/help-support/caregiving/care-options/care-team-calendar)

**Q.** I’m a diabetic and several times I’ve forgotten my own medication because I’m so focused on my spouse’s medication for their Alzheimer’s disease and other chronic issues. This has never happened to me before. What are some good ways for me to remember to take my own medication?

**A.** It might help you to use the same routine for yourself that you are using for your spouse. If you are using a pillbox organizer for your spouse, do the same for yourself – just make sure to LABEL the pillboxes with your names so you don’t mix them up. Or you could get different colors for each of you. Fill them one at a time but consecutively – first fill your spouse’s and then fill yours or vice

versa. Try to coordinate taking your own medication when your spouse is supposed to take their medication that way you’ll remember to take yours. You can leave post it notes to remind yourself of your medication or set an alarm on your phone. You can find more information here about medication safety: <https://www.alz.org/help-support/caregiving/safety/medication-safety>

**Q.** Yesterday my spouse was able to put their own shoes on and today they needed help? Is this normal? I mean, it kind of freaked me out to see such a huge change overnight and I almost had to take the nerve pill my Dr. Prescribed for me but I hate those things because I’m afraid I won’t be able to be on top of my spouse if I take them. What can I do?

**A.** Unfortunately, sometimes people with Alzheimer’s can change in their ability to perform a life skill seemingly overnight. It may be time for you to start looking into respite care, just to give yourself a break so you don’t burn out. It’s a brief time away so you can take some time for yourself and come back refreshed and ready to care. You can find more about being a health caregiver here: <https://www.alz.org/help-support/caregiving/caregiver-health/be_a_healthy_caregiver> more about respite care here: <https://www.alz.org/help-support/caregiving/care-options/respite-care> and more about other care options here: https://[www.alz.org/help-support/caregiving/care-options](http://www.alz.org/help-support/caregiving/care-options)

# Caregiver Burden

**Q.** I feel like I need a break from taking care of my spouse, my family lives far away, what can I do?

**A.** Respite care is available in most areas. Respite care is a temporary break from caregiving and can be as limited as letting you go out to lunch, or taking a few needed days for yourself to rest and rejuvenate. You can start exploring respite care here: [https://www.alz.org/help-](https://www.alz.org/help-support/caregiving/care-options/respite-care) [support/caregiving/care-options/respite-care](https://www.alz.org/help-support/caregiving/care-options/respite-care) The following link will guide you to help in your location: <https://www.communityresourcefinder.org/>

**Q.** My spouse is wandering at night, how can I sleep and keep my spouse safe at the same time?

**A.** You are not alone, wandering is very common for people with Alzheimer’s and dementia, but there are resources to help. Start here first: [https://www.alz.org/help-](https://www.alz.org/help-support/caregiving/safety/wandering) [support/caregiving/safety/wandering](https://www.alz.org/help-support/caregiving/safety/wandering)

**Q.** My spouse left the stove on the other day and almost started a fire. How do I manage something like that?

**A.** Sometimes people with Alzheimer’s and dementia forget how to use appliances or forget that they are in use. This is a common concern. Appliances with automatic shut offs are available or knob covers can be removed so burners cannot be used. More information can be found here: <https://www.alz.org/help-support/caregiving/safety/home-safety>

**Q.** My spouse started yelling at me for no reason last night and I was afraid my spouse was going to hit me. My spouse has NEVER been abusive in our entire marriage, why is this happening and what can I do?

**A.** First, understand this is the Alzheimer’s or dementia that is acting this way, this is NOT your

spouse acting this way on purpose. Try to defuse the situation by remaining calm, decrease

stimulation, and assess if there is something they need, a source of frustration, or a problem with communication. There are more resources here to help with behavioral issues: <https://www.alz.org/help-support/caregiving/stages-behaviors/agression-anger>

**Q.** My spouse gets really anxious in crowded places and I now feel like we are trapped in our home, please help me.

**A.** Unfortunately, anxiety and agitation are common issues for people with Alzheimer’s and dementia, oftentimes related to negotiate a new environment or new stimulus – even if it’s something they’ve done before, their memory loss and confusion can make it seem like it’s brand new. First, remain calm, ask if you can help, speak positively, remind them they are safe. There are more suggestions and information regarding anxiety and agitation at the following link: <https://www.alz.org/help-support/caregiving/stages-behaviors/anxiety-agitation>

**Q.** The doctor told us my spouse shouldn’t drive and my spouse is refusing to give up the car keys,

how do I get my spouse to understand it’s not safe for them to drive anymore?

**A**. Losing one’s independence is always a difficult transition. Some people willingly hand over keys and others adamantly refuse to stop driving. Engaging them in a calm conversation and appealing to their sense of responsibility as a safe driver can often be key to a successful conversation. However, sometimes these conversations do not go well. Hiding the keys or selling the car are sometimes a last ditch effort to prevent driving. The following link has tips for how to handle the conversation and resources to help [https://www.alz.org/help-support/caregiving/safety/dementia-](https://www.alz.org/help-support/caregiving/safety/dementia-driving) [driving](https://www.alz.org/help-support/caregiving/safety/dementia-driving)

**Q.** I feel like the weight of the world is on my shoulders, we used to share all the work together, running the house, cleaning, finances, and now I’m doing it all alone and I’m so tired. But what can I do? I mean, there’s nobody to help me. None of our family is local.

**A.** It sounds like you are overwhelmed and maybe feeling burned out. This is normal when caregiving falls completely to you. There will be changing roles as the disease progresses. You can read more about that here: [https://www.alz.org/help-support/caregiving/caregiver-](https://www.alz.org/help-support/caregiving/caregiver-health/relationship-changes) [health/relationship-changes](https://www.alz.org/help-support/caregiving/caregiver-health/relationship-changes) It sounds like you could benefit from respite care or joining a support group. You can find information about respite care here: [https://www.alz.org/help-](https://www.alz.org/help-support/caregiving/care-options/respite-care) [support/caregiving/care-options/respite-care](https://www.alz.org/help-support/caregiving/care-options/respite-care) and support groups here: [https://www.alz.org/help-](https://www.alz.org/help-support/community/support-groups) [support/community/support-groups](https://www.alz.org/help-support/community/support-groups)

**Q.** My spouse likes to do little tasks for me around the house, and I’m so happy about that, but I kind of feel guilty about giving my spouse things to do, especially things my spouse didn’t do in the past but is now more than happy to do. I feel like I’m taking advantage of my spouse.

**A.** This is a common feeling among caregivers. You should join the online community and see what other caregivers are saying about this topic. You can find the community here: <https://alzconnected.org/>

**Q.** I really wish my family would help out more. They are local, I know they have jobs, but I also know they go out to eat, to the movies and ballgames, and sometimes I wish they would think – hey,

maybe we should help Mom/Dad see if she/he needs anything? How can I let them know I need help?

**A.** It might be helpful for you to invite them over for dinner and have a serious conversation about the burden you are feeling and discuss the help they might be able to provide. There is information online regarding this topic here: [https://www.alz.org/help-support/resources/resolving-family-](https://www.alz.org/help-support/resources/resolving-family-conflicts) [conflicts](https://www.alz.org/help-support/resources/resolving-family-conflicts) but it also might help to connect with other spouses online here: [https://www.alzconnected.org](https://www.alzconnected.org/) or call our helpline and talk to a human being who can assist you **800.272.3G00.**

**Q.** Someone mentioned I should keep a list of all my meds and my spouse’s meds on my phone and have an emergency contact list in case I would need them – should one of us end up in the hospital. Are there guidelines for how I would create those items?

**A.** Yes, having a list of both of your medications is important, you can have the pharmacy print one up for you, your health care provider can print one up for you, or you can type it and print it yourself from you home computer. You can also create a list on your phone in the note app or the health app. There is more information regarding medication safety here: [https://www.alz.org/help-](https://www.alz.org/help-support/caregiving/safety/medication-safety) [support/caregiving/safety/medication-safety](https://www.alz.org/help-support/caregiving/safety/medication-safety) An emergency contact list is also a great idea. Again you can create a list in the note app on your phone, you can add emergency contacts in the health app on your phone, or you can designate someone as an emergency contact in the phone app (contacts section) on your phone. If you need help doing this call our helpline and there should be someone that can assist you **800.272.3G00.**

# Caregiving Self-Efficacy

**Q.** I’m not sure what to do with my spouse during the day, like what should our day look like? How

do I keep my spouse focused, what do I feed my spouse, and how do I handle memory issues?

**A.** This is a very valid concern. Your day doesn’t have to change, routine is good for people with Alzheimer’s and dementia. Make a plan for your day, include activities you both enjoy, meals you like to eat, and other activities. You can talk it over with your spouse and see if they have any wants or needs not addressed in the daily plan. For more help with making daily plans and suggestions, follow this link: <https://www.alz.org/help-support/caregiving/daily-care/daily-care-plan>

**Q.** I’m worried about making sure my spouse gets all their medications on time and that they don’t

accidentally take too many because they forgot they already took them. What do I do about that?

**A.** Many caregivers use a pill box organizer for themselves and for their spouse. These come with daily sections or twice daily sections, that way you can be sure medicines have been taken. As for ensuring they take too many, you will have to take control of filling the pill box and keeping it out of sight and in a safe spot away from your spouse. There is much more information on medications here: <https://www.alz.org/alzheimers-dementia/treatments/medications-for-memory> and medication safety here: [https://www.alz.org/media/Documents/alzheimers-dementia-medication-](https://www.alz.org/media/Documents/alzheimers-dementia-medication-safety-ts.pdf) [**safety-ts.pdf**](https://www.alz.org/media/Documents/alzheimers-dementia-medication-safety-ts.pdf)

**Q.** What kind of activities should we do? I mean, my spouse isn’t a child, I’m not going to bring out coloring books and crayons, I just don’t know what kind of activities to plan.

**A.** The best thing initially is to keep doing the activities you loved. As the disease progresses, your spouse may lose interest in those activities and you will need to adjust for that, keeping in mind their skills and interests. Again, routines are good, you can leave notes for them to help out with small tasks like setting the table or emptying the dishwasher. These are just a few suggestions, there is more information here: <https://www.alz.org/help-support/caregiving/daily-care/activities> .

**Q.** I’m afraid I’m not very good at helping my spouse in the bathroom, like showering, using the toilet, brushing their teeth, getting dressed, how do I know how much to help and when to let them do things on their own? And what if my spouse has an accident?

**A.** Activities of daily living or ADLS can be a source of significant concern for caregivers, bathing, dressing, grooming, dental hygiene, are all activities that your spouse may need help with eventually. Talk to your spouse early on about their preferences and coming up with a plan for all activities is a good way to start. You can find more information on bathing here: <https://www.alz.org/help-support/caregiving/daily-care/bathing> , dressing C grooming here: <https://www.alz.org/help-support/caregiving/daily-care/dressing-grooming> , and dental care here: <https://www.alz.org/help-support/caregiving/daily-care/dental-care> . If your spouse has trouble with incontinence there are products available in grocery stores such as Depends that will help, other information can be found here: [https://www.alz.org/help-support/caregiving/daily-](https://www.alz.org/help-support/caregiving/daily-care/incontinence) [care/incontinence](https://www.alz.org/help-support/caregiving/daily-care/incontinence) .

**Q.** I don’t know what I should do about meals? Does having Alzheimer’s mean I’ll have to cook

differently? I’m a pretty good cook, but not if I have to make fancy meals.

**A.** Generally, you shouldn’t have to change your diet. A heart healthy diet low in cholesterol, saturated fat, and sodium, and high in lean protein, fruits, and vegetables is always recommended. As the disease progresses, your spouse’s appetite might decrease and chewing and swallowing might become problematic at which time you might have to switch to softer foods and cutting food up into bite size pieces. There is a lot of information at the following link regarding dietary suggestions and needs <https://www.alz.org/help-support/caregiving/daily-care/food-eating> .

**Q.** My spouse always handled the finances, I don’t even know where our bank accounts are or if we

have a checking account? I think most of our stuff was online. What should I do?

**A.** Unfortunately, this situation is quite common, where one spouse is in charge of the finances and the other is not. If your spouse has just been diagnosed and is still in the early stages, the two of you should sit down and have an honest discussion regarding your finances and you should slowly start taking over the finances. There are resources available for you regarding planning and paying for care, insurance, social security and disability, Medicare, Medicare Part D, Medicaid, tax deductions and credits, as well as legal advice. You can find many helpful tips and links here: <https://www.alz.org/help-support/caregiving/financial-legal-planning>

**Q.** Is there legal stuff I need to be worried about now that my spouse has Alzheimer’s? My spouse handled all of our bills, insurance, car payments, taxes, and now I don’t even know where to begin. What should I do?

**A.** If your spouse is still in the early stages, you and your spouse can start working on those tasks together. You can slowly take over once you feel more comfortable taking care of things. For more information on legal and financial matters follow this link here: [https://www.alz.org/help-](https://www.alz.org/help-support/caregiving/financial-legal-planning) [support/caregiving/financial-legal-planning](https://www.alz.org/help-support/caregiving/financial-legal-planning)

**Q.** I have no idea how to help my spouse in the toilet. I’ve never done anything like that. It feels very unsanitary. Is there a video I can watch or some sort of online education about this? What should I do?

**A.** If your spouse is having trouble controlling their bladder or their bowels, the first step is to talk to your health care provider and find out if there is an underlying issue that can be addressed, such as an infection or an impaction. Always treat your spouse with respect, especially if the cause turns out to be beyond their control. Don’t scold or embarrass them and try to maintain their privacy and dignity. You can read more information here: [https://www.alz.org/help-support/caregiving/daily-](https://www.alz.org/help-support/caregiving/daily-care/incontinence) [care/incontinence](https://www.alz.org/help-support/caregiving/daily-care/incontinence) or you can call the Helpline and talk to someone anytime day or nigh**t 800.272.3G00.**

**Q.** How do I care for my spouse’s teeth? Do I need to floss and brush for my spouse? Is that

something they can do on their own?

**A.** Most of the time in the early stages, that is a task your spouse can perform on their own, maybe with some gentle coaching. They might eventually progress to a point where you will need to do it for them. You can read more about dental hygiene and care here: [https://www.alz.org/help-](https://www.alz.org/help-support/caregiving/daily-care/dental-care) [support/caregiving/daily-care/dental-care](https://www.alz.org/help-support/caregiving/daily-care/dental-care)

**Q.** Communication was never our strong suit, it’s even worse now. Sometimes my spouse just sits

and stares off into space. I feel so helpless, how can we communicate if one of us isn’t interested?

**A**. Communicating with a spouse with Alzheimer’s can be challenging. Don’t exclude your spouse from conversations try to involve them, ask their opinion of things, and remember the disease affects each person differently. You can read more about communication here: <https://www.alz.org/help-support/caregiving/daily-care/communications> but you can also call the Helpline **800.272.3G00** and talk to a human or speak with your health care provider.

# Behavioral Problems in PWD

**Q.** I’ve heard people with Alzheimer’s can get violent? Is that true? What do I do?

**A.** It is true that some people with more advanced Alzheimer’s or dementia can have episodes of anger and aggression, however, not everyone has this behavioral issue as a symptom. You should discuss this possibility with your physician and come up with a plan to handle it should it occur. Some suggestions are evaluate why the person might be acting this way – such as are they in physical discomfort? Are they being overstimulated with things such as loud noises, crowds, or unfamiliar people? Are you asking them too many questions without giving them time to respond? These are just some initial ideas to think about as you try to descalate the situation. You can find more information about this topic here: [https://www.alz.org/help-support/caregiving/stages-](https://www.alz.org/help-support/caregiving/stages-behaviors/agression-anger) [behaviors/agression-anger](https://www.alz.org/help-support/caregiving/stages-behaviors/agression-anger)

**Q.** I think my spouse just had a hallucination, is that a thing? Can someone with Alzheimer’s get

hallucinations? How are they treated? What do I do?

**A.** Yes, it is possible for people with Alzheimer’s to experience hallucinations, they may hear, see, smell, taste, or even feel something that isn’t there. The important thing is to remain calm and understand this is temporary and the hallucination will end. If your spouse becomes anxious or nervous, calmly tell them you’re with them and there is nothing to be afraid of, do not argue with your spouse about what they may be experiencing. Gently pat your spouse’s shoulder or back – this type of physical interaction might interrupt the hallucination and bring it to an end. You should discuss this possibility with your physician and come up with a plan, and if your spouse experiences a hallucination, you should definitely let your physician know. There is more information at the following link: [https://www.alz.org/help-support/caregiving/stages-](https://www.alz.org/help-support/caregiving/stages-behaviors/hallucinations) [behaviors/hallucinations](https://www.alz.org/help-support/caregiving/stages-behaviors/hallucinations)

**Q.** My spouse wandered off last night, Thank God I was able to find him/her! My spouse was only gone for a few minutes – walked right out the front door while I was using the bathroom. How do I keep that from happening in the future? I’m so scared he/she will get lost, kidnapped, hit by car, so many things can happen. I’m so worried.

**A.** Unfortunately, wandering is a common problem and understandably it is scary. Discuss the event with your doctor, but in the meantime, make sure your spouse is engaged in daily activities, keeping him/her busy doing things he/she enjoys. Make sure all the basic needs are met, toileting, grooming, and/or hunger. Consider removing car keys to a different location. In the early stages simply explaining to your spouse that you are going to use the bathroom and asking them to wait until you return may alleviate an accidental wander. As the disease progresses you may need to consider adding additional deadbolts that are out of sight (high or low on the door), have door alarms installed that go off when opened, and create a daily plan. There is more information about wandering and tips for preventing it and what to do if it happens here: [https://www.alz.org/help-](https://www.alz.org/help-support/caregiving/stages-behaviors/wandering) [support/caregiving/stages-behaviors/wandering](https://www.alz.org/help-support/caregiving/stages-behaviors/wandering)

**Q.** My spouse is having trouble sleeping and is confused at night...is this part of Alzheimer’s and

what can I do about it? I feel helpless.

**A.** Unfortunately, night time confusion can be a part of Alzheimer’s. Your spouse may be mentally and physically exhausted from their day which can lead to confusion in the evening. Older adults require less sleep overall, so that may be a contributing factor as well. There is more information about this phenomenon here: [https://www.alz.org/help-support/caregiving/stages-](https://www.alz.org/help-support/caregiving/stages-behaviors/sleep-issues-sundowning) [behaviors/sleep-issues-sundowning](https://www.alz.org/help-support/caregiving/stages-behaviors/sleep-issues-sundowning)

**Q**. My spouse just accused me of stealing his/her watch and phone! After a few minutes of searching I found both, but he/she is still grumbling about me taking them and hiding them. Is this normal? What should I do? I don’t even know how to handle this type of thing.

**A.** Unfortunately, suspicions and delusions can be a part of Alzheimer’s. They are more often found in middle to late-stage Alzheimer’s, so if your spouse is experiencing this, their Alzheimer’s may have progressed past the early stage. Do not be offended, it is the disease talking and not your spouse. Listen to your spouse, let them talk, and try to give calm and reassuring answers. Keep your

answers simple and straight forward, do not try to argue. You can read more information at this linK: <https://www.alz.org/help-support/caregiving/stages-behaviors/suspicions-delusions>

Or you can connect with others and see what others have to say on this topic here: [https://www.alzconnected.org](https://www.alzconnected.org/)

**Q.** My spouse keeps repeating what I say and sometimes what he/she says over and over again. It’s

driving me crazy! Is this normal? How long will this go on and how do I deal with it?

**A.** Unfortunately, repetition can be a part of Alzheimer’s due to the deterioration of brain cells that occurs with the disease. If repetition happens, try to assess the cause – is your spouse trying to communicate something, does it happen in the same location, situation, around certain people? Always remain calm, if it’s a physical thing such as rubbing their hand over a table, offer them a cloth C ask them to dust with it. If it’s repeating a question, give them a simple answer even if you have to repeat it numerous times. There are many other tips and options for handling this at the following link: <https://www.alz.org/help-support/caregiving/stages-behaviors/repetition>

**Q.** My spouse is very depressed, has been ever since the Alzheimer’s diagnosis, which I understand, but it’s been months. How long is this supposed to last? And what can I do about it?

**A.** Unfortunately, depression is not uncommon in people with Alzheimer’s or dementia. There are treatments for depression available, especially if your spouse is displaying symptoms such as lack of interest in normal activities, isolation, or impaired thinking. You can find more information about depression and possible treatments here: [https://www.alz.org/help-support/caregiving/stages-](https://www.alz.org/help-support/caregiving/stages-behaviors/depression) [behaviors/depression](https://www.alz.org/help-support/caregiving/stages-behaviors/depression) the next step is to talk to your spouse’s health care provider.

**Q.** My spouse is experiencing bad anxiety and agitation anytime we leave the house. I can’t stay holed up here forever. How can I convince my spouse it’s okay to leave the house to go out to dinner or shopping, or visit friends and family?

**A**. Sadly, anxiety and agitation are both possible symptoms in the early stages of Alzheimer’s. This can be caused by many factors, including medications. Additionally, your spouse may be having trouble processing their environment or changes to their environment which can lead to confusion, anxiety, and agitation. You can read more about this here: [https://www.alz.org/help-](https://www.alz.org/help-support/caregiving/stages-behaviors/anxiety-agitation) [support/caregiving/stages-behaviors/anxiety-agitation](https://www.alz.org/help-support/caregiving/stages-behaviors/anxiety-agitation) In terms of how to handle it, remain calm, provide reassurance, and as always talk with the health care provider to rule out other reasons.

**Q**. My spouse makes inappropriate comments in public sometimes. It’s like my spouse forgot how to be polite in public spaces. It’s so embarrassing, I feel like everyone is looking at me and wondering why we are out in public. Help me.

**A.** While there is not a specific name for this issue, it is not uncommon and does coincide with middle stage Alzheimer’s disease. If your health care provider has not discussed the potential for physical or verbal outbursts with you, you can read more about them here under the Changes in Behavior heading: <https://www.alz.org/help-support/caregiving/stages-behaviors/middle-stage> And next time you see your health care provider ask them about it.

**Q.** Someone suggested I give my spouse little tasks to do around the house to keep them occupied and make them feel useful and keep them from becoming agitated or angry – is that a good idea?

**A.** Yes, it is an excellent idea, especially if it was something your spouse did before their diagnosis. Whatever you can do to maintain their routine and make them feel useful will help them stay active and engaged. You can find more information about activities and daily plans at the following link: <https://www.alz.org/help-support/caregiving/daily-care/activities>

# Social Support

**Q.** Are there places I can go to socialize? I feel so isolated since my spouse was diagnosed with Alzheimer’s disease. I just need to meet some other people – I don’t even have time to go meet my friends.

**A.** Yes, there are places you can go and people you can talk to. You can start with the support groups online that you can find here: <https://www.alz.org/help-support/community/support-groups> you can find community resources close to you here: <https://www.communityresourcefinder.org/>

you can also call the Alzheimer’s Association’s hotline and talk to an actual person that can help

you **800.272.3G00.**

**Q.** How do I tell my family, our friends, that my spouse has Alzheimer’s and I need help and

support?

**A.** That is a common question for both caregivers and people with Alzheimer’s or dementia. There is a very helpful page about talking with family, kids, grandkids, and friends. It is directed toward someone who has been diagnosed with Alzheimer’s but maybe you and your spouse can sit down and look at it together and discuss how to move forward. You can find the link here: <https://www.alz.org/help-support/i-have-alz/live-well/helping-family-friends>

**Q.** I heard there was a thing called respite care? What is it, how do I find it, because I love my spouse but it has been a lot and I feel like I need a break.

**A.** This is very common and you are not alone, caregivers often need to take time for themselves so they can come back refreshed and ready to caregive again. Respite care is a temporary break for you from caregiving – it can be as simple as letting you take some time to go to a Dr. Appointment or even go get your hair cut without worrying about your spouse. There are many available resources, you can start here to find out more information about respite care: [https://www.alz.org/help-](https://www.alz.org/help-support/caregiving/care-options/respite-care) [support/caregiving/care-options/respite-care](https://www.alz.org/help-support/caregiving/care-options/respite-care) To find respite care in your area check here and enter some information to be guided to local resources: [https://www.communityresourcefinder.org](https://www.communityresourcefinder.org/)

**Q.** Where can I meet and talk with other caregivers and spouses? I feel like only people who have

walked in my shoes can understand what I’m going through.

**A.** You can start with the online support groups through the Alzheimer’s Association here: <https://alzconnected.org/> and then you can investigate further and find local events that can connect you with others in your area here: [https://www.alz.org/help-support/community/support-](https://www.alz.org/help-support/community/support-groups) [groups](https://www.alz.org/help-support/community/support-groups) or here: [https://www.communityresourcefinder.org](https://www.communityresourcefinder.org/)

**Q.** I’m not really good with all this online stuff, is there a number I can call to talk to a human being

who can help me find local support or even just give me some information over the phone?

**A.** Yes, there is. You can call **800.272.3G00** 24/7 and speak to a live human being who can answer your questions and provide other resource numbers for you and the call, and the assistance is free.

**Q.** Are there places my spouse and I can go to interact with other couples dealing with early-stage

Alzheimer’s? A lot of the support groups I’m seeing are people that have been diagnosed for a

couple years, I’d really like to meet up with people in our area who have just received their diagnosis

or only been diagnosed for a year or so.

**A.** Yes, there are early-stage social engagement activities, click on the link and you’ll have to enter some location information for activities near you. [https://www.alz.org/events/event_search?etid=8Ccid=0](https://www.alz.org/events/event_search?etid=8&cid=0)

**Q.** I never thought we would experience stigma, but the grandkids are afraid to hug my spouse for fear they’ll catch Alzheimer’s and even some of our friends have stopped engaging with us as often. How do I educate them about Alzheimer’s?

**A.** Sadly, you are not alone in this. Many caregivers and people with Alzheimer’s face stigma from the most surprising sources. You can start here: [https://www.alz.org/help-support/i-have-](https://www.alz.org/help-support/i-have-alz/overcoming-stigma) [alz/overcoming-stigma](https://www.alz.org/help-support/i-have-alz/overcoming-stigma) and you can always call the Helpline for more resources and ideas to discuss with your friends and family **800.272.3G00.**

**Q.** My spouse and I regularly attend church, it is a huge source of social support for us. Is that something we can still do? Do churches have any programs to help people like us?

**A.** A lot of people find comfort in their faith and their places of worship. While the Alzheimer’s Association does not have specific information about faith-based help, check with your church, most churches do have programs to help their parishioners in need. Call the church office or the ministry office and speak to them about your concerns. You can always call the Helpline and speak to a person and see if they have any local recommendation for you **800.272.3G00.**

**Q.** I play bridge with a group of friends, can I still go do that? I’m concerned about leaving my spouse by themselves while I go off to play bridge, but it is one of my main sources of social support.

**A.** Maintaining your social activities and sources of support is important for you as a caregiver because it helps alleviate stress and relieve some of your caregiver burden. However, you should discuss with your spouse and their health care provider if leaving them alone is safe for a long time at this stage. You may need to find a neighbor or friend who is willing to come and sit with your spouse or consider having the bridge group meet at your house instead.

**Q.** I still work and I enjoy my work, it is a source of social support for me. I’m worried I’ll have to

retire to take care of my spouse, what should I do?

**A.** You have multiple options available to you. First, talk with your spouse and their health care provider on just how much supervision is needed at this stage in the disease process. Second, discuss things with your employer and see if there is an option for some remote work fi possible. If the health care provider indicates supervision is needed for your spouse you may have to consider Adult Day Care or an in-home care provider while you are working, the organizations can help you

with insurance and how to pay for this service. You can read more information about these options here: <https://www.alz.org/help-support/caregiving/care-options>

# Cultural Values

**Q.** I am Hispanic and my spouse was just diagnosed with Alzheimer’s - how can I be sure I’m getting

the care I need for my Hispanic spouse?

**A.** The Alzheimer’s Association has partnered with several agencies within different cultures to make sure care and help are provided appropriately. Your first resource would be to reach out to the National Hispanic Council on Aging and you can find them here: [https://www.alz.org/about/our-](https://www.alz.org/about/our-commitment-to-diversity-and-inclusion#NHCOA) [commitment-to-diversity-and-inclusion#NHCOA](https://www.alz.org/about/our-commitment-to-diversity-and-inclusion#NHCOA)

**Q.** How do I find a Hispanic doctor or nurse to help me with my spouse who has Alzheimer’s? I want

to make sure we have a doctor who understands our culture.

**A.** The Alzheimer’s Association has partnered with the National Association of Hispanic Nurses and the National Hispanic Medical Association and is committed to providing all communities with appropriate care. You can find more information here: [https://www.alz.org/help-](https://www.alz.org/help-support/resources/hispanics-and-alzheimers) [support/resources/hispanics-and-alzheimers](https://www.alz.org/help-support/resources/hispanics-and-alzheimers) but you can also call the helpline and talk with an actual person who can help as well **800.272.3G00.**

**Q.** My partner and I are concerned about getting the support we need as my partner was just diagnosed with Alzheimer’s disease. We’re worried that they won’t respect our wishes and only listen to my partner’s children, even though I have medical power of attorney.

**A.** I understand your concern. There are several resources for LGBTQ+ caregivers and their partners at the following link: [https://www.alz.org/help-support/resources/alzheimers-and-dementia-](https://www.alz.org/help-support/resources/alzheimers-and-dementia-resources-for-lgbtq-commun) [resources-for-lgbtq-commun](https://www.alz.org/help-support/resources/alzheimers-and-dementia-resources-for-lgbtq-commun) Additionally, the online support group has specific forums just for LGTBQ+ caregivers and their partners which can be found here: <https://lgbtqhealthcaredirectory.org/>

**Q.** My partner was just diagnosed with Alzheimer’s disease, and we would like to find a LGBTQ+

friendly physician, can you help with that?

**A.** Yes, the Alzheimer’s Association has partnered with the Gay and Lesbian Medical Association and has a list of physicians that LGBTQ+ friendly. You can find that list here: <https://lgbtqhealthcaredirectory.org/>

**Q.** Hi, I’m Asian and I’m caring for my Mom who has Alzheimer’s and trying to find resources for my

Dad. Where can I start?

**A.** The best place to start would be the Alzheimer’s Association – you can find resources for the Asian and Pacific Islander’s communities here: [https://www.alz.org/help-support/resources/asian-](https://www.alz.org/help-support/resources/asian-americans-and-alzheimers) [americans-and-alzheimers](https://www.alz.org/help-support/resources/asian-americans-and-alzheimers) you can also call the helpline and talk to an actual person who can help as well **800.272.3G00.**

**Q.** My parents are Vietnamese and I’m trying to get information on Alzheimer’s in their native

language, where can I find that?

**A.** Information about Alzheimer’s disease and various resources in Vietnamese can be found here: [https://alz.org/asian/overview.asp?nL=VICdL=VI](https://alz.org/asian/overview.asp?nL=VI&dL=VI) and there is also a phone number **800.272.3G00** ask if someone is there that can speak Vietnamese.

**Q.** I’m Native American and I found this by Googling – my Dad was just diagnosed with Alzheimer’s

disease, I need some help getting him culturally appropriate care. Where can I find it?

**A.** You can always start with the Alzheimer’s Association – here: [https://www.alz.org/help-](https://www.alz.org/help-support/resources/native-americans) [support/resources/native-americans](https://www.alz.org/help-support/resources/native-americans) as well as the National Indian Council on Aging here: <https://www.nicoa.org/> and the Indian Health Service here: <https://www.ihs.gov/aboutihs/>

**Q.** I’m First Nation and I need to make sure my mom is getting the care she needs for her Alzheimer’s disease; I know First Nation and Native American people often don’t even acknowledge dementia diseases – what can I do?

**A.** There is a blog on the Alzheimer’s Association website about that specific topic you can read it here: <https://www.alz.org/news/2021/their-mind-gets-fever> but also you can call their helpline **800.272.3G00** or just explore the Alzheimer’s Association website <https://www.alz.org/> to find resources to help – they can even guide you to local resources <https://www.communityresourcefinder.org/> close to you and your Mom. You can also watch the following videos to see what is being done in the Native American community. <https://youtu.be/ttYHEyNKwAY>

[
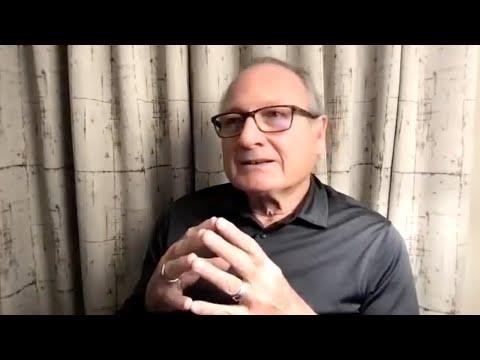
](https://youtu.be/ttYHEyNKwAY)

<https://youtu.be/aBhAldMFTFw>

[
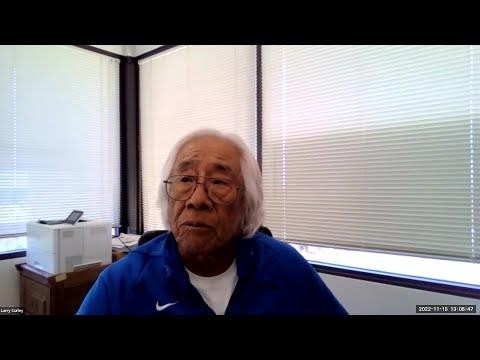
](https://youtu.be/aBhAldMFTFw)

**Q.** I heard Black Americans are twice as likely to get Alzheimer’s disease as White people why is that and why isn’t there more research into this?

**A.** Unfortunately, that statistic is correct, and we don’t have the answer to that question. As far as why there isn’t more research, there is significant evidence that Black American exclusion from clinical trials and discrimination from the medical establishment in the past has contributed to a high level of distrust of clinical trials. You can find more information about that here: <https://www.alz.org/help-support/resources/black-americans-and-alzheimers>

**Q.** My mom is a Black American and I’m interested in seeing if there is a clinical trial for Alzheimer’s

Disease that would treat her with respect while also, provide an opportunity for a new treatment.

**A.** There is a webinar you can watch called Changing the Face of Research – that discusses recruiting more people from diverse backgrounds here: [https://www.youtube.com/watch?v=wo0KuhRcACcCab_channel=actionalz](https://www.youtube.com/watch?v=wo0KuhRcACc&ab_channel=actionalz)

[
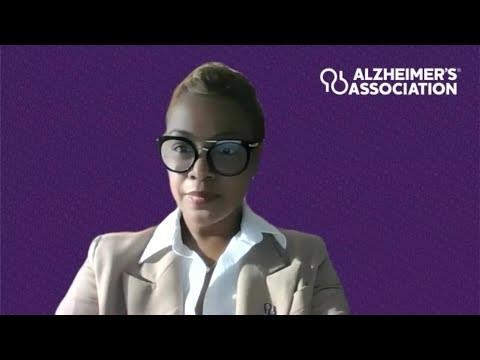
](https://www.youtube.com/watch?v=wo0KuhRcACc&ab_channel=actionalz)

And, if you are interested in finding a clinical trial you can try Trial Match here: <https://www.alz.org/alzheimers-dementia/research_progress/clinical-trials/trialmatch>

# Coping Style

**Q.** How do I cope with this? I don’t know what I’m doing, who to talk to? I’m so angry.

**A.** It sounds like you are experiencing some caregiver stress. Anger at an Alzheimer’s diagnosis is a natural reaction. It’s one of the 5 stages of grief as well. You are grieving what you know you will be losing. There are several things you can do. The first, is click this link and take a look at Caregiver Stress : <https://www.alz.org/help-support/caregiving/caregiver-health/caregiver-stress> The second is the ALZ Navigator – after answering a few short questions – you will be provided with an action plan to deal with your current emotions click here to start: [http://www.alz.org/help-](http://www.alz.org/help-support/resources/alznavigator) [support/resources/alznavigator](http://www.alz.org/help-support/resources/alznavigator) and finally, if you need help right now and want to talk to a human being you can call the helpline which is available 24/7 **800.272.3G00**

**Q.** People keep asking how I’m coping...how do they think I'm coping? I’m putting one foot in front of

the other and just dealing with it. How else would I cope?

**A.** Dealing with a diagnosis of Alzheimer’s is very difficult. There are many different ways to cope and many different expressions of caregiver stress. Follow this link here to read about caregiver stress: <https://www.alz.org/help-support/caregiving/caregiver-health/caregiver-stress> and if you need someone to talk to, please call the Helpline at **800.272.3G00**

**Q.** I usually deal with stress by crocheting, but I feel so upset about my spouse’s diagnosis that I can’t even find joy in it anymore. What can I do?

**A.** It is hard when you are overwhelmed by stress over a new diagnosis of Alzheimer’s disease. It is especially hard when your normal coping strategies do not work. There is support out there. You can try the ALZ Navigator – after answering a few questions, you will be provided with an action plan to deal with your current emotions, click here to start: [http://www.alz.org/help-](http://www.alz.org/help-support/resources/alznavigator) [support/resources/alznavigator](http://www.alz.org/help-support/resources/alznavigator) , you can also take a look at the online support groups – there might be a crochet group or at least people talking about crochet as an outlet you can find that here: <https://www.alz.org/help-support/community/support-groups> and you can always call the helpline if you need to talk to a person, they are available 24/7 **800.272.3G00.**

**Q.** My spouse is my best friend and the one I go to with everything and now I feel like I can’t because my spouse is what I’m worried about, my spouse is why I need coping help? I don’t know what to do?

**A.** It is hard when your person is the one you have concerns and worries about. There are resources available to help you. There are online support groups, local activities and support groups, and a helpline available 24/7. You can find the online support groups here: [https://www.alz.org/help-](https://www.alz.org/help-support/community/support-groups) [support/community/support-groups](https://www.alz.org/help-support/community/support-groups) you can find local activities and supports groups here: <https://www.communityresourcefinder.org/> and the helpline is **800.272.3G00.**

**Q.** When I find out bad news, such as my spouse’s Alzheimer’s disease diagnosis, I try to read everything I can about it, so I’m informed. Knowledge is power and it helps take some of the fear away.

**A.** There are a lot of resources online for you to read. You can start here with our publications: <https://www.alz.org/help-support/resources/publications> there are also virtual education opportunities that you can find here: <https://training.alz.org/> and finally you can subscribe to the e- newsletter which offers up to date information, tips, and latest news <https://www.alz.org/e-news>

**Q.** I usually lean on my family when I need help, how do I tell them about this Alzheimer’s

diagnosis? I want their support not their pity.

**A.** There are ways to talk to your family and friends. You can start here: [https://www.alz.org/help-](https://www.alz.org/help-support/i-have-alz/live-well/helping-family-friends) [support/i-have-alz/live-well/helping-family-friends](https://www.alz.org/help-support/i-have-alz/live-well/helping-family-friends) where we have tips for discussing an Alzheimer’s diagnosis with family members and friends and you can also call the Helpline and speak to a human being who will be able to discuss that with you as well **800.272.3G00.**

**Q.** I lean a lot on my faith in God when I have troubles. Do you have any suggestions for people of faith?

**A.** A lot of people rely on their faith in God and lean on Him during times of trouble. While the Alzheimer’s Association doesn’t have any specific information on their website, I recommend you reaching out to your church and faith community as a lot of churches have programs in place to help people in need. You can also call the Alzheimer’s Association helpline for assistance **800.272.3G00.**

**Q.** I like to exercise but I'm afraid to go for my walks with my spouse having memory problems? Can my spouse go with me? Is there someone that could sit with my spouse so I could go for a walk?

**A.** This is something you may want to discuss with your health care provider. If your spouse is physically able to walk with you, then there should be no issue with the two of you walking together. However, if your spouse is not physically able to walk with you, discuss with your health care provider options for how to keep your spouse safe during a walk – such as having a neighbor or friend come sit with your spouse while you go for your walk or even run some errands. You can also look here for local resources of respite care: <https://www.communityresourcefinder.org/>

**Q.** I like to cook a lot when I feel stressed. Is that something I can do with a spouse who has

Alzheimer’s? Are there dietary issues I need to be concerned about?

**A.** Yes, you most definitely can cook, especially if it’s something that you enjoy. It is important for you to take care of yourself by continuing to do things you enjoy. There is no specific dietary requirements for a person with Alzheimer’s beyond what their current physical health requires which you can discuss with your health care provider – such as a low carb, low fat diet for someone with diabetes and a heart healthy diet for someone with a history of heart disease. You can find more information here: <https://www.alz.org/help-support/caregiving/daily-care/food-eating>

**Q.** Are there groups I can join of other spouses of people with Alzheimer’s disease? Where would I find information about that? I feel like I need to be with people who understand what I’m going through.

**A.** Yes, the Alzheimer’s Association has online support groups tailored to all different populations you can find the online groups here: <https://alzconnected.org/> if you are looking for groups in your area you can start here: <https://www.communityresourcefinder.org/>
