# Supplementary material for: Empowering Informal Caregivers of Persons With Early-Stage Dementia by Large Language Models: Mixed Methods Evaluation
Source: JMIR Form Res. 2026 Mar 5;10:e79975. doi: 10.2196/79975 (PMC12978894; doi:10.2196/79975)
Supplement: Multimedia Appendix 3 [file formative-v10-e79975-s003.pdf]

Domain 1 - Cultural Values: <u>10Q&As</u>

Q1. I am Hispanic and my spouse was just diagnosed with Alzheimer's - how can I be sure I'm getting the care I need for my Hispanic spouse?
A.
The Alzheimer's Association has partnered with several agencies within different cultures to make sure care and help are provided appropriately. Your first resource would be to reach out to the National Hispanic Council on Aging and you can find them here: https://www.alz.org/about/our-commitment-to-diversity-and-inclusion#NHCOA or you can visit the Alzheimer's Association Hispanic sites here: http://alz.org/hispanic or here: http://alz.org/espanol.

Prompt:
How can I be sure I'm getting the care I need for my spouse? Please provide 5 most convenient care providers.
my spouse is Hispance
my spouse is diagnosed with Alzheimer's
the Alzheimer's Association has partnered with several agencies within different cultures to make sure care and help
the Alzheimer's Association has 24/7 helpline 800.272.3900
the National Hispanic Council on Aging is the first resource I can reach out to
Hispanic Americans and Alzheimer's has Alzheimer's Navigator program, Support groups and discussions in Spanish on ALZconnected
other resources include National Association of Hispanic Nurses, National Hispanic Medical Association and the Mexican Consulate and their Ventanilla de Salud(VDS) program
the Alzheimer's Association Hispanic sites here: http://alz.org/hispanic or here: http://alz.org/espanol

Attempt 1:
How can I be sure I'm getting the care I need for my spouse?
my spouse is Hispance
my spouse is diagnosed with Alzheimer's
the Alzheimer's Association has partnered with several agencies within different cultures to make sure care and help
the Alzheimer's Association has 24/7 helpline 800.272.3900
the National Hispanic Council on Aging is the first resource I can reach out to
Hispanic Americans and Alzheimer's has Alzheimer's Navigator program, Support groups and discussions in Spanish on ALZconnected
other resources include National Association of Hispanic Nurses, National Hispanic Medical Association and the Mexican Consulate and their Ventanilla de Salud(VDS) program

Ans:
It sounds like you're seeking culturally appropriate and comprehensive care for your Hispanic spouse who has been diagnosed with Alzheimer's. **You're already aware of some excellent resources, and I can help you organize these and suggest ways to ensure your spouse gets the care they need. Here's a structured approach:**
1. Use the Alzheimer's Association Resources
   - 24/7 Helpline: Call the Alzheimer's Association helpline at 800.272.3900 to speak with someone who can guide you toward personalized resources, including culturally tailored care options.
   - Alzheimer's Navigator: Use this program to create a personalized action plan for care management, addressing your spouse's unique needs.
   - Support Groups in Spanish: Join online or in-person support groups for Spanish-speaking families through ALZconnected to share experiences and gain support.
2. Connect with Culturally Specific Organizations
   - National Hispanic Council on Aging (NHCOA): As your first point of contact, NHCOA can provide guidance on local resources, support networks, and culturally competent care providers.
   - National Association of Hispanic Nurses (NAHN): They can help you find Spanish-speaking nurses and caregivers who understand your spouse's cultural background.

- National Hispanic Medical Association (NHMA): Contact this organization for referrals to Hispanic healthcare professionals who specialize in Alzheimer's care.

3. Utilize Community Programs
    - Mexican Consulate - Ventanilla de Salud (VDS): Visit the local Mexican Consulate's health window program for free health information, referrals, and guidance in Spanish.
    - Check with local Hispanic community centers or churches for additional support or care recommendations.
4. Advocate for Tailored Healthcare
    - Ensure all healthcare providers are aware of your spouse's cultural background and specific needs to offer culturally sensitive care.
    - Ask about bilingual staff or interpreters during medical appointments to help communicate effectively.
5. Engage in Long-term Care Planning
    - Explore legal and financial planning for Alzheimer's care with a focus on resources available to Hispanic communities.
    - Consider respite care or adult day care programs with Spanish-speaking staff to give you and your family breaks while ensuring your spouse is well cared for.
6. Educate Yourself and Your Family
    - Access Spanish-language educational materials about Alzheimer's to help your family better understand the disease and ways to support your spouse.
    - Attend workshops or webinars offered by Alzheimer's organizations that are designed for Hispanic caregivers.

These resources and steps can help ensure your spouse receives compassionate, culturally appropriate care while supporting you in this journey.

Attempt 2:

How can I be sure I'm getting the care I need for my spouse? Please provide 5 most convenient care providers.
my spouse is Hispance
my spouse is diagnosed with Alzheimer's
the Alzheimer's Association has partnered with several agencies within different cultures to
make sure care and help
the Alzheimer's Association has 24/7 helpline 800.272.3900
the National Hispanic Council on Aging is the first resource I can reach out to
Hispanic Americans and Alzheimer's has Alzheimer's Navigator program, Support groups and discussions in Spanish on ALZconnected
other resources include National Association of Hispanic Nurses, National Hispanic Medical Association and the Mexican Consulate and their Ventanilla de Salud(VDS) program

Ans:

It's wonderful that you're considering culturally appropriate care and support for your spouse's Alzheimer's diagnosis. Based on the details you provided, here are **five convenient care providers or resources** to ensure your spouse receives the care they need:

1. Alzheimer's Association

- **Services**: 24/7 helpline (800.272.3900), Alzheimer's Navigator program, support groups, and ALZconnected discussions in Spanish.
- **Why It's Convenient**: Nationwide resources and personalized support tailored for Hispanic families.
- **Next Step**: Call their helpline to discuss your spouse's needs and identify local services.

2. National Hispanic Council on Aging (NHCOA)

- **Services**: Advocacy, education, and resources for Hispanic older adults and their families.
- **Why It's Convenient**: They can guide you to community-based services specific to Hispanic Americans.
- **Next Step**: Contact NHCOA to learn about local programs that can support your spouse.

3. National Hispanic Medical Association (NHMA)

- **Services**: Network of Hispanic healthcare professionals and resources for culturally appropriate medical care.
- **Why It's Convenient**: They can connect you to healthcare providers experienced in treating Hispanic families.

- **Next Step**: Visit their website or contact them to find a local physician or specialist.

4. Mexican Consulate & Ventanilla de Salud (VDS)

- **Services**: Health promotion and disease prevention programs for the Hispanic community, including support for families dealing with chronic illnesses like Alzheimer's.
- **Why It's Convenient**: Available at Mexican Consulates across the U.S., providing easy access to resources in Spanish.
- **Next Step**: Contact your nearest Mexican Consulate to ask about VDS services.

5. National Association of Hispanic Nurses (NAHN)

- **Services**: Assistance finding Hispanic nurses and culturally appropriate healthcare.
- **Why It's Convenient**: NAHN can help locate bilingual healthcare providers.
- **Next Step**: Reach out to the NAHN to find qualified nurses familiar with Alzheimer's care.

Additional Tips:

- Join a **Spanish-speaking support group** through ALZconnected or other local networks.
- Utilize **caregiver resources and respite care services** offered by local agencies.
- Maintain open communication with your spouse's medical team about their evolving needs.

Would you like me to help you locate specific local providers or organizations?

Q2. How do I find a Hispanic doctor or nurse to help me with my spouse who has Alzheimer's? I want to make sure we have a doctor who understands our culture.
A. The Alzheimer's Association has partnered with the National Association of Hispanic Nurses and the National Hispanic Medical Association and is committed to providing all communities with appropriate care. You can find more information here:
https://www.alz.org/help-support/resources/hispanics-and-alzheimers
but you can also call the helpline and talk with an actual person who can help as well 800.272.3900.
Additional resources would be to reach out to the National Hispanic Council on Aging and you can find them here: https://www.alz.org/about/our-commitment-to-diversity-and-inclusion#NHCOA or you can visit the Alzheimer's Association Hispanic sites here: http://alz.org/hispanic or here: http://alz.org/espanol .

Prompt:

Attempt2:

How to find a Hispanic doctor or nurse to help me with my spouse? Please provide 4 most convenient resource.
my spouse has Alzheimer's
the doctor or nurse has to understand Hispanic culture
the Alzheimer's Association
the National Association of Hispanic Nurses
the National Hispanic Medical Association
a helpline 800.272.3900
the National Hispanic Council on Aging: https://www.alz.org/about/our-commitment-to-diversity-and-inclusion#NHCOA
the Alzheimer's Association Hispanic sites here: http://alz.org/hispanic or here: http://alz.org/espanol

Q3. My partner and I are concerned about getting the support we need as my partner was just diagnosed with Alzheimer's disease. We're worried that they won't respect our wishes and only listen to my partner's children, even though I have medical power of attorney.

A. I understand your concern. There are several resources for LGBTQ+ caregivers and their partners at the following link: https://www.alz.org/help-support/resources/alzheimers-and-dementia-resources-for-lgbtq-commun
Additionally, the online support group has specific forums just for LGTBQ+ caregivers and their partners which can be found here:
https://lgbtqhealthcaredirectory.org/

Prompt:

Attempt1:
Where can we get the support?
my partner was just diagnosed with Alzheimer's disease
I have medical power of attorney
we don' want our caregivers listen to my partner's children
the resources for LGBTQ+ caregivers and their partners are Dementia and the LGBTQ+ community, SAGE, SAGECare, Lambda Legal
Northern California and Northern Nevada
SAGE National LGBTQ+ Elder Hotline connects older adults with resources: 877.360.LGBT (5428)
Eldercare Locator helps care partners and care givers find care for older adults: 800.677.1116
the online support group has specific forums just for LGTBQ+ caregivers and their partners includes ALZConnected®

Ans:
I'm sorry to hear about your partner's diagnosis. It sounds like you're doing everything you can to find support and resources. Here are some targeted suggestions:

1. **SAGE National LGBTQ+ Elder Hotline**: Call 877.360.LGBT (5428) for resources specifically tailored to LGBTQ+ elders and their caregivers.
2. **Eldercare Locator**: Reach out at 800.677.1116 for help finding caregiving services in Northern California and Northern Nevada.
3. **ALZConnected®**: This online forum includes dedicated spaces for LGBTQ+ caregivers, offering peer support and shared experiences. You can join at ALZConnected.
4. **Local Alzheimer's Association Chapter**: They may provide LGBTQ+ specific caregiver resources, local support groups, and educational materials. Check their website or call their helpline at 800.272.3900.
5. **SAGECare**: Offers training and resources for caregivers working with LGBTQ+ seniors. Their website can help you ensure that any professional services you use are inclusive.
6. **Lambda Legal**: For questions about your rights as a medical power of attorney holder and to safeguard your partner's care preferences against interference, this organization provides guidance and legal resources.

If you need help navigating any of these resources, let me know!

Q4. My partner was just diagnosed with Alzheimer's disease, and we would like to find a LGBTQ+friendly physician, can you help with that?
A. Yes, the Alzheimer's Association has partnered with the Gay and Lesbian Medical Association and has a list of physicians that LGBTQ+ friendly. You can find that list here: https://lgbtqhealthcaredirectory.org/

Prompt:

Last Attempt:

Where can we find a LGBTQ+friendly physician? Please provide both online and offline resources.
#my partner was just diagnosed with Alzheimer's disease
the Alzheimer's Association
the Gay and Lesbian Medical Association
a list of physicians that LGBTQ+ friendly, LGBTQ+ Healthcare Directory
providers by approach are Trauma Informed Care, Sex Positive, Racial Equity, Informed Consent, Weight Inclusive, Harm Reduction
#. You can find that list here:https://lgbtqhealthcaredirectory.org/

Where can we find a LGBTQ+friendly physician? Please provide both online and offline resources.
#my partner was just diagnosed with Alzheimer's disease
the Alzheimer's Association
the Gay and Lesbian Medical Association
a list of physicians that LGBTQ+ friendly, LGBTQ+ Healthcare Directory
help types include LGBTQ-affirming Therapy, Family Medicine, Gender Affirming Hormone Therapy, Trans&Nonbinary Health, PrEP, Obstetrics and Gynecology
providers by approach are Trauma Informed Care, Sex Positive, Racial Equity, Informed Consent, Weight Inclusive, Harm Reduction

Q5. Hi, I'm Asian and I'm caring for my Mom who has Alzheimer's and trying to find resources for my Dad. Where can I start?
A. The best place to start would be the Alzheimer's Association – you can find resources for the Asian and Pacific Islander's communities here:
https://www.alz.org/help-support/resources/asian-americans-and-alzheimers
you can also call the helpline and talk to an actual person who can help as well 800.272.3900.
For local resources: https://www.communityresourcefinder.org or reach out to the local National Capital Area chapter here: https://www.alz.org/nca

Prompt:

Where can my dad find caring resources for my mom?
my mom has Alzheimer's
my dad is elder
I am Asian
resources for the Asian and Pacific Islander's communities
the Alzheimer's Association
helpline is 800.272.3900
local resources: https://www.communityresourcefinder.org
the local National Capital Area chapter here: https://www.alz.org/nca
For local resources: https://www.communityresourcefinder.org

Q6. My parents are Vietnamese and I'm trying to get information on Alzheimer's in their native language, where can I find that?
A. Information about Alzheimer's disease and various resources in Vietnamese can be found here:
https://alz.org/asian/overview.asp?nL=VI&dL=VI
and there is also a phone number 800.272.3900 ask if someone is there that can speak Vietnamese.
There is a video here about Alzheimer's in the Vietnamese population as well
https://www.youtube.com/watch?v=GCugHe1dHVs

Prompt:

Where can I find information on Alzheimer's? Please provide both online and offline resources.
my parents are Vietnamese
Alzheimer's association
alz.org, Vietnamese
helpline is 800.272.3900
a video here about Alzheimer's in the Vietnamese population as well:
https://www.youtube.com/watch?v=GCugHe1dHVs

Q7. I'm Native American and I found this by Googling – my Dad was just diagnosed with Alzheimer's disease, I need some help getting him culturally appropriate care. Where can I find it?
A. You can always start with the Alzheimer's Association – here:
https://www.alz.org/help-support/resources/native-americans
as well as **the National Indian Council on Aging** here:
https://www.nicoa.org/
and **the Indian Health Service** here: https://www.ihs.gov/aboutihs/

Prompt:

Where can I find culturally appropriate care for my dad?
my Dad was just diagnosed with Alzheimer's disease
I am Native American
the Alzheimer's Association
the National Indian Council on Aging
the Indian Health Service

Q8. I'm First Nation and I need to make sure my mom is getting the care she needs for her Alzheimer's disease; I know First Nation and Native American people often don't even acknowledge dementia diseases – what can I do?
A. There is a blog on the Alzheimer's Association website about that specific topic you can read it here: https://www.alz.org/news/2021/their-mind-gets-fever
but also you can call their helpline 800.272.3900 or just explore the Alzheimer's Association website https://www.alz.org/ to find resources to help – they can even guide you to local resources https://www.communityresourcefinder.org/ close to you and your Mom. You can also watch the following videos to see what is being done in the Native American community.
https://youtu.be/ttYHEyNKwAY
https://youtu.be/aBhAldMFTFw

Prompt:

Where can I get the care for my mom?
my mom has Alzheimer's disease
I am First Nation
First Nation and Native American people often don't even acknowledge dementia diseases
#a blog on the Alzheimer's Association website about that specific topic you can read it here:
https://www.alz.org/news/2021/their-mind-gets-fever
the Alzheimer's Association: https://www.alz.org/
helpline 800.272.3900
local resources close to my area
the Native American community
You can also watch the following videos to see what is being done in the Native American community.
https://youtu.be/ttYHEyNKwAY
https://youtu.be/aBhAldMFTFw

Q9. I heard Black Americans are twice as likely to get Alzheimer's disease as White people why is that and why isn't there more research into this?
A. Unfortunately, that statistic is correct, and we don't have the answer to that question. As far as why there isn't more research, there is significant evidence that Black American exclusion from clinical trials and discrimination from the medical establishment in the past has contributed to a high level of distrust of clinical trials. You can find more information about that here:
https://www.alz.org/help-support/resources/black-americans-and-alzheimers

Prompt:

Black Americans are twice as likely to get Alzheimer's disease as White people.
#1. why?
#2. why not more research into it?
#3. Black Americans and Alzheimers
#4. Black American exclusion from clinical trials and discrimination from the medical establishment
#You can find more information about that here:
https://www.alz.org/help-support/resources/black-americans-and-alzheimers

Q10. My mom is a Black American and I'm interested in seeing if there is a clinical trial for Alzheimer's Disease that would treat her with respect while also, provide an opportunity for a new treatment.
A. There is a webinar you can watch called Changing the Face of Research – that discusses recruiting more people from diverse backgrounds here:
https://www.youtube.com/watch?v=wo0KuhRcACc&ab_channel=actionalz
And, if you are interested in finding a clinical trial you can try Trial Match here:
https://www.alz.org/alzheimers-dementia/research_progress/clinical-trials/trialmatch

Prompt:

Where can I find a clinical trial and a new treatment for Alzheimer's disease for my mom?
#my mom is a Black American
I want my mom to be treated with respect
Changing the Face of Research
Trial Match
if you are interested in finding a clinical trial you can try Trial Match here:
https://www.alz.org/alzheimers-dementia/research_progress/clinical-trials/trialmatch

Domain 2 – Social Support: 10Q&As

Q1. Are there places I can go to socialize? I feel so isolated since my spouse was diagnosed with Alzheimer's disease. I just need to meet some other people – I don't even have time to go meet my friends.
A. Yes, there are places you can go and people you can talk to. You can start with the support groups online that you can find here: https://www.alz.org/help-support/community/support-groups you can find community resources close to you here: https://www.communityresourcefinder.org/ you can also call the Alzheimer's Association's hotline and talk to an actual person that can help you 800.272.3900.
Or you can click this link for resources specific to the National Capital Area (NCA) https://www.alz.org/nca

Prompt:

Where can I go to socialize?
my spouse was diagnosed with Alzheimer's disease
I feel isolated very much, I just need to meet some people
I don't have time to meet with my friends
online supporting groups
community resources
the Alzheimer's Association's hotline is 800.272.3900
the link for resources specific to the National Capital Area (NCA): https://www.alz.org/nca

Q2. How do I tell my family, our friends, that my spouse has Alzheimer's and I need help and support?
A. That is a common question for both caregivers and people with Alzheimer's or dementia. There is a very helpful page about talking with family, kids, grandkids, and friends. It is directed toward someone who has been diagnosed with Alzheimer's but maybe you and your spouse can sit down and look at it together and discuss how to move forward. You can find the link here:
https://www.alz.org/help-support/i-have-alz/live-well/helping-family-friends
you can call the Alzheimer's Association's hotline here: **800.272.3900** or reach out to the local National Capital Area chapter here: https://www.alz.org/nca

Prompt:

My spouse has Alzheimer's. How do I:
#1. tell my family
#2. tell our friends
#3. Ask for help and support : https://www.alz.org/help-support/i-have-alz/live-well/helping-family-friends
call the Alzheimer's Association's hotline here: **800.272.3900**
reach out to the local National Capital Area chapter here: https://www.alz.org/nca

Q3. I heard there was a thing called respite care? What is it, how do I find it, because I love my spouse but it has been a lot and I feel like I need a break.
A. This is very common and you are not alone, caregivers often need to take time for themselves so they can come back refreshed and ready to caregive again. Respite care is a temporary break for you from caregiving – it can be as simple as letting you take some time to go to a Dr. Appointment or even go get your hair cut without worrying about your spouse. There are many available resources, you can start here to find out more information about respite care:
https://www.alz.org/help-support/caregiving/care-options/respite-care
To find respite care in your area check here and enter
some information to be guided to local resources: https://www.communityresourcefinder.org

Prompt:

Regarding respite care,
#1. What is it?
#2. How do I find it?
online resource: https://www.alz.org/help-support/caregiving/care-options/respite-care
local resources: https://www.communityresourcefinder.org

Q4. Where can I meet and talk with other caregivers and spouses? I feel like only people who have walked in my shoes can understand what I'm going through.
A. You can start with the online support groups through the Alzheimer's Association here:
https://alzconnected.org/ and then you can investigate further and find local events that can connect you with others in your area here:
https://www.alz.org/help-support/community/support-groups or here: https://www.communityresourcefinder.org
or you can call the Alzheimer's Association's hotline here: **800.272.3900** or reach out to the local National Capital Area chapter here: https://www.alz.org/nca

Prompt:

Where can I meet and talk with other caregivers and spouses? Both online and offline resources.
my spouse has Alzheimer's disease
the Alzheimer's Association: https://alzconnected.org/
local events: https://www.alz.org/help-support/community/support-groups or
https://www.communityresourcefinder.org

call the Alzheimer's Association's hotline here: **800.272.3900**
reach out to the local National Capital Area chapter here: https://www.alz.org/nca

Q5. I'm not really good with all this online stuff, is there a number I can call to talk to a human being who can help me find local support or even just give me some information over the phone?
A. Yes, there is. You can call 800.272.3900 24/7 and speak to a live human being who can answer your questions and provide other resource numbers for you and the call, and the assistance is free.

Prompt:

Is there a number I can call to talk to a human being?
help me find local support or even
I prefer to speak to a human being , call 800.272.3900 24/7
other resource numbers

Q6. Are there places my spouse and I can go to interact with other couples dealing with early-stage Alzheimer's? A lot of the support groups I'm seeing are people that have been diagnosed for a couple years, I'd really like to meet up with people in our area who have just received their diagnosis or only been diagnosed for a year or so.
A. Yes, there are early-stage social engagement activities, click on the link and you'll have to enter some location information for activities near you.
https://www.alz.org/events/event_search?etid=8&cid=0
or you can call the Alzheimer's Association's hotline here: **800.272.3900** or reach out to the local National Capital Area chapter here: https://www.alz.org/nca

Prompt:

How can we interact with other couples?
our area
local support group resources
dealing with early-stage Alzheimer's
early-stage social engagement activities: https://www.alz.org/events/event_search?etid=8&cid=0
call the Alzheimer's Association's hotline here: **800.272.3900**
reach out to the local National Capital Area chapter here: https://www.alz.org/nca

Q7. I never thought we would experience stigma, but the grandkids are afraid to hug my spouse for fear they'll catch Alzheimer's and even some of our friends have stopped engaging with us as often. How do I educate them about Alzheimer's?
A. Sadly, you are not alone in this. Many caregivers and people with Alzheimer's face stigma from the most surprising sources. You can start here:
https://www.alz.org/help-support/i-have-alz/overcoming-stigma
and you can always call the Helpline for more resources and ideas to
discuss with your friends and family 800.272.3900.

Prompt:

How do I educate my friends and family about Alzheimer's?
me and my spouse experience Alzheimer's stigma
my grandkids are afraid to hug my spouse
some of my friends stopped engaging with us as often

support resource: https://www.alz.org/help-support/i-have-alz/overcoming-stigma
helpline: 800.272.3900
#reach out to the local National Capital Area chapter here: https://www.alz.org/nca

Q8. My spouse and I regularly attend church, it is a huge source of social support for us. Is that something we can still do? Do churches have any programs to help people like us?
A. A lot of people find comfort in their faith and their places of worship. While the Alzheimer's Association does not have specific information about faith-based help, check with your church, most churches do have programs to help their parishioners in need. Call the church office or the ministry office and speak to them about your concerns. You can always call the Helpline and speak to a person and see if they have any local recommendation for you 800.272.3900.

Prompt:

My spouse and I are diagnosed Alzheimer's.
#1. can we still attend church as usual?
#2. do churches have support programs for us?
#call the church office or the ministry office
#call the helpline at 800.272.3900 for recommendation

Q9. I play bridge with a group of friends, can I still go do that? I'm concerned about leaving my spouse by themselves while I go off to play bridge, but it is one of my main sources of social support.
A. Maintaining your social activities and sources of support is important for you as a caregiver because it helps alleviate stress and relieve some of your caregiver burden. However, you should discuss with your spouse and their health care provider if leaving them alone is safe for a long time at this stage. You may need to find a neighbor or friend who is willing to come and sit with your spouse or consider having the bridge group meet at your house instead.
There are early-stage social engagement activities, click on the link and you'll have to enter some location information for activities near you. https://www.alz.org/events/event_search?etid=8&cid=0 or you can call the Alzheimer's Association's hotline here: **800.272.3900** or reach out to the local National Capital Area chapter here: https://www.alz.org/nca or you could look into respite care. Respite care is a temporary break for you from caregiving – it can be as simple as letting you take some time to go to a Dr. Appointment or even go get your hair cut without worrying about your spouse. There are many available resources, you can start here to find out more information about respite care: https://www.alz.org/help-support/caregiving/care-options/respite-care To find respite care in your area check here and enter some information to be guided to local resources:

https://www.communityresourcefinder.org or you can call the Alzheimer's Association's hotline here: **800.272.3900** or reach out to the local National Capital Area chapter here: https://www.alz.org/nca

Prompt:
How do I balance between my social activities and caregiving my spouse?
my spouse has Alzheimer's
I like playing bridge
I need to maintain social activities
I need to discuss with my spouse
I need to talk to the health care provider
I can ask friends to come to my house
early-stage social engagement activities: https://www.alz.org/events/event_search?etid=8&cid=0
call the Alzheimer's Association's hotline here: **800.272.3900**
reach out to the local National Capital Area chapter here: https://www.alz.org/nca
look into respite care
available resources, you can start here to find out more information about respite care: https://www.alz.org/help-support/caregiving/care-options/respite-care
#To find respite care in your area check here and enter some information to be guided to local resources: https://www.communityresourcefinder.org
reach out to the local National Capital Area chapter here: https://www.alz.org/nca

Q10. I still work and I enjoy my work, it is a source of social support for me. I'm worried I'll have to retire to take care of my spouse, what should I do?
A. You have multiple options available to you.
First, talk with your spouse and their health care provider on just how much supervision is needed at this stage in the disease process.
Second, discuss things with your employer and see if there is an option for some remote work fi possible. If the health care provider indicates supervision is needed for your spouse you may have to consider Adult Day Care or an in-home care provider while you are working, the organizations can help you with insurance and how to pay for this service. You can read more information about these options here: https://www.alz.org/help-support/caregiving/care-options . You can also call the Alzheimer's Association's hotline here: **800.272.3900** or reach out to the local National Capital Area chapter here: https://www.alz.org/nca for help finding local resources.

Prompt:

My spouse has Alzheimer's, what should I do?
#I still work
#I enjoy my work as social support
#I don't want to retire to take care of my spouse
#remote work
#health care provider advice
Adult Day Care or an in-home care provider while you are working: : https://www.alz.org/help-support/caregiving/care-options
call the Alzheimer's Association's hotline here: **800.272.3900**
reach out to the local National Capital Area chapter here: https://www.alz.org/nca for help finding local resources

Domain 3 – Dementia Literacy: <u>10Q&As</u>

Q1. My spouse has just been diagnosed with dementia – what do I do?
A. A dementia diagnosis is life-changing and will take time to process, but there are resources available. You can start here:
https://www.alz.org/help-support/caregiving/stages-behaviors/accepting_the_diagnosis

Prompt:

What do I do?
#my spouse has just been diagnosed with dementia
#online resource: https://www.alz.org/help-support/caregiving/stages-behaviors/accepting_the_diagnosis
#support resources

Q2. What is Alzheimer's Disease? What do I tell my family?
A. Alzheimer's Disease is the most common form of dementia – more information here:
https://www.alz.org/alzheimers-dementia/what-is-alzheimers .
Regarding what to tell your family, remember Alzheimer's Disease is nobody's fault and there is help available. For help telling your family you can start here:
https://www.alz.org/help-support/i-have-alz/know-what-to-expect/sharing-your-diagnosis

Prompt:

Regarding Alzheimer's Disease,
#1. What is it?
#2. How to educate my family?
#3. Learning resource: https://www.alz.org/alzheimers-dementia/what-is-alzheimers
#4. Support resource: https://www.alz.org/help-support/i-have-alz/know-what-to-expect/sharing-your-diagnosis

Q3. What are the early symptoms or warning signs of Alzheimer's?
A. Early symptoms include but are not limited to: Memory Loss, Difficulties in daily tasks, challenges in solving problems – for more details click here:
https://www.alz.org/alzheimers-dementia/10_signs

Prompt:

What do you know about Alzheimer's?
#1. What are the early symptoms
#2. What are the warning signs
#3. Learning resources: https://www.alz.org/alzheimers-dementia/10_signs

Q4. How is Alzheimer's or dementia treated?
A. There are many treatment options available including medications for memory, cognitive disfunction, as well as behavioral disorders – more info is available here:
https://www.alz.org/alzheimers-dementia/treatments

Prompt:

How to treat Alzheimer's or dementia?
#Learning resources: https://www.alz.org/alzheimers-dementia/treatments

Q5. I've heard insurance won't cover my spouse's treatment, is that true?
A. There have been some restrictions placed on treatment coverage, but with cooperation between you and your physician, some coverage may be available – check here for more information:
https://www.alz.org/alzheimers-dementia/treatments/cms-medicare-coverage

Prompt:

Insurance won't cover my spouse's Alzheimer's treatment.
#1.Is this true?
#2. How to find treatment coverage?
#3. Learning resources: https://www.alz.org/alzheimers-dementia/treatments/cms-medicare-coverage

Q6. How can I learn more about Alzheimer's and dementia – are there online courses I can take? I'm afraid of this happening to me and I want to know what to watch out for.
A. There are online courses with the Alzheimer's Association – including "Understanding Alzheimer's and Dementia" **https://training.alz.org/products/4053/understanding-alzheimers-and-dementia ?** and "Knowing the 10 signs – Early Detection Matters"
https://training.alz.org/products/4062/10-warning-signs-of-alzheimers ? .

Prompt:

Are there online courses about Alzheimer's and dementia?
#1.I want to know what to watch out for
#2. Online learning resources
#3. the Alzheimer's Association – including "Understanding Alzheimer's and Dementia" https://training.alz.org/products/4053/understanding-alzheimers-and-dementia ? and "Knowing the 10 signs – Early Detection Matters"
https://training.alz.org/products/4062/10-warning-signs-of-alzheimers ?

Q7. What kind of tests can be done to test for Alzheimer's or dementia early?
A. While this area is still being researched, promising results have been shown in FDA approved brain imaging, but other tests are currently being researched including blood work and other biomarkers – there is more information regarding the current tests here:
https://www.alz.org/alzheimers-dementia/research_progress/earlier-diagnosis

Prompt:

What kind of tests can be done to test for Alzheimer's or dementia early?
#brain imaging
#blood work
#other biomarkers
#learning resource: https://www.alz.org/alzheimers-dementia/research_progress/earlier-diagnosis

Q8. What can I do to prevent Alzheimer's from happening to me too?
A. While there is no specific preventative path to guard against Alzheimer's or dementia, there are several things you can do now to decrease risk, including physical activity, a heart healthy diet, social activities, and keeping mentally active. More information can be found here:
https://www.alz.org/alzheimers-dementia/research_progress/prevention

Prompt:

How to prevent from obtaining Alzheimer's?
learning resources: https://www.alz.org/alzheimers-dementia/research_progress/prevention
physical activity
a heart healthy diet
social activities
keeping mentally active.
More information can be found here:
https://www.alz.org/alzheimers-dementia/research_progress/prevention

Q9. My grandkids are afraid they are going to catch Alzheimer's from us. How can we reassure them that won't happen?
A. It is not uncommon for younger children to be afraid of diseases they don't understand. You can find more information here
https://www.alz.org/help-support/i-have-alz/live-well/helping-family-friends
about talking to your family and especially the younger members of your family and
reassuring them that Alzheimer's is not contagious.

Prompt:

How to educate my family about Alzheimer's?
my grandkids are afraid to catch it
learning resources: https://www.alz.org/help-support/i-have-alz/live-well/helping-family-friends

Q10. I have the ironic problem of my family fighting over who is going to help me take care of my spouse. Never thought that would be a problem but here I am. What can I do?
A. Unfortunately, an Alzheimer's diagnosis can bring out strong feelings and sometimes conflicting feelings in family members. This can be stressful for the person with Alzheimer's and their spouse who is often left as a referee. There is information available online regarding family conflicts here:
https://www.alz.org/help-support/resources/resolving-family-conflicts

Prompt:

How to deal with family conflicts regarding caregiving a family member with Alzheimer's?
#family members fight with the caregivers
#learning resources: https://www.alz.org/help-support/resources/resolving-family-conflicts

Domain 4 – Coping Style: 10Q&As

Q1. How do I cope with this? I don't know what I'm doing, who to talk to? I'm so angry.
A. It sounds like you are experiencing some caregiver stress. Anger at an Alzheimer's diagnosis is a natural reaction. It's one of the 5 stages of grief as well. You are grieving what you know you will be losing. There are several things you can do. The first, is click this link and take a look at Caregiver Stress : https://www.alz.org/help-support/caregiving/caregiver-health/caregiver-stress
The second is the ALZ Navigator – after answering a few short questions – you will be provided with an action plan to deal with your current emotions click here to start: http://www.alz.org/help-support/resources/alznavigator
and finally, if you need help right now and want to talk to a human being you can call the helpline which is available 24/7 800.272.3900

Prompt:

How do I cope with caregiver stress?
5 stages of grief on Alzheimer's diagnosis
# Refer to Caregiver Stress : https://www.alz.org/help-support/caregiving/caregiver-health/caregiver-stress
Use ALZ Navigator, action plan to deal with emotions: http://www.alz.org/help-support/resources/alznavigator
Use helpline: 24/7, 800.272.3900

Q2. People keep asking how I'm coping...how do they think I'm coping? I'm putting one foot in front of the other and just dealing with it. How else would I cope?
A. Dealing with a diagnosis of Alzheimer's is very difficult. There are many different ways to cope and many different expressions of caregiver stress. Follow this link here to read about caregiver stress:
https://www.alz.org/help-support/caregiving/caregiver-health/caregiver-stress
and if you need someone to talk to, please call the Helpline at 800.272.3900

Prompt:
How do I cope with a diagnosis of Alzheimer's?
learning resources regarding caregiver stress: https://www.alz.org/help-support/caregiving/caregiver-health/caregiver-stress
helpline at 800.272.3900

Q3. I usually deal with stress by crocheting, but I feel so upset about my spouse's diagnosis that I
i can't even find joy in it anymore. What can I do?
A. It is hard when you are overwhelmed by stress over a new diagnosis of Alzheimer's disease. It is especially hard when your normal coping strategies do not work. There is support out there. You can try the ALZ Navigator – after answering a few questions, you will be provided with an action plan to deal with your current emotions, click here to start:
http://www.alz.org/help-support/resources/alznavigator , you can also take a look at the online support groups – there might be a crochet group or at least people talking about crochet as an outlet you can find that here:
https://www.alz.org/help-support/community/support-groups
and you can always call the helpline if you need to talk to a person, they are available 24/7 800.272.3900.

Prompt:
What can I do?
I feel so upset about my spouse's diagnosis of Alzheimer's
I usually deal with stress by crocheting
support from ALZ Navigator, an action plan to deal with your current emotions: http://www.alz.org/help-support/resources/alznavigator
the online support groups – there might be a crochet group or at least people talking about crochet as an outlet you can find that here:
https://www.alz.org/help-support/community/support-groups
call the helpline if you need to talk to a person, they are available 24/7 800.272.3900.

Q4. My spouse is my best friend and the one I go to with everything and now I feel like I can't because my spouse is what I'm worried about, my spouse is why I need coping help? I don't know what to do?
A. It is hard when your person is the one you have concerns and worries about. There are resources available to help you. There are online support groups, local activities and support groups, and a helpline available 24/7. You can find the online support groups here: https://www.alz.org/help-support/community/support-groups
you can find local activities and supports groups here:
https://www.communityresourcefinder.org/
and the helpline is 800.272.3900.

Prompt:
What should I do?
my spouse is diagnosed with Alzheimer's
resources available to help: https://www.alz.org/help-support/community/support-groups
local activities and supports groups: https://www.communityresourcefinder.org/
helpline available for 24/7 at 800.272.3900

Q5. When I find out bad news, such as my spouse's Alzheimer's disease diagnosis, I try to read everything I can about it, so I'm informed. Knowledge is power and it helps take some of the fear away.
A. There are a lot of resources online for you to read. You can start here with our publications: https://www.alz.org/help-support/resources/publications there are also virtual education opportunities that you can find here: https://training.alz.org/ and finally you can subscribe to the e-newsletter which offers up to date information, tips, and latest news
https://www.alz.org/e-news

Prompt:

Where to find learning resources regarding Alzheimer's?
my spouse was diagnosed as Alzheimer's
resources online: https://www.alz.org/help-support/resources/
virtual education opportunities: https://training.alz.org/
subscribe to the e-newsletter which offers up to date information, tips, and latest news https://www.alz.org/e-news

Q6. I usually lean on my family when I need help, how do I tell them about this Alzheimer's diagnosis? I want their support not their pity.
A. There are ways to talk to your family and friends. You can start here: https://www.alz.org/help-support/i-have-alz/live-well/helping-family-friends
where we have tips for discussing an Alzheimer's diagnosis with family members and friends and you can also call the Helpline and speak to a human being who will be able to discuss that with you as well 800.272.3900.

Prompt:

How do I tell my family about my Alzheimer's?
ways to talk to your family and friends: https://www.alz.org/help-support/i-have-alz/live-well/helping-family-friends
tips for discussing an Alzheimer's diagnosis with family members and friends
helpline at 800.272.3900.

Q7. I lean a lot on my faith in God when I have troubles. Do you have any suggestions for people of faith?
A. A lot of people rely on their faith in God and lean on Him during times of trouble. While the Alzheimer's Association doesn't have any specific information on their website, I recommend you reaching out to your church and faith community as a lot of churches have programs in place to help people in need. You can also call the Alzheimer's Association helpline for assistance
800.272.3900.

Prompt:

Do you have any suggestions for people of faith?
Alzheimer's disease
the Alzheimer's Association doesn't have any specific information on their website
reach out to your church and faith community for help
call the Alzheimer's Association helpline for assistance 800.272.3900.

Q8. I like to exercise but I'm afraid to go for my walks with my spouse having memory problems? Can my spouse go with me? Is there someone that could sit with my spouse so I could go for a walk?
A. This is something you may want to discuss with your health care provider. If your spouse is physically able to walk with you, then there should be no issue with the two of you walking together. However, if your spouse is not physically able to walk with you, discuss with your health care provider options for how to keep your spouse safe during a walk – such as having a neighbor or friend come sit with your spouse while you go for your walk or even run some errands. You can also look here for local resources of respite care:
https://www.communityresourcefinder.org/

Prompt:

Is there someone that could sit with my spouse so I could go for a walk?
my spouse has memory problems
my spouse was diagnosed Alzheimer's
discuss with your health care provider for options
how to keep your spouse safe during a walk
local resources of respite care: https://www.communityresourcefinder.org/

Q9. I like to cook a lot when I feel stressed. Is that something I can do with a spouse who has Alzheimer's? Are there dietary issues I need to be concerned about?
A. Yes, you most definitely can cook, especially if it's something that you enjoy. It is important for you to take care of yourself by continuing to do things you enjoy. There is no specific dietary requirements for a person with Alzheimer's beyond what their current physical health requires which you can discuss with your health care provider – such as a low carb, low fat diet for someone with diabetes and a heart healthy diet for someone with a history of heart disease. You can find more information here:
https://www.alz.org/help-support/caregiving/daily-care/food-eating

Prompt:

When my spouse has Alzheimer's,
can I still cook for releasing stress?
are there dietary issues I need to be concerned about?
no specific dietary requirements for a person with Alzheimer's beyond what their current physical health requires
discuss with your health care provider – such as a low carb, low fat diet for someone with diabetes and a heart healthy diet for someone with a history of heart disease
learning resources: https://www.alz.org/help-support/caregiving/daily-care/food-eating

Q10. Are there groups I can join of other spouses of people with Alzheimer's disease? Where would I find information about that? I feel like I need to be with people who understand what I'm going through.
A. Yes, the Alzheimer's Association has online support groups tailored to all different populations you can find the online groups here: https://alzconnected.org/ if you are looking for groups in your area you can start here: https://www.communityresourcefinder.org/

Prompt:

Where would I find groups I can join of other spouses of people with Alzheimer's disease?
I need to be with people who understand what I'm going through
the Alzheimer's Association has online support group: https://alzconnected.org/
groups in your area: https://www.communityresourcefinder.org/

Domain 5 - Digital Health Literacy: 10Q&As

Q1. I don't know how to do all this new-fangled computer stuff, am I talking to a robot right now? Where can I learn how to look stuff up about my health?

A. ~~Hi, yes, you are talking to an artificial intelligence platform. Feel free to type your questions in the box provided and I will try to answer them for you or give you a link to click that will help you find them. It is my job to help you find the most reliable and up to date information for your questions.~~

You can start here for general information regarding your health https://www.alz.org/help-support/caregiving/caregiver-health you should also check with your healthcare provider and see if they have a patient portal and can help you access it. Also, a lot of communities have classes through community centers or the YMCA that will help you, try looking at AARP first, all you have to do is put in your zip code, their link is here https://my.aarpfoundation.org/locator/digital-skills-ready/

Prompt:

How to look up a computer stuff about my health?
#general information regarding your health https://www.alz.org/help-support/caregiving/caregiver-health
#check with your healthcare provider and see if they have a patient portal and can help you access it
#communities have classes through community centers or the YMCA
#looking at AARP first, put in your zip code, the link is https://my.aarpfoundation.org/locator/digital-skills-ready/

Q2. I need to find some answers about my spouse's Alzheimer's diagnosis – where should I look first?
A. Hi, you can type your questions here and I will try to answer them, and I can also provide you with reliable links to websites that can give you additional information. A good place to start is the Alzheimer's Association located here: https://www.alz.org/

Prompt:
Where to go to find some answers about my spouse's Alzheimer's diagnosis?
A good place to start is the Alzheimer's Association located here: https://www.alz.org/

Q3. All this technology and information on the internet is so confusing – I don't know which sites to believe – some say to use supplements and others say supplements are worthless – who do I trust?
A. Hi. There is a lot of confusing information out on the internet, I can help you by providing you with reliable and up to date information as well as links to respected resources. Regarding your question of supplements, according to the Alzheimer's Association there is no single food, drink, or supplement that has been recommended for the prevention or treatment of Alzheimer's. For more information you can explore here: https://www.alz.org/help-support/brain_health/adopt_a_healthy_diet

Prompt:
reliable and up to date information on supplements:
according to the Alzheimer's Association there is no single food, drink, or supplement that has been recommended for the prevention or treatment of Alzheimer's
you can explore by the link: https://www.alz.org/help-support/brain_health/adopt_a_healthy_diet

Q4. I'm so afraid of scams on the internet and on my phone, how do I know I'm looking at a legitimate Alzheimer's support group in my area?
A. The old adage from the U.S. Post Office regarding mail fraud is still one of the best pieces of advice regarding scams: "If it sounds/looks too good to be true, it probably is." Regarding your specific concern about support groups – you can find an online community here: https://alzconnected.org/ or you can go to the following link and input your zip code for groups in your area: https://www.alz.org/events/event_search?etid=2&cid=0

Prompt:

How to find a legitimate Alzheimer's support group in my area?
an online community here: https://alzconnected.org/
go to the following link and input your zip code for groups in your area: https://www.alz.org/events/event_search?etid=2&cid=0

Q5. I was on what I thought was the right website for Alzheimer's information but they asked for a bunch of personal information like addresses and credit card numbers so I closed the window.
A. Unfortunately, there are scam sites out there on the internet – the official site for the Alzheimer's Association is here: https://www.alz.org/ If you ever give out your credit card company to a suspicious website or person, you need to call your credit card company immediately and talk to their fraud department. Don't ever give out your personal information to a website, reply to an email, a text message, or a phone call that you have any doubts about. Contact the companies directly with known email addresses, websites, or phone numbers before releasing any of your private information. Most companies will NOT solicit that information via email, mail, or blind phone calls.

Prompt:

Regarding the right website for Alzheimer's information:
the official site for the Alzheimer's Association is https://www.alz.org/.
Don't ever give out your personal information to a website, reply to an email, a text message, or a phone call that you have any doubts about.
Contact the companies directly with known email addresses, websites, or phone numbers before releasing any of your private information.
Most companies will NOT solicit that information via email, mail, or blind phone calls.

Q6. The health insurance company said I need to create some sort of online account? How do I do that? Is there someone that can help me? Can you help me?
A. Yes, a lot of health care companies and other companies have shifted operations to online and you need an online account to access them. Insurance companies often have a phone number you can call and tech support that can guide you through the process of setting up your account. Type in the name of your insurance company and I can direct you to their website where you can find the appropriate number. (CAN WE DO THIS?) As a last resort, you can try the Alzheimer's Association helpline and see if they can talk you through it… **800.272.3900.**

Prompt:

How do I create an online account?
health care companies and other companies have shifted operations to online
Insurance companies often have a **phone number** you can call
Insurance companies often have **tech support** that can guide you through the process of setting up your account
Type in the name of your insurance company for the corresponding website and find the appropriate number
you can try the Alzheimer's Association helpline by **800.272.3900.**

Q7. I need help navigating health insurance and Medicare? Medicare B? Medicare D? What does all that information mean? And I need it explained in language that I can understand.
A. Financial concerns are common among caregivers of people with Alzheimer's. There are online resources that can help. Start here:
https://www.alz.org/help-support/i-have-alz/plan-for-your-future/financial_planning
There are links on that page to help explain both Medicare and Medicare B and Medicare D.

Prompt:

The guidance on navigating health insurance and Medicare for the caregivers of people with Alzheimer's:
#the online resources that can help: https://www.alz.org/help-support/i-have-alz/plan-for-your-future/financial_planning
Medicare
Medicare B
Medicare D

Q8. The hospital told me I needed to have an advanced directive...I think I know what that is, but how do I find information about them? Attorneys are expensive and they charge by the hour I don't have the money to pay an attorney to explain all that to me, I want to know what I need to do before I walk into an attorney's office.
A. The Alzheimer's Association has information about all of those things online. You can find it here: https://www.alz.org/help-support/i-have-alz/plan-for-your-future/legal_planning
if you have further questions you can reach out to their Helpline which is available 24/7 at 800.272.3900.

Prompt:

How do I find information about have an advanced directive?
Attorneys are expensive and they charge by the hour I don't have the money to pay an attorney to explain all that to me, I want to know what I need to do before I walk into an attorney's office.
The Alzheimer's Association has information about all of those things online.
You can find it here: https://www.alz.org/help-support/i-have-alz/plan-for-your-future/legal_planning
if you have further questions you can reach out to their Helpline which is available 24/7 at 800.272.3900.

Q9. What is the difference between a Power of Attorney and a Power of Attorney for Health Care? I tried Googling it and it was just too confusing. Is there a trusted source you can link me to?
A. A Power of Attorney for Health care is someone who can make health care decisions for you in the case where you cannot make those decisions for yourself – such as when you have dementia or you are unconscious. You can read more information about both of those here:
https://www.alz.org/help-support/i-have-alz/plan-for-your-future/legal_planning

Prompt:
What is the difference between a Power of Attorney and a Power of Attorney for Health Care?
A Power of Attorney for Health care is someone who can make health care decisions for you in the case where you cannot make those decisions for yourself – such as when you have dementia or you are unconscious.
You can read more information about both of those here:
https://www.alz.org/help-support/i-have-alz/plan-for-your-future/legal_planning

Q10. My spouse has Alzheimer's disease, high blood pressure, and diabetes. Where can I find information about my spouse's medications and if there are any interactions?
A. One of the best resources you have regarding medication interactions is your pharmacist. If you want to just quickly look at a specific drug to understand it you can go here and enter the drug name and choose the dosage out of the drop-down menu: https://www.cvs.com/druginfo/ but again, you should check with your pharmacist for more specific information or your health care provider.

Prompt:

Where can I find information about my spouse's medications and if there are any interactions?
My spouse has Alzheimer's disease, high blood pressure, and diabetes.
One of the best resources you have regarding medication interactions is your pharmacist.
If you want to just quickly look at a specific drug to understand it you can go here and enter the drug name and choose the dosage out of the drop-down menu: https://www.cvs.com/druginfo/
you should check with your pharmacist for more specific information or your health care provider.

Domain 6 – Caregiver Health: 10Q&As

Q1. I'm not in the best health, how can I take care of my spouse and make sure my health needs are met too.
A. Taking care of a spouse requires you to take care of yourself too, a common phrase is "a**".** You can do this by seeing your doctor regularly, being physically active, and eating healthfully. There are some tips for these as well as how to cope here:
https://www.alz.org/help-support/caregiving/caregiver-health/be_a_healthy_caregiver

Prompt:

My spouse has Alzheimer's, how could I take care of both of us?
seeing your doctor regularly, being physically active, and eating healthfully
some tips for these as well as how to cope here:
https://www.alz.org/help-support/caregiving/caregiver-health/be_a_healthy_caregiver

Q2. I'm feeling depressed, this whole caregiver thing is hard, and I feel alone.
A. You are not alone, these feelings are very normal and common amongst caregivers. You can find help for depression, stress, and tips to cope by starting here:
https://www.alz.org/help-support/caregiving/caregiver-health/caregiver-depression

Prompt:

As a caregiver, how to avoid feeling depressed and alone?
You can find help for depression, stress, and tips to cope by starting here:
https://www.alz.org/help-support/caregiving/caregiver-health/caregiver-depression

Q3. I've heard there are online support groups, but I have no idea which one to choose? How do I know these aren't scam groups?
A. There are reliable online support groups. ALZConnected is a reliable online support group for caregivers such as yourself – you can start here by creating an account:
https://alzconnected.org
You can also scroll down to the bottom and enter your zip code for a list of local support events for Alzheimer's & dementia caregivers.

Prompt:

Which online support group to choose for avoiding scams?
ALZConnected is a reliable online support group for caregivers
creating an account for ALZConnected by https://alzconnected.org
scroll down to the bottom of website https://alzconnected.org and enter your zip code for a list of local support events for Alzheimer's & dementia caregivers

Q4. I'm feeling an enormous amount of stress caring for my spouse, are there any available resources because I'm afraid of getting burned out.
A. This is completely understandable. Taking care of someone with Alzheimer's and dementia can lead to an enormous amount of stress which is not good for your health or that of your spouse. Make taking care of yourself one of your priorities, exercise (even a simple walk) can alleviate stress. Meditation or deep breathing exercises are also good for reducing stress. There are community resources available through the Alzheimer's Association Community Resource Finder (located here: http://www.communityresourcefinder.org), as well as your local chapter https://www.alz.org/local_resources/find_your_local_chapter .
Additional information regarding caregiver stress can be found here:
https://www.alz.org/help-support/caregiving/caregiver-health/caregiver-stress

Prompt:

What are the resources that I can use to avoid enormous amount of stress caring for my spouse, who has Alzheimer's :
taking care of yourself one of your priorities, exercise (even a simple walk) can alleviate stress
Meditation or deep breathing exercises are good for reducing stress
community resources available through the Alzheimer's Association Community Resource Finder:
http://www.communityresourcefinder.org
your local chapter
https://www.alz.org/local_resources/find_your_local_chapter .
information regarding caregiver stress can be found here:
https://www.alz.org/help-support/caregiving/caregiver-health/caregiver-stress

Q5. My spouse is no longer interested in being intimate with me, did I do something wrong? We've always had a very active sex life and now it's nonexistent.
A. Unfortunately, decreased sexual desire is common in both the Alzheimer's patient and the caregiver for different reasons. There are other ways for you to connect and maintain intimacy, it will probably not be the same but it could be fulfilling in different ways. There is more information at the following link:
https://www.alz.org/help-support/caregiving/caregiver-health/relationship-changes

Prompt:

How come my spouse is no longer interested in being intimate with me? My spouse has Alzheimer's:
decreased sexual desire is common in both the Alzheimer's patient and the
Caregiver
information about other ways for you to connect and maintain intimacy:
https://www.alz.org/help-support/caregiving/caregiver-health/relationship-changes

Q6. I am so angry that my spouse is like this! Why did this have to happen? I try not to be angry, but I just feel so helpless and so angry. Please help.
A. Anger is a common stage in the grief process. You are mourning the loss of who your spouse was and are experiencing anger over what you are losing. You are not alone. Others have felt this way.
There is more information here:
https://www.alz.org/help-support/caregiving/caregiver-health/grief-loss-as-alzheimers-progresses
but you could also look into the Alzheimer's support
groups in your area https://www.alz.org or even a grief support group in your area.

Prompt:

What should I do if I am angry about my spouse's behavior because of Alzheimer's disease?
Anger is a common stage in the grief process.
You are mourning the loss of who your spouse was and are experiencing anger over what you are losing.
You are not alone. Others have felt this way.
more information here:
https://www.alz.org/help-support/caregiving/caregiver-health/grief-loss-as-alzheimers-progresses
you could also look into the Alzheimer's support
groups in your area https://www.alz.org or even a grief support group in your area.

Q7. I've just been diagnosed with high blood pressure and my doctor is telling me I need to reduce my stress, how do I do that as a caregiver?
A. Yes, it can be hard to reduce stress as a caregiver. There are some resources online, you can read more here about caregiver stress: https://www.alz.org/help-support/caregiving/caregiver-health/caregiver-stress and you find resources to help your stress here: https://www.alz.org/help-support/caregiving/caregiver-health/caregiver-stress-check

Prompt:

How do I do as a caregiver if I've been diagnosed with high blood pressure and need to reduce my stress? My spouse has Alzheimer's or Dementia.
it can be hard to reduce stress as a caregiver.
some resources online, you can read more here about caregiver stress:
https://www.alz.org/help-support/caregiving/caregiver-health/caregiver-stress
you find resources to help your stress here: https://www.alz.org/help-support/caregiving/caregiver-health/caregiver-stress-check

Q8. I've had several people offer to help me, sit with my spouse so I can get some time alone, run errands for me, and even cook for me, but I'm having trouble keeping track of all of them which is increasing my stress levels. My calendar app on my phone isn't good enough. Is there another app or way I can keep track of things like that and maybe they can see it tto,so they see where the gaps are?
A. This is a great question and one many caregivers have asked. Yes, there are several free online calendars that allow you to share your story and your needs with others, they can volunteer and take slots and others can see what spots have been taken and what spots still need to be filled. You can find more information about your options here:
https://www.alz.org/help-support/caregiving/care-options/care-team-calendar

Prompt:

What should I do if I'm having trouble keeping track of all of the events which is increasing my stress levels? My spouse has Alzheimer's or Dementia.
there are several free online calendars that allow you to share your story and your needs with others, they can volunteer and take slots and others can see what spots have been taken and what spots still need to be filled.
You can find more information about your options here:
https://www.alz.org/help-support/caregiving/care-options/care-team-calendar

Q9. I'm a diabetic and several times I've forgotten my own medication because I'm so focused on my spouse's medication for their Alzheimer's disease and other chronic issues. This has never happened to me before. What are some good ways for me to remember to take my own medication?
A. It might help you to use the same routine for yourself that you are using for your spouse. If you are using a pillbox organizer for your spouse, do the same for yourself – just make sure to LABEL the pillboxes with your names so you don't mix them up. Or you could get different colors for each of you. Fill them one at a time but consecutively – first fill your spouse's and then fill yours or vice versa. Try to coordinate taking your own medication when your spouse is supposed to take their medication that way you'll remember to take yours. You can leave post it notes to remind yourself of your medication or set an alarm on your phone. You can find more information here about medication safety:
https://www.alz.org/help-support/caregiving/safety/medication-safety

Prompt:

What should I do if I'm a diabetic and several times I've forgotten my own medication because I'm so focused on my spouse's medication for their Alzheimer's disease and other chronic issues?
It might help you to use the same routine for yourself that you are using for your spouse.
If you are using a pillbox organizer for your spouse, do the same for yourself – just make sure to LABEL the pillboxes with your names so you don't mix them up.
you could get different colors for each of you.
Fill them one at a time but consecutively – first fill your spouse's and then fill yours or vice versa.
Try to coordinate taking your own medication when your spouse is supposed to take their medication that way you'll remember to take yours.
You can leave post it notes to remind yourself of your medication or set an alarm on your phone.
You can find more information here about medication safety:
https://www.alz.org/help-support/caregiving/safety/medication-safety

Q10. Yesterday my spouse was able to put their own shoes on and today they needed help? Is this normal? I mean, it kind of freaked me out to see such a huge change overnight and I almost had to take the nerve pill my Dr. Prescribed for me but I hate those things because I'm afraid I won't be able to be on top of my spouse if I take them. What can I do?
A. Unfortunately, sometimes people with Alzheimer's can change in their ability to perform a life skill seemingly overnight. It may be time for you to start looking into respite care, just to give yourself a break so you don't burn out. It's a brief time away so you can take some time for yourself and come back refreshed and ready to care. You can find more about being a health caregiver here:
https://www.alz.org/help-support/caregiving/caregiver-health/be_a_healthy_caregiver
more about respite care here: https://www.alz.org/help-support/caregiving/care-options/respite-care and more about other care options here: https://www.alz.org/help-support/caregiving/care-options

Prompt:

What can I do if my spouse can change in their ability to perform a life skill seemingly overnight. My spouse has Alzheimer's.
sometimes people with Alzheimer's can change in their ability to perform a life skill seemingly overnight. It may be time for you to start looking into respite care, just to give yourself a break so you don't burn out.
It's a brief time away so you can take some time for yourself and come back refreshed and ready to care.
You can find more about being a health caregiver here: https://www.alz.org/help-support/caregiving/caregiver-health/be_a_healthy_caregiver
more about respite care here: https://www.alz.org/help-support/caregiving/care-options/respite-care
more about other care options here: https://www.alz.org/help-support/caregiving/care-options

Domain 7 – Caregiver Burden

Q1. I feel like I need a break from taking care of my spouse, my family lives far away, what can I do?
A. Respite care is available in most areas. Respite care is a temporary break from caregiving and can be as limited as letting you go out to lunch, or taking a few needed days for yourself to rest and rejuvenate. You can start exploring respite care here: https://www.alz.org/help-support/caregiving/care-options/respite-care The following link will guide you to help in your location: https://www.communityresourcefinder.org/

Prompt:

How could I find a break from taking care of my spouse, who has Alzheimer's,
#Respite care provides caregivers a temporary rest from caregiving.
Respite care is a temporary break from caregiving and can be as limited as letting you go out to lunch, or taking a few needed days for yourself to rest and rejuvenate.
exploring respite care here: https://www.alz.org/help-support/caregiving/care-options/respite-care
guide you to help in your location: https://www.communityresourcefinder.org/
#Alzheimer's Association: 24/7 helpline 800.272.3900
#Services: Alzheimer's Association, AARP, Care at home, Community services, Housing option and Medical Services.
#Care Option: In-Home Care, Adult Day Centers, Long-Term Care.
#Overcoming concern: Cost, Reliability, Guilt.
Center for Dementia Respite Innovation

Q2. My spouse is wandering at night, how can I sleep and keep my spouse safe at the same time?
A. You are not alone, wandering is very common for people with Alzheimer's and dementia, but there are resources to help. Start here first: https://www.alz.org/help-support/caregiving/safety/wandering

Prompt:

How can I sleep and keep my spouse safe? My spouse is wondering at night and diagnosed with Alzheimer's,
wandering is very common for people with Alzheimer's and dementia
resources to help: https://www.alz.org/help-support/caregiving/safety/wandering
Alzheimer's Association: 24/7 helpline 800.272.3900
#Common symptoms: to wander or become lost or confused about their location.
Who's at risk for wandering?
Reduce the risk of wandering: daily plan, engage in structured activities.
#Home safety Checklist
When someone with dementia is missing within 15 minutes, call 911.

Q3. My spouse left the stove on the other day and almost started a fire. How do I manage something like that?
A. Sometimes people with Alzheimer's and dementia forget how to use appliances or forget that they are in use. This is a common concern. Appliances with automatic shut offs are available or knob covers can be removed so burners cannot be used. More information can be found here: https://www.alz.org/help-support/caregiving/safety/home-safety

Prompt:

How do I manage that my spouse left the stove on the other day and almost started a fire? My spouse is diagnosed with Alzheimer's,
Alzheimer's disease causes changes in the brain and body that may affect safety.
people with Alzheimer's and dementia forget how to use appliances or forget that they are in use
information can be found here: https://www.alz.org/help-support/caregiving/safety/home-safety
#Alzheimer's Association: 24/7 helpline 800.272.3900
#How dementia affects safety: judgment, sense of time and place, Behavior, physical ability, sense.
#Home safety Checklist
Home safety tips: safety devices, evaluate environment, avoid safety hazards, prepared for emergencies, install locks out of sight, avoid injury in the bathroom, Secure large furniture, keep walkways empty, placed medication in locked place.

Q4. My spouse started yelling at me for no reason last night and I was afraid my spouse was going to hit me. My spouse has NEVER been abusive in our entire marriage, why is this happening and what can I do?
A. First, understand this is the Alzheimer's or dementia that is acting this way, this is NOT your spouse acting this way on purpose. Try to defuse the situation by remaining calm, decrease stimulation, and assess if there is something they need, a source of frustration, or a problem with communication. There are more resources here to help with behavioral issues: https://www.alz.org/help-support/caregiving/stages-behaviors/agression-anger

Prompt:

My spouse is diagnosed with Alzheimer's or dementia, my spouse started yelling at me for no reason, what do I do to avoid being hit?
the Alzheimer's or dementia causes abusive and aggressive behavior
Try to defuse the situation by remaining calm, decrease stimulation, and assess if there is something they need, a source of frustration, or a problem with communication.
resources here to help with behavioral issues: https://www.alz.org/help-support/caregiving/stages-behaviors/agression-anger
Alzheimer's Association: 24/7 helpline 800.272.3900
#stages and behaviors: Aggression and Anger
Treating Behavioral Symptoms
#Communication Tips: poor communication issue.
#How to respond: Don't get upset, identify immediate cause, focusing on feelings, relaxation, safety
ALZConnected, an online support community and message boards.

Q5. My spouse gets really anxious in crowded places and I now feel like we are trapped in our home, please help me.
A. Unfortunately, anxiety and agitation are common issues for people with Alzheimer's and dementia, oftentimes related to negotiate a new environment or new stimulus – even if it's something they've done before, their memory loss and confusion can make it seem like it's brand new. First, remain calm, ask if you can help, speak positively, remind them they are safe. There are more suggestions and information regarding anxiety and agitation at the following link: https://www.alz.org/help-support/caregiving/stages-behaviors/anxiety-agitation

Prompt:

What should I do if my spouse gets really anxious in crowded places and I now feel like we are trapped in our home? My spouse is diagnosed with Alzheimer's or dementia.
anxiety and agitation are common issues for people with Alzheimer's and dementia, oftentimes related to negotiate a new environment or new stimulus – even if it's something they've done before, their memory loss and confusion can make it seem like it's brand new.
First, remain calm, ask if you can help, speak positively, remind them they are safe.
suggestions and information regarding anxiety and agitation at the following link: https://www.alz.org/help-support/caregiving/stages-behaviors/anxiety-agitation
#Alzheimer's Association: 24/7 helpline 800.272.3900
#stages and behaviors: Anxiety and Agitation
Possible causes: Treating Behavioral Symptoms
Prevent or reduce agitation
Responsibility: what to Do and what to Say
#Association or groups: Brexpiprazole (Rexulti®)

Q6. The doctor told us my spouse shouldn't drive and my spouse is refusing to give up the car keys, how do I get my spouse to understand it's not safe for them to drive anymore?
A. Losing one's independence is always a difficult transition. Some people willingly hand over keys and others adamantly refuse to stop driving. Engaging them in a calm conversation and appealing to their sense of responsibility as a safe driver can often be key to a successful conversation. However, sometimes these conversations do not go well. Hiding the keys or selling the car are sometimes a last ditch effort to prevent driving. The following link has tips for how to handle the conversation and resources to help https://www.alz.org/help-support/caregiving/safety/dementia-driving Additionally, AARP offers driver safety courses online for a reasonable fee https://www.aarp.org/auto/driver-safety/

Prompt:

how do I get my spouse to quit driving? My spouse has Alzheimer's.
Losing one's independence is always a difficult transition.
Some people willingly hand over keys and others adamantly refuse to stop driving.
Engaging them in a calm conversation and appealing to their sense of responsibility as a safe driver can often be key to a successful conversation. However, sometimes these conversations do not go well.
Hiding the keys or selling the car are sometimes a last ditch effort to prevent driving.
The following link has tips for how to handle the conversation and resources to help https://www.alz.org/help-support/caregiving/safety/dementia-driving
AARP offers driver safety courses online for a reasonable fee https://www.aarp.org/auto/driver-safety/
#Alzheimer's Association: 24/7 helpline 800.272.3900
#Dementia and Driving: Transportation Option.
Learn the facts about driving safety
#Driving evaluation: Signs of unsafe driving
#Alzheimer's Association and the National Highway Traffic Safety Administration (NHTSA).

Q7. I feel like the weight of the world is on my shoulders, we used to share all the work together, running the house, cleaning, finances, and now I'm doing it all alone and I'm so tired. But what can I do? I mean, there's nobody to help me. None of our family is local.
A. It sounds like you are overwhelmed and maybe feeling burned out. This is normal when caregiving falls completely to you. There will be changing roles as the disease progresses. You can read more about that here:
https://www.alz.org/help-support/caregiving/caregiver-health/relationship-changes
It sounds like you could benefit from respite care or joining a support group. You can find information about respite care here:
https://www.alz.org/help-support/caregiving/care-options/respite-care and support groups here:
https://www.alz.org/help-support/community/support-groups

Prompt:
Where can I get help if I have to do all the house work and finances by myself? My spouse has Alzheimer's.
You are overwhelmed and maybe feeling burned out. This is normal when caregiving falls completely to you.
There will be changing roles as the disease progresses.
read more about that here:
https://www.alz.org/help-support/caregiving/caregiver-health/relationship-changes
you could benefit from respite care or joining a support group.
find information about respite care here:
https://www.alz.org/help-support/caregiving/care-options/respite-care
find support groups here: https://www.alz.org/help-support/community/support-groups
#Alzheimer's Association: 24/7 helpline 800.272.3900
#Relationship changes: Intimacy, family and Friends, changing roles.
#Financial and Legal Plan, Overcoming stigma.
#Community: ALZConnected®, Virtual support group.
#Caregivers support: Respite Care

Q8. My spouse likes to do little tasks for me around the house, and I'm so happy about that, but I kind of feel guilty about giving my spouse things to do, especially things my spouse didn't do in the past but is now more than happy to do. I feel like I'm taking advantage of my spouse.
A. This is a common feeling among caregivers. You should join the online community and see what other caregivers are saying about this topic. You can find the community here:
https://alzconnected.org/

Prompt:

Is it okay if I ask my spouse to do little tasks for me around the house? My spouse has Alzheimer's.
Feeling guilty is a common feeling among caregivers.
You should join the online community and see what other caregivers are saying about this topic.
You can find the community here: https://alzconnected.org/
#Alzheimer's Association: 24/7 helpline 800.272.3900
#Caregivers Supoprt groups and Community: ALZConnected® an online community
Get Help and Support, Day or Night
#Assistance: over 200 languages through discussion.

Q9. I really wish my family would help out more. They are local, I know they have jobs, but I also know they go out to eat, to the movies and ballgames, and sometimes I wish they would think – hey, maybe we should help Mom/Dad see if she/he needs anything? How can I let them know I need help?
A. It might be helpful for you to invite them over for dinner and have a serious conversation about the burden you are feeling and discuss the help they might be able to provide. There is information online regarding this topic here: https://www.alz.org/help-support/resources/resolving-family-conflicts but it also might help to connect with other spouses online here: https://www.alzconnected.org or call our helpline and talk to a human being who can assist you **800.272.3900.**

Prompt:

How could I ask my family members for help if my spouse has Alzheimer's?
It might be helpful for you to invite them over for dinner and have a serious conversation about the burden you are feeling and discuss the help they might be able to provide.
There is information online regarding this topic here: https://www.alz.org/help-support/resources/resolving-family-conflicts
it also helps to connect with other spouses online here: https://www.alzconnected.org
#Alzheimer's Association: 24/7 helpline 800.272.3900
#Resolving Family Conflicts: Tips for families
#Caregivers Community: ALZConnected®, support group from area.
#Helpline or call (800.272.3900)

Q10. Someone mentioned I should keep a list of all my meds and my spouse's meds on my phone and have an emergency contact list in case I would need them – should one of us end up in the hospital. Are there guidelines for how I would create those items?
A. Yes, having a list of both of your medications is important, you can have the pharmacy print one up for you, your health care provider can print one up for you, or you can type it and print it yourself from you home computer. You can also create a list on your phone in the note app or the health app. There is more information regarding medication safety here: https://www.alz.org/help-support/caregiving/safety/medication-safety An emergency contact list is also a great idea. Again you can create a list in the note app on your phone, you can add emergency contacts in the health app on your phone, or you can designate someone as an emergency contact in the phone app (contacts section) on your phone. If you need help doing this call our helpline and there should be someone that can assist you **800.272.3900.**

Prompt:
Are there guidelines for how I would keep a list of all my meds and my spouse's meds on my phone and have an emergency contact list in case I would need them?
having a list of both of your medications is important, you can have the pharmacy print one up for you, your health care provider can print one up for you, or you can type it and print it yourself from you home computer.
You can also create a list on your phone in the note app or the health app
more information regarding medication safety here: https://www.alz.org/help-support/caregiving/safety/medication-safety
An emergency contact list is also a great idea. Again you can create a list in the note app on your phone, you can add emergency contacts in the health app on your phone, or you can designate someone as an emergency contact in the phone app (contacts section) on your phone.
#Alzheimer's Association: 24/7 helpline 800.272.3900
#Medication Safety: Working with doctor, tips for giving medication.
#Home safety Checklist, emergency contact.
#Caregivers Community: ALZConnected®, support group from area.
#Helpline or call (800.272.3900)

Domain 8 – Caregiving Self-Efficacy

Q1. I'm not sure what to do with my spouse during the day, like what should our day look like? How do I keep my spouse focused, what do I feed my spouse, and how do I handle memory issues?
A. This is a very valid concern. Your day doesn't have to change, routine is good for people with Alzheimer's and dementia. Make a plan for your day, include activities you both enjoy, meals you like to eat, and other activities. You can talk it over with your spouse and see if they have any wants or needs not addressed in the daily plan. For more help with making daily plans and suggestions, follow this link: https://www.alz.org/help-support/caregiving/daily-care/daily-care-plan

Prompt:

How to deal with the following situations as my spouse is diagnosed with Alzheimer's?
keep my spouse focused,
feed my spouse
handle memory issues
maintain routine is good for people with Alzheimer's and dementia.
Make a plan for your day, include activities you both enjoy, meals you like to eat, and other activities.
talk it over with your spouse and see if they have any wants or needs not addressed in the daily plan
help with making daily plans and suggestions, follow this link: https://www.alz.org/help-support/caregiving/daily-care/daily-care-plan

Q2. I'm worried about making sure my spouse gets all their medications on time and that they don't accidentally take too many because they forgot they already took them. What do I do about that?
A. Many caregivers use a pill box organizer for themselves and for their spouse. These come with daily sections or twice daily sections, that way you can be sure medicines have been taken. As for ensuring they take too many, you will have to take control of filling the pill box and keeping it out of sight and in a safe spot away from your spouse. There is much more information on medications here: https://www.alz.org/alzheimers-dementia/treatments/medications-for-memory and medication safety here: https://www.alz.org/media/Documents/alzheimers-dementia-medication-**safety-ts.pdf**

Prompt:

What do I do to make sure that my spouse gets all their medications on time and they don't accidentally take too many because they forgot they already took them? My spouse has Alzheimer's:
caregivers use a pill box organizer for themselves and for their spouse
daily sections or twice daily sections that way you can be sure medicines have been taken
take control of filling the pill box and keeping it out of sight and in a safe spot away from your spouse
information on medications here: https://www.alz.org/alzheimers-dementia/treatments/medications-for-memory
medication safety here: https://www.alz.org/media/Documents/alzheimers-dementia-medication-**safety-ts.pdf**

Q3. What kind of activities should we do? I mean, my spouse isn't a child, I'm not going to bring out coloring books and crayons, I just don't know what kind of activities to plan.
A. The best thing initially is to keep doing the activities you loved. As the disease progresses, your spouse may lose interest in those activities and you will need to adjust for that, keeping in mind their skills and interests. Again, routines are good, you can leave notes for them to help out with small tasks like setting the table or emptying the dishwasher. These are just a few suggestions, there is more information here: https://www.alz.org/help-support/caregiving/daily-care/activities .

Prompt:

what kind of activities to plan if my spouse is diagnosed with Alzheimer's ?
The best thing initially is to keep doing the activities you loved
as disease progresses, your spouse may lose interest in those activities and you will need to adjust for that
keeping in mind their skills and interests
routines are good, you can leave notes for them to help out with small tasks like setting the table or emptying the dishwasher.
suggestions information here: https://www.alz.org/help-support/caregiving/daily-care/activities .

Q4. I'm afraid I'm not very good at helping my spouse in the bathroom, like showering, using the toilet, brushing their teeth, getting dressed, how do I know how much to help and when to let them do things on their own? And what if my spouse has an accident?
A. Activities of daily living or ADLS can be a source of significant concern for caregivers, bathing, dressing, grooming, dental hygiene, are all activities that your spouse may need help with eventually. Talk to your spouse early on about their preferences and coming up with a plan for all activities is a good way to start. You can find more information on bathing here: https://www.alz.org/help-support/caregiving/daily-care/bathing , dressing & grooming here: https://www.alz.org/help-support/caregiving/daily-care/dressing-grooming , and dental care here: https://www.alz.org/help-support/caregiving/daily-care/dental-care . If your spouse has trouble with incontinence there are products available in grocery stores such as Depends that will help, other information can be found here: https://www.alz.org/help-support/caregiving/daily-care/incontinence .

Prompt:

How to take care of my spouse in the bathroom? My spouse has Alzheimer's.
showering
using the toilet
brushing their teeth
getting dressed
how do I know how much to help and when to let them do things on their own?
what to do if my spouse has an accident?
Activities of daily living or ADLS can be a source of significant concern for caregivers, bathing, dressing, grooming, dental hygiene, are all activities that your spouse may need help with eventually
Talk to your spouse early on about their preferences and coming up with a plan for all activities is a good way to start.
information on bathing here: https://www.alz.org/help-support/caregiving/daily-care/bathing
dressing & grooming here: https://www.alz.org/help-support/caregiving/daily-care/dressing-grooming
dental care here: https://www.alz.org/help-support/caregiving/daily-care/dental-care
If your spouse has trouble with incontinence there are products available in grocery stores such as Depends that will help, information here: https://www.alz.org/help-support/caregiving/daily-care/incontinence .

Q5. I don't know what I should do about meals? Does having Alzheimer's mean I'll have to cook differently? I'm a pretty good cook, but not if I have to make fancy meals.
A. Generally, you shouldn't have to change your diet. A heart healthy diet low in cholesterol, saturated fat, and sodium, and high in lean protein, fruits, and vegetables is always recommended. As the disease progresses, your spouse's appetite might decrease and chewing and swallowing might become problematic at which time you might have to switch to softer foods and cutting food up into bite size pieces. There is a lot of information at the following link regarding dietary suggestions and needs https://www.alz.org/help-support/caregiving/daily-care/food-eating .

Prompt:

Do I have to cook differently if my spouse has Alzheimer's?
you shouldn't have to change your diet.
A heart healthy diet low in cholesterol, saturated fat, and sodium, and high in lean protein, fruits, and vegetables is always recommended.
As the disease progresses, your spouse's appetite might decrease and chewing and swallowing might become problematic at which time you might have to switch to softer foods and cutting food up into bite size pieces.
information at the following link regarding dietary suggestions and needs https://www.alz.org/help-support/caregiving/daily-care/food-eating .

Q6. My spouse always handled the finances, I don't even know where our bank accounts are or if we have a checking account? I think most of our stuff was online. What should I do?
A. Unfortunately, this situation is quite common, where one spouse is in charge of the finances and the other is not. If your spouse has just been diagnosed and is still in the early stages, the two of you should sit down and have an honest discussion regarding your finances and you should slowly start taking over the finances. There are resources available for you regarding planning and paying for care, insurance, social security and disability, Medicare, Medicare Part D, Medicaid, tax deductions and credits, as well as legal advice. You can find many helpful tips and links here: https://www.alz.org/help-support/caregiving/financial-legal-planning

Prompt:

My spouse has Alzheimer's, how do I handle the finances?
quite common, where one spouse is in charge of the finances and the other is not.
in the early stages, the two of you should sit down and have an honest discussion regarding your finances and you should slowly start taking over the finances.
find resources available for you regarding planning and paying for care, insurance, social security and disability, Medicare, Medicare Part D, Medicaid, tax deductions and credits, as well as legal advice
helpful tips and links here: https://www.alz.org/help-support/caregiving/financial-legal-planning

Q7. Is there legal stuff I need to be worried about now that my spouse has Alzheimer's? My spouse handled all of our bills, insurance, car payments, taxes, and now I don't even know where to begin. What should I do?
A. If your spouse is still in the early stages, you and your spouse can start working on those tasks together. You can slowly take over once you feel more comfortable taking care of things. For more information on legal and financial matters follow this link here: https://www.alz.org/help-support/caregiving/financial-legal-planning

Prompt:
My spouse has Alzheimer's, My spouse handled all of our bills, insurance, car payments, taxes, and now I don't even know where to begin. What should I do?
in the early stages, you and your spouse can start working on those tasks together.
slowly take over once you feel more comfortable taking care of things.
information on legal and financial matters follow this link here:
https://www.alz.org/help-support/caregiving/financial-legal-planning

Q8. I have no idea how to help my spouse in the toilet. I've never done anything like that. It feels very unsanitary. Is there a video I can watch or some sort of online education about this? What should I do?
A. If your spouse is having trouble controlling their bladder or their bowels, the first step is to talk to your health care provider and find out if there is an underlying issue that can be addressed, such as an infection or an impaction. Always treat your spouse with respect, especially if the cause turns out to be beyond their control. Don't scold or embarrass them and try to maintain their privacy and dignity. You can read more information here: https://www.alz.org/help-support/caregiving/daily-care/incontinence or you can call the Helpline and talk to someone anytime day or nigh**t 800.272.3900.** you can find community resources close to you here: https://www.communityresourcefinder.org/ or you can click this link for resources specific to the National Capital Area (NCA) https://www.alz.org/nca

Prompt:
My spouse has Alzheimer's, how to help my spouse in the toilet?
a video you can watch
online education
If your spouse is having trouble controlling their bladder or their bowels, the first step is to talk to your health care provider and find out if there is an underlying issue that can be addressed, such as an infection or an impaction.
Always treat your spouse with respect, especially if the cause turns out to be beyond their control.
Don't scold or embarrass them and try to maintain their privacy and dignity.
read more information here: https://www.alz.org/help-support/caregiving/daily-care/incontinence
call the Helpline and talk to someone anytime day or night 800.272.3900.
find community resources close to you here: https://www.communityresourcefinder.org/
click this link for resources specific to the National Capital Area (NCA) https://www.alz.org/nca

Q9. How do I care for my spouse's teeth? Do I need to floss and brush for my spouse? Is that something they can do on their own?
A. Most of the time in the early stages, that is a task your spouse can perform on their own, maybe with some gentle coaching. They might eventually progress to a point where you will need to do it for them. You can read more about dental hygiene and care here: https://www.alz.org/help-support/caregiving/daily-care/dental-care

Prompt:

My spouse has Alzheimer's, how do I care for my spouse's teeth?
in the early stages, your spouse can floss and brush without help
maybe with some gentle coaching
eventually progress to a point where you will need to floss and brush for them
read more about dental hygiene and care here: https://www.alz.org/help-support/caregiving/daily-care/dental-care

Q10. Communication was never our strong suit, it's even worse now. Sometimes my spouse just sits and stares off into space. I feel so helpless, how can we communicate if one of us isn't interested?
A. Communicating with a spouse with Alzheimer's can be challenging. Don't exclude your spouse from conversations try to involve them, ask their opinion of things, and remember the disease affects each person differently. You can read more about communication here: https://www.alz.org/help-support/caregiving/daily-care/communications but you can also call the Helpline **800.272.3900** and talk to a human or speak with your health care provider.

Prompt:

My spouse has Alzheimer's, how to maintain smooth communication?
Communicating with a spouse with Alzheimer's can be challenging.
Don't exclude your spouse from conversations try to involve them, ask their opinion of things, and remember the disease affects each person differently.
read more about communication here: https://www.alz.org/help-support/caregiving/daily-care/communications
call the Helpline 800.272.3900 and talk to a human or speak with your health care provider.

Domain 9 – Behavioral Problems in PWD

Q1. I've heard people with Alzheimer's can get violent? Is that true? What do I do?
A. It is true that some people with more advanced Alzheimer's or dementia can have episodes of anger and aggression, however, not everyone has this behavioral issue as a symptom. You should discuss this possibility with your physician and come up with a plan to handle it should it occur. Some suggestions are evaluate why the person might be acting this way – such as are they in physical discomfort? Are they being overstimulated with things such as loud noises, crowds, or unfamiliar people? Are you asking them too many questions without giving them time to respond? These are just some initial ideas to think about as you try to descalate the situation. You can find more information about this topic here: https://www.alz.org/help-support/caregiving/stages-behaviors/agression-anger you can find community resources close to you here: https://www.communityresourcefinder.org/ you can also call the Alzheimer's Association's hotline and talk to an actual person that can help you **800.272.3900.** Or you can click this link for resources specific to the National Capital Area (NCA) https://www.alz.org/nca

Prompt:

How to deal with people with Alzheimer's if they get violent?
It is true that some people with more advanced Alzheimer's or dementia can have episodes of anger and aggression
not everyone has this behavioral issue as a symptom
discuss this possibility with your physician and come up with a plan to handle it should it occur.
suggestions are evaluate why the person might be acting this way – such as are they in physical discomfort? Are they being overstimulated with things such as loud noises, crowds, or unfamiliar people? Are you asking them too many questions without giving them time to respond? These are just some initial ideas to think about as you try to descalate the situation
find more information about this topic here: https://www.alz.org/help-support/caregiving/stages-behaviors/agression-anger
find community resources close to you here: https://www.communityresourcefinder.org/
call the Alzheimer's Association's hotline and talk to an actual person that can help you 800.272.3900.
click this link for resources specific to the National Capital Area (NCA) https://www.alz.org/nca

Q2. I think my spouse just had a hallucination, is that a thing? Can someone with Alzheimer's get hallucinations? How are they treated? What do I do?
A. Yes, it is possible for people with Alzheimer's to experience hallucinations, they may hear, see, smell, taste, or even feel something that isn't there. The important thing is to remain calm and understand this is temporary and the hallucination will end. If your spouse becomes anxious or nervous, calmly tell them you're with them and there is nothing to be afraid of, do not argue with your spouse about what they may be experiencing. Gently pat your spouse's shoulder or back – this type of physical interaction might interrupt the hallucination and bring it to an end. You should discuss this possibility with your physician and come up with a plan, and if your spouse experiences a hallucination, you should definitely let your physician know. There is more information at the following link: https://www.alz.org/help-support/caregiving/stages-behaviors/hallucinations. You can find community resources close to you here: https://www.communityresourcefinder.org/ you can also call the Alzheimer's Association's hotline and talk to an actual person that can help you **800.272.3900.** Or you can click this link for resources specific to the National Capital Area (NCA) https://www.alz.org/nca

Prompt:

How to deal with the Alzheimer's with hallucination?
it is possible for people with Alzheimer's to experience hallucinations, they may hear, see, smell, taste, or even feel something that isn't there
to remain calm and understand this is temporary and the hallucination will end.
If your spouse becomes anxious or nervous, calmly tell them you're with them and there is nothing to be afraid of, do not argue with your spouse about what they may be experiencing.
Gently pat your spouse's shoulder or back – this type of physical interaction might interrupt the hallucination and bring it to an end.
discuss this possibility with your physician and come up with a plan, and if your spouse experiences a hallucination, you should definitely let your physician know
find community resources close to you here: https://www.communityresourcefinder.org/
call the Alzheimer's Association's hotline and talk to an actual person that can help you 800.272.3900
click this link for resources specific to the National Capital Area (NCA) https://www.alz.org/nca

Q3. My spouse wandered off last night, Thank God I was able to find him/her! My spouse was only gone for a few minutes – walked right out the front door while I was using the bathroom. How do I keep that from happening in the future? I'm so scared he/she will get lost, kidnapped, hit by car, so many things can happen. I'm so worried.
A. Unfortunately, wandering is a common problem and understandably it is scary. Discuss the event with your doctor, but in the meantime, make sure your spouse is engaged in daily activities, keeping him/her busy doing things he/she enjoys. Make sure all the basic needs are met, toileting, grooming, and/or hunger. Consider removing car keys to a different location. In the early stages simply explaining to your spouse that you are going to use the bathroom and asking them to wait until you return may alleviate an accidental wander. As the disease progresses you may need to consider adding additional deadbolts that are out of sight (high or low on the door), have door alarms installed that go off when opened, and create a daily plan. There is more information about wandering and tips for preventing it and what to do if it happens here: https://www.alz.org/help-support/caregiving/stages-behaviors/wandering. You can find community resources close to you here: https://www.communityresourcefinder.org/ you can also call the Alzheimer's Association's hotline and talk to an actual person that can help you **800.272.3900.** Or you can click this link for resources specific to the National Capital Area (NCA) https://www.alz.org/nca

Prompt:

How do I keep my spouse from wandering off? My spouse has Alzheimer's.
wandering is a common problem and understandably it is scary
Discuss the event with your doctor, but in the meantime, make sure your spouse is engaged in daily activities, keeping him/her busy doing things he/she enjoys.
#Make sure all the basic needs are met, toileting, grooming, and/or hunger.
#Consider removing car keys to a different location.
#In the early stages simply explaining to your spouse that you are going to use the bathroom and asking them to wait until you return may alleviate an accidental wander.
#As the disease progresses you may need to consider adding additional deadbolts that are out of sight (high or low on the door), have door alarms installed that go off when opened, and create a daily plan.
information about wandering and tips for preventing it and what to do if it happens here: https://www.alz.org/help-support/caregiving/stages-behaviors/wandering.
find community resources close to you here: https://www.communityresourcefinder.org/
call the Alzheimer's Association's hotline and talk to an actual person that can help you 800.272.3900
click this link for resources specific to the National Capital Area (NCA) https://www.alz.org/nca

Q4. My spouse is having trouble sleeping and is confused at night...is this part of Alzheimer's and what can I do about it? I feel helpless.
A. Unfortunately, night time confusion can be a part of Alzheimer's. Your spouse may be mentally and physically exhausted from their day which can lead to confusion in the evening. Older adults require less sleep overall, so that may be a contributing factor as well. There is more information about this phenomenon here: https://www.alz.org/help-support/caregiving/stages-behaviors/sleep-issues-sundowning. You can find community resources close to you here: https://www.communityresourcefinder.org/ you can also call the Alzheimer's Association's hotline and talk to an actual person that can help you **800.272.3900.** Or you can click this link for resources specific to the National Capital Area (NCA) https://www.alz.org/nca

Prompt:
what can I do if my spouse is having trouble sleeping and is confused at night? My spouse has Alzheimer's.
night time confusion can be a part of Alzheimer's.
Your spouse may be mentally and physically exhausted from their day which can lead to confusion in the evening.
Older adults require less sleep overall, so that may be a contributing factor as well.
information about this phenomenon here: https://www.alz.org/help-support/caregiving/stages-behaviors/sleep-issues-sundowning
call the Alzheimer's Association's hotline and talk to an actual person that can help you 800.272.3900
click this link for resources specific to the National Capital Area (NCA) https://www.alz.org/nca

Q5. My spouse just accused me of stealing his/her watch and phone! After a few minutes of searching I found both, but he/she is still grumbling about me taking them and hiding them. Is this normal? What should I do? I don't even know how to handle this type of thing.
A. Unfortunately, suspicions and delusions can be a part of Alzheimer's. They are more often found in middle to late-stage Alzheimer's, so if your spouse is experiencing this, their Alzheimer's may have progressed past the early stage. Do not be offended, it is the disease talking and not your spouse. Listen to your spouse, let them talk, and try to give calm and reassuring answers. Keep your answers simple and straight forward, do not try to argue. You can read more information at this linK: https://www.alz.org/help-support/caregiving/stages-behaviors/suspicions-delusions
Or you can connect with others and see what others have to say on this topic here: https://www.alzconnected.org

Prompt:

What should I do if my spouse becomes suspicions and delusions? My spouse has Alzheimer's.
suspicions and delusions can be a part of Alzheimer's
They are more often found in middle to late-stage Alzheimer's, so if your spouse is experiencing this, their Alzheimer's may have progressed past the early stage.
Do not be offended, it is the disease talking and not your spouse.
Listen to your spouse, let them talk, and try to give calm and reassuring answers.
Keep your answers simple and straight forward, do not try to argue.
read more information at this linK: https://www.alz.org/help-support/caregiving/stages-behaviors/suspicions-delusions
connect with others and see what others have to say on this topic here: https://www.alzconnected.org

Q6. My spouse keeps repeating what I say and sometimes what he/she says over and over again. It's driving me crazy! Is this normal? How long will this go on and how do I deal with it?
A. Unfortunately, repetition can be a part of Alzheimer's due to the deterioration of brain cells that occurs with the disease. If repetition happens, try to assess the cause – is your spouse trying to communicate something, does it happen in the same location, situation, around certain people? Always remain calm, if it's a physical thing such as rubbing their hand over a table, offer them a cloth & ask them to dust with it. If it's repeating a question, give them a simple answer even if you have to repeat it numerous times. There are many other tips and options for handling this at the following link: https://www.alz.org/help-support/caregiving/stages-behaviors/repetition

Prompt:
What should I do if my spouse keeps repeating what I say and sometimes what he/she says over and over again? My spouse has Alzheimer's.
#repetition can be a part of Alzheimer's due to the deterioration of brain cells that occurs with the disease.
#If repetition happens, try to assess the cause – is your spouse trying to communicate something, does it happen in the same location, situation, around certain people?
#Always remain calm, if it's a physical thing such as rubbing their hand over a table, offer them a cloth & ask them to dust with it.
#If it's repeating a question, give them a simple answer even if you have to repeat it numerous times
other tips and options for handling this at the following link: https://www.alz.org/help-support/caregiving/stages-behaviors/repetition

Q7. My spouse is very depressed, has been ever since the Alzheimer's diagnosis, which I understand, but it's been months. How long is this supposed to last? And what can I do about it?
A. Unfortunately, depression is not uncommon in people with Alzheimer's or dementia. There are treatments for depression available, especially if your spouse is displaying symptoms such as lack of interest in normal activities, isolation, or impaired thinking. You can find more information about depression and possible treatments here: https://www.alz.org/help-support/caregiving/stages-behaviors/depression the next step is to talk to your spouse's health care provider.

Prompt:

What should I do if my spouse has been depressed for months ever since the Alzheimer's diagnosis?
depression is not uncommon in people with Alzheimer's or dementia.
There are treatments for depression available, especially if your spouse is displaying symptoms such as lack of interest in normal activities, isolation, or impaired thinking.
find more information about depression and possible treatments here: https://www.alz.org/help-support/caregiving/stages-behaviors/depression
the next step is to talk to your spouse's health care provider.

Q8. My spouse is experiencing bad anxiety and agitation anytime we leave the house. I can't stay holed up here forever. How can I convince my spouse it's okay to leave the house to go out to dinner or shopping, or visit friends and family?
A. Sadly, anxiety and agitation are both possible symptoms in the early stages of Alzheimer's. This can be caused by many factors, including medications. Additionally, your spouse may be having trouble processing their environment or changes to their environment which can lead to confusion, anxiety, and agitation. You can read more about this here: https://www.alz.org/help-support/caregiving/stages-behaviors/anxiety-agitation In terms of how to handle it, remain calm, provide reassurance, and as always talk with the health care provider to rule out other reasons. You can find community resources close to you here: https://www.communityresourcefinder.org/ you can also call the Alzheimer's Association's hotline and talk to an actual person that can help you **800.272.3900.** Or you can click this link for resources specific to the National Capital Area (NCA) https://www.alz.org/nca

Prompt:

What should I do if my spouse is experiencing bad anxiety and agitation anytime we leave the house. My spouse has Alzheimer's.
anxiety and agitation are both possible symptoms in the early stages of Alzheimer's.
This can be caused by many factors, including medications.
your spouse may be having trouble processing their environment or changes to their environment which can lead to confusion, anxiety, and agitation.
read more about this here: https://www.alz.org/help-support/caregiving/stages-behaviors/anxiety-agitation
In terms of how to handle it, remain calm, provide reassurance, and as always talk with the health care provider to rule out other reasons.
find community resources close to you here: https://www.communityresourcefinder.org/
call the Alzheimer's Association's hotline and talk to an actual person that can help you **800.272.3900.**
click this link for resources specific to the National Capital Area (NCA) https://www.alz.org/nca

Q9. My spouse makes inappropriate comments in public sometimes. It's like my spouse forgot how to be polite in public spaces. It's so embarrassing, I feel like everyone is looking at me and wondering why we are out in public. Help me.
A. While there is not a specific name for this issue, it is not uncommon and does coincide with middle stage Alzheimer's disease. If your health care provider has not discussed the potential for physical or verbal outbursts with you, you can read more about them here under the Changes in Behavior heading: https://www.alz.org/help-support/caregiving/stages-behaviors/middle-stage And next time you see your health care provider ask them about it. You can find community resources close to you here: https://www.communityresourcefinder.org/ you can also call the Alzheimer's Association's hotline and talk to an actual person that can help you **800.272.3900.** Or you can click this link for resources specific to the National Capital Area (NCA) https://www.alz.org/nca

Prompt:

What should I do if my spouse forgot how to be polite in public spaces. My spouse has Alzheimer's.
While there is not a specific name for this issue, it is not uncommon and does coincide with middle stage Alzheimer's disease.
If your health care provider has not discussed the potential for physical or verbal outbursts with you, you can read more about them here under the Changes in Behavior heading: https://www.alz.org/help-support/caregiving/stages-behaviors/middle-stage
you see your health care provider ask them about it.
find community resources close to you here: https://www.communityresourcefinder.org/
call the Alzheimer's Association's hotline and talk to an actual person that can help you 800.272.3900.
click this link for resources specific to the National Capital Area (NCA) https://www.alz.org/nca

Q10. Someone suggested I give my spouse little tasks to do around the house to keep them occupied and make them feel useful and keep them from becoming agitated or angry – is that a good idea?
A. Yes, it is an excellent idea, especially if it was something your spouse did before their diagnosis. Whatever you can do to maintain their routine and make them feel useful will help them stay active and engaged. You can find more information about activities and daily plans at the following link: https://www.alz.org/help-support/caregiving/daily-care/activities

Prompt:

How could I keep my spouse occupied and make my spouse feel useful and keep my spouse from becoming agitated or angry? My spouse has Alzheimer's.
it is an excellent idea to give your spouse little tasks to do around the house if it was something your spouse did before their diagnosis.
Whatever you can do to maintain their routine and make them feel useful will help them stay active and engaged
find more information about activities and daily plans at the following link: https://www.alz.org/help-support/caregiving/daily-care/activities
