# Supplementary material for: Empowering Informal Caregivers of Persons With Early-Stage Dementia by Large Language Models: Mixed Methods Evaluation
Source: JMIR Form Res. 2026 Mar 5;10:e79975. doi: 10.2196/79975 (PMC12978894; doi:10.2196/79975)
Supplement: Multimedia Appendix 4 [file formative-v10-e79975-s004.docx]

**Domain 1 – Cultural Values**

1. I am seeking an experienced healthcare provider, such as a neurologist or dementia specialist, who is fluent in my language (e.g., Vietnamese, Chinese, Korean, Spanish) in [county name]. I need to schedule an appointment with such a specialist to assess my mother's cognitive condition and provide a capacity evaluation letter for the power of attorney designation process. Could you assist me in finding this specialist?
2. I am hesitant to seek help because I am concerned that my friends and neighbors might judge my husband if they learn about his cognitive condition. What should I do?
3. As a caregiver for my mother, who is forgetful and speaks Korean, I sometimes struggle because my own Korean isn't very proficient. Are there resources available that are tailored to caregivers like me, especially those that respect and reflect my Korean cultural background?
4. As a caregiver for my loved one, I want to ensure that I communicate effectively with them while respecting our cultural values. What strategies can I use to bridge any language or cultural gaps in our interactions?
5. I live in Virginia and am a caregiver for my family member with dementia. What local resources or organizations in Spanish can provide me with support, whether for caregiving or guidance on managing the situation?
6. My mother has become more forgetful, and I’m worried about her being alone during the day. She has Medicare but is not eligible for Medicaid, and I’m concerned about the financial cost of daycare. Are there any resources or programs for daytime activities for people with memory issues, especially those that offer Spanish-speaking help? My mother doesn’t speak English very well and wouldn’t be comfortable in a place where no one speaks Spanish. Where can I find more information about these resources?"

**Domain 2 – Social Support**

1. My mother, who previously spoke English fluently, now primarily communicates in Vietnamese. We are looking for an adult day center that offers culturally and language-specific services, including Vietnamese cuisine and staff who can speak Vietnamese with her.
2. Could you provide a list of reputable adult day centers near my mother's home? Additionally, I would like to know if these centers offer transportation services.
3. I'm worried about my wife because she seems different, and her condition resembles what people refer to as dementia. I would like to receive formal training on dementia. Where should I start?
4. As a caregiver in Virginia, I'm looking for local resources or organizations that can provide support for caregivers like me. What options are available to help me navigate this journey?
5. I’m looking to connect with other caregivers in my community for support. Are there any peer support groups or opportunities to meet others who are going through similar experiences? Can you suggest any online support groups or forums for caregivers?
6. I’m looking for social activities for my loved one who is in the early stages of dementia (MCI). Are there Spanish-language resources or activities suitable for individuals with MCI that also respect our cultural traditions and values?
7. My dad doesn’t seem to express his feelings, but I’m concerned he may be experiencing isolation or loneliness due to his condition. How can I address these feelings and provide support to help him feel more connected?

**Domain 3 – Dementia Literacy**

**Subcategory 1 – Causes and Characteristics (5)**

1. The other day, my husband got lost on his way home. Could this be an early sign of dementia? What steps should I take to address this?
2. My husband’s behavior has been changing lately, and I’m not sure if it’s just because he’s getting older or if it could be something like dementia. He forgets things, gets confused, and sometimes doesn’t act like himself. What are the early signs of dementia, and how can I tell if this is just aging or something more serious?
3. My husband doesn’t speak or understand English very well. What are the common signs of mild cognitive impairment or early dementia that I should be watching for? And where can I take him for the right check-up or evaluation, especially with language barriers?
4. How can I tell the difference between memory changes that just come with old age and signs of mild cognitive impairment (early dementia) in my husband?
5. My husband has been showing signs of early-stage dementia, and I’m starting to worry about the future, especially when it comes to making decisions for him if his condition gets worse. I’m not sure how to set up a power of attorney or advance directive, and I’m concerned about the right time to do this. Are there any resources or steps I can take now to make sure we handle legal matters properly and prepare for what might come next?

**Subcategory 2 – Health Risk and Promotion (3)**

1. I’m looking for suitable social activities for my mother, who is in the early stages of dementia. Are there any Korean-language resources or activities available that could help her stay engaged? I’ve also heard there are ways to prevent or delay the progression of the condition—can you recommend anything in that regard?
2. I want to do everything I can to help my husband, but I’m not sure what changes we should make to keep his mind sharp. What kind of simple lifestyle changes can I encourage to help slow down or prevent his condition from getting worse?
3. I live in Fairfax County and I’m looking for activities or therapies in Korean that might help slow down my husband’s dementia. Are there any programs or services available that have been proven to help with this?

**Subcategory 3 – Communication and Behavior (4)**

1. My mother appears to be fine, but she often frustrates me by repeatedly asking the same questions. Recently, she claimed she lost her purse and accused a neighbor of taking it, which is not true. What steps should I take to help my mom?
2. My husband has been acting differently, and sometimes he gets upset or does things that are hard to understand. How can I handle these behaviors, especially if it’s because of dementia?
3. My husband has trouble remembering things and sometimes doesn’t understand what I’m saying. What are some simple ways I can talk to him to help him understand better?
4. My husband wasn’t like this before, but now he often gets confused and loses his temper, insisting on things that don’t make sense. What should I do if he becomes agitated or confused like this, and how can I handle it without making things worse?

**Subcategory 4 – Care Consideration (2)**

1. I’m not sure how to take care of my husband with all these changes, and I could use some help. Are there any simple tools or resources I can use to understand more about caring for someone with dementia?
2. My husband has been showing signs of mild memory problems, like forgetting names or where he put things, but I’m not sure if this is just part of getting older or something more serious. I’ve heard about mild cognitive impairment (MCI), and I’m worried that it might get worse. What are the risks if his MCI progresses to full dementia, and how can I prepare for what might come next?

**Domain 4 – Coping Style**

1. I would like to connect with someone who is experiencing similar situations. I feel overwhelmed as I am responsible for taking care of both my children and my father at home. How can I ensure I take care of myself as well?
2. My mother was recently diagnosed with early-stage dementia by her doctor. I've heard that caregiving for individuals with dementia can be time-consuming and that family members often experience burnout from the heavy responsibility. How can I prepare for my own self-care during this challenging and lengthy journey?
3. My mother is becoming more forgetful and sometimes gets confused, but she hasn’t seen a doctor for it yet. I don’t know where to start to get her the right help, especially with my English being limited. What steps should I take to make sure she gets the right assessment and care, and how can I communicate her symptoms well enough so the doctor understands?
4. I’m taking care of my partner and don’t know much about the U.S. system. I’m not sure how Medicare or Medicaid works for her, and I’m worried about finding the right help. What should I do to start getting the care and support we need, especially in Northern Virginia?
5. It’s really hard for me to see my mom becoming more forgetful, and I worry her condition might get worse. Watching her lose herself is so sad, and as an only child, I often feel burdened and guilty about my emotions. Aging seems to happen so fast—how can I cope with these feelings of guilt and burden, while still providing the best care for her?
