# Supplementary figures and images for: Empowering Informal Caregivers of Persons With Early-Stage Dementia by Large Language Models: Mixed Methods Evaluation
Source: JMIR Form Res. 2026 Mar 5;10:e79975. doi: 10.2196/79975 (PMC12978894; doi:10.2196/79975)

**Figure S1. Interface for test question no.18 with two types of responses.**

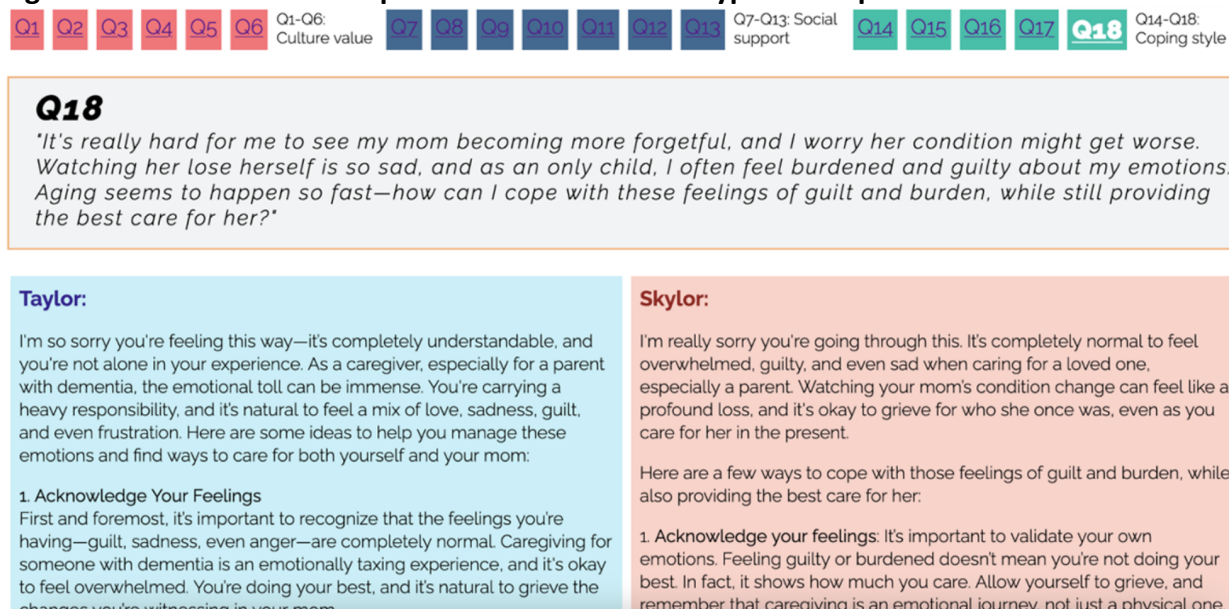

Supplement: Multimedia Appendix 5 [file formative-v10-e79975-s005.pdf]
