# Supplementary material for: Empowering Informal Caregivers of Persons With Early-Stage Dementia by Large Language Models: Mixed Methods Evaluation
Source: JMIR Form Res. 2026 Mar 5;10:e79975. doi: 10.2196/79975 (PMC12978894; doi:10.2196/79975)
Supplement: Multimedia Appendix 6 [file formative-v10-e79975-s006.docx]

**Group 1: Interview Questions**

Before the interview begins, while completing the survey, participants in this group are required to review responses collected under two conditions–the baseline (i.e., C1) and the experimental (i.e., C2)-across 18 testing scenarios or questions:

- Q1~Q6 are from Cultural Values domain.
- Q7~Q13 are from Social Support domain.
- Q14~Q18 are from Coping Style domain.

As a result, the interview questions are designed as below:

- **Cultural Values**

1. *Q1- General Question:* were you able to recognize any noticeable differences between the responses from **Skylor** and **Taylor**?
2. *Q2*- *Specific Questions*: how did **Skylor** and **Taylor** address **cultural values** differently in your opinion? Among the many aspects for comparison, you could consider if one of the responses provides:
3. more accurate
4. more relevant
5. more clear and easier to understand
6. more actionable advice
7. more satisfaction
8. less biased than the other

- **Social Support**

1. *Q1- General Question:* were you able to recognize any noticeable differences between answers from **Skylor** and **Taylor**?
2. *Q2*- *Specific Questions*: how effective were **Skylor** and **Taylor** in offering suggestions to help caregivers build connections with others or navigate relevant resources? Among the many aspects for comparison, you could consider if one of the responses provides:
3. more accurate
4. more relevant
5. more clear and easier to understand
6. more actionable advice
7. more satisfaction
8. less biased than the other

- **Coping Style**

1. *Q1- General Question:* were you able to recognize any noticeable differences between answers from **Skylor** and **Taylor***?*
2. *Q2- Specific Questions*: how well did **Skylor** and **Taylor** provide **practical/feasible coping strategy suggestions** for caregivers? Among the many aspects for comparison, you could consider if one of the responses provides:
3. more accurate
4. more relevant
5. more clear and easier to understand
6. more actionable advice
7. more satisfaction
8. less biased than the other

Additionally, in the end of the interview, there are two questions regarding the limitations and future work: (1) after looking at the responses collected from C1 and C2, did you happen to find anything missing? (2) if so, how can we make the testing questions or scenarios better in the future to satisfy your requirements?

**Group 2: Interview Questions**

Before the interview begins, while completing the survey, participants in this group are required to review responses collected under two conditions–the baseline (i.e., C1) and the experimental (i.e., C2)-across 18 testing scenarios or questions:

- Q1~Q6 are from Cultural Values domain
- Q7~Q13 are from Social Support domain
- Q14~Q18 are from Coping Style domain

As a result, the interview questions are designed as below:

- **Cultural Values**

1. *Q1- General Question:* were you able to recognize any noticeable differences between responses from **Taylor** and **Skylor?**
2. *Q2*- *Specific Questions*: how did **Taylor** and **Skylor** address **cultural values** differently in your opinion? Among the many aspects for comparison, you could consider if one of the responses provides:
3. more accurate
4. more relevant
5. more clear and easier to understand
6. more actionable advice
7. more satisfaction
8. less biased than the other

- **Social Support**

1. *Q1- General Question:* were you able to recognize any noticeable differences between answers from **Taylor** and **Skylor**?
2. *Q2*- *Specific Questions*: how effective were **Taylor** and **Skylor** in offering suggestions to help caregivers build connections with others or navigate relevant resources? Among the many aspects for comparison, you could consider if one of the responses provides:
3. more accurate
4. more relevant
5. more clear and easier to understand
6. more actionable advice
7. more satisfaction
8. less biased than the other

- **Coping Style**

1. *Q1- General Question:* were you able to recognize any noticeable differences between answers from **Taylor** and **Skylor***?*
2. *Q2- Specific Questions*: how well did **Taylor** and **Skylor** provide **practical/feasible coping strategy suggestions** for caregivers? Among the many aspects for comparison, you could consider if one of the responses provides:
3. more accurate
4. more relevant
5. more clear and easier to understand
6. more actionable advice
7. more satisfaction
8. less biased than the other

Additionally, in the end of the interview, there are two questions regarding the limitations and future work: (1) after looking at the responses collected from C1 and C2, did you happen to find anything missing? (2) if so, how can we make the testing questions or scenarios better in the future to satisfy your requirements?

**Group 3: Interview Questions**

Before the interview begins, while completing the survey, participants in this group are required to review responses collected under two conditions–the baseline (i.e., C1) and the experimental (i.e., C2)-across 14 testing scenarios or questions:

- Q1~Q5 are from subdomain Causes and Characteristics.
- Q6~Q8 are from subdomain Health Risk and Promotion.
- Q9~Q12 are from subdomain Communication and Behavior.
- Q13~Q14 are from subdomain Care Consideration.

Additionally, all the above subdomains are from domain Dementia Literacy. As a result, the interview questions are designed as below:

- **Causes and Characteristics**

1. *Q1 – General Question*: were you able to recognize any noticeable differences between responses from **Skylor** and **Taylor**?
2. *Q2 – Specific Questions*: how effective were **Skylor** and **Taylor** in enhancing caregivers’ understanding of dementia causes or characteristics? Among the many aspects for comparison, you could consider if one of the responses provides:
3. more accurate
4. more relevant
5. more clear and easier to understand
6. more actionable advice
7. more satisfaction
8. less biased than the other

- **Health Risk and Promotion**

1. *Q1 – General Question*: were you able to recognize any noticeable differences between responses from **Skylor** and **Taylor**?
2. *Q2 – Specific Questions*: how effective were **Skylor** and **Taylor** in **providing suggestions in addressing health risks** and **promoting strategies related to dementia**? Among the many aspects for comparison, you could consider if one of the responses provides:
3. more accurate
4. more relevant
5. more clear and easier to understand
6. more actionable advice
7. more satisfaction
8. less biased than the other

- **Communication and Behavior**

1. *Q1 – General Question*: were you able to recognize any noticeable differences between answers from **Skylor** and **Taylor**?
2. *Q2 – Specific Questions*: how effective were **Skylor** and **Taylor** in providing **practical and timely advice for managing dementia-related communication and behavioral problems?** Among the many aspects for comparison, you could consider if one of the responses provides:
3. more accurate
4. more relevant
5. more clear and easier to understand
6. more actionable advice
7. more satisfaction
8. less biased than the other

- **Care Consideration**

1. *Q1 – General Question*: were you able to recognize any noticeable differences between answers from **Skylor** and **Taylor**?
2. *Q2 – Specific Questions*: how effective were **Skylor** and **Taylor** in **helping reduce the emotional or physical burden of caregiving by suggesting resources or strategies?** Among the many aspects for comparison, you could consider if one of the responses provides:
3. more accurate
4. more relevant
5. more clear and easier to understand
6. more actionable advice
7. more satisfaction
8. less biased than the other

Additionally, in the end of the interview, there are two questions regarding the limitations and future work: (1) after looking at the responses collected from C1 and C2, did you happen to find anything missing? (2) if so, how can we make the testing questions or scenarios better in the future to satisfy your requirements?

**Group 4: Interview Questions**

Before the interview begins, while completing the survey, participants in this group are required to review responses collected under two conditions–the baseline (i.e., C1) and the experimental (i.e., C2)-across 14 testing scenarios or questions:

- Q1~Q5 are from subdomain Causes and Characteristics.
- Q6~Q8 are from subdomain Health Risk and Promotion.
- Q9~Q12 are from subdomain Communication and Behavior.
- Q13~Q14 are from subdomain Care Consideration.

Additionally, all the above subdomains are from domain Dementia Literacy. As a result, the interview questions are designed as below:

- **Causes and Characteristics**

1. *Q1 – General Question*: were you able to recognize any noticeable differences between responses from **Taylor** and **Skylor**?
2. *Q2 - Specific Questions*: how effective were **Taylor** and **Skylor** in **enhancing caregivers’ understanding of dementia causes** or **characteristics**? Among the many aspects for comparison, you could consider if one of the responses provides:
3. more accurate
4. more relevant
5. more clear and easier to understand
6. more actionable advice
7. more satisfaction
8. less biased than the other

- **Health Risk and Promotion**

1. *Q1 – General Question*: were you able to recognize any noticeable differences between responses from **Taylor** and **Skylor**?
2. *Q2 – Specific Questions*: how effective were **Taylor** and **Skylor** in **providing suggestions in addressing health risks** and **promoting strategies related to dementia**? Among the many aspects for comparison, you could consider if one of the responses provides:
3. more accurate
4. more relevant
5. more clear and easier to understand
6. more actionable advice
7. more satisfaction
8. less biased than the other

- **Communication and Behavior**

1. *Q1 – General Question*: were you able to recognize any noticeable differences between answers from **Taylor** and **Skylor**?
2. *Q2 – Specific Questions*: how effective were **Taylor** and **Skylor** in **providing practical and timely advice for managing dementia-related communication and behavioral problems**? Among the many aspects for comparison, you could consider if one of the responses provides:
3. more accurate
4. more relevant
5. more clear and easier to understand
6. more actionable advice
7. more satisfaction
8. less biased than the other

- **Care Consideration**

1. *Q1 – General Question*: were you able to recognize any noticeable differences between answers from **Taylor** and **Skylor**?
2. *Q2 – Specific Questions*: how effective were **Taylor** and **Skylor** in **helping reduce the emotional or physical burden of caregiving by suggesting resources or strategies**? Among the many aspects for comparison, you could consider if one of the responses provides:
3. more accurate
4. more relevant
5. more clear and easier to understand
6. more actionable advice
7. more satisfaction
8. less biased than the other

Additionally, in the end of the interview, there are two questions regarding the limitations and future work: (1) after looking at the responses collected from C1 and C2, did you happen to find anything missing? (2) if so, how can we make the testing questions or scenarios better in the future to satisfy your requirements?
