# Supplementary material for: Empowering Informal Caregivers of Persons With Early-Stage Dementia by Large Language Models: Mixed Methods Evaluation
Source: JMIR Form Res. 2026 Mar 5;10:e79975. doi: 10.2196/79975 (PMC12978894; doi:10.2196/79975)
Supplement: Multimedia Appendix 7 [file formative-v10-e79975-s007.pdf]

Table S1. Results of the Mann–Whitney U tests comparing baseline and experimental conditions across evaluation questions. These results correspond to ***Figure 3*** in the main manuscript.

EQ #	U-statistic	P-value	Distribution Comparison
1	17746.5	0.4927120006676545	BC and EC are similar
2	17755.0	0.46515921701175544	BC and EC are similar
3	18277.5	0.8695111669299835	BC and EC are similar
**4**	**14660.5**	**0.0001715812247345768 ★**	**BC and EC are different**
5	17476.0	0.340810171661183	BC and EC are similar
**6**	**15306.0**	**0.00281426710176572 ★**	**BC and EC are different**
7	18079.0	0.7182039060388797	BC and EC are similar
8	18274.0	0.8647671659117145	BC and EC are similar
**9**	**15821.0**	**0.011621425757608052 ★**	**BC and EC are different**

Here, **BC** represents the *baseline condition* (C1 in the main manuscript), and **EC** represents the *experimental condition* (C2 in the main manuscript).

For the comparisons, we used a significance threshold of **P = 0.05**, corresponding to a 95% confidence level. Specifically:

1. If **P < 0.05**, we conclude that there is a **significant difference** between BC and EC—that is, the two conditions have **different distributions**.
2. If **P ≥ 0.05**, we conclude that there is **no significant difference** between BC and EC—that is, the two conditions have **similar distributions**.

Accordingly, based on the P-values reported in the above **Table S1**, we reached the conclusion presented in the main manuscript that:

for evaluation items Q1–Q9 across **four domains (192 responses): cultural values, social support, coping style, and dementia literacy**. Statistically significant differences were observed for evaluation **Q4 (usefulness and supportiveness, P = 0.0001715812247345768**), evaluation **Q6 (overall satisfaction, P = 0.00281426710176572)**, and evaluation **Q9 (response relevance, P = 0.011621425757608052),** indicating that **C2 outperformed C1 in these three aspects (Mann–Whitney U test; *P*<0.05).**

Table S2. Results of the Mann–Whitney U tests comparing baseline and experimental conditions across evaluation questions. These results correspond to ***Figure 4*** in the main manuscript.

EQ #	U-statistic	P-value	Distribution Comparison
1	502.5	0.08403707894787009	BC and EC are similar
2	552.0	0.25016342640544975	BC and EC are similar
3	587.0	0.47012748962421513	BC and EC are similar
4	528.0	0.15191591769379456	BC and EC are similar
5	585.0	0.46567187506626573	BC and EC are similar
**6**	**421.0**	**0.007494349690097845 ★**	**BC and EC are Different**
7	589.5	0.4933341287732409	BC and EC are similar
8	629.0	0.8208732261430952	BC and EC are similar
**9**	**436.0**	**0.01068205918643196 ★**	**BC and EC are Different**

Here, **BC** represents the *baseline condition* (C1 in the main manuscript), and **EC** represents the *experimental condition* (C2 in the main manuscript).

For the comparisons, we used a significance threshold of **P = 0.05**, corresponding to a 95% confidence level. Specifically:

1. If **P < 0.05**, we conclude that there is a **significant difference** between BC and EC—that is, the two conditions have **different distributions**.
2. If **P ≥ 0.05**, we conclude that there is **no significant difference** between BC and EC—that is, the two conditions have **similar distributions**.

Accordingly, based on the P-values reported in **Table S2**, we reached the conclusion presented in the main manuscript that:

for evaluation items Q1–Q9 within the **cultural values domain (36 responses**). Statistically significant differences were observed for evaluation **Q6 (overall satisfaction, P = 0.007494349690097845)** and evaluation **Q9 (response relevance, P = 0.01068205918643196**), indicating that **C2 outperformed C1 in these two aspects (Mann–Whitney U test; P<0.05).**

Table S3. Results of the Mann–Whitney U tests comparing baseline and experimental conditions across evaluation questions. These results correspond to ***Figure 5*** in the main manuscript.

EQ #	U-statistic	P-value	Distribution Comparison
1	3579.5	0.8409027771150863	BC and EC are Similar
2	3528.0	1.0	BC and EC are Similar
3	3609.5	0.4861043947960322	BC and EC are Similar
**4**	**2341.5**	**1.8539460067479616e-06 ★**	**BC and EC are Different**
5	3352.0	0.5002408329697325	BC and EC are Similar
**6**	**2998.0**	**0.04684496028137807 ★**	**BC and EC are Different**
7	3565.5	0.882634162266415	BC and EC are Similar
8	3505.5	0.9207220532704454	BC and EC are Similar
9	3121.5	0.10301544257514618	BC and EC are Similar

Here, **BC** represents the *baseline condition* (C1 in the main manuscript), and **EC** represents the *experimental condition* (C2 in the main manuscript).

For the comparisons, we used a significance threshold of **P = 0.05**, corresponding to a 95% confidence level. Specifically:

1. If **P < 0.05**, we conclude that there is a **significant difference** between BC and EC—that is, the two conditions have **different distributions**.
2. If **P ≥ 0.05**, we conclude that there is **no significant difference** between BC and EC—that is, the two conditions have **similar distributions**.

Accordingly, based on the P-values reported in the above **Table S3**, we reached the conclusion presented in the main manuscript that:

for evaluation items Q1–Q9 within the **dementia literacy domain (84 responses)**. Statistically significant differences were observed for evaluation **Q4 (usefulness and supportiveness,** P = **1.8539460067479616e-06)** and evaluation **Q6 (overall satisfaction,** P =**0.04684496028137807),** indicating that **C2 outperformed C1 in these two aspects (Mann–Whitney U test; P<0.05).**
