# Supplementary material for: Empowering Informal Caregivers of Persons With Early-Stage Dementia by Large Language Models: Mixed Methods Evaluation
Source: JMIR Form Res. 2026 Mar 5;10:e79975. doi: 10.2196/79975 (PMC12978894; doi:10.2196/79975)
Supplement: Multimedia Appendix 8 [file formative-v10-e79975-s008.docx]

**Table S1.** Reports on the comparison of answer-score distribution in terms of mean values for each evaluation criterion (answers collected across all four domains).

| Mean | EQ1 | EQ2 | EQ3 | EQ4 | EQ5 | EQ6 | EQ7 | EQ8 | EQ9 |
| --- | --- | --- | --- | --- | --- | --- | --- | --- | --- |
| **C1** | 6.15 | 6.45 | 6.40 | 5.86 | 6.00 | 5.44 | 6.07 | 6.39 | 5.67 |
| **C2** | 6.21 | 6.51 | 6.41 | 6.26 | 6.10 | 4.58 | 6.11 | 6.45 | 5.96 |

**Table S2.** Reports on the comparison of answer-score distribution in terms of standard deviation values for each evaluation criterion (answers collected across all four domains).

| Std Dev | EQ1 | EQ2 | EQ3 | EQ4 | EQ5 | EQ6 | EQ7 | EQ8 | EQ9 |
| --- | --- | --- | --- | --- | --- | --- | --- | --- | --- |
| **C1** | 1.03 | 0.82 | 0.95 | 1.30 | 1.22 | 1.49 | 1.22 | 1.04 | 1.34 |
| **C2** | 1.03 | 0.78 | 0.96 | 1.20 | 1.15 | 2.16 | 1.21 | 0.92 | 1.32 |

**Table S3.** Reports on the comparison of answer-score distribution in terms of mean values for each evaluation criterion (answers collected from the cultural values domain).

| Mean | EQ1 | EQ2 | EQ3 | EQ4 | EQ5 | EQ6 | EQ7 | EQ8 | EQ9 |
| --- | --- | --- | --- | --- | --- | --- | --- | --- | --- |
| **C1** | 5.72 | 5.97 | 5.97 | 5.75 | 5.44 | 5.00 | 5.78 | 6.17 | 5.44 |
| **C2** | 6.08 | 6.22 | 6.08 | 6.11 | 5.64 | 5.53 | 5.94 | 6.25 | 5.97 |

**Table S4.** Reports on the comparison of answer-score distribution in terms of standard deviation values for each evaluation criterion (answers collected from the cultural values domain).

| Std Dev | EQ1 | EQ2 | EQ3 | EQ4 | EQ5 | EQ6 | EQ7 | EQ8 | EQ9 |
| --- | --- | --- | --- | --- | --- | --- | --- | --- | --- |
| **C1** | 0.84 | 0.87 | 0.83 | 1.23 | 1.12 | 1.25 | 1.16 | 1.01 | 0.86 |
| **C2** | 0.79 | 0.79 | 0.92 | 1.07 | 1.13 | 1.57 | 1.15 | 0.92 | 0.76 |

**Table S5.** Reports on the comparison of answer-score distribution in terms of mean values for each evaluation criterion (answers collected from the dementia literacy domain).

| Mean | EQ1 | EQ2 | EQ3 | EQ4 | EQ5 | EQ6 | EQ7 | EQ8 | EQ9 |
| --- | --- | --- | --- | --- | --- | --- | --- | --- | --- |
| **C1** | 6.46 | 6.90 | 6.95 | 6.21 | 6.45 | 6.11 | 6.31 | 6.49 | 6.32 |
| **C2** | 6.46 | 6.90 | 6.94 | 6.82 | 6.54 | 6.45 | 6.35 | 6.57 | 6.57 |

**Table S6.** Reports on the comparison of answer-score distribution in terms of standard deviation values for each evaluation criterion (answers collected from the dementia literacy domain).

| Std Dev | EQ1 | EQ2 | EQ3 | EQ4 | EQ5 | EQ6 | EQ7 | EQ8 | EQ9 |
| --- | --- | --- | --- | --- | --- | --- | --- | --- | --- |
| **C1** | 0.98 | 0.33 | 0.26 | 1.08 | 0.93 | 1.37 | 1.22 | 1.18 | 1.09 |
| **C2** | 0.97 | 0.33 | 0.24 | 0.60 | 0.79 | 1.13 | 1.13 | 0.98 | 0.92 |
