# Supplementary material for: Empowering Informal Caregivers of Persons With Early-Stage Dementia by Large Language Models: Mixed Methods Evaluation
Source: JMIR Form Res. 2026 Mar 5;10:e79975. doi: 10.2196/79975 (PMC12978894; doi:10.2196/79975)
Supplement: Multimedia Appendix 9 [file formative-v10-e79975-s009.pdf]

Cond1 – Vanilla ChatGPT(C1)   vs. Cond2 – PromptEngineered ChatGPT(C2)

Domain 1 - Cultural Values

1. I am seeking an experienced healthcare provider, such as a neurologist or dementia specialist, who is fluent in my language (e.g., Vietnamese, Chinese, Korean, Spanish) in [county name]. I need to schedule an appointment with such a specialist to assess my mother's cognitive condition and provide a capacity evaluation letter for the power of attorney designation process. Could you assist me in finding this specialist?

Cond1	Cond2
To help you find a specialist who meets these criteria, I suggest the following steps:<br><br>1. **Local Healthcare Networks**:<br>Contact large hospitals or healthcare networks in your area. Many facilities offer multilingual services and have directories of specialists who speak various languages. For example:<br>o Search for neurologists or dementia specialists in your local hospital's department of neurology or geriatrics.<br>o Ask if they have providers who speak your preferred language.<br>2. **Online Directories**:<br>Websites like Zocdoc, Healthgrades, or Amwell allow you to filter searches by specialty, location, and language preference.<br>3. **Community Organizations**:<br>Local cultural or community organizations often have resources or recommendations for multilingual healthcare providers. They may also assist with scheduling and navigating the healthcare process.<br>4. **Referrals from Primary Care Providers**:<br>If your mother has a primary care physician, they can provide a referral to a neurologist or specialist and may know of providers fluent in her language.<br>5. **Area Agencies on Aging (AAA)**:<br>Contact your local AAA office for resources and recommendations for specialists who provide capacity evaluations and language support.<br><br>If you'd like, you can share the county or general area, and I can assist further by helping locate options specific to your region.	I can help guide you in finding a healthcare provider who meets your needs. However, I don't have access to specific local databases for real-time appointments or availability. Here are steps you can take to find an experienced neurologist or dementia specialist who speaks your language and can assist with the cognitive evaluation and power of attorney process:<br><br>1. **Alzheimer's Association**: Use their **Community Resource Finder** to locate specialists in your county. You can search by location, services, and languages spoken.<br>o **Link**: Community Resource Finder<br>2. **Health Insurance Provider**: Check with your health insurance company for a list of neurologists or dementia specialists in your area who speak your language. Insurance companies often have provider directories where you can filter by specialty and language.<br>3. **Local Hospitals or Clinics**: Contact local hospitals or medical centers and ask if they have dementia specialists or neurologists fluent in your language. Many hospitals have departments dedicated to Alzheimer's or dementia care.<br>4. **Cultural Organizations**: Reach out to local community centers or cultural organizations serving your language group (e.g., Vietnamese, Chinese, Korean, Spanish) for recommendations on healthcare providers who speak the language and specialize in dementia.<br>5. **Online Directories**: Use online medical directories such as:<br>o **Zocdoc** (www.zocdoc.com): You can search for neurologists or dementia specialists, check their availability, and filter by language spoken.<br>o **American Academy of Neurology** (www.aan.com): A directory to find neurologists who specialize in dementia care.<br>6. **Alzheimer's Association Helpline**: You can also contact the Alzheimer's Association's

	helpline (800.272.3900) to get personalized help in finding a specialist who speaks your language and can assist with the evaluation and power of attorney process.<br><br>Let me know if you need further assistance in a specific county or if you'd like more information on any of these options!

2. I am hesitant to seek help because I am concerned that my friends and neighbors might judge my husband if they learn about his cognitive condition. What should I do?

Cond1	Cond2

It's natural to feel hesitant about seeking help when you're concerned about how others might perceive your situation. However, prioritizing your husband's health and well-being—and your own—is essential. Here are some steps to help you navigate this:<br><br>**1. Educate Yourself About the Condition**<br><br>● Learning more about your husband's cognitive condition can help you feel more confident in addressing it and discussing it with others, should the need arise.<br>● Understanding the condition also allows you to separate the reality of his needs from potential stigma.<br><br>**2. Seek Professional Support**<br><br>● Reach out to healthcare providers, therapists, or support groups. Professionals maintain confidentiality, so you can get help without worrying about others knowing.<br>● Consider involving a social worker or counselor who specializes in cognitive conditions; they can help you navigate resources and manage your concerns about judgment.<br><br>**3. Find Trusted Allies**<br><br>● Identify close friends, family members, or neighbors who are compassionate and understanding. Sharing with one or two trusted individuals can provide emotional support.<br>● Frame the discussion around your husband's needs rather than the diagnosis, if that feels more comfortable.<br><br>**4. Join a Support Group**<br><br>● Connecting with people facing similar challenges can be incredibly helpful. These groups often offer practical advice and emotional support without judgment.<br>● Many groups meet online, offering anonymity if you're worried about privacy.<br><br>**5. Set Boundaries**<br><br>● It's okay to keep personal details private. If people inquire, you can share only what you're comfortable with, such as, "He's	It's completely understandable to feel concerned about how others might perceive your husband's cognitive condition, especially given the stigma that can sometimes surround Alzheimer's and other forms of dementia. Here are a few steps to help manage your feelings and approach the situation with confidence:<br><br>**1. Understand that Stigma is Common, But Can Be Overcome**<br><br>Many people face similar feelings of isolation when dealing with Alzheimer's or dementia. The stigma often arises from a lack of understanding or awareness about these conditions. The more you learn about Alzheimer's, the better equipped you'll be to educate others and challenge misconceptions.<br><br>**2. Educate Yourself and Others**<br><br>Talking about Alzheimer's can reduce stigma over time. You can choose to educate close friends and neighbors in a way that feels comfortable for you. You might not need to disclose everything about your husband's condition, but even sharing general information can help people understand what he's going through.<br><br>The **Alzheimer's Association** provides helpful resources for discussing Alzheimer's with family, friends, and others, which might make the conversation easier when you're ready.<br><br>**3. Set Boundaries**<br><br>It's perfectly okay to set boundaries regarding who knows about your husband's condition. You don't have to share more than you're comfortable with. You might decide to tell a few close friends or family members, or simply keep things private for now. The important thing is that you have control over what is shared and with whom.<br><br>**4. Focus on Support, Not Judgment**<br><br>Your well-being is important, and seeking support is essential. It may be helpful to connect with others who understand the challenges of caregiving. Support groups for Alzheimer's caregivers, either in-person or online, can offer a space where you can talk openly without fear of judgment. Some **ALZConnected®** forums might be particularly helpful in connecting with people who share similar experiences.

facing some health challenges, and we're working with professionals."<br><br>**6. Reframe the Fear of Judgment**<br><br>● Remember, seeking help is an act of strength and love, not something to be ashamed of.<br>● Most people admire courage in the face of adversity. If anyone judges, it reflects on them, not on you or your husband.<br><br>**7. Consider Counseling for Yourself**<br><br>● Counseling can help you process your concerns about stigma and judgment. It also gives you tools to handle challenging conversations with confidence.<br><br>Would you like suggestions for specific resources or support networks?	**5. Seek Professional Support**<br><br>A counselor, social worker, or therapist specializing in dementia care can help you navigate your feelings about judgment and privacy. These professionals can offer strategies for dealing with your concerns and provide emotional support.<br><br>**6. Confidentiality and the Alzheimer's Association**<br><br>If you're unsure about disclosing your husband's condition, the **Alzheimer's Association** Helpline (800.272.3900) can provide support and guide you on how to handle conversations with others, including tips on maintaining your privacy.<br><br>**7. Remember, It's About Your Husband's Well-Being**<br><br>It's natural to want to protect your husband's dignity and privacy. But ultimately, your decision to seek help—whether that's professional, community, or emotional support—is a vital part of maintaining his well-being and ensuring that both of you have the care and support needed as the disease progresses.<br><br>If you're feeling overwhelmed, don't hesitate to reach out for support. You deserve to be heard, and there are resources and people who can help without judgment.<br><br>Let me know if you'd like additional resources or support groups that focus on dementia caregiving and help for spouses.

3. As a caregiver for my mother, who is forgetful and speaks Korean, I sometimes struggle because my own Korean isn't very proficient. Are there resources available that are tailored to caregivers like me, especially those that respect and reflect my Korean cultural background?

Cond1	Cond2
Caring for a loved one, especially when navigating language and cultural differences, can be challenging. Here are some tailored resources and suggestions for you:<br><br>**1. Language and Communication Support**<br><br>● **Korean-English Phrasebooks for Caregiving**: Look for caregiving-specific language guides or apps like *Papago* or *Google Translate* with a focus on healthcare and daily needs.<br>● **Community Classes**: Check if there are Korean language or culture workshops in your area, often offered by cultural centers or libraries.<br><br>**2. Culturally Relevant Caregiving Resources**<br><br>● **The Korean American Family Service Center (KAFSC)**: Offers support tailored to Korean-speaking families, including caregiver resources.<br>● **Korean Community Centers**: Many larger cities have Korean-specific support groups or centers that provide advice and cultural resources.<br>● **Local Senior Support Networks**: Contact your area's Agency on Aging for referrals to programs that work with Korean communities.<br><br>**3. Dementia or Forgetfulness-Specific Support**<br><br>● **Alzheimer's Association Resources**: Their website includes Korean-language materials and tips for managing memory-related conditions.<br>● **Books and Media in Korean**: Libraries or online stores may have simple Korean-language materials you can use to engage with your mom in familiar and comforting ways.<br><br>**4. Caregiver Support Groups**<br><br>● **Online Forums and Social Media**: Look for caregiver groups, especially those focusing on Asian-American or Korean caregivers.<br>● **Faith-Based Organizations**: Many Korean churches have caregiver support ministries or programs.	Caring for a loved one, especially when there are language and cultural differences, can be challenging, but there are several resources that can offer support tailored to your situation as a caregiver for your mother, who speaks Korean. Here are some resources that may help:<br><br>**1. Alzheimer's Association – Korean Language Resources**<br><br>The **Alzheimer's Association** offers a variety of resources for caregivers in different languages, including Korean. You can find information on caregiving, managing symptoms, and support groups, all in Korean. They also offer a 24/7 helpline in multiple languages (including Korean), which can help guide you through caregiving challenges.<br><br>● **Korean Language Resources**: alz.org/Korean<br>● **Alzheimer's Association Helpline**: 800.272.3900 (Available in Korean)<br><br>**2. National Asian Alzheimer's and Dementia Resource Center (NAADRC)**<br><br>The **NAADRC** provides culturally competent resources and materials for Alzheimer's caregivers in Asian communities, including Korean Americans. Their resources include educational materials, webinars, and culturally relevant support for caregiving.<br><br>● **Website**: NAADRC<br>● **Alzheimer's Disease and Dementia Resources for Asian Communities**: The center has a variety of tools specifically geared toward caregivers who may be facing language barriers.<br><br>**3. Korean American Alzheimer's Association (KAAA)**<br><br>The **Korean American Alzheimer's Association** offers resources in Korean for families coping with Alzheimer's disease. They provide information on managing Alzheimer's care, support groups, and other community-based resources tailored for the Korean-speaking population.

Would you like assistance finding specific organizations or tools near you? Let me know how else I can help!	• **Website**: Look for local Korean American Alzheimer's resources through community centers or healthcare providers in your area.<br><br>**4. Local Korean Community Organizations**<br><br>Many local Korean community organizations or churches may offer caregiver support, counseling, and resources for dementia care. These organizations often have culturally competent programs and can connect you with other caregivers who share similar cultural backgrounds.<br><br>• **Search for local Korean community centers** or organizations like **Korean American Senior Citizens Association (KASCA)**, or other groups that specialize in senior and healthcare services.<br><br>**5. Support Groups for Korean Caregivers**<br><br>Support groups for caregivers can provide a safe space for you to share your experiences and challenges. Many Korean American communities offer support groups where you can connect with others who are going through similar experiences.<br><br>• **Local Alzheimer's chapters** often have bilingual support groups (including Korean). You can reach out to your local **Alzheimer's Association** chapter for more information.<br>• **ALZConnected®**: An online community where you can connect with other caregivers and ask questions, including those about navigating language barriers.<br><br>**6. Language Assistance Services**<br><br>Many healthcare providers offer language assistance services for caregivers and patients. You can ask about these services when you schedule doctor appointments for your mother or contact local hospitals and medical centers. These services can help bridge the language gap and ensure that you and your mother understand medical instructions, treatment plans, and caregiving advice.<br><br>• **Interpreter Services**: Many hospitals and clinics offer interpreter services, including for Korean, which can help during doctor visits.<br><br>**7. Counseling and Therapy Services**

	If you're feeling overwhelmed by the caregiving duties and language barriers, consider counseling services that can offer both emotional support and strategies for managing caregiving stress. Some mental health professionals specialize in helping caregivers from culturally diverse backgrounds, including Korean families.<br><br>**8. Korean Language Caregiver Guides**<br><br>There are caregiver handbooks available in Korean that provide useful tips for managing Alzheimer's care. These guides often include sections on communication, understanding Alzheimer's symptoms, and day-to-day caregiving tips, all designed with the Korean culture in mind.<br><br>If you'd like more information or help in finding specific resources in your area, I can help you look into local services or guide you through further steps. Your caregiving efforts are incredibly important, and it's commendable that you're seeking out culturally relevant support.

4. As a caregiver for my loved one, I want to ensure that I communicate effectively with them while respecting our cultural values. What strategies can I use to bridge any language or cultural gaps in our interactions?

**Cond1**	**Cond2**
As a caregiver, effective communication while respecting cultural values is key to fostering a	As a caregiver for your loved one, it's important to recognize that effective communication is key to maintaining their dignity, emotional well-being, and

supportive and positive relationship. Here are some strategies to bridge any language or cultural gaps:

1. **Learn About Their Culture**: Take the time to understand your loved one's cultural background, traditions, and values. This can help you avoid misunderstandings and ensure your actions and words are respectful.
2. **Use Simple and Clear Language**: If there's a language barrier, try to speak slowly, use clear and simple words, and avoid idiomatic expressions that may not be easily understood. Non-verbal cues like gestures or visual aids can also be helpful.
3. **Listen Actively**: Be patient and open when listening. Encourage your loved one to express themselves, and be sensitive to both verbal and non-verbal communication. Sometimes, silence can be an important part of communication in certain cultures.
4. **Seek Cultural Sensitivity Training**: Consider taking cultural competency courses or workshops that focus on understanding different communication styles, customs, and social norms in caregiving. This can provide valuable insight into how to communicate more effectively and avoid missteps.
5. **Incorporate Traditions and Rituals**: Whenever possible, honor cultural customs or practices that are meaningful to your loved one. This could be through food, music, prayers, or routines that make them feel at home and respected.
6. **Use Technology and Translation Tools**: If there is a language barrier, make use of translation apps or tools to help you communicate more effectively. You can also use video calls to facilitate better understanding when physical presence is not possible.
7. **Establish a Comfortable Environment**: Make sure the physical and emotional environment is welcoming and safe. A calm, culturally respectful space can encourage better communication.
8. **Be Mindful of Body Language**: Different cultures interpret body language differently, so be aware of how gestures, eye contact, and physical proximity are perceived.
9. **Encourage Family Involvement**: In some cultures, family plays a crucial role in caregiving. Engaging with family members can help you understand the cultural expectations and support your loved one in a way that aligns with their values.

fostering a connection. Bridging language and cultural gaps is especially important when your loved one has a cognitive condition like Alzheimer's, as it can help reduce confusion, anxiety, and enhance their quality of life.

Here are some strategies you can use to communicate effectively while respecting cultural values:

1. Simplify Your Language

- **Use Clear and Simple Language**: Alzheimer's often affects a person's ability to process complex information. Speak slowly and use short, simple sentences to help them understand.
- **Avoid Complex Questions**: Instead of asking multiple questions at once, break them down into one simple question at a time.

2. Non-Verbal Communication

- **Body Language**: Use facial expressions, gestures, and touch to communicate warmth, understanding, and reassurance. In many cultures, non-verbal cues play a significant role in communication.
- **Maintain Eye Contact**: In some cultures, eye contact is seen as a sign of respect, while in others, it may be avoided. Be mindful of this when engaging with your loved one.

3. Incorporate Cultural Touchstones

- **Cultural References**: Use familiar cultural phrases, traditions, or comforting practices that are meaningful to your loved one. For example, you might use traditional greetings, share stories or songs from their childhood, or include religious rituals that may provide comfort.
- **Respect for Authority and Family Structure**: In some cultures, elders are highly respected, and maintaining this respect can strengthen your relationship. Always refer to your loved one with honor, acknowledging their life experience.

4. Use Culturally Relevant Resources

- **Cultural Sensitivity in Caregiving**: Seek out caregiving resources that take into account the specific cultural background of your loved one. For example, Alzheimer’s resources that

10. **Respect Their Pace**: Understand that cultural differences might affect how quickly someone adjusts to new situations or accepts care. Be patient and give your loved one the time they need to feel comfortable.

By combining cultural understanding with clear communication, you can provide care that is both compassionate and respectful.

reflect Korean, Vietnamese, or Hispanic cultural norms can provide tailored advice on communication and caregiving strategies.

- **Interpreter Services**: If your loved one speaks a language you're not fluent in, look for interpreter services or bilingual healthcare providers. This will ensure you're both on the same page about medical care, instructions, and any changes in their condition.

5. Respect Their Personal Preferences

- **Personal and Cultural Preferences**: Many individuals have personal ways of communicating, eating, or performing daily rituals, which can be tied to their cultural identity. For example, some cultures emphasize group activities or shared meals as important social rituals. Try to incorporate these into your caregiving routine.
- **Offer Choices**: Alzheimer's can cause your loved one to feel a loss of control. Offering small choices (e.g., "Would you like to wear this shirt or that shirt?") can provide them with some autonomy and help reduce frustration.

6. Establish a Routine

- **Consistency and Familiarity**: A consistent routine helps reduce anxiety and confusion, particularly for someone with Alzheimer's. Incorporate familiar cultural activities into their daily schedule, like family meals, watching traditional shows, or music from their cultural background.
- **Cultural Holidays and Celebrations**: Acknowledge and celebrate cultural holidays and traditions that are important to your loved one, even in small ways. This can help them feel connected to their identity and bring comfort.

7. Be Patient and Compassionate

- **Avoid Confrontation**: Alzheimer's often leads to confusion, aggression, or frustration, and it's important not to take these behaviors personally. In some cultures, there's a strong value placed on maintaining peace and avoiding confrontation. Maintain a calm demeanor and avoid arguing, even if they repeat something or make no sense.

	● **Listen Actively**: Show empathy and active listening by nodding, making appropriate responses, and maintaining a relaxed posture. Even if your loved one's words don't make sense, try to validate their feelings.<br><br>**8. Seek Support from Culturally Competent Professionals**<br><br>● **Culturally Sensitive Healthcare Providers**: Finding doctors, social workers, or therapists who understand the cultural nuances of caregiving can make a huge difference. Look for professionals who are trained to work with individuals from diverse cultural backgrounds and who are sensitive to their unique needs.<br>● **Community Support Groups**: Many cultures have support groups where caregivers can share their experiences and gain advice from others who understand their specific challenges. Check with local community centers, religious groups, or ethnic-specific organizations for support groups.<br><br>**9. Use Music, Art, or Religion**<br><br>● **Familiar Music or Rituals**: Music can have a calming effect and can help evoke memories of past experiences. Playing traditional music or singing songs that your loved one used to enjoy may help with communication.<br>● **Religious or Spiritual Practices**: Many families incorporate faith-based practices into caregiving. If this is part of your cultural background, including prayer, meditation, or religious rituals can offer comfort to both you and your loved one.<br><br>**10. Keep the Environment Familiar**<br><br>● **Cultural Environment**: Decorate their living space with objects, symbols, or art that reflects their cultural heritage. This can make them feel more comfortable and grounded in familiar surroundings.<br>● **Familiar Foods**: Offering traditional foods and maintaining familiar meal practices can enhance the quality of life for your loved one and help them feel more at ease.<br><br>**Additional Resources:**<br><br>● **Alzheimer's Association**: Look for caregiving tools and materials in your

	language or culture. They have resources for caregivers from different backgrounds.<br>● **Local Cultural Organizations**: Many local cultural organizations offer support, educational programs, and caregiving resources that are culturally relevant.<br>● **Support Groups**: If available in your area, joining a culturally specific support group for caregivers can help you share experiences and learn from others who may have similar cultural challenges.<br><br>By blending culturally respectful caregiving practices with effective communication strategies, you can create an environment where your loved one feels understood and valued, while also making the caregiving process more manageable for yourself. If you'd like further assistance with finding specific resources, feel free to reach out for more detailed information!

5. I live in Virginia and am a caregiver for my family member with dementia. What local resources or organizations in Spanish can provide me with support, whether for caregiving or guidance on managing the situation?

**Cond1**	**Cond2**
In Virginia, there are several resources and organizations that provide support in Spanish for caregivers of individuals with dementia. Here are a few options:	As a caregiver in Virginia, there are several Spanish-language resources and organizations that can provide valuable support, guidance, and information as you

1. **Alzheimer's Association - Virginia Chapter**<br>The Alzheimer's Association offers a range of services for caregivers, including information on dementia, support groups, and guidance for caregivers. They provide some materials in Spanish and may have Spanish-speaking staff or volunteers for assistance.<br>Website: Alzheimer's Association Virginia<br>Phone: 1-800-272-3900<br>2. **Virginia Department for Aging and Rehabilitative Services (DARS)**<br>DARS offers programs to assist caregivers, and their website includes resources for family caregivers in Spanish. They also help with connecting to local services like respite care and support groups.<br>Website: DARS Family Caregiver Support<br>Phone: 1-800-552-3402<br>3. **Caregiver Resource Center - Area Agencies on Aging**<br>Many Area Agencies on Aging in Virginia offer caregiver support services, some of which are available in Spanish. They provide educational resources, caregiving tips, and respite care options.<br>Website: Virginia's Area Agencies on Aging<br>Phone: 1-800-677-1116<br>4. **The National Alliance for Caregiving**<br>While they are a national organization, they provide resources that include caregiving in Spanish and can direct you to local resources.<br>Website: National Alliance for Caregiving<br>5. **Cuidado de la Familia (Family Caregiving)**<br>A Spanish-language resource that includes caregiving advice, articles, and videos for family caregivers of those with dementia or other health issues.<br>Website: Cuidado de la Familia<br><br>If you need more personalized assistance, many local support groups or healthcare providers can also offer connections to Spanish-speaking counselors or peer support groups.	care for your family member with dementia. Here are some key resources to consider:<br><br>**1. Alzheimer's Association – Virginia Chapter**<br><br>• **Spanish Resources**: The Alzheimer's Association offers support, information, and resources in Spanish. This includes educational materials, helplines, and local events tailored to Spanish-speaking families.<br>• **Helpline**: You can call the Alzheimer's Association's 24/7 helpline at 800.272.3900, where assistance in Spanish is available.<br>• **Spanish-language Website**: **Alzheimer's Association en Español** offers a wealth of information, including caregiver resources, tips for managing dementia, and more.<br>• **Community Resource Finder**: You can use the Alzheimer's Association's Community Resource Finder to find local Spanish-speaking healthcare providers, support groups, and other services.<br><br>**2. National Hispanic Council on Aging (NHCOA)**<br><br>• **Website**: The NHCOA provides information and resources that focus on the needs of Hispanic/Latino seniors and their caregivers. While not specific to dementia, they offer culturally relevant resources for caregivers and information about navigating healthcare services in Spanish.<br>• **Website Link**: NHCOA<br><br>**3. AARP Virginia**<br><br>• **Spanish Resources**: AARP offers caregiving tips and resources in Spanish through their **AARP en Español** website. They cover a range of caregiving topics, including dementia care and support for family caregivers.<br>• **Local Chapters**: Reach out to local AARP chapters in Virginia to inquire about Spanish-language programs and support groups.<br><br>**4. Ventanilla de Salud**<br><br>• **What It Offers**: A program by the Mexican Consulate that offers health resources and support for Spanish-speaking individuals. It provides general health information, including resources for Alzheimer's care and dementia.

	• **Contact**: Check with your local Mexican Consulate in Virginia for more details on this program and the health services they offer.<br>• **Website Link**: **Ventanilla de Salud**<br><br>**5. Virginia Department for Aging and Rehabilitative Services (DARS)**<br><br>• **Dementia Care Resources**: DARS offers programs and services for seniors, including resources for caregivers. They may have Spanish-language materials and guidance for managing dementia or other related conditions.<br>• **Local Area Agencies on Aging**: Many local area agencies offer support for caregivers and may have Spanish-speaking staff. You can contact them for resources specific to dementia care.<br>• **Contact Information**: **Virginia DARS**<br><br>**6. Hispanic/Latino Caregiver Support Groups**<br><br>• **Local Support Groups**: Some local communities in Virginia may offer caregiver support groups tailored for Spanish-speaking families. These groups can provide emotional support, shared caregiving experiences, and tips for managing dementia.<br>• **Alzheimer's Association Support Groups**: Some local Alzheimer's Association chapters offer Spanish-speaking support groups. You can inquire with your local chapter or use the Community Resource Finder.<br><br>**7. Inova Health System (Fairfax, VA)**<br><br>• **Cultural Sensitivity**: Inova offers culturally sensitive services, including Spanish-speaking healthcare providers who can assist with dementia and caregiving needs.<br>• **Alzheimer's Care and Memory Services**: Inova offers memory care services and could have resources in Spanish for caregivers.<br>• **Contact**: Inova Health System<br><br>**8. Local Churches and Religious Organizations**<br><br>• Many local churches in Virginia that serve Spanish-speaking communities offer programs or support groups for caregivers. Religious organizations can provide both emotional and

	spiritual support for caregivers and their families.<br><br>**9. Culturally Competent Home Care Agencies**<br><br>● **Home Care Providers**: Look for home care agencies that specialize in dementia care and have Spanish-speaking staff. These agencies can help with in-home care for your loved one, and the caregivers will be able to communicate with your family in Spanish.<br><br>**10. Spanish-language Materials**<br><br>● **Books and Pamphlets**: Many libraries in Virginia and local organizations provide caregiving books and materials in Spanish. You can contact your local library to find resources specifically about dementia care in Spanish.<br><br>**Steps to Take:**<br><br>● **Contact the Alzheimer's Association Helpline** (800.272.3900) to inquire about specific Spanish-language services and support in your area.<br>● **Search for local resources using the Community Resource Finder** to find healthcare providers, agencies, and support groups that offer Spanish-language services.<br>● **Reach out to your local area agency on aging** for additional caregiver services, as they may have bilingual staff or Spanish-language resources.<br><br>Let me know if you need more specific recommendations or help finding any particular resource!

6. My mother has become more forgetful, and I'm worried about her being alone during the day. She has Medicare but is not eligible for Medicaid, and I'm concerned about the financial cost of daycare. Are there any resources or programs for daytime activities for people with memory issues, especially those that offer Spanish-speaking help? My mother doesn't speak English very well and wouldn't be comfortable in a place where no one speaks Spanish. Where can I find more information about these resources?"

Cond1	Cond2
I'm sorry to hear about your mother's situation, and I understand how challenging it can be to find appropriate resources. There are several programs and resources that may help, particularly for individuals with memory issues who also need support in Spanish. Here are some potential options:<br><br>**1. Adult Day Care Programs**<br><br>● **Medicare**: While Medicare generally doesn't cover the cost of adult daycare services, it may cover certain therapies or treatments that can be part of memory care. Some adult daycare centers might also offer sliding scale fees or accept private insurance.<br>● **Specialized Daycare Programs**: Many adult daycare centers offer services specifically for individuals with dementia or memory issues, including memory care activities. Some may offer bilingual services or have Spanish-speaking staff. You can contact local providers directly to inquire about this.<br>● **Alzheimer's Association**: The Alzheimer's Association has local chapters across the U.S., and they often maintain directories of adult daycare programs, some of which cater specifically to Spanish speakers or offer bilingual services. Their website also provides a wealth of information on support services, including caregiver resources.<br><br>**2. Community Resources and Senior Centers**<br><br>● **Area Agencies on Aging (AAA)**: These local agencies may have programs specifically tailored to seniors with memory impairments. Many of these centers provide assistance for seniors who speak languages other than English, including Spanish. Contacting your local AAA can help you find daycare options and other support services.<br>● **Senior Centers**: Some community-based senior centers offer bilingual services and programs for people with memory issues. They may provide transportation, meals, and recreational activities.<br><br>**3. Medicare Advantage Plans and Other Health Insurance Options**<br><br>● **Medicare Advantage Plans**: If your mother has a Medicare Advantage plan, it may offer additional benefits, including coverage for	I understand your concerns about your mother's memory issues and her comfort in a setting where Spanish is spoken. Fortunately, there are resources that can support you in finding daytime activities for her, especially with a focus on Spanish-speaking environments. Here are some options:<br><br>**1. Adult Day Care Programs**<br><br>Many **adult day care programs** provide social and recreational activities for individuals with dementia or memory issues. Some programs are specifically tailored to the needs of Spanish-speaking individuals and caregivers. These programs can offer a safe and engaging environment for your mother during the day while giving you time for yourself.<br><br>**To Find Local Spanish-Speaking Adult Day Care Options:**<br><br>● **National Adult Day Services Association (NADSA)**: The NADSA offers a tool to help you find adult day care programs, some of which may offer Spanish-speaking services. You can search for local providers and inquire about language services. NADSA's website<br>● **Area Agencies on Aging (AAA)**: Many **AAAs** provide information on adult day programs, including those that offer services in Spanish. Contact your local AAA for more information. You can find your local AAA here: Eldercare Locator.<br><br>**2. Local Community Resources for Spanish Speakers**<br><br>Organizations that serve the Latino community often provide memory support programs, including adult day care, respite care, and other services in Spanish. You may want to explore resources that specifically cater to Spanish-speaking individuals and caregivers.<br><br>**Some local resources to consider include:**<br><br>● **The Alzheimer's Association**: They offer resources in Spanish and may be able to connect you with day programs and caregivers who speak Spanish. You can call their helpline at **800.272.3900** for assistance in finding a Spanish-speaking provider.<br>● **Ventanilla de Salud (Health Window)**: As mentioned earlier, this program offered by the Mexican Consulate can provide resources on

adult day services or memory care programs. Some of these plans may also offer extra services like transportation or bilingual caregivers.

- **Supplemental Insurance**: Some private insurance plans or long-term care insurance might help cover daycare costs, especially if they offer a memory care rider.

4. State and Local Programs

- **State Programs for Caregivers**: Each state often has its own programs for caregivers and seniors, including subsidies for adult daycare or respite care. For example, some states offer "programs of all-inclusive care for the elderly" (PACE), which may provide adult daycare as part of a comprehensive package of services.
- **Local Churches or Nonprofits**: Some faith-based organizations or local charities run free or low-cost programs for seniors, including Spanish-speaking caregivers or activity programs.

5. Online Directories and Information

- **Eldercare Locator**: The U.S. Administration on Aging runs the Eldercare Locator, a resource to find services and programs for seniors. You can search for adult daycares, memory care programs, and bilingual services.
- **Care.com**: This website can help you find in-home caregivers, including those who speak Spanish and specialize in dementia or Alzheimer's care. It may not be as cost-effective as daycare, but it could offer an alternative.

How to Find More Information:

- **Call your local Area Agency on Aging**: They can help connect you to adult daycare centers and other resources.
- **Contact your local Alzheimer's Association chapter**: They often have comprehensive lists of dementia-friendly care options, some of which are bilingual.
- **Search online resources**: Websites like Eldercare Locator or Care.com may help you find services and caregivers in your area.

Alzheimer’s care and help you access Spanish-speaking services in your area. Visit Ventanilla de Salud for more information.

3. Medicare Support Programs

Since your mother has Medicare, you may be able to explore some programs or services that could help with her care costs or provide daytime activities. **Medicare does not cover adult daycare** directly, but some Medicare Advantage plans may offer coverage for day programs or respite care as part of their benefits.

- **Medicare Advantage Plans (Part C)**: Some plans may offer additional benefits like adult day services. You should contact her insurance provider to see if any programs or benefits are available.
- **Medicare Savings Programs**: If your mother's income is low, she may be eligible for programs that help cover out-of-pocket costs, even though she doesn't qualify for Medicaid. Contact **1-800-MEDICARE** or visit Medicare.gov for more information.

4. Spanish-Speaking Support Groups

Connecting with other caregivers who speak Spanish can also help you find resources or programs that might not be easily accessible otherwise. The Alzheimer’s Association has several **support groups for Spanish speakers**, and many local organizations may have support groups in Spanish as well.

- **ALZConnected®**: An online forum where you can find other caregivers and share information on local resources that may offer Spanish-speaking support.

5. Local Hispanic Organizations

Consider contacting local Hispanic community organizations that provide services to seniors. These organizations may offer information on affordable daycare or other resources.

- **National Hispanic Council on Aging (NHCOA)**: They may offer connections to programs and services for seniors in the Spanish-speaking community. You can visit their website NHCOA for more details.

It's great that you're proactive in seeking help for your mother. I recommend starting by reaching out to local agencies and organizations dedicated to elderly care and memory issues—they can guide you in finding the right resources.	**How to Start:**<br><br>1. **Contact the Alzheimer's Association Helpline** at **800.272.3900** for assistance in finding programs that are culturally competent and Spanish-speaking.<br>2. **Search the Community Resource Finder** on the Alzheimer's Association website to find local resources in your area.<br>3. **Reach out to local community organizations** like those serving the Latino community (e.g., the Mexican Consulate or other cultural centers).<br>4. **Inquire about Medicare Advantage** plans and see if they offer benefits that include adult day care or respite care.<br><br>If you need more information or help navigating these resources, feel free to reach out!

Domain 2 – Social Support

1. My mother, who previously spoke English fluently, now primarily communicates in Vietnamese. We are looking for an adult day center that offers culturally and language-specific services, including Vietnamese cuisine and staff who can speak Vietnamese with her.

<table>
<tr><th>Cond1</th><th>Cond2</th></tr>
<tr><td>Finding a culturally specific adult day center for your mother is an excellent way to ensure she feels comfortable, understood, and well cared for. Here's how you can approach this:

Steps to Find a Vietnamese-Specific Adult Day Center:
1. Local Area Research
o Use keywords like "Vietnamese adult day care near me" or "Vietnamese senior services [your city]" to start your search.
o Check local directories or community organizations that serve Vietnamese populations.
2. Contact Local Resources
o Area Agency on Aging (AAA): Many cities have AAAs that can provide tailored recommendations for adult day services.
o Vietnamese Cultural Associations or Churches: These organizations often collaborate with service providers catering to the Vietnamese community.
o Community Health Clinics: They may have information about culturally tailored services.
3. Evaluate Centers
o Ask if the center offers Vietnamese cuisine, has Vietnamese-speaking staff, and provides activities familiar to Vietnamese culture.
o Request a tour or attend an open house to observe the environment and ensure it aligns with your mother's needs.
4. Online Resources
Websites like Care.com, Eldercare Locator, or AARP's Community Resource Finder often have detailed listings of adult day centers with filters for cultural and linguistic preferences.
5. Word of Mouth
Connect with Vietnamese community groups or local Facebook groups for recommendations. Personal experiences can guide you to the best options.</td><td>I understand that you're seeking an adult day center that offers culturally and language-specific services, especially with staff who can speak Vietnamese and provide Vietnamese cuisine. Here are a few steps you can take to find the right facility for your mother:

1. Search for Vietnamese-speaking Day Centers: You can start by reaching out to local Alzheimer's and dementia organizations, such as the Alzheimer's Association, to inquire about adult day centers that cater to Vietnamese-speaking individuals. They may have a directory of resources that offer services tailored to specific cultural needs.

2. Contact Local Vietnamese Community Centers: Many Vietnamese communities in larger cities have resources for aging adults. Vietnamese community centers or senior centers may provide day programs for elderly individuals and have bilingual staff available.

3. Online Resources and Directories: Use the Alzheimer's Association's Community Resource Finder tool (https://www.communityresourcefinder.org) to search for adult day centers. You can filter by language, such as Vietnamese, and see if there are any centers nearby that meet your needs.

4. Speak with Healthcare Providers: Sometimes, healthcare providers, especially those involved in Alzheimer's care, may have recommendations for culturally sensitive care options in your area.

5. Inquire About Cultural Activities: When you contact adult day centers, ask about their approach to culturally appropriate activities, including meal options (like Vietnamese cuisine) and staff who can communicate in your mother's preferred language. Some centers may also organize cultural events and social activities that align with your family's values.</td></tr>
</table>

If you’d like assistance narrowing down a search, feel free to share your location, and I can help locate some resources for you!	6. **Consider Transportation and Location**: Make sure the center is accessible and that transportation options are available, especially if you don’t have a reliable means of getting her there.<br><br>Would you like me to help search for some specific centers in your area or provide any additional resources?

2. Could you provide a list of reputable adult day centers near my mother's home? Additionally, I would like to know if these centers offer transportation services.

<table>
<tr><th>Cond1</th><th>Cond2</th></tr>
<tr><td>
To help you find adult day centers near your mother's home and learn whether they offer transportation services, I will need to know your location or the city your mother lives in. Could you provide that information?

USA, Fairfax, VA22032

Here are some reputable adult day centers near Fairfax, VA 22032, with information on their transportation services where available:

1. Fairfax Adult Day Health Care Center
○ Location: 4414 Stringfellow Rd, Chantilly, VA 20151
○ Transportation: They offer transportation services within the local area, but it’s best to confirm details directly with the center.
2. Arden Courts of Fair Oaks
○ Location: 12469 Lee Jackson Memorial Hwy, Fairfax, VA 22033
○ Transportation: Arden Courts generally provides transportation services for local residents, but you should check with them for specifics on pickup and drop-off.
3. Mason Senior Center
○ Location: 4100 Chain Bridge Rd, Fairfax, VA 22030
○ Transportation: They offer transportation services for local seniors, including to and from the center.
4. Green Spring Village Adult Day Services
○ Location: 7470 Spring Village Dr, Springfield, VA 22150
○ Transportation: This center offers transportation services, but confirming the details regarding specific areas served would be helpful.
5. Inova Adult Day Health Services
○ Location: 6430 General Green Way, Alexandria, VA 22312
○ Transportation: Inova provides transportation services for seniors, but you should reach out directly for more details about your mother’s specific location.
</td><td>
To assist you in finding reputable adult day centers near your mother's home, I'll need to know your location or the specific area where you're seeking services. Could you provide the city or region where your mother resides? Additionally, I can help check if these centers offer transportation services, but specific details may require calling the centers directly or visiting their websites.

Once I have that information, I can help guide you toward finding the best options for your mother.

USA, Fairfax, VA, 22032

Here are a few adult day centers near Fairfax, VA that might be able to meet your needs for culturally specific services, including potentially Vietnamese-language support:

1. Fairfax County Adult Day Health Care
○ Located in Herndon, Lewinsville, Lincolnia, and Mount Vernon. These centers offer medical care and daily activities for individuals with Alzheimer’s. It's best to contact them directly for specific language accommodations and transportation availability. You can reach them at 571-585-2790 or via email at adultday@fairfaxcounty.gov

Fairfax County

.

2. InovaCares for Seniors - PACE
○ Located at 4315 Chain Bridge Road, Fairfax, VA 22030, this program provides comprehensive care for seniors with complex medical and social needs, and it might be a good resource for transportation and language needs. For more details, you can contact them directly at 703-750-3000

Senior Care Resources

.
</td></tr>
</table>

I recommend calling these centers directly to inquire about the specifics of their transportation services, including pickup areas and scheduling.	3. **Braddock Glen**<br>○ Located at 4027 Olley Lane, Fairfax, VA 22032, Braddock Glen provides a range of memory care services and might be able to assist with dementia-related needs. Call for more details on their specific offerings<br><br>Senior Care Resources<br><br>.<br><br>You can also explore the **Neighborhood and Community Services (Fairfax County)**, which offers transportation services and has adult day programs that may include specialized care<br><br>Fairfax County<br>.<br><br>I recommend reaching out to these centers directly to inquire about language-specific services, including Vietnamese support, and to confirm transportation options.

Searched 5 sites ^

"adult day centers in Fairfax VA 22032 offering Vietnamese services transportation"
bing.com

Fairfax County — Virginia Wellness Center for Older Adults - ServiceSource
servicesource.org

Fairfax County — Adult Day Health Care | Neighborhood and Community Services
fairfaxcounty.gov

ServiceSource — Braddock Glen Adult Day Health Care Center in Fairfax, VA
seniorcare.com

SeniorCare — Adult Day Health Care Locations | Neighborhood and Community Services
fairfaxcounty.gov

Senior Care Resources — The 10 Best Adult Day Care Facilities in Fairfax, VA
caring.com

3 I'm worried about my wife because she seems different, and her condition resembles what people refer to as dementia. I would like to receive formal training on dementia. Where should I start?

**Cond1**	**Cond2**
I'm sorry to hear about your concerns regarding your wife. Dementia is a serious condition, and it's important to approach it with the right knowledge and support. There are several ways to start formal training on dementia:<br><br>1. **Alzheimer's Association** – They offer comprehensive resources, including online courses and webinars. You can find both educational materials for caregivers and professional training programs on dementia and Alzheimer's disease. Check their website for more details: Alzheimer's Association Training.<br>2. **National Institute on Aging (NIA)** – The NIA provides a variety of resources on aging and dementia. They also offer publications and information about cognitive decline and how to manage symptoms. Visit: NIA Dementia Resources.<br>3. **Caring for a Loved One with Dementia: A Guide for Caregivers** – This is a training program often offered by universities or local healthcare centers. It's designed for family members of those with dementia, covering topics like symptoms, caregiving strategies, and coping mechanisms.<br>4. **Online Platforms** – Websites like Coursera or edX often offer free or affordable courses on dementia and related conditions. For example, "The Dementia Care: Getting Started with Training" course on Coursera might be a good option.<br>5. **Local Support Groups and Healthcare Providers** – Many healthcare providers offer training sessions and support groups for caregivers. These sessions can help you understand the symptoms and strategies for managing daily life. Check with local hospitals or clinics for recommendations.<br>6. **Books and Resources** – Reading books written by experts in the field can also provide valuable insights. Some popular titles include *The 36-Hour Day* by Nancy L. Mace and Peter V. Rabins, which is a well-regarded resource for caregivers.<br><br>Starting with a combination of these resources will give you a solid foundation in dementia care, which	It's great that you're looking for formal training to better understand dementia. There are several resources and courses that can help you gain a deeper understanding of dementia and how to care for someone with it:<br><br>1. **Alzheimer's Association**: The Alzheimer's Association offers caregiver education and training sessions, including workshops, webinars, and local support groups. Their resources cover a variety of topics related to dementia care. You can find more information and register for sessions on their website.<br><br>2. **National Institute on Aging (NIA)**: The NIA provides various educational materials for caregivers, including training on dementia and Alzheimer's disease. They also have research-based recommendations for caregiving practices. You can explore their resources here.<br><br>3. **Caring for a Person with Dementia**: The Family Caregiver Alliance offers online caregiver education programs, including dementia-specific training. They cover aspects such as managing daily care, understanding dementia, and navigating the healthcare system. Learn more here.<br><br>4. **Teepa Snow's Positive Approach to Care**: Teepa Snow is a well-known expert in dementia care, and her Positive Approach to Care training is available online. This method helps caregivers understand the person with dementia's perspective and adapt care approaches accordingly. More details can be found here.<br><br>You can also reach out to your local Alzheimer's Association chapter in Northern Virginia for more information on training opportunities in your area.

could help you understand your wife's condition better and support her through the changes	

4. As a caregiver in Virginia, I'm looking for local resources or organizations that can provide support for caregivers like me. What options are available to help me navigate this journey?

Cond1	Cond2
There are several resources and organizations in Virginia that provide support for caregivers. Here are some options:<br><br>**1. Virginia's Caregiver Support Program (CSP)**<br><br>● **Website**: Virginia's Caregiver Support Program<br>● **Services**: Offers caregiver education, training, and financial support in the form of grants for respite care and other services to reduce caregiver stress. They also provide information on services for family caregivers of older adults.<br><br>**2. Virginia Family Caregiver Support Program (VFCSP)**<br><br>● **Website**: VFCSP<br>● **Services**: This program provides support services such as respite care, training, and information to caregivers of older adults, helping them maintain their own health while caregiving.<br><br>**3. Aging and Disability Resource Centers (ADRCs)**<br><br>● **Website**: ADRC Locator<br>● **Services**: ADRCs provide information and referral services for caregivers and individuals with disabilities or aging issues, connecting you to resources that can help with caregiving responsibilities.<br><br>**4. Alzheimer's Association - Central and Western Virginia Chapter**<br><br>● **Website**: Alzheimer's Association<br>● **Services**: Provides support, educational materials, and respite care specifically for caregivers of individuals with Alzheimer's and other dementias.<br><br>**5. Caregiver Action Network (CAN)**<br><br>● **Website**: Caregiver Action Network<br>● **Services**: While national in scope, CAN offers various resources, tips, and community	As a caregiver in Virginia, there are several organizations and resources that can provide support to help you navigate the challenges of caregiving, especially when caring for a loved one with Alzheimer's or dementia:<br><br>1. **Alzheimer's Association – Virginia Chapter**: The Alzheimer's Association offers a wide range of resources and support for caregivers, including educational programs, support groups, and a 24/7 helpline (800.272.3900). Their website also features useful resources like care planning tools and information on local events. You can access more information specific to Virginia through the Alzheimer's Association Virginia Chapter.<br><br>2. **Virginia Department for Aging and Rehabilitative Services (DARS)**: DARS provides various services to support caregivers in Virginia, including respite care, information on local services, and assistance with navigating long-term care options. Their website offers a caregiver handbook and links to services across the state. More information is available at DARS Virginia.<br><br>3. **Caregiver Resource Center (CRC)**: The CRC provides free information, counseling, and assistance for caregivers, including support groups, educational sessions, and resources about Alzheimer's and other dementias. You can visit their site for more details at Caregiver Resource Center.<br><br>4. **Virginia Caregiver Coalition (VCC)**: The VCC offers resources, training, and advocacy for caregivers across Virginia. They provide valuable support to caregivers, helping them understand their rights, access caregiving tools, and manage the emotional and practical aspects of caregiving. Visit Virginia Caregiver Coalition for further details.<br><br>5. **AARP Virginia**: AARP offers support and information for family caregivers, including guides on navigating long-term care, financial planning, and handling stress. Their website features helpful articles and tools to manage

forums where caregivers can connect and share experiences.<br><br>**6. SeniorNavigator**<br><br>- **Website**: SeniorNavigator<br>- **Services**: A resource guide that provides a comprehensive directory of services, events, and programs throughout Virginia for seniors and their caregivers.<br><br>**7. Respite Care Services**<br><br>- Many local area agencies on aging or community organizations offer respite care services, allowing caregivers to take a break while ensuring their loved one is properly cared for. Check with your local agency on aging for available services.<br><br>Additionally, there are numerous support groups, training programs, and online forums tailored to caregivers in Virginia. Reaching out to these organizations can connect you with local networks, help reduce stress, and provide essential support.	caregiving responsibilities. You can explore their resources at AARP Virginia Caregiving.<br><br>6. **Area Agencies on Aging (AAA)**: Virginia has a network of AAAs that can connect you with local caregiving services, including respite care, transportation assistance, and home care. Find your local AAA at Virginia AAA Locator.<br><br>These resources provide invaluable guidance and help you take care of yourself while caring for your spouse. It's important to utilize them for both practical support and emotional well-being.

5. I’m looking to connect with other caregivers in my community for support. Are there any peer support groups or opportunities to meet others who are going through similar experiences? Can you suggest any online support groups or forums for caregivers?

**Cond1**	**Cond2**
It's great that you're looking to connect with others for support! Here are some suggestions for both local and online opportunities to connect with fellow caregivers:<br>In-Person Support Groups (Community-Based)<br>1. Local Hospitals or Healthcare Providers: Many hospitals and health centers host caregiver support groups. You could ask your doctor or local health services if they know of any in-person gatherings or workshops.<br>2. Area Agencies on Aging (AAA): These agencies often have caregiver programs and might host regular meetups or events.<br>3. Nonprofit Organizations: Organizations like the Caregiver Action Network or the Alzheimer's Association often have local chapters that organize support groups.<br>4. Faith-Based Groups: Churches, synagogues, mosques, or other faith communities may offer caregiver support groups or prayer circles for caregivers.<br>5. Community Centers or Libraries: These venues often host support groups, especially those aimed at mental health or caregiving.<br>Online Support Groups and Forums<br>1. Caregiver Action Network (CAN): This nonprofit offers a wide range of resources for caregivers, including an online support community where you can connect with others through discussion boards.<br>2. AARP Caregiving Community: AARP has a large, active online community where caregivers can share stories, advice, and encouragement.<br>3. Reddit’s Caregiver Community: Subreddits like r/Caregivers offer a place for caregivers to discuss their experiences and share resources.<br>4. Facebook Groups: There are many private Facebook groups for caregivers, such as "Caregivers Support Group" or "Caregiver Support Network." These groups are often private to ensure confidentiality and emotional safety.<br>5. MyCaringPlan: This online community offers resources and peer support for caregivers. It's great for those looking for a mix of practical tips and emotional support.<br>6. National Alliance for Caregiving (NAC): They offer some digital resources, including support networks, and help connect caregivers to one another.	Connecting with other caregivers for support is an important part of managing the challenges you may face. There are several ways to find peer support groups and opportunities to meet others in your community, as well as online forums where you can connect with caregivers who share similar experiences.<br><br>**Local Support Groups in Virginia:**<br><br>1. **Alzheimer's Association Virginia Chapter**: The Alzheimer’s Association offers in-person and virtual support groups for caregivers. These groups are led by trained facilitators and offer a safe space to share experiences and receive emotional support. You can find local support groups in Virginia at their Support Groups page.<br><br>2. **Caregiver Resource Center (CRC)**: The CRC provides resources for caregivers, including opportunities to join peer support groups. They host both in-person and virtual caregiver groups. Visit their Caregiver Support page for more information on finding local groups.<br><br>3. **Area Agencies on Aging (AAA)**: Each region in Virginia has an Area Agency on Aging that can connect you to local caregiver support groups. You can locate your local AAA at Virginia AAA Locator.<br><br>4. **Senior Services of Alexandria**: They offer caregiver programs that include support groups and educational resources. Find more at Senior Services of Alexandria.<br><br>**Online Support Groups and Forums:**<br><br>1. **AlzConnected**: An online community offered by the Alzheimer’s Association where caregivers can connect with others, share advice, and discuss their experiences in a safe environment. Visit AlzConnected for more information.<br><br>2. **AARP’s Caregiving Community**: AARP provides an online platform where caregivers can join forums, ask questions, and connect

7. CaringBridge: While primarily a platform for sharing health updates, CaringBridge also has caregiver support groups where you can find community and emotional support.<br>8. Wellness Support Websites (e.g., Care.com or EmpowerCaregiving.com): These sites have forums and resources for caregivers, offering peer support, tips, and advice.<br>Would you like me to help you find specific groups in your area or assist with signing up for any of these online platforms?	with others. Their resources and support forums can be found at AARP Caregiving.<br><br>3. **The Caregiver Action Network (CAN)**: CAN offers a variety of online tools, including an online forum where caregivers can share stories, find advice, and receive support. Check out Caregiver Action Network for more details.<br><br>4. **Reddit – Dementia Caregivers Community**: The subreddit r/DementiaCaregivers is a community where people share their caregiving experiences, provide emotional support, and offer practical advice. It's a great way to engage with others online. Visit r/DementiaCaregivers to join the conversation.<br><br>**Virtual Peer Support:**<br><br>● Many virtual caregiver support groups are available, particularly through organizations like the Alzheimer's Association and AARP, which can connect you with peers who are managing similar challenges from the comfort of your home.<br><br>By connecting with both local and online support groups, you can gain insights, share your own experiences, and find emotional support from people who understand the unique challenges of caregiving.

6. I'm looking for social activities for my loved one who is in the early stages of dementia (MCI). Are there Spanish-language resources or activities suitable for individuals with MCI that also respect our cultural traditions and values?

**Cond1**	**Cond2**
It's wonderful that you're looking for meaningful activities for your loved one with Mild Cognitive Impairment (MCI), and cultural sensitivity is important in enhancing the experience. Here are some suggestions for Spanish-language resources and culturally respectful activities:<br>1. Cognitive and Social Stimulation<br>● Memory Games and Puzzles in Spanish: Engage in simple memory exercises, like matching games or jigsaw puzzles. Look for resources or apps designed specifically in Spanish that focus on cognitive stimulation, such as Lumosity or Peak, which offer language options.<br>● Storytelling or Reading: Reading books, stories, or poems in Spanish can encourage recall and stimulate conversation. You can choose works by authors like Gabriel García Márquez or Isabel Allende, or opt for traditional folktales and stories that are culturally significant.<br>2. Music and Dance<br>● Music Therapy: Music plays a powerful role in engaging people with dementia. Playing traditional Spanish or Latin American music (e.g., salsa, flamenco, boleros, or ranchera) can create a familiar and comforting environment. Singing along or dancing to these songs can also help with memory recall.<br>● Dancing: If mobility allows, simple dances like the merengue, bachata, or tango can be enjoyable and culturally engaging.<br>3. Art and Craft<br>● Cultural Craft Activities: Engaging in simple crafts that reflect your culture (such as making piñatas, creating collages with images of traditional foods, festivals, or nature, or even pottery or painting) can spark memories and creativity.<br>● Traditional Recipes: Cooking or even just talking about favorite family recipes in Spanish can trigger pleasant memories. Preparing dishes like tacos, empanadas,	Finding social activities that are both culturally relevant and suitable for individuals with Mild Cognitive Impairment (MCI) in Spanish can greatly enhance your loved one's sense of identity, belonging, and overall well-being. Here are some resources and suggestions to help you identify these types of activities:<br><br>**1. Alzheimer's Association - Spanish-Speaking Resources**<br><br>The **Alzheimer's Association** provides Spanish-language resources that cater specifically to individuals with Alzheimer's or dementia. They offer information on activities that respect cultural values while engaging people with MCI. Some suggestions for activities include:<br><br>● **Music therapy**: Music can be particularly effective in engaging people with MCI, especially songs that are familiar to them from their cultural background.<br><br>● **Art programs**: Simple creative activities such as painting, drawing, or crafting can stimulate cognitive function while also tapping into personal memories and emotions.<br><br>Check out their **Spanish-language section** for caregiver tips and activities: Alzheimer's Association in Spanish.<br><br>**2. Local Day Centers with Cultural Focus**<br><br>Many adult day centers offer culturally relevant programming, including Spanish-language activities for people with dementia. In Virginia, you may find such centers that tailor their activities to respect both language preferences and cultural traditions. For example:<br><br>● **Spanish-speaking staff**: Some centers have Spanish-speaking caregivers who can engage your loved one in meaningful conversations and activities.<br><br>● **Cultural events**: Look for centers that celebrate Hispanic holidays, offer cooking classes featuring traditional Spanish or Latin American dishes, and host music or dance sessions related to Hispanic cultures.<br><br>You can search for local options using resources like the **Alzheimer's Association** and **AgingCare** to find culturally appropriate day centers in your area.<br><br>**3. Senior Centers with Spanish-Language Programming**

or paella together can be both fun and a great bonding experience.<br>4. Social Groups and Support Networks<br>● Spanish-language Dementia Support Groups: Many communities have dementia or caregiver support groups in Spanish. These provide a space to share experiences and advice. Look for local organizations, such as Alzheimer's Association chapters, which may offer groups for Spanish-speaking families.<br>● Community Centers and Churches: Many local cultural or religious institutions (like Latino community centers or churches) often host events, support groups, or social gatherings specifically for Spanish-speaking seniors, which may include activities like bingo, storytelling, or traditional music nights.<br>5. Therapeutic Activities<br>● Pet Therapy or Animal Visits: Many people with dementia find comfort in interacting with pets. Consider Spanish-language pet therapy programs if available, where trained animals visit people in care facilities or homes.<br>● Gardening: If possible, gardening can be a calming and engaging activity. Growing plants or flowers common in your culture, such as cilantro, tomatoes, or marigolds, can evoke positive memories.<br>6. Technology Resources<br>● Virtual Visits and Apps: Apps like Alzheimer's Spanish (an app providing cognitive training in Spanish) or MyCognition offer brain training exercises in Spanish that are simple and fun.<br>● Video Calls with Family: Encourage family to stay connected through video calls in Spanish, where they can talk, reminisce, or share stories. Familiar voices can be comforting.<br>7. Spiritual and Religious Activities<br>● Daily Devotions or Prayer: For those who value spirituality, reading la Biblia (the Bible) or participating in daily devotions in Spanish can be comforting and meaningful. Many families also participate in rosarios or prayers in their native language to provide structure and connection.	Many senior centers have programs designed for Spanish-speaking older adults. These programs often include:<br>● **Exercise classes** (like Zumba or yoga, tailored for seniors)<br>● **Language-based social activities**: Spanish-language movie nights, group discussions, and storytelling<br>● **Spiritual and cultural activities**: Participation in religious services in Spanish or community gatherings during Hispanic cultural celebrations.<br><br>Contacting local **Hispanic/Latino community centers** in Virginia may also help you find activities that combine cognitive stimulation with cultural traditions. A good place to start would be centers such as:<br>● **Latino Council of Virginia**<br>● **Casa de Maryland**<br><br>**4. Online Activities and Virtual Communities**<br><br>Several websites and apps provide virtual activities in Spanish for individuals with dementia:<br>● **BrainHQ** and **Lumosity** offer brain exercises in Spanish that can help improve cognitive abilities in the early stages of dementia.<br>● **Alzheimer's Spanish Resources**: Online platforms such as Alzheimer's.org and **AlzConnected** provide resources, including social activity ideas, support groups, and forums in Spanish.<br><br>These resources allow your loved one to engage in activities remotely or complement in-person events with online content that respects their language and cultural preferences.<br><br>**5. Spanish-Language Music and Dance Programs**<br><br>Music and movement can be powerful tools to engage individuals with dementia. Look for **music therapy programs** in Spanish that use traditional instruments, songs, and dance that reflect the cultural background. This can help bring comfort, joy, and a sense of connection to your loved one. Many local senior centers, cultural organizations, or even churches may offer Spanish-language dance classes or music therapy sessions.<br><br>By using these resources, you can find activities that engage both your loved one's mind and their cultural heritage, helping maintain their identity and dignity while providing them with meaningful ways to stay active.

By blending cognitive stimulation, socialization, and cultural values, you can create a supportive and enriching environment for your loved one with MCI. If you need more specific resources, such as links to Spanish-language apps or local support groups, I can help further!	

7. My dad doesn't seem to express his feelings, but I'm concerned he may be experiencing isolation or loneliness due to his condition. How can I address these feelings and provide support to help him feel more connected?

**Cond1**	**Cond2**
It sounds like you deeply care about your dad's emotional well-being, and your concern is a great first step in supporting him. Here are a few suggestions to help address his potential feelings of isolation or loneliness:<br>1. **Create a Safe Space for Communication**:<br>o Approach him gently and without pressure. You can start by saying something like, "I've noticed that you seem a bit down lately, and I just want to make sure you're okay. If you ever feel like talking, I'm here to listen."<br>o If he's not comfortable opening up, that's okay. Just letting him know you're available and willing to listen can mean a lot.<br>2. **Engage in Activities Together**:<br>o Sometimes people who are isolated may not express it directly. You could invite him to do activities he enjoys or even try new ones together—whether it's a hobby, going for walks, or watching a movie. Spending quality time can subtly help combat loneliness.<br>o Involving him in simple tasks, such as cooking together or even small home repairs, can create opportunities for connection.<br>3. **Encourage Socialization, If Possible**:<br>o If his condition allows, encourage social interaction with friends, family, or community groups. Help him reconnect with old friends, or consider finding clubs or activities he might enjoy, like local classes or support groups.<br>o Be mindful of his comfort level. If he's hesitant, start small, such as inviting one person over for a short visit.<br>4. **Check for Underlying Emotional or Physical Issues**:<br>o Sometimes isolation can stem from physical or emotional difficulties, such as mobility issues, chronic pain, or depression. Gently suggest that he talk to a doctor or therapist if you think these factors may be contributing to his feelings of isolation.<br>5. **Focus on Non-Verbal Support**:	If you're concerned that your dad might be feeling isolated or lonely, especially if he has difficulty expressing these feelings, there are several steps you can take to help him feel more connected:<br><br>**1. Create Regular Social Engagement**<br><br>● **Consistent Visits**: Regular visits from family or friends can provide important emotional support and help break the cycle of loneliness. Even if your dad is not expressing emotions verbally, your presence and efforts to spend quality time with him can be very comforting.<br>● **Group Activities**: Engage your dad in social settings such as community activities or family gatherings, even if he doesn't actively participate. The presence of others and the sense of being part of a group can reduce feelings of isolation.<br><br>**2. Encourage Communication through Non-Verbal Means**<br><br>● **Music Therapy**: Music has been shown to help those with dementia or Alzheimer's express themselves, even if words are difficult. Listening to music, singing, or even dancing can be an excellent way to connect emotionally with your dad.<br>● **Art Therapy**: Encourage creative activities like drawing or painting, which can allow him to express feelings that are hard to articulate. Art can be a meaningful way for him to process emotions without needing to speak.<br><br>**3. Focus on Familiar Routines**<br><br>● Maintaining familiar routines can help your dad feel more secure and connected to his environment. Try to keep meal times, social events, and hobbies as consistent as possible, which can also provide opportunities for gentle social interactions.<br><br>**4. Provide Emotional Reassurance**<br><br>● Even if your dad is not openly expressing his feelings, regularly offering reassurance through simple, comforting statements can help him feel connected. Gentle touches, such

- If your dad struggles to express his feelings verbally, non-verbal forms of support, like writing a letter, creating a photo album, or offering small tokens of affection, might help bridge the gap. This shows you understand his emotional needs without overwhelming him.

6. **Promote a Healthy Routine**:
   - Encourage daily routines that include self-care, exercise, and activities he enjoys. Staying physically active can help improve mood and provide natural opportunities for socializing.
7. **Respect His Boundaries**:
   - While it's important to offer support, it's equally important to respect his boundaries. Some people may not feel ready to open up, and pressuring him could make him retreat further.

Above all, your love and presence are invaluable, and by offering consistent emotional support, you'll help him feel more connected, even if it takes time.

as holding hands, and verbal affirmations like "I'm here for you" can reduce feelings of loneliness.

5. Join Support Groups

- If your dad is open to socializing or you feel that it could help him, joining a support group can provide an outlet for both of you. There are many groups for caregivers and those living with dementia, where you can share experiences and strategies. These groups can be a source of emotional support and help your dad feel less isolated.
- **Virtual Support**: If in-person meetings are difficult, online support groups for dementia caregivers can offer emotional connections. Platforms such as **AlzConnected** provide forums where people can discuss challenges and find solidarity in their experiences.

6. Professional Support

- If loneliness persists or worsens, consider seeking the advice of a professional therapist, especially one who specializes in dementia or elder care. They may be able to help both you and your dad cope with emotions that are difficult to express, even when words are not involved.

Resources:

- **Alzheimer's Association** offers useful guides and support for dementia patients and their families. For information on support and activities, you can check out their **local chapters** and **caregiver resources** here.
- **Family Caregiver Alliance** provides information on how to address isolation and loneliness in elderly individuals with Alzheimer's and other cognitive disorders here.

By taking steps to address emotional isolation in a way that respects your dad's needs and preferences, you can help ensure that he feels more supported and connected, even if he isn't able to express it openly.

Domain 3 – Dementia Literacy

1. My mother appears to be fine, but she often frustrates me by repeatedly asking the same questions. Recently, she claimed she lost her purse and accused a neighbor of taking it, which is not true. What steps should I take to help my mom?

<table>
<tr><th>Cond1</th><th>Cond2</th></tr>
<tr><td>It sounds like your mother may be experiencing some cognitive challenges, which can be frustrating for both her and you. Repeated questions and misplaced items, like her purse, can sometimes be symptoms of memory issues or conditions like mild cognitive impairment or dementia. Here are some steps you can take to help:
1. Observe and Document: Keep track of her behavior to see if these issues are becoming more frequent or severe. Note any changes in memory, behavior, or mood. This can be helpful when discussing her condition with a healthcare professional.
2. Talk to a Doctor: If you haven't already, it may be time for her to see a healthcare provider for a full assessment. A doctor can evaluate her cognitive function, rule out medical conditions (like vitamin deficiencies, infections, or thyroid problems), and provide a diagnosis or recommend a treatment plan.
3. Stay Calm and Patient: When she repeats questions, try to stay calm and avoid showing frustration. Gently remind her of the answer, and offer reassurance. Repeating yourself without frustration can help her feel more comfortable and less anxious.
4. Create a Routine: Having a consistent daily routine can help reduce confusion. Regular activities and a predictable environment can help your mom feel more grounded.
5. Memory Aids: Encourage the use of memory aids, such as a calendar or a journal. Keep important items, like her purse, in the same place, and consider labeling things around the house for easier identification.
6. Be Compassionate About the Accusations: If she accuses someone, like the neighbor, of taking her purse, it's likely due to confusion or fear, not intentional distrust. You can gently redirect the conversation, and if she continues to insist, validate her feelings without getting into a confrontation. You might say, "I understand you're upset. Let's look for it together."
7. Consider Supportive Services: If the situation progresses, it may be helpful to look into supportive services like adult day care, therapy, or a caregiver to provide additional help.</td><td>It sounds like your mother may be experiencing some cognitive changes, which can sometimes be a sign of early-stage dementia or Alzheimer's, though it's important not to jump to conclusions without a proper evaluation. Here are some steps you can take to help her:
1. Remain Calm and Patient:
o Repetition of questions and accusations are common in people with cognitive issues, including dementia. It's important to stay patient, as these behaviors are typically not intentional.
o When she repeats questions, answer them simply and consistently. Avoid getting frustrated or correcting her, as this may cause additional distress.
2. Validate Her Feelings:
o When she accuses someone of taking the purse, instead of arguing, try to validate her feelings. You could say something like, "I understand that you're upset about the purse, but I can help you find it. Let's look together."
3. Try to Investigate the Cause:
o Repeated questions could be a sign of anxiety or uncertainty, while accusations of theft may be linked to memory issues or confusion. It's possible she misplaces her items and becomes anxious, which can lead to accusations.
4. Create a Safe, Organized Environment:</td></tr>
</table>

8. Take Care of Yourself: Caring for someone with cognitive issues can be emotionally draining. Make sure you're seeking support when needed and giving yourself time to recharge.<br>By taking a proactive approach, you can help ensure her well-being while also maintaining a supportive environment for both of you.	○ Keep her living space organized and have designated spots for important items like her purse, keys, etc., which may reduce confusion and anxiety.<br>○ If you can, label drawers or areas where personal items are kept.<br>5. **Consider a Medical Evaluation:**<br>○ If the repetition and accusations continue or worsen, it may be a good idea to consult her doctor. A healthcare provider can assess her cognitive function and determine if there are underlying conditions like dementia or other issues.<br>6. **Use Calming Techniques:**<br>○ If your mother is upset about the situation, offering a calming activity like a favorite hobby, music, or even a gentle walk may help distract her and reduce anxiety.<br>7. **Take Care of Yourself:**<br>○ Caring for someone who is experiencing cognitive challenges can be emotionally taxing. Make sure you're taking time for your own self-care, seeking support when necessary, and considering caregiver resources available to you.<br>If you’re concerned about her overall cognitive health, a conversation with her healthcare provider can help guide next steps.

2 The other day, my husband got lost on his way home. Could this be an early sign of dementia? What steps should I take to address this?

**Cond1**	**Cond2**
Getting lost, especially when it hasn't happened before, can indeed be a concerning sign, but it's not necessarily an early indicator of dementia. However, it could suggest that your husband is experiencing some cognitive changes, which may warrant closer attention. Dementia can affect memory, spatial awareness, and the ability to navigate familiar environments, but many factors can also contribute to this behavior, such as stress, medication side effects, fatigue, or even changes in routine.<br><br>**Here are some steps you can take to address this situation:**<br>1. Observe for Other Symptoms: Pay attention to whether he is also experiencing memory issues, confusion, difficulty finding the right words, trouble with tasks that require planning or organization, or mood changes. These could be signs of cognitive decline or dementia.<br>2. Consult a Doctor: It's important to have him evaluated by a healthcare professional, especially if you notice other signs of cognitive impairment. A doctor can assess whether this behavior might be due to dementia, other medical conditions, or age-related changes. They might recommend cognitive tests or even brain imaging.<br>3. Consider Safety Measures: If he continues to get lost or show confusion, consider strategies to ensure his safety. This might include having a medical alert system, using GPS tracking devices, or modifying his routine to minimize confusion.<br>4. Review Medications: Sometimes medications can cause side effects that affect memory and cognitive function. Check with his healthcare provider to see if any of his medications could be contributing to this issue.<br>5. Encourage a Healthy Lifestyle: Encourage him to maintain a healthy lifestyle, including a balanced diet, regular physical activity, and mental exercises. These can support cognitive health.<br>6. Start a Conversation: Have an open and supportive conversation with your husband about your concerns. It's important that he feels involved in the process of understanding what might be going on.<br>If you feel unsure about any aspect, seeking support from a healthcare professional is the best next step.	Yes, getting lost, even on familiar routes, can be an early sign of dementia, especially if your husband had no previous issues with navigating or remembering his way home. This kind of disorientation can occur as dementia or Alzheimer's disease begins to affect memory, spatial awareness, and cognitive function.<br><br>Here are some steps you can take to address this situation:<br><br>**1. Monitor for Other Signs:**<br>● In addition to getting lost, look for other signs of memory loss or confusion, such as forgetting appointments, repeating questions, struggling with familiar tasks, or changes in behavior or mood.<br>● If you notice a pattern, it may be time to consult a doctor for a thorough evaluation.<br><br>**2. Visit a Healthcare Provider:**<br>● Make an appointment with your husband's healthcare provider to discuss this incident and any other concerning changes. The doctor may recommend cognitive testing or refer you to a specialist, such as a neurologist, for further assessment.<br>● Early diagnosis can help with planning and accessing treatments or therapies that can improve quality of life and slow the progression of dementia.<br><br>**3. Improve Safety Measures:**<br>● **Driving Safety:** If your husband is still driving, consider discussing his driving abilities with his doctor. They can help assess whether it is safe for him to continue driving. Dementia can impair judgment, reaction time, and memory, making driving dangerous.<br>● **Home Safety:** Make sure the home environment is safe and navigable. You may want to implement simple safety

They can guide you on the most appropriate course of action.	measures, such as labeling rooms or adding signage to help with orientation.<br><br>**4. Create a Routine:**<br><br>● People with early-stage dementia often do well with routine and structure. You can help your husband by establishing a daily routine that includes regular outings or familiar activities, which can provide a sense of security and reduce confusion.<br><br>**5. Consider GPS Tracking:**<br><br>● If your husband continues to have difficulty navigating, you might want to consider using a GPS tracking device or an app on his phone that can help you track his location. Some wearable devices designed for individuals with memory problems also have GPS tracking capabilities.<br><br>**6. Stay Calm and Supportive:**<br><br>● Experiencing disorientation can be frightening for both your husband and you. It's important to be patient, avoid placing blame, and offer reassurance. Make sure he knows you're there to support him.<br><br>**7. Consider Support and Education:**<br><br>● Caring for someone with dementia can be challenging, and it may be helpful to connect with support groups or caregivers' resources. You can learn from others who are going through similar experiences and gain valuable advice.<br><br>For further guidance, you might want to reach out to the Alzheimer's Association or a local support group. They can offer resources and assistance specific to your situation.<br><br>Let me know if you need any more information or support.

3. I'm looking for suitable social activities for my mother, who is in the early stages of dementia. Are there any Korean-language resources or activities available that could help her stay engaged? I've also heard there are ways to prevent or delay the progression of the condition—can you recommend anything in that regard?

<table>
<tr><th>Cond1</th><th>Cond2</th></tr>
<tr><td>
It's great that you're looking for ways to keep your mother engaged and support her well-being. Here are some ideas for both social activities and ways to potentially delay the progression of dementia, with a focus on Korean-language resources:

Social Activities for Early-Stage Dementia:
1. Music Therapy: Korean folk songs or popular music from her generation can trigger memories and provide comfort. You might look for local or online music therapy programs, or even play her favorite songs at home.
2. Arts and Crafts: Simple art projects, such as drawing, painting, or crafting, can help maintain cognitive function and stimulate creativity. You can find tutorials or art classes in Korean, either online or through community centers.
3. Group Exercises: Many elderly Koreans enjoy light, gentle exercises like tai chi or dancing. Look for local community groups or senior centers that offer these activities in Korean, which could promote physical and social well-being.
4. Korean Drama or Movie Viewing: Watching familiar, comforting TV shows or films, especially older ones she used to enjoy, can provide both engagement and a sense of continuity. Some online platforms offer Korean-language shows with subtitles to help with comprehension.
5. Memory Games: Playing simple memory games, like matching pairs, can be a fun and stimulating way to keep her mind active. There are memory games available in Korean, both physical and digital.

Korean-Language Resources:
● Korean Alzheimer's Disease Association (KADA): This organization offers resources, caregiver support, and educational materials in Korean. They might have programs tailored to dementia patients.
● Caregiver Support Groups: Many communities have local or online groups for Korean-speaking caregivers. These could be helpful for both you and your mother.

Ways to Delay the Progression of Dementia:
1. Mental Stimulation: Keeping the brain active through puzzles, games, reading, and learning new
</td><td>
It's great that you're looking for ways to keep your mother engaged. There are several social activities and resources that can be beneficial for someone in the early stages of dementia, including:

1. Cultural and Social Activities: You can look for community centers or organizations offering dementia-friendly social programs in the Korean community. In some areas, cultural centers, senior centers, and Alzheimer's support groups offer tailored activities in the Korean language. Check with local Alzheimer's Association chapters or community health organizations for specifics on this.

2. Creative Engagement: Activities like music therapy, art classes, gardening, or simple crafts are great ways for someone with dementia to express themselves. You can also introduce reminiscence activities such as looking through old family photos or playing traditional Korean games, which may help bring back happy memories and stimulate conversation.

3. Physical Activities: Simple physical activities like walking, stretching, or yoga can help maintain mobility, reduce stress, and promote overall well-being. Check if there are senior-friendly exercise programs available in your area.

As for slowing or delaying the progression of dementia, some strategies include:

● Mental Stimulation: Encourage activities that stimulate the mind, such as reading, puzzles, or games that require memory or problem-solving. Even simple tasks that promote cognitive function can help.

● Physical Exercise: Regular exercise is proven to benefit brain health. Activities like walking, swimming, or dancing can improve mood, sleep, and overall physical health.
</td></tr>
</table>

skills can help slow cognitive decline. Activities such as learning new words in Korean or playing word puzzles can help.<br>2. Physical Activity: Regular exercise has been shown to benefit brain health. Activities like walking, stretching, or dancing can improve circulation and cognitive function.<br>3. Healthy Diet: A balanced diet rich in fruits, vegetables, and omega-3 fatty acids (like fish) may help support brain health. In Korea, the Mediterranean diet and traditional Korean dishes like kimchi can contribute to overall well-being.<br>4. Social Interaction: Staying socially active is key. Encourage her to participate in family gatherings, community events, or just regular social outings. Even a daily phone call with a friend or relative can provide cognitive benefits.<br>5. Quality Sleep: Ensure she gets enough rest, as sleep is crucial for cognitive function. You might need to work with a doctor or caregiver to address any sleep disturbances.<br>6. Medication and Medical Support: Consult with a healthcare provider to discuss medications that may help manage symptoms. There are also therapies aimed at slowing the progression of dementia.<br>These activities and strategies are aimed at enhancing her quality of life while potentially slowing the progression of dementia. If you're looking for specific local resources in Korea, or want more recommendations tailored to your mother's interests, I'd be happy to help!	● **Healthy Diet**: A balanced diet rich in fruits, vegetables, lean proteins, and whole grains, along with omega-3 fatty acids found in fish, can support brain health. The Mediterranean diet, in particular, is often recommended for its potential cognitive benefits.<br>● **Social Interaction**: Staying socially active, whether through family, friends, or group activities, helps maintain cognitive and emotional health. It reduces isolation and provides emotional support.<br>I recommend talking to your mother's healthcare provider to create a tailored plan to address her specific needs. Would you like help finding specific resources in your area?

4. My husband’s behavior has been changing lately, and I’m not sure if it’s just because he’s getting older or if it could be something like dementia. He forgets things, gets confused, and sometimes doesn’t act like himself. What are the early signs of dementia, and how can I tell if this is just aging or something more serious?

**Cond1**	**Cond2**
It’s understandable to be concerned when you notice changes in your husband's behavior, especially if they seem out of character. While some degree of memory loss and confusion can be a normal part of aging, significant changes in behavior and cognition can sometimes signal dementia. **Here are some early signs of dementia to look out for:**<br>1. Memory Loss: One of the most common early signs of dementia is difficulty remembering recent events, appointments, or conversations. If your husband repeatedly forgets things he used to remember, or asks the same questions over and over, it may be worth paying attention.<br>2. Difficulty with Familiar Tasks: People with early dementia may struggle with tasks that were once routine or familiar, such as managing finances, cooking, or following a recipe. If he's becoming easily frustrated or confused while trying to complete these tasks, it could indicate a problem.<br>3. Confusion with Time and Place: Losing track of the day, date, or where he is can also be an early sign. If your husband is getting lost in places he knows well, or has trouble recognizing the passage of time, this is worth noting.<br>4. Changes in Judgment or Decision-Making: Dementia can affect the ability to make sound decisions. This might show up as poor financial decisions, neglecting personal hygiene, or making unsafe choices.<br>5. Personality or Mood Changes: People with dementia often experience mood swings, anxiety, depression, or even paranoia. If your husband has been acting unusually irritable, withdrawn, or emotional, this might be a sign.<br>6. Difficulty with Communication: If he is having trouble following or joining in conversations, or forgetting common words, it might indicate cognitive decline.<br>7. Withdrawal from Social Activities: A person with dementia may become less interested in hobbies or activities they once enjoyed. If he seems to be withdrawing socially or avoiding family interactions, this could be a concern.<br>To differentiate between normal aging and something more serious like dementia, it's important to consider the severity and consistency of these changes. Mild forgetfulness or confusion can be a part of aging, but if	It's understandable to be concerned when you notice changes in your husband's behavior, and it can be difficult to distinguish between normal aging and early signs of dementia. While age-related changes in memory and cognition are common, there are certain signs of dementia that differ from typical aging. Here are some early signs of dementia to watch for:<br>1. **Memory Loss**: While occasional forgetfulness is normal with aging, someone with dementia may repeatedly forget recent events or important information (like appointments or conversations) and struggle to retain new information.<br>2. **Difficulty with Familiar Tasks**: People with dementia may have trouble completing everyday tasks they once performed easily, like managing finances, cooking, or remembering how to drive to familiar locations.<br>3. **Confusion with Time and Place**: Forgetting the day of the week or becoming confused about where they are or how they got there can be signs of dementia. They might also lose track of time, forgetting whether it's morning or evening.<br>4. **Challenges with Communication**: Difficulty finding the right words, repeating themselves, or losing their train of thought in conversations is common. They may also struggle to follow or participate in conversations.<br>5. **Changes in Mood and Personality**: Dementia can cause noticeable shifts in mood, such as becoming easily upset, anxious, or depressed. A person may also exhibit unusual behaviors like irritability, aggression, or apathy.<br>6. **Poor Judgment or Decision-Making**: Dementia can impact someone's ability to

the changes are disrupting daily life or becoming more pronounced over time, it could suggest a condition like dementia.

If you're concerned, it's a good idea to talk to a healthcare professional. A doctor can evaluate his cognitive function through tests and determine whether further assessment or interventions are needed. Early diagnosis can help manage symptoms and plan for future care.

make sound judgments. For example, they may show poor judgment with money, hygiene, or safety, such as leaving the stove on or making inappropriate decisions.

7. **Disorientation or Wandering**: A person with dementia may become disoriented and wander, even in familiar places. This can happen particularly if they are confused about where they are.

How to Tell if It's Just Aging or Something More Serious:

- **Duration and Frequency**: If your husband's symptoms are frequent, worsen over time, or involve multiple cognitive areas (memory, thinking, decision-making), it may suggest something more serious, like dementia. Aging typically involves occasional memory lapses without significant impairment.
- **Impact on Daily Life**: If the changes are significantly interfering with his ability to perform daily tasks (personal care, managing finances, getting around), it may indicate dementia.
- **Progression**: Normal aging changes are often gradual and don't impair independence. Dementia symptoms typically progress and can affect a person's ability to live independently over time.

Next Steps:

1. **Consult a Healthcare Provider**: It's important to consult your husband's doctor to discuss the changes you're observing. A physician can conduct a thorough evaluation to assess cognitive function, review medical history, and rule out other conditions (such as depression or medication side effects) that could be contributing to the changes.
2. **Cognitive Testing**: There are specific tests that can help assess memory and thinking skills. A neuropsychological

	evaluation may help determine if dementia is present.<br><br>3. **Maintain a Routine**: If your husband is experiencing cognitive changes, maintaining a routine can help provide structure and reduce confusion.<br><br>4. **Document Symptoms**: Keep track of the changes you're noticing—such as when they occur, their frequency, and how they affect daily activities. This can be helpful when discussing the situation with a healthcare provider.<br><br>If you feel that your husband's behavior is more than just aging, seeking a diagnosis as early as possible can help with planning and accessing appropriate treatments and support. If you need support, the Alzheimer's Association has resources available for caregivers, including a 24/7 helpline (800.272.3900).

5. My husband has been acting differently, and sometimes he gets upset or does things that are hard to understand. How can I handle these behaviors, especially if it's because of dementia?

<table>
<tr><th>Cond1</th><th>Cond2</th></tr>
<tr><td>I'm really sorry to hear that your husband is going through changes in his behavior. It can be challenging to manage, especially when dementia is involved. Here are a few suggestions that might help:

1. Educate Yourself About Dementia: Understanding the symptoms and progression of dementia can give you insight into why your husband might be acting in ways that seem unusual or hard to understand. Different types of dementia can affect memory, mood, and behavior in various ways.
2. Stay Calm and Patient: It can be difficult when your husband gets upset or behaves in ways that are hard to interpret. Try to stay calm and avoid reacting with frustration or anger, as this can make the situation worse. Speak in a calm, reassuring tone and give him time to process information.
3. Communicate Simply and Clearly: People with dementia may have trouble understanding complex sentences or remembering what has been said. Use simple, clear language and short sentences. Avoid asking too many questions at once, and give him time to respond.
4. Provide Consistent Routines: Routine and familiarity can help reduce confusion and anxiety for someone with dementia. Try to keep daily activities and schedules as predictable as possible, and avoid sudden changes in environment or routine.
5. Offer Reassurance and Validation: If your husband becomes upset or agitated, try to validate his feelings without correcting him. Instead of saying "That's not true," you could say, "I understand that you feel that way." This approach can help reduce his frustration.
6. Encourage Engagement: While some days might be harder than others, try to encourage activities that your husband enjoys, like music, gentle exercise, or hobbies he still finds fulfilling. Activities can help improve mood and reduce agitation.
7. Monitor Health and Seek Support: Sometimes behavior changes in dementia can be due to physical health problems like infections or medication side effects. Regular check-ups with his doctor are essential. You may also want to connect with a support</td><td>I'm sorry to hear that your husband is going through these difficult changes. It can be challenging to manage behaviors that result from dementia, but understanding some common strategies can help you provide the support he needs while also protecting your well-being.

Here are some approaches to handle the behaviors you're noticing:

1. Stay Calm and Patient

● Why it helps: People with dementia often experience confusion, anxiety, or frustration, and these emotions may be expressed through behaviors such as agitation, anger, or withdrawal. When this happens, try to remain calm and patient, as your response can have a calming effect.
● What to do: Breathe deeply and avoid reacting with frustration. Speak in a calm, reassuring voice. It can also help to validate his feelings by saying things like, "I understand you're upset, I'm here to help."

2. Use Simple, Clear Communication

● Why it helps: Dementia can affect a person's ability to process information or follow complex conversations.
● What to do: Speak slowly and clearly, using simple words and short sentences. Maintain eye contact and make sure there's minimal distraction (like background noise) when communicating. Give your husband time to process the information and respond.

3. Redirect or Distract

● Why it helps: If your husband becomes agitated or upset, redirection can help to shift his focus away from a stressful or confusing topic.</td></tr>
</table>

group for caregivers, which can offer practical advice and emotional support.<br>8. **Consider Professional Help**: If the behaviors are escalating or becoming too overwhelming, it may be helpful to involve a healthcare professional, like a neurologist or a geriatric psychiatrist, who can assess the situation and recommend interventions. Caregiving agencies or professionals trained in dementia care can also provide additional support.<br><br>Taking care of yourself is also very important during this time. It can be emotionally and physically taxing to care for someone with dementia, so make sure to seek support when you need it.	• **What to do**: Offer a distraction, like suggesting a different activity, talking about something he enjoys, or even changing the setting. For example, "Would you like to go for a walk?" or "I think we should have some tea together."<br><br>**4. Address Unmet Needs**<br><br>• **Why it helps**: Sometimes behaviors are driven by physical or emotional needs, such as hunger, pain, discomfort, or fear.<br>• **What to do**: Before jumping to conclusions, check if he is hungry, thirsty, in pain, or uncomfortable. Sometimes, physical needs like needing to go to the bathroom or feeling too hot or cold can trigger frustration.<br><br>**5. Maintain a Routine**<br><br>• **Why it helps**: People with dementia often feel more comfortable and less agitated when there is a predictable routine.<br>• **What to do**: Try to keep daily routines as consistent as possible. Set regular times for meals, activities, and rest. This can help reduce anxiety and confusion.<br><br>**6. Limit Environmental Stimulation**<br><br>• **Why it helps**: Overstimulation (from noise, people, or bright lights) can increase agitation or confusion in someone with dementia.<br>• **What to do**: Create a calm, quiet environment. Turn off the TV, reduce loud noises, or move to a more peaceful area when your husband seems overwhelmed.<br><br>**7. Be Aware of Triggers**<br><br>• **Why it helps**: Understanding what causes the behaviors can help prevent or manage them more effectively.<br>• **What to do**: Pay attention to what seems to trigger negative behavior (certain people, places, or activities). Keeping a journal of these incidents can help identify patterns, which can then inform adjustments in care.

	**8. Involve Him in Simple Tasks**<br>● **Why it helps**: Engaging your husband in activities that are meaningful to him can reduce frustration and help maintain a sense of purpose.<br>● **What to do**: Encourage him to participate in simple tasks that he enjoys, such as folding laundry, watering plants, or helping with meal prep. Even small tasks can boost self-esteem and provide a sense of accomplishment.<br>**9. Avoid Arguing**<br>● **Why it helps**: People with dementia may have difficulty processing what is real, and arguing with them can escalate frustration.<br>● **What to do**: If your husband becomes upset or confused about something, it's better to avoid arguing or correcting him. Instead, gently acknowledge what he's saying without insisting on "facts" that may not be clear to him.<br>**10. Seek Professional Support**<br>● **Why it helps**: If your husband's behaviors become difficult to manage or if you need further assistance, professional help can provide additional strategies and support.<br>● **What to do**: Talk to his healthcare provider about any changes in behavior. They can offer guidance and might suggest treatments or therapies that can improve his quality of life and help manage difficult behaviors.<br>---<br>**Additional Support:**<br>● **Respite Care**: Taking care of someone with dementia can be overwhelming. Consider respite care services, where a trained professional can temporarily take over caregiving duties to give you a break.<br>● **Support Groups**: Talking to other caregivers can be reassuring and helpful. You can share experiences and coping

	strategies with others who understand what you're going through.<br><br>**When to Seek Medical Attention:**<br><br>● If his behavior becomes unsafe or leads to severe confusion, aggression, or other concerning symptoms, it's important to seek medical advice immediately. Sometimes, changes in behavior could indicate a medical issue (such as an infection or medication side effect) that needs attention.<br><br>Taking small, mindful steps can help reduce the stress for both you and your husband. Remember that dementia affects each person differently, so being flexible and responsive to his individual needs will be key to managing these changes effectively.

6. My husband has trouble remembering things and sometimes doesn't understand what I'm saying. What are some simple ways I can talk to him to help him understand better?

<table>
<tr><th>Cond1</th><th>Cond2</th></tr>
<tr><td>It's great that you want to make communication easier for your husband. Here are some strategies that may help him understand you better:
1. Speak Slowly and Clearly: Giving your husband time to process information can make a big difference. Avoid rushing your words and make sure your voice is calm and clear.
2. Use Simple Words and Sentences: Try to keep your sentences short and to the point. Avoid complex phrases or long explanations, as these can be harder to follow.
3. Repeat Information: Sometimes repetition is key. If something seems unclear, try restating it in a simpler way or using different words.
4. Use Visual Cues: Sometimes showing him something or pointing to an object can help make your point clearer. Visual aids can support verbal communication and make it easier to understand.
5. Maintain Eye Contact: This helps to keep his attention on the conversation. If possible, try to reduce distractions around you so he can focus on what you're saying.
6. Use Written Reminders: If he struggles with memory, writing things down can be helpful. You can also leave notes or reminders that he can refer back to later.
7. Ask for Feedback: After explaining something, ask him if he understands or if he has any questions. This can help you gauge his understanding and adjust if needed.
8. Be Patient: It might take a little extra time, but your patience can make a big difference. If he's having trouble, try not to get frustrated. Instead, offer gentle encouragement.
9. Ensure a Comfortable Environment: A quiet, calm space can help him focus better. Try to have conversations in places with fewer distractions.

Would you like more personalized tips based on your specific situation?</td><td>It's challenging when your husband has difficulty remembering things and understanding what you're saying, but there are several strategies that can help improve communication and make it easier for him to follow along. Here are some simple ways to talk to him:

1. Speak Slowly and Clearly
● Why it helps: People with memory and comprehension difficulties may need more time to process what you're saying.
● What to do: Use short, simple sentences and pause between each one. Speak slowly and calmly, and avoid using overly complex words or jargon.

2. Use Simple Words and Direct Instructions
● Why it helps: Alzheimer's or dementia can make it harder to follow long explanations or abstract concepts.
● What to do: Break down what you want to say into simple words and straightforward instructions. For example, instead of saying "Please put the dish in the cupboard," say "Please put the plate in the kitchen cupboard."

3. Use Non-Verbal Cues
● Why it helps: Sometimes visual cues can make it easier for him to understand what you're asking.
● What to do: Point to things or demonstrate the action you're asking him to do. If you're asking him to sit down, you could gesture to the chair and gently guide him. Facial expressions and body language can also communicate warmth and reassurance.

4. Ask One Question at a Time
● Why it helps: Multiple questions can be overwhelming and confusing.
● What to do: Ask one simple question at a time and wait for a response before</td></tr>
</table>

	asking another. For example, instead of "Do you want to eat lunch now or later?", ask "Would you like lunch now?" and wait for an answer before moving to the next question.<br><br>**5. Use Reassurance and Be Patient**<br><br>● **Why it helps**: People with memory issues may become anxious or frustrated when they don't understand something.<br><br>● **What to do**: Reassure him that it's okay and that you're there to help. If he doesn't understand right away, calmly repeat the information or question, or try a different way of explaining it. Avoid showing frustration.<br><br>**6. Maintain Eye Contact and Stay Close**<br><br>● **Why it helps**: Sometimes a person with dementia may struggle with hearing or focusing, so being close and maintaining eye contact can help them stay engaged.<br><br>● **What to do**: Position yourself at his eye level and make sure you're facing him when talking. This helps capture his attention and makes it easier for him to focus on the conversation.<br><br>**7. Give Him Time to Respond**<br><br>● **Why it helps**: Processing information takes longer for some people with dementia.<br><br>● **What to do**: After asking a question or making a request, give him extra time to respond. Be patient and don't interrupt while he's thinking.<br><br>**8. Use Familiar Topics or Memory Aids**<br><br>● **Why it helps**: Sticking to topics he's familiar with can make it easier for him to engage in conversation.<br><br>● **What to do**: Talk about things that he enjoys or remembers well, like favorite activities, past experiences, or family memories. You can also use photographs or other visual aids to help him recall important details.

9. Avoid Arguing or Correcting

- **Why it helps**: If your husband forgets something or misunderstands, it can lead to frustration for both of you. Correcting him might cause embarrassment or agitation.
- **What to do**: If he makes a mistake or has trouble recalling something, try to gently redirect the conversation rather than correcting him. For example, if he forgets an event, say something like, "That sounds like it was an interesting time," instead of correcting the memory.

10. Provide Written Reminders

- **Why it helps**: Some people with dementia benefit from seeing written instructions or notes.
- **What to do**: If your husband struggles with remembering specific details, you can write down important things on a whiteboard, notepad, or sticky notes. For example, you can have reminders like "Lunch is at 12 PM" or "We're going to the doctor at 2 PM."

Example Scenario:

- **You**: *"Hi, sweetheart. Do you want to eat now?"* (Ask one question at a time)
- **Him**: *"What?"* (He's unsure or distracted)
- **You**: *"Would you like lunch now? We can have some soup."* (Use simple words, point to the food)
- **Him**: *"Yes, that sounds nice."* (He agrees)

Final Tip:

Each person with dementia experiences communication challenges differently, so it might take some time to find the strategies that work best for your husband. Stay flexible and patient, and don't hesitate to ask for professional guidance if you're unsure about how to best communicate with him.

7. I'm not sure how to take care of my husband with all these changes, and I could use some help. Are there any simple tools or resources I can use to understand more about caring for someone with dementia?

**Cond1**	**Cond2**
I'm sorry you're going through this. Caring for someone with dementia can be challenging, but there are definitely tools and resources available that can help you navigate this process. Here are some simple and effective strategies you can use to better understand and care for your husband:<br>1. Dementia Education Resources:<br>● Alzheimer's Association: They offer comprehensive guides on how to care for someone with dementia, including behavioral changes, communication tips, and caregiving strategies. They have a website and a 24/7 helpline: 1-800-272-3900.<br>o Alzheimer's Association Caregiving Resources<br>● National Institute on Aging: Offers helpful articles and videos on caregiving, from early signs to advanced stages of dementia.<br>o NIA Dementia Caregiving Resources<br>2. Support Groups:<br>● Joining a support group can help you connect with others going through similar experiences. Both online and in-person options exist. These groups can offer emotional support and practical advice.<br>● The Alzheimer's Association and AARP both offer support groups for caregivers.<br>3. Practical Tools for Caregivers:<br>● Caregiver Journals: Keep track of his behavior, your caregiving tasks, and any changes you notice. This will help you stay organized and allow you to share useful information with healthcare professionals.<br>● Daily Routine Apps: Apps like CareZone or CaringBridge allow you to manage appointments, medication schedules, and share updates with family.<br>4. Self-Care Resources for You:<br>● It's crucial to take care of yourself too. Websites like Caregiver Action Network provide resources to help you avoid burnout.<br>● Schedule some time each day for self-care, even if it's just a short break, to recharge and manage stress.<br>5. Books and Guides for Caregivers:<br>● "The 36-Hour Day" by Nancy L. Mace and Peter V. Rabins: This book is a classic guide for family caregivers, providing practical advice on managing dementia care.	Taking care of a spouse with dementia can feel overwhelming, especially with the emotional and practical challenges it brings. It's great that you're seeking resources and tools to help you navigate this journey. Here are some helpful tools and resources to guide you in understanding and caring for your husband:<br><br>**1. The Alzheimer's Association**<br><br>● **Website**: www.alz.org<br>● The Alzheimer's Association provides comprehensive resources on everything from early symptoms to end-of-life care. They offer caregiving tips, support groups, educational materials, and a 24/7 helpline.<br>o **24/7 Helpline**: 1-800-272-3900<br>o **Key Resources**: You can find articles on managing behavior changes, communication strategies, and specific caregiving techniques that will help you support your husband.<br><br>**2. Dementia Caregiver Support Groups**<br><br>● Connecting with others who are experiencing similar challenges can provide emotional support and valuable advice.<br>● **Alzheimer's Association** offers local support groups (many are virtual or phone-based) where you can connect with other caregivers to share experiences and coping strategies. Check the website for local options.<br><br>**3. National Institute on Aging (NIA)**<br><br>● **Website**: www.nia.nih.gov<br>● The NIA provides reliable, up-to-date information about Alzheimer's and related dementias. They also offer guides on caregiving and tips for managing daily tasks.<br>o **Caregiver Resource Center**: Helpful guides and research-based information on how dementia affects loved ones and how you can adapt

- "Contented Dementia" by Oliver James: This approach focuses on creating a life for your loved one that is fulfilling, despite the challenges of dementia.

6. Behavioral Management Tools:

- Communication Tips: Dementia often affects a person's ability to communicate. It may help to speak slowly, use simple words, and offer reassurance. Avoid arguing or correcting them, and focus on offering comfort.
- Structured Environment: Maintaining a routine can reduce confusion. Set consistent times for meals, activities, and sleep.

7. Professional Help:

- Consider hiring a professional caregiver or seeking adult day programs to give yourself some respite and ensure your husband gets the best care.
- Geriatric care managers can also offer guidance on managing the day-to-day aspects of caregiving.

These tools and resources should give you a good starting point. Don't hesitate to reach out to others who understand this journey, and remember that it’s okay to ask for help.

your caregiving approach as the disease progresses.

4. Alzheimer's Caregiver Manual

- This **free downloadable manual** from the Alzheimer’s Association provides guidance on everyday tasks, handling difficult behaviors, and offering emotional support to your spouse.

5. Daily Planner Tools

- **Memory Aids and Tools**: Simple tools, like daily planners or reminder apps, can help you and your husband keep track of daily activities, appointments, and medications. Here are a few to consider:
    - **MyTherapy**: A medication management app that reminds you when to take medications.
    - **Pill Organizer Box**: A physical pillbox can be very helpful for organizing medications into daily compartments, reducing the risk of missed or double doses.
    - **Day Clock**: A clock with the date and time can help orient your husband and reduce confusion about the day or time of day.

6. The Alzheimer's Foundation of America (AFA)

- **Website**: www.alzfdn.org
- AFA offers caregiving advice, tools, and resources specific to managing Alzheimer's care. They have a focus on education and family support and provide a national helpline for caregivers.
    - They also offer **webinars** and **guides** on how to deal with different stages of Alzheimer's, including how to manage behaviors, finances, and legal matters.

7. Books for Caregivers

- Many caregivers find it helpful to read books that offer advice on how to care for someone with dementia. A few widely recommended titles include:
    - **"The 36-Hour Day"** by Nancy L. Mace and Peter V. Rabins: A comprehensive guide for caregivers

of people with Alzheimer’s and other dementias.
    - **"Creating Moments of Joy"** by Jolene Brackey: A positive approach to helping someone with Alzheimer's maintain a sense of joy and dignity.

8. Local Resources and Services

- **Home Care Services**: If caregiving becomes too difficult or if you need respite, local home care services can help. Contact your local Area Agency on Aging (AAA) to inquire about home care options in your area.
- **Respite Care**: Some facilities offer short-term respite care to give caregivers a break. You can find more about these services through the Alzheimer's Association or local senior services.

9. Technology Aids

- **Medical Alert Systems**: These are helpful for peace of mind, especially if your husband has a tendency to wander. Systems can be equipped with GPS tracking and emergency response features.
- **Smart Home Devices**: Voice-controlled devices like **Amazon Alexa** or **Google Assistant** can help with reminders for medications, appointments, or daily tasks.

10. Personal Care Resources

- **Caregiving Tips for Daily Activities**: Websites like the Alzheimer's Association and National Institute on Aging offer specific advice on helping your husband with daily tasks like eating, bathing, grooming, and managing medications.

A Few Key Steps You Can Take:

- **Assess his symptoms**: Keep a journal of his behaviors, memory lapses, and any changes you observe. This will help you and his doctor assess his condition.
- **Involve a healthcare provider**: Early intervention can help address symptoms and

	give you the tools to manage the changes more effectively.<br>● **Create a routine**: A consistent routine helps reduce confusion. This includes regular meal times, sleep schedules, and familiar activities.<br>● **Manage your own well-being**: It's important to care for yourself as well. Take breaks when needed, reach out to support groups, and don't hesitate to seek professional help if you feel overwhelmed.<br>---<br>You don’t have to face this alone—there are many resources, communities, and professionals who can guide and support you through this journey.

Domain 3 – Dementia Literacy

8. I want to do everything I can to help my husband, but I'm not sure what changes we should make to keep his mind sharp. What kind of simple lifestyle changes can I encourage to help slow down or prevent his condition from getting worse?

**Cond1**	**Cond2**
It's wonderful that you're looking to support your husband in maintaining his cognitive health. Depending on his condition, there are a variety of lifestyle changes that can help slow cognitive decline or keep his mind sharp. Here are some simple yet effective changes you can encourage:<br>1. Physical Exercise<br>Regular physical activity is one of the most powerful ways to protect cognitive function. Encourage him to do something active every day, even if it's a short walk, cycling, or swimming. Aim for at least 30 minutes most days of the week. Exercise improves blood flow to the brain and supports the growth of new brain cells.<br>2. Mental Stimulation<br>Keeping the brain engaged through activities that challenge thinking can help maintain cognitive function. These could include:<br>● Puzzles or brain games (crosswords, Sudoku, or memory games).<br>● Learning something new, like a language or a hobby.<br>● Reading books or watching educational videos on interesting topics.<br>● Playing musical instruments or learning new skills.<br>3. Healthy Diet<br>A balanced diet rich in nutrients can support brain health. Encourage:<br>● Mediterranean diet: High in fruits, vegetables, whole grains, olive oil, and fish.<br>● Omega-3 fatty acids: Found in fatty fish (salmon, mackerel), flaxseeds, and walnuts.<br>● Antioxidants: Found in berries, dark leafy greens, and colorful vegetables.<br>● Avoiding excessive sugar, processed foods, and unhealthy fats.<br>4. Quality Sleep<br>Good sleep is essential for cognitive function. Encourage a consistent sleep schedule, and aim for 7-9 hours of quality sleep. Creating a calming bedtime routine and reducing exposure to screens before bed can help improve sleep quality.<br>5. Social Engagement<br>Maintaining strong social connections is vital for brain health. Encourage him to:<br>● Stay in touch with friends and family.	Supporting your husband's cognitive health can involve practical lifestyle changes that not only promote his well-being but also create opportunities for meaningful engagement between you both. While dementia itself cannot always be prevented or reversed, these strategies may help slow its progression and improve quality of life.<br><br>**1. Encourage Mental Stimulation**<br><br>● **Brain-boosting activities**:<br>○ Puzzles, word games, Sudoku, or simple math exercises.<br>○ Learning something new, like a hobby or skill (e.g., painting, gardening).<br>● **Reading or listening to books**: Select materials that align with his interests, or listen to audiobooks together.<br>● **Memory aids**: Use calendars, labeled pictures, or apps designed for memory exercises.<br><br>**2. Focus on Physical Activity**<br><br>● **Daily walks**: Gentle physical activity can boost blood flow to the brain and improve mood.<br>● **Exercise programs**:<br>○ Simple yoga or chair exercises for flexibility and strength.<br>○ Dancing or tai chi, which combines movement with mental engagement.<br>● Aim for **30 minutes of activity** a day, tailored to his abilities.<br><br>**3. Maintain a Nutritious Diet**<br><br>● Consider the **MIND Diet** (a combination of the Mediterranean and DASH diets):<br>○ **Leafy greens**: Spinach, kale, or broccoli.<br>○ **Berries**: Blueberries and strawberries, which support brain health.

● Participate in community activities or group hobbies.<br>● Engage in meaningful conversations or join social clubs.<br>6. Stress Management<br>Chronic stress can impair cognitive function. Encourage practices that promote relaxation, such as:<br>● Mindfulness meditation or yoga.<br>● Deep breathing exercises.<br>● Spending time in nature.<br>● Regular breaks during the day to reduce mental overload.<br>7. Routine Medical Check-ups<br>Ensure he stays on top of regular health check-ups, including monitoring blood pressure, cholesterol, and blood sugar levels. These can all impact cognitive function if left untreated.<br>8. Limit Alcohol and Tobacco<br>Excessive alcohol and smoking can impair cognitive function, so reducing or eliminating these habits can help protect brain health.<br>9. Staying Hydrated<br>Dehydration can negatively impact brain function. Make sure he drinks enough water throughout the day.<br>By focusing on these key areas, you can help him maintain or even improve his cognitive health. You might want to also explore any condition-specific advice or interventions that are recommended for his situation, whether it's medication or specialized therapies. Your support will be invaluable in helping him stay engaged and healthy!	o **Healthy fats**: Olive oil, avocados, and nuts.<br>o **Fish**: Salmon, mackerel, or tuna for omega-3 fatty acids.<br>o Limit sugary and highly processed foods.<br>● Stay **hydrated**: Dehydration can worsen confusion and fatigue.<br><br>**4. Create a Structured Routine**<br><br>● A consistent daily schedule can reduce anxiety and confusion. Include:<br>o Time for meals, activities, rest, and relaxation.<br>o Clear cues, like labeled clocks, for time and tasks.<br>● Keep distractions low and simplify his environment.<br><br>**5. Promote Social Connections**<br><br>● Isolation can speed cognitive decline, so encourage meaningful social interactions:<br>o Join a local dementia-friendly group or cultural club.<br>o Invite close friends or family for short visits.<br>o Attend caregiver-supported activities where he can interact with others.<br><br>**6. Prioritize Sleep Hygiene**<br><br>● A restful night's sleep is vital for brain health:<br>o Create a calm bedtime routine with soothing music or dim lighting.<br>o Limit caffeine and large meals before bed.<br>o Keep the bedroom dark and cool to encourage quality sleep.<br><br>**7. Manage Stress and Anxiety**<br><br>● **Relaxation techniques**:<br>o Breathing exercises or mindfulness meditation.<br>o Music therapy: Play familiar and calming songs.<br>● Provide reassurance and support during moments of confusion or frustration.

	**8. Address Hearing and Vision**<br><br>- Hearing and vision problems can exacerbate cognitive challenges:<br>- Ensure regular check-ups for hearing aids or glasses.<br>- Reduce background noise and use simple cues to aid communication.<br><br>**9. Limit Multitasking**<br><br>- Simplify tasks to reduce cognitive overload:<br>- Break activities into smaller, manageable steps.<br>- Focus on one thing at a time and encourage patience.<br><br>**10. Seek Professional Guidance**<br><br>- Consult with a neurologist or geriatrician for advice tailored to your husband's specific condition.<br>- Ask about cognitive therapy programs or medications that may help maintain his current abilities.<br><br>**11. Explore Cognitive Apps and Tools**<br><br>- **Memory and focus apps**:<br>- Lumosity, Elevate, or apps designed for seniors.<br>- Use simple reminders on phones or tablets to keep him engaged and oriented.<br><br>**12. Stay Flexible and Compassionate**<br><br>- Focus on what your husband can do, rather than what he struggles with.<br>- Celebrate small victories, and adapt activities as his abilities change.<br><br>Would you like help finding specific programs or tools in your area to support these changes? Let me know, and we can explore resources together!

9. I live in Fairfax County and I'm looking for activities or therapies in Korean that might help slow down my husband's dementia. Are there any programs or services available that have been proven to help with this?

**Cond1**	**Cond2**
There are several programs and therapies in the Fairfax County area that could support your husband in managing dementia, and while there might not be specific programs entirely in Korean, many services do provide support for individuals from diverse backgrounds. Here are a few options you could consider:<br>1. Korean-American Alzheimer's Association (KAAA)<br>● Programs: They provide services and support specifically for Korean-speaking families dealing with dementia. The KAAA has educational programs, support groups, and resources for caregivers, which can be very helpful.<br>● Location: Although they may not be based in Fairfax, they serve the greater Washington, D.C., area, including Northern Virginia. You could contact them for more specific information about local programs.<br>2. Fairfax County Aging Services<br>● Fairfax County offers various programs for people with dementia, including caregiver support and therapy services. You can reach out to the Fairfax Area Agency on Aging to inquire about language-specific services or therapists who may speak Korean. They may also offer dementia-friendly activities and memory care programs.<br>3. Memory Care Programs<br>● Some local senior living communities or memory care facilities may offer specialized programs for dementia patients that include therapeutic activities like music therapy, art therapy, or cognitive exercises that can help slow the progression of dementia. Ask about Korean-speaking staff or programs to make your husband more comfortable.<br>4. Therapies for Dementia<br>● Cognitive Stimulation Therapy (CST): Research has shown that CST can be effective for people with mild to moderate dementia. Some facilities may offer this therapy in different languages, so it's worth checking if any in your area offer CST in Korean.<br>● Music Therapy: Korean music therapy could be beneficial, especially if your husband has a connection to his native culture through music.<br>● Reminiscence Therapy: This therapy helps engage memory through discussion of past events, often	Fairfax County offers various services and programs tailored to the needs of individuals with dementia, and there are also resources in the Korean language to accommodate your cultural and linguistic needs. Below are some activities, therapies, and resources that may be beneficial:<br><br>**1. Local Programs and Activities**<br><br>**Fairfax County Adult Day Health Care Centers**<br><br>● These centers provide structured activities, including memory games, exercise, and socialization opportunities, which can help slow cognitive decline.<br>● Some centers may have Korean-speaking staff or culturally tailored services. Call to inquire about language support.<br>● Locations:<br>○ **Herndon Harbor Adult Day Health Center**<br>○ **Lincolnia Adult Day Health Center**<br>● **Contact**: Fairfax County Aging, Disability, and Caregiver Resources Line at **703-324-7948**.<br><br>**Korean Community Service Center of Greater Washington (KCSC)**<br><br>● Offers culturally relevant support for Korean seniors, including memory care workshops, health education, and social activities.<br>● **Contact**: 703-354-6345<br>● **Website**: www.kcscgw.org<br><br>**Korean Senior Centers and Churches**<br><br>● Many Korean churches in the area, such as **Korean Central Presbyterian Church** in Centreville or **Open Door Presbyterian Church**, host senior programs, which may include exercise classes, music therapy, and social gatherings.<br>● These settings can provide a comforting environment for your husband while fostering community connections.<br><br>**2. Proven Therapies and Activities**

<table>
<tr>
<td>using photographs, objects, or music that holds meaning for the individual.

You can begin by contacting local senior services in Fairfax County or Korean-specific organizations like KAAA to explore more tailored options. Additionally, family caregivers' support groups in Korean could also be an avenue for gathering more local recommendations from others in similar situations.</td>
<td>

Music Therapy

- Music therapy has been shown to help individuals with dementia recall memories and improve mood. Look for programs that incorporate traditional Korean music.
- **Aging Matters Music Program (Korean-specific)**: Call the **Fairfax County Health Department** to inquire about culturally relevant music therapy offerings.

Art or Craft Programs

- Art activities can stimulate creativity and improve focus. Korean-specific craft sessions (e.g., making traditional Korean decorations or calligraphy) are often hosted by senior centers or community groups.

Exercise and Movement

- Programs such as tai chi or gentle stretching have been shown to improve cognitive and physical health.
- Some Korean groups in the area host such classes, including the KCSC.

3. Online Resources for Dementia-Friendly Activities

YouTube Korean Dementia Resources

- Many YouTube channels in Korean provide cognitive exercises, storytelling, or guided activities specifically for seniors with dementia.
  - Search for **치매 예방 운동 (Dementia Prevention Exercises)** or **기억력 향상 활동 (Memory Improvement Activities)**.

Apps for Brain Health

- **세로 기억력 게임 (Vertical Memory Game)** or **Korean-specific memory training apps** available on smartphones may be a fun way to engage your husband in cognitive exercises.

4. Memory Cafés

</td>
</tr>
</table>

	Fairfax County has **Memory Cafés**, where caregivers and their loved ones can socialize, participate in activities, and receive support. Some cafés may offer Korean-speaking facilitators:<br><br>● Contact the **Insight Memory Care Center** at **703-204-4664** to ask about Korean-language Memory Cafés.<br><br>**5. Caregiver Education and Support**<br><br>● The **Alzheimer's Association National Capital Area Chapter** provides educational materials and workshops for caregivers in Korean, covering strategies to engage loved ones with dementia.<br>○ **Contact**: 1-800-272-3900 (ask for Korean-speaking assistance).<br>○ **Website**: www.alz.org<br><br>**6. Culturally Specific Meals**<br><br>Nutrition plays a vital role in brain health. Many adult day programs and Korean community centers ensure culturally familiar meals like Korean cuisine are served, helping your husband feel more at home.<br><br>**7. Fairfax County Virtual Activities**<br><br>During inclement weather or when staying home, virtual activities can engage your husband. Look into:<br><br>● **Fairfax County Virtual Senior Centers**: They sometimes offer culturally specific programs.<br><br>**Next Steps**<br><br>1. **Contact the Fairfax County Aging and Disability Services**:<br>○ Call **703-324-7948** and ask about Korean-specific programs for individuals with dementia.<br>2. **Reach out to the Korean Community Service Center (KCSC)** for culturally tailored dementia programs.<br>3. Visit or call local Korean churches or senior groups for more information on group activities.<br>4. Explore private therapists or geriatric care providers fluent in Korean for one-on-one therapies.

	Let me know if you'd like help contacting any of these organizations or need assistance finding more resources!

10. My husband doesn't speak or understand English very well. What are the common signs of mild cognitive impairment or early dementia that I should be watching for? And where can I take him for the right check-up or evaluation, especially with language barriers?

**Cond1**	**Cond2**
Mild cognitive impairment (MCI) and early dementia can sometimes be hard to spot, especially when there's a language barrier. However, there are some common signs you can watch for:<br>1. Memory issues: He may forget things more often, like appointments or recent conversations, and have trouble remembering important events.<br>2. Difficulty with problem-solving: Tasks that used to be simple, like balancing a checkbook or planning activities, might become harder for him.<br>3. Language problems: He may struggle to find the right words, repeat himself, or have trouble understanding or following conversations, even in his primary language.<br>4. Confusion or disorientation: He might get confused about time, place, or people, or become easily lost in familiar areas.<br>5. Difficulty with daily tasks: He might have trouble managing daily activities like cooking, bathing, or dressing.<br>6. Personality changes: His mood or behavior could change. He might become more anxious, depressed, or withdrawn.<br>7. Poor judgment or decision-making: He could make uncharacteristic mistakes or show poor judgment in situations he previously handled well.<br>For the check-up or evaluation, you can consider the following:<br>● Primary Care Physician (PCP): Start by taking him to a doctor who can assess his cognitive function and refer you to a specialist if needed. Look for a doctor who speaks his primary language or has access to an interpreter.<br>● Neurologist: A neurologist specializes in brain health and can conduct tests to assess cognitive impairment. Some clinics offer multilingual staff or translation services.<br>● Memory or dementia clinic: Some hospitals have clinics specifically for dementia and cognitive issues. They may provide evaluations in multiple languages or have language services available.<br>● Geriatrician: A doctor specializing in the care of older adults can help assess if changes in cognition are related to normal aging or early dementia.<br>If you're concerned about language barriers, it's a good idea to ask the clinic about interpreter services before making an appointment. Many health systems have	Recognizing the early signs of mild cognitive impairment (MCI) or early dementia in someone who has language barriers can be challenging but is important for timely care. Below are the common symptoms to watch for, tailored to your situation, along with suggestions for culturally and linguistically accessible resources.<br><br>**Common Signs of Mild Cognitive Impairment (MCI) or Early Dementia**<br><br>1. **Memory Issues**:<br>o Frequently forgetting recent events, conversations, or appointments.<br>o Repeating questions or stories multiple times within a short period.<br>2. **Language Difficulties**:<br>o Struggling to find words or mixing up familiar terms, even in their native language.<br>o Difficulty following conversations or instructions in any language.<br>3. **Disorientation**:<br>o Getting lost in familiar places, such as the neighborhood or home.<br>o Losing track of time, days, or seasons.<br>4. **Changes in Personality or Mood**:<br>o Becoming more withdrawn, anxious, or irritable.<br>o Showing less interest in hobbies or social interactions.<br>5. **Judgment and Decision-Making**:<br>o Difficulty handling finances, forgetting to pay bills, or making impulsive decisions.<br>o Confusion over how to perform familiar tasks, like cooking or using household appliances.<br>6. **Difficulty with Multitasking or Planning**:<br>o Trouble organizing daily tasks, managing schedules, or focusing on more than one thing at a time.<br>7. **Physical Signs**:<br>o Misplacing items and being unable to retrace steps to find them.<br>o Changes in gait, coordination, or physical behavior.

bilingual staff or can arrange for an interpreter to help with communication during medical visits.	**Where to Take Him for a Check-Up**<br><br>Given the language barrier, it's essential to find a healthcare provider who speaks your husband's language or has access to interpreter services. Since you're in **Virginia**, here's how you can proceed:<br><br>**1. Multilingual Memory Assessment Clinics**<br><br>Look for clinics or specialists who provide cognitive assessments and have staff fluent in your husband's language (e.g., Korean, Spanish, Vietnamese, Chinese).<br><br>● **Fairfax Memory Program**:<br>○ Offers cognitive evaluations for individuals with memory concerns.<br>○ Contact **703-324-7948** and request language-specific assistance.<br>● **Inova Memory and Cognitive Disorders Program**:<br>○ Specializes in diagnosing and treating memory issues.<br>○ Located in Northern Virginia, they may provide interpreter services if no bilingual providers are available.<br><br>**2. Geriatricians or Neurologists with Language Support**<br><br>● Use the **Community Resource Finder**: www.communityresourcefinder.org to locate dementia specialists who speak your husband's language.<br>● Consider contacting the **Korean Community Service Center (KCSC)** at **703-354-6345** for referrals to culturally sensitive healthcare providers.<br><br>**3. Alzheimer's Association Helpline**<br><br>Call **1-800-272-3900** to speak with someone who can help you locate a provider fluent in your husband's language.<br><br>**4. Local Memory Assessment Centers**<br><br>● **Insight Memory Care Center**:<br>○ Provides assessments, caregiver support, and resources in the Northern Virginia area.<br>○ Call **703-204-4664** to ask about language accommodations.

	**5. University-Based Clinics**<br><br>● **Georgetown Memory Disorders Program**:<br>o They often have specialists familiar with diverse cultural backgrounds and offer interpreter services.<br><br>**How to Prepare for the Evaluation**<br><br>1. **Document Concerns**:<br>o Keep a log of symptoms, including when they began and how they've progressed.<br>o Note specific situations where his memory, language, or behavior has been concerning.<br>2. **Bring a Caregiver or Family Member**:<br>o If possible, bring someone who speaks both English and his native language to help bridge gaps.<br>3. **Ask About Culturally Relevant Tests**:<br>o Many cognitive assessments are language-specific. Request that they use tools appropriate for his cultural background to avoid misdiagnosis.<br><br>**Other Resources**<br><br>1. **Fairfax County Services for Older Adults**:<br>o Offers caregiver support and senior services tailored to diverse communities.<br>o Call **703-324-7948**.<br>2. **Ethnic Senior Centers**:<br>o Local Korean, Spanish, or other ethnic community centers often have programs for seniors, including health screenings.<br>3. **Online Support**:<br>o Join caregiver forums or support groups for dementia caregivers in your language. The Alzheimer's Association hosts multilingual caregiver groups.<br><br>**Next Steps**<br><br>1. Watch for the signs mentioned above and document any patterns of concern.<br>2. Schedule an appointment with a memory specialist or neurologist with language accommodations.

	3. Explore support resources for you as a caregiver to help navigate this journey together.<br><br>Let me know if you'd like me to help contact these organizations or find additional resources tailored to your needs!

11. How can I tell the difference between memory changes that just come with old age and signs of mild cognitive impairment (early dementia) in my husband?

**Cond1**	**Cond2**
It's understandable to want to differentiate between normal age-related memory changes and early signs of mild cognitive impairment (MCI), which could potentially lead to dementia. Here are some key differences that can help guide you:<br>1. Frequency and Severity of Memory Loss<br>● Normal Aging: Occasional forgetfulness is common, such as misplacing keys or forgetting an appointment, but these instances are generally infrequent and not disruptive to daily life.<br>● Mild Cognitive Impairment: Memory loss becomes more frequent and noticeable. For example, your husband might forget recent conversations, appointments, or important events, and this forgetfulness may become more persistent over time.<br>2. Impact on Daily Functioning<br>● Normal Aging: There may be slight difficulty with multitasking or recalling specific names, but overall, he can manage daily tasks independently, like cooking, paying bills, and driving.<br>● Mild Cognitive Impairment: There might be noticeable difficulty with routine tasks that were once easy for him. This could include getting lost in familiar places, trouble managing finances, or forgetting how to perform once-familiar activities.<br>3. Cognitive Changes Beyond Memory<br>● Normal Aging: Occasional word-finding difficulties or slower processing speeds are common but don't usually interfere with overall conversation or comprehension.<br>● Mild Cognitive Impairment: There may be more frequent problems with thinking, reasoning, or making decisions. He might struggle with complex tasks, have trouble following conversations, or show unusual decision-making behaviors.<br>4. Changes in Mood or Behavior<br>● Normal Aging: While older adults might experience some mood changes (like frustration with technology), these are usually manageable and transient.<br>● Mild Cognitive Impairment: There can be more significant mood swings, irritability, or apathy. Your husband might become withdrawn, anxious, or depressed due to his awareness of his cognitive difficulties.<br>5. Awareness of Cognitive Decline	Understanding the differences between normal age-related memory changes and signs of mild cognitive impairment (MCI) or early dementia can help you determine whether your husband's symptoms are part of the natural aging process or something more serious. Here's a guide to help you distinguish between the two:<br><br>**1. Normal Aging Memory Changes**<br><br>As people age, their brain processes information more slowly, and occasional memory lapses are common. These changes do not interfere with daily life or independence. Some examples include:<br><br>● **Forgetting names or appointments but remembering them later.**<br>● **Occasionally misplacing items** like glasses or keys but finding them after retracing steps.<br>● **Having difficulty multitasking or concentrating** in noisy or distracting environments.<br>● Taking **a little longer to recall information** but eventually getting there.<br>● Occasionally needing **reminders for tasks** or errands.<br><br>These lapses are not progressive, and they do not significantly impact daily functioning.<br><br>**2. Signs of Mild Cognitive Impairment (MCI)**<br><br>MCI is a condition where cognitive changes are noticeable but not severe enough to interfere significantly with daily life. It may be an early stage of dementia. Symptoms include:<br><br>● **Frequently forgetting recent conversations, events, or appointments.**<br>o Example: Repeatedly asking the same question after getting an answer.<br>● **Difficulty making decisions or solving problems** that used to be simple.<br>● Increased **trouble with planning or organizing**, such as managing finances or preparing meals.<br>● **Misplacing items** and being unable to retrace steps to find them.<br>● **Changes in language**, like trouble finding the right words during conversations.

- Normal Aging: People often recognize when their memory is a little slower, but they usually can still compensate for it.
- Mild Cognitive Impairment: He may be more aware of his cognitive decline. People with MCI may notice their memory or thinking abilities are changing but may be able to compensate in ways that delay the recognition of bigger problems.

6. Duration

- Normal Aging: Memory lapses are generally occasional, temporary, and don't worsen quickly.
- Mild Cognitive Impairment: Changes tend to persist or worsen over time, indicating a potential progression toward more serious cognitive issues.

When to Seek Medical Advice:

If you're noticing that your husband's memory issues or cognitive changes are becoming more frequent or affecting his day-to-day functioning, it might be time to consult a doctor. Early detection of MCI can help manage symptoms and possibly slow progression. A healthcare professional can conduct tests (like memory assessments and neuropsychological evaluations) to help determine if the symptoms are due to normal aging, MCI, or another condition.

It's important to monitor the changes over time and keep track of any patterns or concerns you notice to discuss with his healthcare provider.

- Becoming **easily distracted or overwhelmed** in busy environments.

With MCI, these changes are noticeable to others but do not yet affect the ability to live independently.

3. Early Signs of Dementia

Dementia involves progressive cognitive decline that affects daily functioning and independence. Symptoms of early dementia may include:

- **Getting lost in familiar places** or being unable to follow directions.
- **Confusion about time or place**, such as forgetting what day it is or why they're in a certain location.
- **Difficulty managing finances or medications** that were previously handled with ease.
- Trouble **following conversations or understanding instructions** in any language.
- Significant **mood or personality changes**, such as increased irritability, apathy, anxiety, or depression.
- **Poor judgment or decision-making**, such as wearing inappropriate clothing for the weather or responding inappropriately to social situations.
- Loss of **interest in hobbies or social activities.**

These symptoms typically progress over time and begin to interfere with work, social interactions, and daily responsibilities.

Key differences: table

Steps to Take If You're Concerned

If you're noticing signs of MCI or dementia, here's what you can do:

1. **Start a Symptom Journal**:
   - Document the behaviors, frequency, and context (e.g., forgetting appointments, getting lost, mood changes). This will help you track patterns and provide clear information to a doctor.
2. **Consult a Primary Care Doctor**:
   - Start with your husband's primary care provider to rule out treatable

causes like vitamin deficiencies, thyroid issues, depression, or medication side effects.

3. **Get a Cognitive Evaluation**:
    - Ask for a referral to a neurologist, geriatrician, or memory care specialist who can perform detailed cognitive tests and imaging (if necessary).
4. **Involve Specialists with Language Support**:
    - If language is a barrier, look for healthcare providers who speak your husband's language or use interpreter services to ensure he feels comfortable and understood.
5. **Encourage Healthy Habits**:
    - Regular physical activity, a balanced diet, mental stimulation, and social engagement can help maintain brain health and possibly slow cognitive decline.
6. **Educate Yourself**:
    - Learn more about dementia and caregiving resources. Organizations like the **Alzheimer's Association** offer free online information and support.

Where to Get Help

- **Fairfax County Services for Older Adults**:
    - Call **703-324-7948** for local dementia resources, including cognitive screenings and caregiver support.
- **Memory Care Clinics**:
    - Contact the **Inova Memory and Cognitive Disorders Program** or **Georgetown Memory Disorders Program** for comprehensive evaluations.
- **Alzheimer's Association Helpline**:
    - Call **1-800-272-3900** for 24/7 support, including guidance for finding local specialists.
- **Community Resource Finder**:
    - Use www.communityresourcefinder.org to search for nearby memory specialists or support groups.

Final Thought

You know your husband best. If you feel that his behavior is unusual or affecting daily life, trust your

	instincts and seek professional advice. Early evaluation can lead to better planning and interventions to support both him and you. Let me know if you'd like help finding specialists or resources tailored to his needs!

Key Differences

Normal Aging	MCI or Early Dementia
Forgets names but recalls later.	Repeatedly forgets names or events and does not recall them.
Misplaces items occasionally.	Loses items often and cannot retrace steps to find them.
Slower thinking but able to adapt.	Trouble solving familiar problems or making decisions.
Independent in daily life.	Needs help with tasks like bills, shopping, or cooking.
Personality and mood stable.	Noticeable changes in mood, behavior, or personality.

---

12. My husband has been showing signs of mild memory problems, like forgetting names or where he put things, but I'm not sure if this is just part of getting older or something more serious. I've heard about mild cognitive impairment (MCI), and I'm worried that it might get worse. What are the risks if his MCI progresses to full dementia, and how can I prepare for what might come next?

<table>
<tr><th>Cond1</th><th>Cond2</th></tr>
<tr><td>
It's understandable to be concerned if you're noticing signs of memory issues in your husband. Mild cognitive impairment (MCI) is a condition where people experience noticeable changes in memory or thinking that are beyond what's typical for their age, but not severe enough to interfere significantly with daily life. MCI can be a precursor to dementia, but not everyone with MCI will go on to develop it. In fact, some people with MCI may remain stable or even see improvements over time.
If MCI progresses to dementia, the risks depend on the type of dementia that develops, with Alzheimer's disease being the most common. As dementia progresses, it can cause more severe cognitive decline, affecting memory, language, decision-making, and the ability to perform everyday tasks. Eventually, this can lead to significant changes in behavior and personality, requiring more support for the person affected.
The risks of progression from MCI to dementia include:

Increased memory loss: Short-term and long-term memory may be affected more.
Difficulty with daily activities: Tasks like dressing, cooking, or managing finances may become challenging.
Mood and personality changes: Depression, irritability, or apathy can occur.
Loss of independence: As cognitive and functional abilities decline, a person may need more help from caregivers.

How to prepare for the future:

Consult a doctor: A healthcare provider, especially a neurologist, can help diagnose MCI and track its progression. Early diagnosis and intervention may provide treatments or strategies that slow progression.
Stay informed: Learn more about MCI, dementia, and Alzheimer's disease. Understanding the condition can help you manage expectations and plan ahead.
Create a supportive environment: Help your husband manage memory issues by keeping a routine, using reminders (e.g., notes or alarms), and reducing stressors that could exacerbate his symptoms.

</td><td>
It's understandable to be concerned about your husband's memory problems and what they might mean for the future. Mild Cognitive Impairment (MCI) is often a transitional stage between normal aging and dementia, but not everyone with MCI develops dementia. Here's what you need to know about the risks, what you can do to prepare, and how to support your husband moving forward:
Understanding MCI and Its Risks

What Is MCI?
MCI involves noticeable cognitive changes, such as memory lapses or difficulty concentrating, but these changes do not significantly interfere with daily life or independence.
Progression Risks:
About 10-15% of people with MCI develop dementia each year, compared to about 1-2% of the general older adult population. Over five years, 30-50% of people with MCI may progress to dementia.
Factors that increase the risk of progression include:
A family history of Alzheimer's or other dementias.
The presence of the APOE-e4 gene, a genetic risk factor for Alzheimer's.
MCI caused by Alzheimer's-related changes (e.g., amyloid plaques).
Signs of impaired executive functioning, language difficulties, or spatial awareness problems.
Stability or Reversal:
For some, MCI remains stable for years or may even improve, especially if it is caused by reversible factors like stress, depression, sleep issues, or medication side effects.

How to Prepare If MCI Progresses to Dementia

Early Planning:

</td></tr>
</table>

4. Consider cognitive training: Some evidence suggests that mental exercises or activities that promote brain health, such as puzzles, reading, or learning new skills, may help slow cognitive decline.
5. Encourage physical and social activity: Regular exercise and social interactions can improve mood and support brain health.
6. Plan for the future: Talk about legal, financial, and medical planning. Consider having discussions around power of attorney, wills, and healthcare preferences before things progress.
7. Get support: Being a caregiver for someone with MCI or dementia can be challenging. Look into support groups for caregivers or professional help to ease the burden on you.

It's important to note that many people with MCI do not develop dementia. Also, treatments and lifestyle changes may help slow progression if dementia does develop. Seeking medical advice is a crucial first step to understand the condition and manage it effectively.

- **Legal and Financial Preparation**:
  - Consult with an attorney to establish or update essential documents, such as a durable power of attorney, advance directives, and wills. This ensures your husband's wishes are respected if his cognitive decline progresses.
- **Capacity Assessment**:
  - Schedule an evaluation with a neurologist or dementia specialist to determine the extent of your husband's memory issues and whether they warrant further monitoring or intervention.

2. **Monitor Symptoms**:
   - Track changes in his memory, reasoning, mood, and daily functioning. Share this information with healthcare providers to detect patterns or progression.
3. **Educate Yourself**:
   - Learn about the **different types of dementia**, as symptoms and progression vary (e.g., Alzheimer's vs. vascular dementia).
   - Explore caregiver training programs or resources, like those offered by the **Alzheimer's Association**.
4. **Plan for Care Needs**:
   - Consider how to adapt your home to accommodate future challenges (e.g., safety features like locks, removal of trip hazards).
   - Research local support services, such as adult day programs or in-home care, so you'll know where to turn if he needs additional help.

What You Can Do Now to Slow Progression

While there's no guaranteed way to prevent MCI from progressing to dementia, certain lifestyle changes may help slow cognitive decline:

1. **Encourage Healthy Habits**:
   - **Physical Activity**:
     - Moderate exercise (e.g., walking, swimming, yoga) improves blood flow to the brain and may delay cognitive decline.

- **Diet**:
  - A brain-healthy diet, like the **Mediterranean** or **MIND diet**, emphasizes fruits, vegetables, whole grains, lean protein, and healthy fats.
- **Sleep**:
  - Ensure your husband gets quality sleep, as poor sleep (e.g., sleep apnea or insomnia) is linked to cognitive decline.

2. **Engage His Mind**:
   - Keep his brain active with **puzzles**, **word games**, or hobbies he enjoys. Learning something new, like a language or musical instrument, can also stimulate cognitive function.
   - Encourage social engagement, which can reduce isolation and improve mental health.
3. **Manage Health Conditions**:
   - Conditions like hypertension, diabetes, high cholesterol, and depression can contribute to cognitive decline. Ensure he attends regular check-ups and adheres to prescribed treatments.
4. **Minimize Risk Factors**:
   - Avoid smoking and excessive alcohol consumption.
   - Address hearing loss with hearing aids if necessary, as hearing impairment has been linked to a higher risk of dementia.
5. **Consider Cognitive Rehabilitation**:
   - Some therapists specialize in memory training or compensatory strategies to help people with MCI maintain independence longer.

Support for You as a Caregiver

1. **Caregiver Support**:
   - Look for local support groups or online communities, like those offered by the **Alzheimer's Association** or **Caregiver Action Network**, to connect with others who understand what you're going through.
2. **Respite Services**:
   - Consider respite care options if you need a break. Local adult day centers

	or temporary in-home care can help lighten your load.<br>3. **Education and Training**:<br>○ Many organizations, such as the **Alzheimer's Association**, offer workshops and resources to help caregivers learn how to manage symptoms and plan for the future.<br><br>**Resources**<br><br>● **Alzheimer's Association (24/7 Helpline)**: Call **1-800-272-3900** for advice and support.<br>● **Community Resource Finder**: www.communityresourcefinder.org to locate memory specialists and caregiver services near you.<br>● **Inova Memory Disorders Program** (Fairfax, VA): Offers comprehensive evaluations for memory issues.<br>● **Aging Together (Virginia)**: Local resources for caregiving and memory support.<br><br>**Resources**<br><br>● **Alzheimer's Association (24/7 Helpline)**: Call **1-800-272-3900** for advice and support.<br>● **Community Resource Finder**: www.communityresourcefinder.org to locate memory specialists and caregiver services near you.<br>● **Inova Memory Disorders Program** (Fairfax, VA): Offers comprehensive evaluations for memory issues.<br>● **Aging Together (Virginia)**: Local resources for caregiving and memory support.<br><br>By being proactive, you can create a supportive environment for your husband and ensure both of you are prepared for whatever the future holds. If you'd like assistance finding specific resources in your area, let me know—I'm here to help!

13. My husband has been showing signs of early-stage dementia, and I'm starting to worry about the future, especially when it comes to making decisions for him if his condition gets worse. I'm not sure how to set up a power of attorney or advance directive, and I'm concerned about the right time to do this. Are there any resources or steps I can take now to make sure we handle legal matters properly and prepare for what might come next?

**Cond1**	**Cond2**
I'm really sorry you're facing such a challenging situation. It's great that you're thinking ahead and planning for the future. Setting up legal documents like a power of attorney and advance directive is an important step in ensuring that your husband's wishes are respected, especially as his condition progresses. Here are some steps and resources that might help you navigate the process:<br><br>1. Power of Attorney (POA)<br>A power of attorney allows someone to make legal and financial decisions on your behalf if you're unable to do so yourself. There are two types of POA you might consider:<br>● Durable Power of Attorney for Health Care: This gives someone the authority to make medical decisions for your husband if he becomes unable to make those decisions himself.<br>● Durable Power of Attorney for Finances: This allows someone to manage financial matters if your husband becomes incapacitated.<br>When to set it up: Ideally, you should set these up before the condition worsens, when your husband can still participate in the decision-making process and express his wishes. If he's already having trouble with memory or cognition, consider acting soon.<br>2. Advance Directive<br>An advance directive (also called a living will) provides instructions about the kind of medical care your husband would want in the event he can no longer communicate his preferences. This could include decisions on life support, resuscitation, or palliative care.<br>When to set it up: It's best to do this early on when your husband can still make his preferences clear. Some states combine this with the healthcare power of attorney into one document, while others keep them separate.<br>3. Living Will<br>This is a specific type of advance directive that outlines specific medical procedures your husband would want or not want in certain situations, like end-of-life care.<br>When to set it up: As with the POA, it should be done while your husband is still capable of making his wishes known. This way, there's no confusion later.<br>4. Consult an Attorney	It's wise to start thinking about legal and financial planning early when your husband is still in the early stages of dementia and can participate in decisions. Setting up documents like a power of attorney (POA) and advance directives is crucial for ensuring his wishes are honored as his condition progresses. Here's how to get started:<br><br>**1. Understanding Key Legal Documents**<br><br>These are the primary legal tools to consider:<br><br>● **Durable Power of Attorney (POA)**:<br>o Assigns someone (e.g., you) the authority to make financial or legal decisions on his behalf. A *durable* POA remains valid even if your husband loses capacity to make decisions.<br>● **Healthcare Power of Attorney**:<br>o Authorizes someone to make medical decisions if he cannot express his wishes.<br>● **Advance Healthcare Directive**:<br>o Outlines his preferences for medical care, including life-sustaining treatments, resuscitation, and end-of-life care.<br>● **Living Will**:<br>o A written statement specifying medical treatments he does or doesn't want in case of a terminal illness or severe incapacitation.<br>● **Will**:<br>o Specifies how his assets should be distributed after his death.<br>● **Revocable Living Trust (Optional)**:<br>o Allows assets to be managed for his benefit while he's alive and facilitates transfer of assets after his death without going through probate.<br><br>**2. Timing: Why Act Now?**<br><br>● While your husband is in the early stages of dementia, he is likely still legally competent to understand and sign documents.<br>● Waiting too long may result in his inability to participate, which could require a costly and

Because laws around POA and advance directives can vary by state, it's often helpful to consult with an attorney who specializes in elder law. They can help ensure that all the paperwork is valid and in line with your state's laws. They can also guide you in selecting the right person to serve as the agent in both financial and healthcare decisions.

5. Discussing It with Your Husband

Although it can be difficult, it's important to have a conversation with your husband about his wishes for the future. This is especially crucial if there's any possibility he could become unable to make decisions later. If he's in the early stages of dementia, now might still be a good time for him to have some input.

6. Resources

- Alzheimer's Association: They provide resources on legal and financial planning, including advance directives and powers of attorney. You can find guides and even tools to help with decision-making.
- National Institute on Aging (NIA): The NIA offers advice on setting up legal and financial plans as well as guidance on long-term care.
- Aging Life Care Association: This group offers support from professionals who can help with legal, medical, and financial planning for dementia care.

7. Financial and Long-Term Care Planning

In addition to powers of attorney and advance directives, you may want to consider how to handle your husband's finances, especially if he's unable to manage them. Some families set up trust funds or other strategies to safeguard assets.

Next Steps:

- Talk to your husband about his wishes and document them.
- Consult with an attorney who specializes in elder law.
- Set up the appropriate legal documents (POA, advance directive, living will).
- Reach out to resources like the Alzheimer's Association to learn more about financial and long-term care planning.

It's understandable to feel overwhelmed, but taking these steps now can provide peace of mind and make sure your husband's wishes are honored as his condition progresses.

time-consuming court process to appoint a guardian or conservator.

3. Steps to Take

A. Talk with Your Husband

- Have an open and calm discussion about his wishes for the future. Ensure he understands the purpose of these documents and that they reflect his preferences.

B. Consult an Elder Law Attorney

- Work with an attorney who specializes in elder law or estate planning, as they understand the complexities of dementia-related cases.
- They can ensure that documents are legally sound and meet state-specific requirements (e.g., Virginia law).

C. Prepare Necessary Documentation

- Gather essential information:
    - List of assets, income, debts, and insurance policies.
    - Names of trusted individuals to serve as agents or decision-makers.

D. Create and Execute the Documents

- Sign the documents in accordance with your state's requirements. Most require signatures to be witnessed and/or notarized.
- Store these documents in a safe but accessible location and share copies with relevant parties (e.g., doctors, financial institutions, family members).

4. Planning for Future Care

A. Capacity Evaluation

- To ensure validity, you may want a doctor to assess and document your husband's mental capacity before signing legal documents.

B. Financial Planning

- Review his current financial situation, including savings, investments, and insurance policies.

	• Consider **long-term care insurance** or Medicaid planning (if applicable) to prepare for future caregiving costs.<br><br>**C. Explore Local Resources**<br><br>• Contact the **Alzheimer's Association** or **Aging Together** (Virginia-based organization) for guidance on legal and financial planning.<br>• Many local Area Agencies on Aging offer free workshops or consultations on elder law.<br><br>**5. Support Resources**<br><br>Here are organizations and tools that can help:<br><br>• **Alzheimer's Association**:<br>○ Offers free guidance on planning for legal, financial, and caregiving matters. Call their 24/7 helpline at **1-800-272-3900**.<br>○ Legal and Financial Planning Resources.<br>• **Virginia Legal Aid**:<br>○ Provides low-cost or free legal services for eligible individuals. Visit Virginia Legal Aid.<br>• **National Academy of Elder Law Attorneys (NAELA)**:<br>○ Find a local elder law attorney at www.naela.org.<br>• **Fairfax County Area Agency on Aging**:<br>○ Offers resources and referrals for caregivers, including legal aid. Contact them at **703-324-7948** or visit their website.<br>• **Virginia Advance Directives Toolkit**:<br>○ Free tools to create advance directives. Available through the **Virginia State Bar** at www.vsb.org.<br><br>**6. Additional Tips**<br><br>• **Communicate with Family**:<br>○ Share your plans with trusted family members to avoid misunderstandings later.<br>• **Revisit the Plans**:<br>○ Regularly review these documents to ensure they still align with your husband's wishes and your family's circumstances.<br>• **Prepare for Emergencies**:

	◦ Keep a list of emergency contacts, medications, and important documents readily available.<br><br>By addressing these matters now, you'll reduce stress in the future and ensure that your husband's preferences are respected. If you'd like help finding an elder law attorney or other resources, let me know—I'd be happy to assist.

14. My husband wasn't like this before, but now he often gets confused and loses his temper, insisting on things that don't make sense. What should I do if he becomes agitated or confused like this, and how can I handle it without making things worse?

**Cond1**	**Cond2**
I'm really sorry you're experiencing this, and it's understandable that this situation must be quite stressful for both of you. If your husband is showing signs of confusion and irritability that weren't present before, it could be a sign of an underlying issue, such as a medical condition, cognitive decline, or emotional distress. Here are some steps you can take to handle these moments with care and avoid making the situation worse:<br><br>1. Stay Calm and Grounded<br>● When your husband gets agitated or confused, try to remain calm yourself. Your calmness can help de-escalate the situation.<br>● Speak slowly and gently, and try to avoid raising your voice or becoming defensive. Your tone and body language can have a big impact on how he reacts.<br>2. Validate His Feelings<br>● Instead of arguing with him about what he is saying or insisting on logic, try validating his feelings. For example, you can say, "I can see you're upset," or "It sounds like you're feeling confused, and I understand that."<br>● Validation helps to acknowledge his emotional state, which can reduce feelings of frustration or helplessness.<br>3. Avoid Correcting or Confronting His Thoughts<br>● If he's insisting on something that doesn't make sense or is confused, it's usually best not to try to correct him directly. This could cause further agitation or distress. Instead, gently redirect the conversation to a different topic, or calmly offer reassurance without directly contradicting him.<br>4. Check for Medical Causes<br>● If this change in behavior is recent, consider consulting a healthcare professional. Confusion, agitation, and mood changes can sometimes indicate a medical issue, such as a cognitive disorder, medication side effects, or a mental health condition.<br>● A doctor can help rule out any physical or neurological causes, and they may provide specific advice or treatments.<br>5. Use Reassurance and Distraction<br>● In moments when confusion or agitation is overwhelming, try distracting him with something	I'm really sorry you're facing this with your husband. It's tough to see someone you love go through changes, especially when they result in confusion or agitation. When someone with dementia becomes confused or upset, it's important to respond with patience and understanding. Here are some strategies that might help:<br><br>**1. Stay Calm**<br><br>● **Maintain Your Composure**: When your husband becomes agitated, try to remain calm. Take deep breaths and avoid raising your voice. This helps set a reassuring tone, even if you're feeling frustrated.<br>● **Body Language**: Make sure your posture is open and non-threatening. Sometimes, simply sitting down at his level and making eye contact can help him feel more at ease.<br><br>**2. Acknowledge His Feelings**<br><br>● **Validate His Emotions**: It's not necessary to agree with what he's saying, but acknowledge his feelings. For example, if he's confused about something or insists on something that doesn't make sense, say, "I can see that you're upset," or "I understand that this is important to you."<br>● **Avoid Arguing**: If he insists on something that isn't true, like an idea or memory, try not to argue. Instead, gently redirect his attention or reassure him. Arguing can increase agitation and frustration.<br><br>**3. Provide Reassurance**<br><br>● **Gentle Reassurance**: Let him know you're there for him. For example, "I'm here, and everything is going to be okay" or "We'll figure this out together."<br>● **Comfort**: Sometimes a gentle touch on the shoulder or holding his hand can be reassuring if he's open to it. If he's upset, you can also try a calming phrase, like "Let's take a deep breath together."<br><br>**4. Use Simple Language**

calming, like a favorite activity, a memory, or a pleasant topic.<br>● Reassurance can be very effective. Gently remind him that everything is okay and that you're there to help.<br>6. Establish Routine and Familiarity<br>● If possible, try to maintain a consistent routine in daily life, as this can be grounding for someone who is confused or agitated.<br>● Familiar surroundings and activities that bring comfort can help reduce feelings of uncertainty.<br>7. Seek Support<br>● Don't hesitate to lean on support from family, friends, or a therapist. It can be emotionally exhausting to navigate these situations, and having a support system is important for your well-being as well.<br>● Consider looking into support groups for caregivers, which can offer advice and emotional support from others in similar situations.<br>It's important to remember that you're doing your best in a challenging situation, and it's okay to ask for help.	● **Clear and Simple Instructions**: If he's confused, use clear, simple language and short sentences. Avoid overwhelming him with too much information at once.<br>● **Repeat if Necessary**: If he forgets something or becomes fixated on an idea, gently repeat the information in a calm, soothing tone. Be patient, as repetition is often necessary for someone with dementia.<br><br>**5. Avoid Overstimulation**<br><br>● **Limit External Distractions**: If there's a lot of noise, bright lights, or other people, it could be contributing to his confusion or agitation. Try to reduce distractions by moving to a quieter space.<br>● **Create a Calm Environment**: Play soft music, dim the lights, or give him a familiar object (like a favorite blanket) to help him feel safe.<br><br>**6. Distraction Techniques**<br><br>● **Redirect Attention**: Gently steer him away from the topic that's causing distress. You could suggest a calming activity like watching a favorite show, going for a short walk, or engaging in a simple task like folding towels.<br>● **Engage Him in a Familiar Routine**: Many people with dementia find comfort in routine. If he's upset, try guiding him through a task or activity he enjoys, like drawing, gardening, or looking at old photos.<br><br>**7. Address Physical Needs**<br><br>● **Check for Basic Needs**: Sometimes agitation can stem from physical discomfort (e.g., hunger, thirst, needing the bathroom). Make sure he's comfortable and well cared for in terms of basic needs.<br>● **Pain or Illness**: If the confusion or agitation seems out of character, it could be due to pain or illness. It might be a good idea to check with his healthcare provider to rule out any underlying issues.<br><br>**8. Involve a Professional**<br><br>● **Talk to His Healthcare Provider**: It's essential to involve his doctor if his agitation and confusion are becoming more frequent or intense. The doctor can assess whether

	medication adjustments, therapies, or additional interventions are needed.<br>● **Cognitive Behavioral Therapy (CBT)**: Some caregivers find that Cognitive Behavioral Therapy helps with managing behaviors. It could be worth exploring with a professional who specializes in dementia.<br><br>**9. Take Care of Yourself**<br><br>● **Self-Care**: Caring for a loved one with dementia can be emotionally and physically exhausting. Make sure you're also looking after your well-being, whether that means taking breaks, seeking support from other caregivers, or accessing respite care services.<br><br>**10. Consider Support Resources**<br><br>● **Alzheimer's Association Helpline**: If you need advice or just someone to talk to, the Alzheimer's Association helpline is available at **1-800-272-3900**.<br>● **Support Groups**: Connecting with others who understand what you're going through can be really helpful. Look for local or online support groups for caregivers.<br><br>**Additional Resources**<br><br>● **Caring for Someone with Dementia: Tips for Family Caregivers**: The Alzheimer's Association offers free educational materials to help caregivers understand dementia and learn how to manage challenging behaviors.<br>● **Sundowning**: Many individuals with dementia experience worsening symptoms in the evening, a phenomenon known as "sundowning." Learning more about this and its potential causes can help you plan strategies to manage this time of day.<br><br>Dealing with agitation and confusion in dementia can be really challenging, but using these strategies consistently can help keep your husband calm and secure. Remember, it's important to get support for yourself as well. You don't have to do it all alone. Let me know if you need more resources or want to talk about any particular strategies in more detail.

Domain 4 – Copying Style

1. I would like to connect with someone who is experiencing similar situations. I feel overwhelmed as I am responsible for taking care of both my children and my father at home. How can I ensure I take care of myself as well?

Cond1	Cond2
It sounds like you're juggling a lot of responsibilities, and it's understandable that you'd feel overwhelmed. Taking care of both your children and your father is a big task, and it's important to acknowledge the weight of that responsibility. It's just as important, though, to find ways to take care of yourself. Here are some suggestions that may help you balance everything and ensure you're looking after your own well-being:<br><br>1. Set Boundaries and Prioritize Self-Care<br>● Recognize your needs: Taking care of your own physical and mental health is crucial. Try to make time for activities that recharge you, even if it's something small like a quiet cup of tea, a walk, or a few minutes of meditation.<br>● Set realistic limits: It's okay to acknowledge that you can't do everything perfectly. It's important to be kind to yourself and recognize when you need a break or extra help.<br>2. Ask for Help<br>● Share the load: If possible, reach out to family members, friends, or community support for help. Even if it's just a short break to run errands or have some alone time, it can make a huge difference.<br>● Professional support: You might want to explore professional caregiving services or consider respite care for your father, which can give you a chance to recharge. Local organizations or online forums might offer resources or advice from others in similar situations.<br>3. Connect with Others in Similar Situations<br>● Finding a community of others who understand your challenges can help ease feelings of isolation. Online forums or local caregiver support groups can provide both advice and emotional support from people who are going through similar experiences.<br>● Sometimes just talking to someone who "gets it" can make a world of difference.<br>4. Schedule "Me Time"<br>● Try to carve out time for yourself on a regular basis. This doesn't need to be long—just 15-30 minutes can help. This time could be spent doing something you enjoy, like reading, exercising, or	It sounds like you're managing a lot right now, and it's understandable that you would feel overwhelmed. To connect with others in similar situations, you might consider joining online or local caregiver support groups, where people can share experiences and offer advice. Many caregivers find comfort in knowing they are not alone in their challenges.<br><br>For taking care of yourself, here are a few strategies:<br><br>1. **Set Boundaries**: It's important to recognize your limits and say no when you're reaching your capacity. Don't feel guilty for taking time for yourself.<br>2. **Ask for Help**: Reach out to family members, friends, or professional caregivers to give you some time for self-care.<br>3. **Take Small Breaks**: Even if it's just a short walk or a quiet moment alone, taking breaks throughout the day can help recharge you.<br>4. **Practice Self-Compassion**: Remember that you're doing your best, and it's okay not to be perfect.<br>5. **Prioritize Health**: Make time for healthy habits, like eating well, exercising, and getting enough rest.<br><br>You might also find it helpful to talk to a therapist or counselor who specializes in caregiver stress, as they can provide you with tailored coping strategies. Would you like me to look for support groups or specific resources for you?

just resting. It’s essential for maintaining your energy and focus.<br>5. Manage Stress<br>● Caregiving can be stressful. Practice mindfulness or relaxation techniques to reduce stress, such as deep breathing exercises, yoga, or journaling.<br>● Try breaking your day into manageable segments and tackle tasks one at a time to avoid feeling overwhelmed by the bigger picture.<br>6. Establish a Routine<br>● Having a routine can provide structure, reduce stress, and help you feel more in control. While flexibility is important, having a basic schedule for daily tasks can help ease some of the chaos.<br>● If your children are old enough, you could involve them in daily routines and responsibilities to lighten the load.<br>---<br>If you'd like, I can help you find online support groups or resources specifically tailored to caregivers who balance home life with taking care of others. Would that be helpful?	

2. My mother was recently diagnosed with early-stage dementia by her doctor. I've heard that caregiving for individuals with dementia can be time-consuming and that family members often experience burnout from the heavy responsibility. How can I prepare for my own self-care during this challenging and lengthy journey?

**Cond1**	**Cond2**
I'm sorry to hear about your mother's diagnosis. It's understandable that caregiving can be overwhelming, both emotionally and physically. Prioritizing your self-care is essential to maintain your well-being while supporting her. Here are some tips to help you prepare and protect your own mental and physical health:<br><br>1. Set Boundaries: Establish clear limits on how much time and energy you can devote to caregiving. It's okay to take breaks and ask for help when needed.<br>2. Seek Support: Connect with others who are in similar situations. Joining support groups, either in person or online, can provide a sense of community and allow you to share advice and experiences.<br>3. Delegate Tasks: If possible, share caregiving responsibilities with other family members or hire professional help (like a nurse or home aide) to assist with daily tasks. This will lighten your load.<br>4. Practice Stress-Relief Techniques: Incorporate activities like yoga, meditation, or walking into your routine to help manage stress. These practices can reduce anxiety and improve your overall mental health.<br>5. Regular Exercise and Nutrition: Caring for yourself physically is crucial. Regular exercise can boost your mood and energy, while a balanced diet will help keep you healthy and strong.<br>6. Stay Organized: Caregiving involves managing medical appointments, medications, and daily routines. Keeping track of these things with a planner or digital calendar can help reduce stress and make everything more manageable.<br>7. Make Time for Yourself: Even small breaks for hobbies, relaxation, or simply resting can recharge you. Don't feel guilty about taking time for yourself—your own well-being is just as important.<br>8. Educate Yourself: Understanding dementia and its progression can help you adjust your expectations and care strategies. It may also reduce the fear of the unknown, making you feel more in control.<br>9. Consider Professional Counseling: If you find yourself overwhelmed or struggling emotionally, talking to a counselor can provide you with	Caring for a loved one with dementia can indeed be demanding, both physically and emotionally. To help manage your own well-being throughout this journey, here are a few strategies:<br><br>1. **Set realistic expectations**: Recognize that caregiving can be overwhelming at times. Break down tasks into smaller, manageable steps and be kind to yourself when things don't go as planned.<br>2. **Reach out for support**: Connect with local caregiver support groups or online communities where you can share experiences and get advice from others in similar situations. This can help you feel less isolated.<br>3. **Prioritize self-care**: Regularly take time for yourself, even if it's just for a short break each day. Engage in activities you enjoy, whether it's reading, exercising, or spending time with friends.<br>4. **Delegate tasks**: If possible, get help from other family members, friends, or hire a professional caregiver to give you a respite. This can allow you to rest and recharge.<br>5. **Maintain a routine**: Keeping a predictable schedule for both you and your mother can reduce stress. It can also help your mother feel more comfortable and secure.<br>6. **Learn about dementia**: The more you understand the disease, the better you'll be equipped to handle challenges and changes as they arise. Knowledge helps reduce frustration and increases patience.<br>7. **Focus on what you can control**: There will be times when things feel out of your control, but focusing on small, positive actions can help you regain a sense of agency.<br><br>Local resources, such as the Alzheimer's Association, offer information, guidance, and even counseling services to support caregivers like you. Would you like me to look up some local support groups or resources that might be helpful for you?

strategies for managing stress and emotional strain.<br><br>By taking these steps, you can help ensure you're emotionally and physically prepared to face the challenges ahead, while also ensuring that you're taking care of yourself. If you need specific resources or assistance, feel free to ask.	

3. My mother is becoming more forgetful and sometimes gets confused, but she hasn't seen a doctor for it yet. I don't know where to start to get her the right help, especially with my English being limited. What steps should I take to make sure she gets the right assessment and care, and how can I communicate her symptoms well enough so the doctor understands?

**Cond1**	**Cond2**
I understand that it can be challenging to navigate healthcare when you're concerned about your mother's well-being and face language barriers. Here are some steps you can take to ensure she gets the right assessment and care:<br><br>1. Start with a Family Doctor or General Practitioner (GP)<br>● Book an appointment with her primary care doctor (or a new one if needed) for a basic health checkup. You can explain that you're concerned about her forgetfulness and confusion, which could be early signs of a cognitive condition.<br>● Language assistance: Ask the doctor's office if they have translation services available. Many clinics offer support in different languages, and having an interpreter can help make communication easier.<br>2. Describe the Symptoms Clearly<br>● Keep track of her symptoms: Before the appointment, try to note when the confusion or memory issues started and how they've progressed.<br>● Specific examples: Share specific examples, such as if she forgets names, gets confused about time or place, repeats herself, or has difficulty following conversations.<br>● Changes in behavior: Mention any behavioral or mood changes, like irritability, withdrawal, or anxiety.<br>● Daily life impact: Let the doctor know how these issues are affecting her daily life, like problems with cooking, managing finances, or taking care of herself.<br>3. Cognitive Assessment<br>● A doctor will likely perform a basic cognitive test, ask about her medical history, and may refer her to a specialist, such as a neurologist or geriatrician, for further testing.<br>● Request further tests: If the GP doesn't mention it, ask about testing for conditions like Alzheimer's or other forms of dementia, which may require brain imaging or blood tests.<br>4. Ensure Follow-up	It can be challenging when you're concerned about a loved one's cognitive health, especially when language barriers make it harder to communicate effectively with doctors. Here are steps you can take to ensure your mother gets the right assessment and care:<br><br>**1. Find a Healthcare Provider Who Speaks Your Language:**<br><br>Look for doctors, particularly neurologists or geriatricians, who are fluent in your language. This will help ensure that you can communicate your concerns and your mother's symptoms accurately. If you're in a community with a significant number of speakers of your language, there may be local clinics or medical centers with bilingual staff.<br><br>● **Online Resources**: Websites like the Alzheimer's Association's Community Resource Finder can help you search for providers in your area who speak your language.<br>● **Medical Translation Services**: Many hospitals and clinics offer translation services. When you schedule an appointment, ask if they can provide a translator.<br><br>**2. Schedule an Appointment with a Specialist:**<br><br>● A neurologist, geriatrician, or even a primary care doctor can assess your mother's cognitive function. You can ask your doctor to refer you to a specialist if necessary.<br>● Ensure you explain the concerns you have about memory loss and confusion. Bring along any specific examples of behavior or symptoms that you've observed to help the doctor understand the situation.<br><br>**3. Keep Track of Symptoms:**<br><br>● Write down specific symptoms, changes in behavior, and any concerns you have about your mother's cognitive function. This can help the doctor understand her condition.<br>● Note when these issues occur, whether they are frequent or intermittent, and if there is

● If the doctor recommends a specialist, help her make an appointment and follow up on any prescribed tests.<br>● Stay involved in her care by attending appointments if possible or having someone else go with her.<br>5. Explore Support Services<br>● Depending on where you live, there may be community resources to help with language barriers, memory support, or caregiver assistance. This could include local support groups, social workers, or caregiver networks.<br><br>If you'd like help with any specific terms or questions when communicating with the doctor, feel free to let me know! I can help you prepare or translate things to make the process smoother.	anything that seems to trigger the confusion or forgetfulness.<br><br>**4. Prepare for the Appointment:**<br><br>● Bring a family member or trusted friend who is fluent in both English and your language to the appointment, if possible. This can ensure that all concerns are clearly communicated.<br>● If you're on your own, let the doctor's office know ahead of time that you will need language assistance.<br><br>**5. Getting a Diagnosis:**<br><br>● After a doctor evaluates your mother, they may recommend tests to determine the cause of her memory problems. These tests could include cognitive assessments, blood tests, and brain imaging scans.<br>● Based on the results, the doctor will determine whether the memory issues are related to aging, early-stage dementia, or another medical condition.<br><br>**6. Discuss Care Plans:**<br><br>● Once a diagnosis is made, you can ask about treatment options, support services, and resources for caregivers. It's important to plan ahead to ensure your mother receives the right care.<br>● Ask for recommendations for local support groups, dementia services, or community resources available in your language.<br><br>**7. Emotional Support:**<br><br>● Caring for a loved one with cognitive decline can be emotionally taxing. Reach out to local or online support groups where other caregivers, particularly those from similar cultural backgrounds, can offer guidance and empathy.<br><br>Would you like me to assist in finding a healthcare provider in your area who speaks your language or explore any other resources to help you navigate this process?

4. I'm taking care of my partner and don't know much about the U.S. system. I'm not sure how Medicare or Medicaid works for her, and I'm worried about finding the right help. What should I do to start getting the care and support we need, especially in Northern Virginia?

**Cond1**	**Cond2**
I understand how overwhelming navigating the healthcare system can be, especially when you're caring for someone. Here's an overview of how Medicare and Medicaid work, and how you can access the help you need in Northern Virginia:<br><br>**Medicare**<br><br>Medicare is a federal health insurance program for people aged 65 or older and for certain younger people with disabilities. If your partner qualifies for Medicare:<br>● Part A (Hospital Insurance) covers inpatient hospital stays, skilled nursing facility care, and some home health care.<br>● Part B (Medical Insurance) covers outpatient care, doctor visits, preventive services, and other healthcare services.<br>● Part D (Prescription Drug Coverage) helps with the cost of prescription medications.<br>● Medicare Advantage (Part C) is an alternative to Original Medicare, combining Part A and Part B and sometimes Part D, offered by private insurance companies.<br>If your partner is eligible for Medicare, you can enroll them through the Social Security Administration.<br><br>**Medicaid**<br><br>Medicaid is a joint federal and state program that helps with healthcare costs for people with low income. Each state has different eligibility criteria and benefits, so in Virginia, it will depend on factors like income, assets, and whether your partner has any disabilities or other qualifying conditions.<br><br>To apply for Medicaid in Virginia, you can visit the Virginia Department of Medical Assistance Services (DMAS) website or apply through CommonHelp, which is Virginia's online application system.<br><br>**Steps to Take for Care and Support in Northern Virginia**<br>1. Assess Eligibility: First, determine if your partner qualifies for Medicare, Medicaid, or both. For Medicare, eligibility is based on age (65+) or disability. For Medicaid, you'll need to check Virginia's income and asset limits.	Navigating the U.S. healthcare system can be overwhelming, especially when you're caring for a loved one with medical needs. Here's a step-by-step guide to help you understand Medicare, Medicaid, and how to access the right care and support in Northern Virginia.<br><br>**1. Understanding Medicare and Medicaid**<br><br>**Medicare**:<br><br>● Medicare is a federal health insurance program available to people over 65, and also to some younger people with disabilities. There are different parts of Medicare:<br>o **Part A** covers hospital services.<br>o **Part B** covers outpatient services, like doctor visits and some therapies.<br>o **Part D** covers prescription medications.<br>o **Part C** (also called Medicare Advantage) is an alternative to the traditional Medicare plan and may offer additional services, like vision, dental, and hearing coverage.<br>● If your partner is eligible for Medicare, the next step is determining whether the coverage meets her healthcare needs or if supplemental insurance is needed.<br><br>**Medicaid**:<br><br>● Medicaid is a joint federal and state program that helps with medical costs for individuals and families with limited income. Eligibility for Medicaid in Virginia depends on income, assets, and health needs.<br>● If your partner qualifies for Medicaid, it can help cover long-term care costs, which are often not fully covered by Medicare.<br>● Medicaid also helps pay for personal care services, home health care, and nursing home care, if needed.<br><br>**Next Step**: If you're unsure about your partner's eligibility for Medicare or Medicaid, you can start by

2. Contact Virginia's Medicaid Office: Visit the CommonHelp website to start the Medicaid application process. You can also call the Virginia Medicaid Helpline at (800) 552-8627 for assistance.<br>3. Get Assistance with Applications: If you're unsure about navigating the system, you can seek help from local Area Agencies on Aging (AAA) or Medicaid Assistance Programs that help with enrollment. In Northern Virginia, the Northern Virginia Long-Term Care Ombudsman can provide advice and support for caregiving.<br>4. Look into Local Services: If your partner needs additional help, look into home health agencies or adult day care centers in your area. Many offer services that can support your caregiving, such as personal care, therapy, and respite care.<br>5. Contact Medicare Services: If your partner already has Medicare or you're trying to get information about Medicare Advantage plans, you can call 1-800-MEDICARE or visit their website.<br>6. Inquire About Caregiver Support: Many organizations in Northern Virginia, like the Caregiver Action Network and The Family Caregiver Alliance, offer free resources and local support groups.<br><br>Would you like more specific details about any of these steps or need help finding resources in your area?	contacting the **Virginia Medicaid** office or visiting their website.<br><br>**2. Navigating Long-Term Care Options**<br><br>● **In-Home Care**: If your partner needs help with daily tasks, like bathing, dressing, and meal preparation, **Medicaid** might cover home health services, depending on eligibility. These services could be provided by personal care aides or skilled nursing.<br>● **Adult Day Care**: Some adult day centers offer care during the day, helping with social activities, personal care, and supervision. Check with local centers in Northern Virginia to see if they offer Medicaid-eligible services.<br>● **Nursing Homes**: If your partner's condition requires more intensive care, you can explore nursing homes or assisted living facilities. Medicaid may cover long-term stays if certain eligibility requirements are met.<br><br>**3. Finding Support and Resources in Northern Virginia**<br><br>● **Aging and Disability Services**: The **Virginia Department for Aging and Rehabilitative Services (DARS)** is an excellent resource for caregivers. They can help you understand the services and programs available to support your partner's needs, including home care, adult day services, and caregiver support. You can visit their website for local resources: DARS Virginia.<br>● **Area Agencies on Aging (AAA)**: The **Northern Virginia Aging Network (NVAN)** offers support and resources for older adults and caregivers. They provide information on adult day services, caregiver support, and financial assistance programs.<br>o **NVAN Contact**: You can contact them at (703) 324-7948 for more information on services available in your area.<br>● **Alzheimer's and Dementia Services**: If your partner is dealing with cognitive decline, you can reach out to the **Alzheimer's Association, National Capital Area** (NCA). They offer resources in Spanish, provide support for caregivers, and can help connect you with local services.

	○ **Contact**: Call their helpline at 800-272-3900, or visit their website for local resources: Alzheimer's Association NCA.<br><br>**4. Getting Help with Financial and Legal Matters**<br><br>● **Legal Assistance**: It's important to establish a **power of attorney** and **advance directive** for healthcare decisions if your partner hasn't done so already. This ensures that you can make medical decisions on their behalf if necessary.<br>● **Financial Assistance**: If you're struggling with finances, consider reaching out to nonprofit organizations in Northern Virginia that help caregivers manage costs. Many offer assistance with respite care, financial advice, and more.<br><br>**5. Utilizing Caregiver Support Groups**<br><br>● Being a caregiver can be emotionally challenging, and it's important to find emotional and practical support. There are numerous caregiver support groups in Northern Virginia, and some may offer services in Spanish or other languages. These groups can provide you with practical advice, empathy, and resources for dealing with the daily stresses of caregiving.<br><br>**6. Key Resources in Northern Virginia:**<br><br>● **Northern Virginia Family Services**: Offers caregiver support, respite care, and financial assistance programs.<br>● **Home Instead Senior Care**: Provides non-medical in-home care for individuals with dementia, and they have local offices in Northern Virginia.<br>● **Virginia Caregiver Coalition**: A statewide initiative that helps caregivers access respite care, financial advice, and emotional support. You can visit their website for more details.<br><br>Would you like help finding a specific resource or exploring more about applying for Medicare/Medicaid in your area? Let me know, and I can assist further!

5. It’s really hard for me to see my mom becoming more forgetful, and I worry her condition might get worse. Watching her lose herself is so sad, and as an only child, I often feel burdened and guilty about my emotions. Aging seems to happen so fast—how can I cope with these feelings of guilt and burden, while still providing the best care for her?

<table>
<tr><th>Cond1</th><th>Cond2</th></tr>
<tr>
<td>
I'm really sorry you're going through this. It’s completely normal to feel overwhelmed, guilty, and even sad when caring for a loved one, especially a parent. Watching your mom’s condition change can feel like a profound loss, and it's okay to grieve for who she once was, even as you care for her in the present.

Here are a few ways to cope with those feelings of guilt and burden, while also providing the best care for her:

1. Acknowledge your feelings: It’s important to validate your own emotions. Feeling guilty or burdened doesn’t mean you’re not doing your best. In fact, it shows how much you care. Allow yourself to grieve, and remember that caregiving is an emotional journey, not just a physical one.
2. Seek support: Caring for someone, especially as an only child, can feel isolating. Reach out to support groups for caregivers or talk to friends or family members, even if they can’t offer direct help. Sometimes, just sharing your thoughts can lighten the load.
3. Set boundaries: It’s okay to take breaks. You can’t pour from an empty cup, and taking time for yourself helps you be a better caregiver. This might mean finding help from a professional caregiver or family members when you need time to recharge.
4. Focus on what you can control: Aging and illness can be unpredictable, but your ability to provide love, comfort, and support is powerful. While it’s painful to see your mom change, your presence is a gift, and any time you can spend with her is meaningful.
5. Professional help and advice: Consider reaching out to a therapist, counselor, or geriatric care manager. These professionals can provide guidance on managing your own feelings and practical advice for caring for a loved one with memory loss or dementia.
6. Allow yourself grace: You’re doing your best, and it’s okay to feel conflicted. Caregiving is challenging, and you are human. Don’t judge
</td>
<td>
I'm so sorry you're feeling this way—it’s completely understandable, and you're not alone in your experience. As a caregiver, especially for a parent with dementia, the emotional toll can be immense. You're carrying a heavy responsibility, and it’s natural to feel a mix of love, sadness, guilt, and even frustration. Here are some ideas to help you manage these emotions and find ways to care for both yourself and your mom:

1. Acknowledge Your Feelings

First and foremost, it’s important to recognize that the feelings you’re having—guilt, sadness, even anger—are completely normal. Caregiving for someone with dementia is an emotionally taxing experience, and it's okay to feel overwhelmed. You’re doing your best, and it’s natural to grieve the changes you’re witnessing in your mom.

● Give Yourself Permission to Feel: You may feel guilt for feeling sad, but it’s okay to grieve. It's not just about losing your mom physically but emotionally as well. Accepting that grieving is part of the process can help reduce feelings of guilt.
● Seek Understanding: Sometimes just talking to others who have been through similar situations can make you feel less isolated. It’s not just about your mom’s condition but about processing your own emotions, too.

2. Take Care of Yourself

To provide the best care for your mom, you also need to take care of yourself. Without self-care, it’s easy to burn out, which would only make it harder to be there for her.

● Set Boundaries: It’s okay to take breaks. If you're feeling overwhelmed, it’s okay to ask for help. Reach out to friends, family, or professional caregivers for assistance, even if just for a short period, so you can rest.
● Find Time for Yourself: Caregiving can be all-consuming, but find small moments of relief to recharge. Whether it's a hobby, a walk, or just a quiet moment alone, doing something for yourself is crucial.
</td>
</tr>
</table>

yourself too harshly—give yourself permission to have bad days or moments of frustration.

7. **Cherish the present:** While it's painful to watch your mom lose herself, try to focus on small moments of connection. Even if her memory is fading, your presence and the love you share can still bring comfort and joy. Take time to appreciate those moments, however fleeting they may feel.

You are not alone in this, and it's okay to reach out for support and take time for yourself as well. Your emotional well-being matters just as much as your mom's care.

- **Consider Respite Care**: Local respite programs or adult day centers can provide temporary care for your mom, giving you a chance to step away, relax, and recharge.

3. Connect with Support

As an only child, you may feel isolated in this journey. Connecting with others who understand what you're going through can offer immense support. Consider seeking help from:

- **Support Groups**: Look for caregiver support groups, either in person or online, that focus on dementia care. These groups provide a safe space where you can share your feelings without fear of judgment. You can find local groups through organizations like the Alzheimer's Association or other caregiving websites.
- **Online Communities**: There are many online forums and Facebook groups where caregivers share advice, offer emotional support, and validate each other's experiences. Sometimes just hearing someone else say, "I understand," can help lighten your emotional load.

4. Understand the Illness

Understanding what's happening with your mom's dementia can help you navigate the process with more clarity and less anxiety. Dementia is a progressive illness, and while that's hard to accept, learning more about the stages and symptoms can make it easier to adjust expectations and reactions.

- **Educate Yourself**: The more you know about dementia, the more prepared you'll be for the changes that come. Read books, attend workshops, or seek out resources from dementia organizations. The Alzheimer's Association, for example, has many tools for caregivers, including how to cope with your own emotions and provide care.

5. Reframe Your Perspective on Guilt

Guilt is often tied to feeling that we're not doing enough or that we're somehow failing. As a caregiver, you might feel guilty about things you can't control, but it's crucial to remind yourself that caregiving is not a one-size-fits-all task. No one can do it perfectly, and it's okay to have bad days.

- **Focus on What You Can Control**: Take pride in the effort you're putting into caring for your mom. No matter how challenging things may get, remember that you're providing love and support, and that in itself is incredibly important.
- **Let Go of the "Perfect Caregiver" Idea**: There is no such thing as a perfect caregiver. Caregiving is full of moments where you'll feel like you're falling short, but being there for your mom and doing your best is what matters.

6. Create New Moments of Connection

Even as your mom's memory declines, you can still create meaningful moments together. These moments, no matter how small, can help alleviate feelings of loss and keep you both engaged.

- **Focus on What She Can Enjoy**: Even if your mom can't remember things for long, activities that bring joy—like looking at old photos, listening to music, or doing a favorite hobby—can still create moments of connection.
- **Focus on Quality of Life**: Even as dementia progresses, maintaining a focus on quality of life—ensuring that your mom feels safe, loved, and valued—can help both of you through the hardest days.

7. Explore Professional Support

If your feelings of guilt and overwhelm become unmanageable, it might help to speak with a counselor or therapist who specializes in caregiver support. Therapy can give you a space to process emotions like guilt and sadness, and offer coping strategies.

8. Get Help with Practical Issues

In terms of managing the practical aspects of caregiving, including financial support, healthcare decisions, and future planning, it might be helpful to talk with a social worker, legal expert, or financial advisor who specializes in elder care. This can reduce some of the burden of decision-making and provide a clearer path forward.

Remember, it's okay to have tough days, and it's okay to need support. You're doing your best, and that's more than enough. Taking care of yourself and acknowledging your emotions will help you continue

	being there for your mom in the most meaningful way possible.
